# Cellulose carbamates *via* transcarbamoylation/transurethanization of methyl carbamates in superbase–acid conjugate ionic liquids†

**DOI:** 10.1039/d4ra04521a

**Published:** 2024-07-22

**Authors:** Aleksandar R. Todorov, Magdalena Dryś, Eva Gazagnaire, Manisha Podder, Ilkka Kilpeläinen

**Affiliations:** a Materials Chemistry Division, Department of Chemistry, University of Helsinki 00560 Helsinki Finland aleksandar.todorov@helsinki.fi; b Natural Resources Institute Finland Latokartanonkaari 9 00790 Helsinki Finland

## Abstract

A sustainable homogeneous transcarbamoylation/transurethanization protocol for cellulose modification with methyl *N*-substituted carbamates was developed. The protocol utilizes the superbase ionic liquid [mTBNH][OAc], not only as a green reaction medium, but also as a promotor of the transformation. This approach allows to obtain different cellulose carbamates with controllable degrees of substitution. The solubility of the obtained materials from the newly developed method was compared with the solubility of materials obtained from the isocyanate approach, where some intrinsic trends were observed.

## Abstract 2

Herein, we present a sustainable homogeneous protocol for cellulose carbamoylation with methyl *N*-substituted carbamates in the presence of superbase ionic liquids.

## Graphical abstract

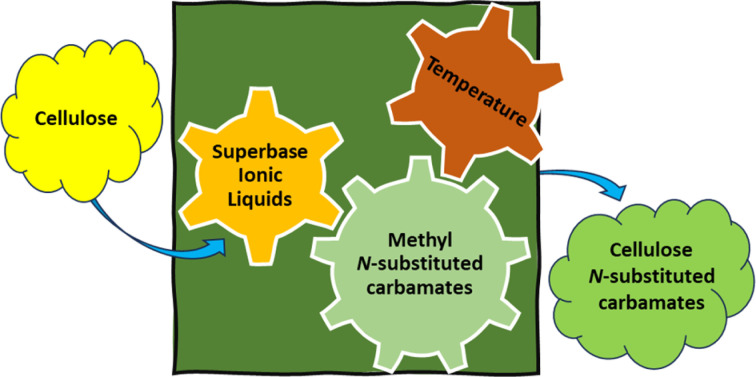

## Introduction

The wood and paper industry produces high amounts of wood products, paper, and cardboard. The main component cellulose is utilized as a material for filters, textiles, and composites, as well as in numerous other applications.^1^ Yet, cellulose's potential is not fully exploited, mainly due to its semi-crystalline nature and the presence of strong intra- and inter-molecular hydrogen-bonds. This peculiarity leads to its insolubility in most of the classic organic solvents and explains the fact that only a handful transformations are known for its efficient chemical modification. As cellulose is a homopolymer of glucose, each of its anhydroglucose units (AGU) carries two secondary and one primary hydroxyl group, which are the main targets for chemical modification reactions. Esterification of the hydroxyl groups is the most common modification used, but more diverse modifications such as oxidations, etherifications, carbonatizations, carbamatizations, *etc.* are also possible.^2–7^

Cellulose carbamate (CC) and its *N*-substituted derivatives (CCDs) are utilized in various applications (note: in this work, the term ‘derivatives’ strictly corresponds to cellulose derivatives with substituted carbamate chain; not to be confused with positional derivatives of cellulose carbamate). For instance, CC is used as starting material in aerogels, thin films, and dye absorbers.^8–10^ In addition, when CC is dissolved and then regenerated in a shape of fibres, it exhibits enhanced stability and improved properties.^11,12^ CC can also be used as an intermediate source for regenerated cellulose fibres (similarly to the cellulose xanthate in the viscose rayon process).^13,14^ One application area of CCDs is their use as a stationary phase for chiral separation columns in high performance liquid chromatography (HPLC). Among them, the most popular derivative is the cellulose 3,5-dimethylphenyl carbamate, commonly known as Chiralcel® OD.^15–18^ Other potential areas of application for CCDs can include food packaging and wound treatment due to the materials good biodegradability and excellent antimicrobial properties.^19–21^ In addition, some CCDs are capable of pH-responsiveness, effective extraction of dyes, or removal of chlorinated phenols.^5,22–25^

Cellulose carbamate and its derivatives can be prepared by direct or indirect approaches, under heterogeneous or homogeneous conditions. Further, several reaction media have been applied, starting from alkaline water/urea mixtures and known cellulose solvents to ionic liquids (ILs) and deep eutectic solvents, or even supercritical carbon dioxide.^5,8,9,19,24,26–33^

**Scheme 1:**
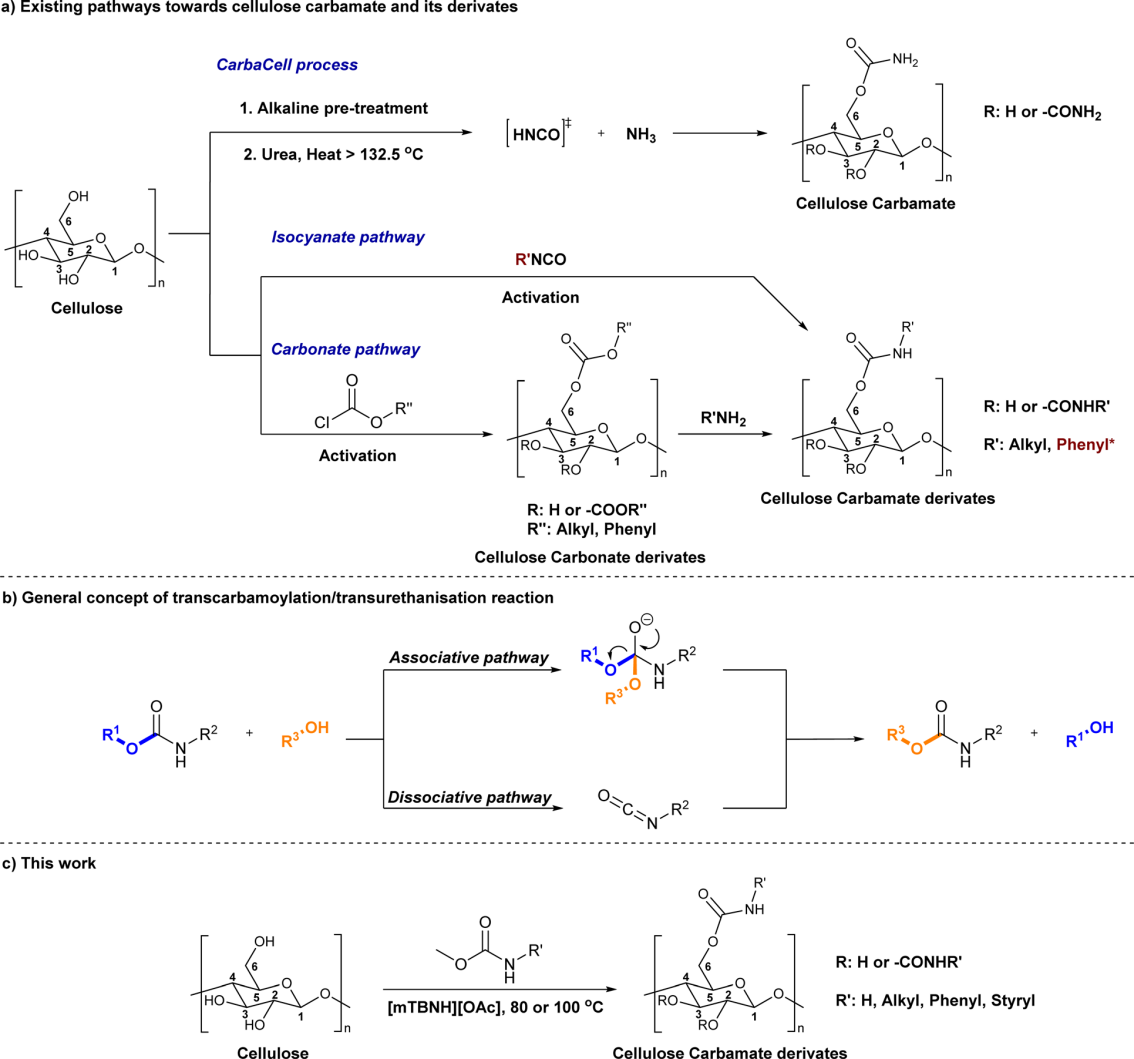
Existing (a), plausible (b), and the newly developed (c) pathways to cellulose carbamate and cellulose carbamate derivatives. * Applies only for the isocyanate pathway.

The direct approach for the preparation of CC, commonly known as the ‘CarbaCell’ process, involves reaction of alkali treated cellulose with urea at temperatures above urea's pyrolysis point (Scheme 1a ‘CarbaCell’ process).^14,34^ The reaction is initiated when urea is heated and releases isocyanic acid, which acts as the reactive intermediate. Despite its simplicity, the method has some possible pitfalls. For the pretreatment step, the method uses rather large amounts of alkaline water. Furthermore, during the pyrolysis, side products are formed (biuret, cyanuric acid, and gaseous ammonia), which may contribute negatively to the environmental impact of the method.^35^ The ‘CarbaCell’ process is currently limited to the preparation of cellulose carbamate, but the preparation of more structurally complex cellulose carbamate derivatives might be possible using different types of urea derivatives as starting compounds.

Replacing the *in situ* generated isocyanic acid with substituted isocyanates allows for the direct preparation of CCDs (Scheme 1a isocyanate pathway). This direct approach to various substituted cellulose carbamates is attractive, but its use is limited to some extent by the commercial availability of the suitable reagents and their toxicity.^36,37^ However, the *in situ* preparation of required isocyanates is possible, but still not thoroughly studied for cellulose modification. Usually, the reaction between unhindered low molecular weight alcohols and isocyanates is rather straightforward. However, the modification of cellulose with isocyanates requires rather harsh conditions (elevated temperature, catalysis, or both), thus jeopardizing the stability of the isocyanate reagent.^18,20,29,38,39^

The two-step indirect approach towards CCDs (Scheme 1a carbonate pathway) includes the preparation and isolation of an active cellulose intermediate.^5,22,23,30,33,40^ In the first step cellulose carbonate is prepared. This is commonly conducted with commercially available chloroformates, though their toxicity and instability under certain conditions should be carefully considered.^5^ The second step includes a nucleophilic attack from the primary amine to the carbonate, followed by alcohol elimination. This specific step is limited to more nucleophilic amines whereas aromatic amines often remain unreactive.^30^ Even if, there is a possibility for cross-linking side reactions, as well as need for preparation, isolation, and purification of the activated cellulose derivatives, the advantages of the process outweighs its disadvantages.^5,30,40^ Overall, this approach should be regarded as a complementary tool and it allows for the synthesis of a wider structural range of CCDs with high degree of substitutions.^33,41,42^

An alternative plausible direct reaction pathway for cellulose could be the transcarbamoylation/transurethanization (TC/TU) reaction with *N*-substituted carbamates (esters of the *N*-phenyl carbamic acid) (Scheme 1b).^43,44^ This particular modification has not been employed for cellulose derivatization so far, but the similarities with the transesterification reaction make it an attractive transformation. Similar to transesterification, TC/TU involves the replacement of the alkoxy functionality in the original *N*-substituted carbamate with another alkoxy group, leading to a new carbamate structure.^45–49^ Basically, the mechanism of this reaction can proceed through an associative or dissociative pathway.^43^ The associative pathway proceeds analogously as in the transesterification mechanism. The selected alcohol attacks the urethane/carbonyl group, followed by simultaneous elimination of the urethane/ester alcohol, generating the desired product. The dissociative pathway proceeds through decomposition of the *N*-substituted carbamate to the corresponding isocyanate. Then the generated isocyanate reacts further with the selected alcohol to form the new product.^43^ Regardless of the reaction pathway, the transfer of the carbamate/urethane functionality requires more energy than in the case of transesterification. The difference between these transformations arises from the fact that the carbamate/urethane carbonyl carbon is much less electrophilic than its ester analogue.^50^ Due to this peculiarity, these transformations are often promoted by acid catalysis, conventional heating, or microwave irradiation.^45–48,51,52^

Particularly interesting finding is that inorganic bases, *e.g.*, sodium hydroxide, potassium hydroxide, or strong organic bases, *e.g.*, 1,5,7-triazabicyclo[4.4.0]dec-5-ene (TBD), potassium *tert*-butoxide, catalyse the TC/TU reaction.^49,50^ In fact, some of the strong organic bases, a.k.a. superbases, are used in the preparation of superbase ionic liquids (SB-ILs), which are known solvents for cellulose.^53–56^ This class of ionic liquids are easy to use, non-toxic and have low vapor pressure. In the field of cellulose chemistry and processing, SB-ILs such as bicyclic guanidine acetates, *e.g.*, 7-methyl-1,5,7-triazabicyclo[4.4.0]dec-5-ene acetate [mTBDH][OAc] and 5/7-methyl-1,5,7-triazabicyclo[4.3.0]non-5-enium acetate [mTBNH][OAc] (mixture of isomers), have attracted significant attention.^53,56^ They have excellent cellulose dissolution capabilities, good recyclability properties and are attractive platform also for chemical modification of cellulose.^57^ Furthermore, as we have shown previously, the superbase-to-acid ratio in these SB-ILs could be altered towards more basic or acidic conditions.^57^ These alterations can provide greater tunability of the reaction conditions and possibly promote the TC/TU cellulose transformation.

**Chart 1:**
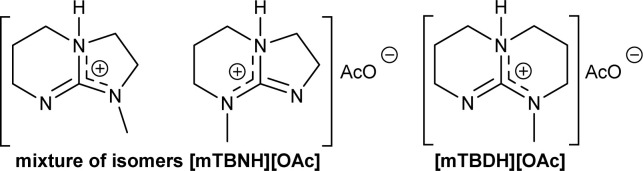
Structural representation of the used SB-ILs in this study.

Herein, we report a novel sustainable, homogeneous procedure for the preparation of cellulose carbamate derivatives with variable degree of substitution (DS), *via* a TC/TU reaction with stable and accessible methyl carbamates (Scheme 1c), utilizing SB-ILs developed in our research group (Chart 1).^58,59^

## Results and discussion

### Reaction optimization

Microcrystalline cellulose (MCC) Avicel® PH-101 purchased from Sigma Aldrich was selected as a model compound to assess the reactivity of cellulose towards TC/TU reactions with methyl *N*-substituted carbamates in the SB-IL [mTBNH][OAc]. Reaction optimization studies were performed separately for aromatic and aliphatic methyl *N*-substituted carbamates, due to the expected differences in their reactivity.^43^ We varied several reaction parameters to find the optimum reaction conditions and achievable DS values (Tables 1 and 2). Namely, we screened the type and amount of reagent, the reaction time and temperature, the composition of the SB-IL (ratio of superbase to acid, Table 3), the SB-IL itself, and the influence of possible co-solvents (note: due to the overall low solvent volumes and high viscosities of the reaction mixtures, resulting in limited possibility for proper stirring, chemically inert co-solvent (gamma-valerolactone) was used during the optimization studies for the aromatic series).

**Table 1:** Reaction parameters variations for aromatic *N*-substituted carbamates^a^

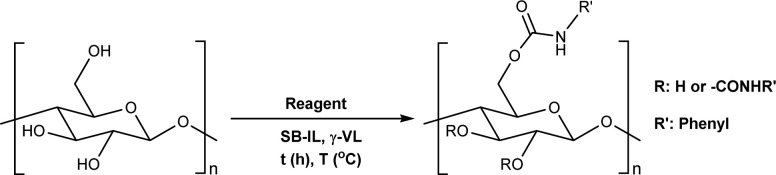					
Entry	Reagent/SB-IL	Equivalents	*T* (°C)	*t* (h)	DS^b^
**Reagent** c					
1	MeOCONHPh	9	80	20	0.18
2	EtOCONHPh	9	80	20	0.11
3	i-PrOCONHPh	9	80	20	0.10
4	*t*-BuOCONHPh	9	80	20	0.11

**Reagent amount (equivalents to AGU)**					
5	MeOCONHPh	3	80	20	0.10
6	MeOCONHPh	6	80	20	0.14
7	MeOCONHPh	9	80	20	0.18
8	MeOCONHPh	12	80	20	0.20
9	MeOCONHPh	15	80	20	0.26

**Reaction time (h)**					
10	MeOCONHPh	9	80	24	0.20
11	MeOCONHPh	9	80	48	0.19
12	MeOCONHPh	9	80	72	0.21

**Reaction temperature (°C) and SB-IL** d					
13	[mTBNH][OAc]	—	60	20	0.07
	γ-VL				
14	[mTBDH][OAc]	—	60	20	0.07
	γ-VL				
15	[mTBNH][OAc]	—	80	20	0.18
	γ-VL				
16	[mTBDH][OAc]	—	80	20	0.14
	γ-VL				
17	[mTBNH][OAc]	—	100	20	0.14
	γ-VL				
18	[mTBDH][OAc]	—	100	20	0.14
	γ-VL				
19	[mTBNH][OAc]	—	120	20	0.07
	γ-VL				
20	[mTBDH][OAc]	—	120	20	0.07
	γ-VL				

a Reactions were performed in sealed 8 mL vials, where the desired amount of reagent was added to 50 mg of MCC dissolved in 1 mL of [mTBNH][OAc] or [mTBDH][OAc] followed by 0.5 mL γ-valerolactone, then the reaction was stirred under the specified conditions.

b DS values are an average of two reactions and were determined by diffusion-edited ^1^H NMR at 65 °C with a standard calibration curve against DS ranges determined under quantitative conditions.

c Methyl *N*-phenylcarbamate (MeOCONHPh), ethyl *N*-phenylcarbamate (EtOCONHPh), iso-propyl *N*-phenylcarbamate (i-PrOCONHPh) and *tert*-butyl *N*-phenylcarbamate (*t*-BuOCONHPh).

d 9 equivalents of MeOCONHPh were used as reagent.

**Table 2:** Reaction parameters variations for aliphatic *N*-substituted carbamates^a^

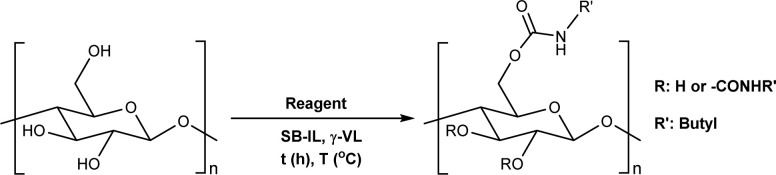					
Entry	Reagent/SB-IL	Equivalents	*T* (°C)	*t* (hours)	DS^b^
**Reagent amount (equivalents to AGU)**					
1	MeOCONHBu	3	120	20	0.10
2	MeOCONHBu	6	120	20	0.16
3	MeOCONHBu	9	120	20	0.23
4	MeOCONHBu	12	120	20	0.32
5	MeOCONHBu	15	120	20	0.36

**Reaction time (hours)**					
6	MeOCONHBu	6	120	24	0.16
7	MeOCONHBu	6	120	48	0.09
8	MeOCONHBu	6	120	72	0.08

**Reaction temperature (°C) and SB-IL** c					
9	[mTBNH][OAc]	—	60	20	0.06
10	[mTBNH][OAc]	—	60	20	0.06
	γ-VL				
11	[mTBDH][OAc]	—	60	20	0.06
	γ-VL				
12	[mTBNH][OAc]	—	80	20	0.09
13	[mTBNH][OAc]	—	80	20	0.08
	γ-VL				
14	[mTBDH][OAc]	—	80	20	0.08
	γ-VL				
15	[mTBNH][OAc]	—	100	20	0.17
16	[mTBNH][OAc]	—	100	20	0.13
	γ-VL				
17	[mTBDH][OAc]	—	100	20	0.13
	γ-VL				
18	[mTBNH][OAc]	—	120	20	0.15
19	[mTBNH][OAc]	—	120	20	0.14
	γ-VL				
20	[mTBDH][OAc]	—	120	20	0.10
	γ-VL				

a Reactions were performed in sealed 8 mL vials, where the desired amount of methyl *N*-butylcarbamate (MeOCONHBu) was added to 50 mg of MCC dissolved in 1 mL of [mTBNH][OAc] or [mTBDH][OAc] with or without addition of 0.5 mL γ-valerolactone, then the reaction was stirred at the specified conditions.

b DS values are an average of two reactions and were determined by diffusion-edited ^1^H NMR at 65 °C with a standard calibration curve against DS ranges, determined under quantitative conditions.

c 6 equivalents of MeOCONHBu were used as reagent.

**Table 3:** Superbase to acid variations of ionic liquid for *N*-substituted carbamates^a^

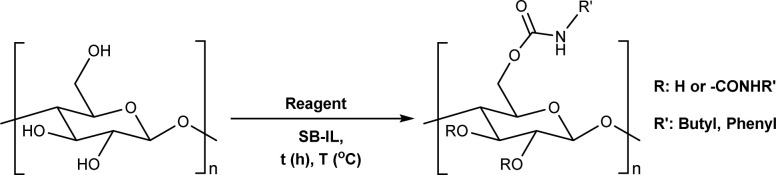					
Entry	SB-IL composition	Equivalents	*T* (°C)	*t* (h)	DS^b^
**MeOCONHPhenyl** c					
1	[mTBNH][OAc]	9	80	20	0.15
	1 : 1.5				
2	[mTBNH][OAc]	9	80	20	0.21
	1 : 1				
3	[mTBNH][OAc]	9	80	20	0.19
	1.5 : 1				
4	[mTBNH][OAc]	9	80	20	0.19
	2 : 1				
5	[mTBNH][OAc]	9	80	20	0.20
	5 : 1				
6	[mTBNH][OAc]	9	80	20	0.21
	10 : 1				

**MeOCONHButyl**					
7	[mTBNH][OAc]	6	120	20	0.11
	1 : 1.5				
8	[mTBNH][OAc]	6	120	20	0.12
	1 : 1				
9	[mTBNH][OAc]	6	120	20	0.10
	1.5 : 1				
10	[mTBNH][OAc]	6	120	20	0.09
	2 : 1				
11	[mTBNH][OAc]	6	120	20	0.08
	5 : 1				
12	[mTBNH][OAc]	6	120	20	0.08
	10 : 1				

a Reactions were performed in sealed 8 mL vials, where the desired amount of methyl *N*-phenylcarbamate (MeOCONHPh) or methyl *N*-butylcarbamate (MeOCONHBu) was added to 50 mg of MCC dissolved in 1 mL of the corresponding [mTBNH][OAc] mixture, then the reaction was stirred at the specified conditions.

b DS values are an average of two reactions and were determined by diffusion-edited ^1^H NMR at 65 °C with a standard calibration curve against DS ranges, determined under quantitative conditions.

c 0.5 mL γ-valerolactone was added as co-solvent to reduce the viscosity of the reaction mixture.

To begin with, we selected different esters of the *N*-phenyl carbamic acid, *e.g.*, methyl, ethyl, i-propyl, and *t*-butyl (Table 1 entries 1–4) to determine their suitability as cellulose carbamoylating reagents. Evidently, from the results, the less bulky reagent methyl *N*-phenylcarbamate (or *N*-phenylcarbamic acid methyl ester, Table 1 entry 1) performed better than the other used reagents providing DS as high as 0.18 in the chosen reaction conditions. In the case of ethyl, i-propyl, and *t*-butyl *N*-phenylcarbamate, we obtained cellulose materials with comparable DS around 0.11, regardless of their growing steric hindrance (Table 1 entries 2–4). These guided us to use methyl *N*-phenylcarbamate or methyl *N*-butylcarbamate for further optimization work.

As expected, with increasing amount of the reagent, we obtained cellulose materials with increasing DS (Tables 1 entries 5–9 and 2 entries 1–5). The results in both (aromatic and aliphatic) series are comparable, but noteworthy the used temperatures are different; 80 °C for aromatic *vs.* 120 °C for aliphatic methyl *N*-substituted carbamates. In general, the observed reactivity differences correspond well with the reactivity order of the carbamate reagents. The order of reactivity is as follows alkyl–OCONH–alkyl < alkyl–OCONH–aryl < aryl–OCONH–alkyl < aryl–OCONH–aryl, where alkyl is an aliphatic substituent and aryl is an aromatic substituent.^43^ The reactivity differences in the series comes naturally from the differences in the mesomeric and inductive effects of the substituents (alkyl *vs.* aryl). Overall, these results allow for greater tunability for the synthesis of targeted cellulose carbamate materials.

Furthermore, quick look at the reaction times provides valuable information not only about the reaction performance, but also on the stability of the products. The formation of the aromatic *N*-substituted cellulose carbamates (Table 1 entries 10–12) remain unaffected from the prolonged reaction times, while the aliphatic *N*-substituted cellulose carbamates (Table 2 entries 6–8) suffer from stability issues, due to the higher temperature used in the transformation. While focusing on the effect of reaction's temperature, we noticed a significant influence over the reaction outcome (Tables 1 entries 13, 15, 17, 19 and 2 entries 9, 12, 15, 18). The aromatic *N*-substituted carbamates demonstrated their best reactivity at 80 °C, while the aliphatic *N*-substituted carbamates required elevated temperature (100 °C). Changes above or below these temperatures affected the DS negatively indicating the limitation of this transformation.

Additionally, we compared the influence of the solvent by utilizing two different SB-ILs. We employed the SB-IL [mTBDH][OAc] similarly as the [mTBNH][OAc]. Due to the higher melting point of [mTBDH][OAc] (82–83 °C), we used gamma-valerolactone as a chemically inert co-solvent. A closer inspection of the experimental data (Tables 1 entries 13–20 and 2 entries 9–20) reveals that both SB-ILs perform equally well. Moreover, we noticed that the added co-solvent does not influence the reaction outcome (Table 2 entries 9–20).

We have earlier studied the transesterification of cellulose with unactivated esters.^57^ In that case, the addition of a superbase leads to an increase of the DS in the prepared cellulose acetates.^57^ Knowing that, we decided to use the same strategy and to test if the addition of acid or base to the reaction mixture will influence the reaction outcome. To study this, we varied the stoichiometry of the used SB-IL [mTBNH][OAc], as presented in Table 3. However, the SB-IL stoichiometry had essentially no effect to the reaction outcome (Table 3). Nevertheless, utilizing the 1 : 1 SB-IL seems to be adequate for this reaction.

### Mechanistic insights

**Scheme 2:**
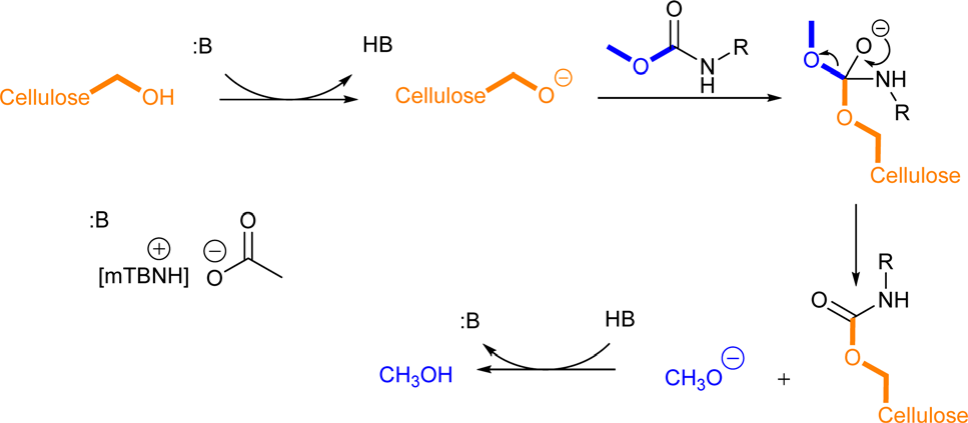
Proposed reaction mechanism for cellulose transcarbamoylation.

Literature suggests four types of mechanistic scenarios for the TC/TU reaction: (1) classical non-catalysed dissociative, (2) acid-, (3) base-, and (4) nucleophile-catalysed associative or dissociative. It is noteworthy to note that in the current case, the third and fourth suggested mechanisms are in fact analogous.^43,56,60,61^ For the classical non-catalysed dissociative pathway to takes place, the reaction temperatures required are well above 200 °C. In our case, the transformation takes place already at 80 to 100 °C and increasing the temperature above these only deteriorated the reaction outcome, which rules out this pathway. At a closer look (Table 3 entry 1, 2, 7 and 8) the addition of acetic acid seems to decrease the formation of the desired product. This implies that the acid catalysed pathway is not likely. The absence of a clear trend in the base catalysed reactions (Table 3 entries 2–6 and 8–12) suggests that the reaction is also not base catalysed. However, one should note that the 1 : 1 SB-IL acetate salts possess basic character themselves and it is plausible that the reaction proceeds through associative nucleophilic attack (Scheme 2). At this stage, the dissociative pathway could not be ruled out either.

**Fig. 1:**
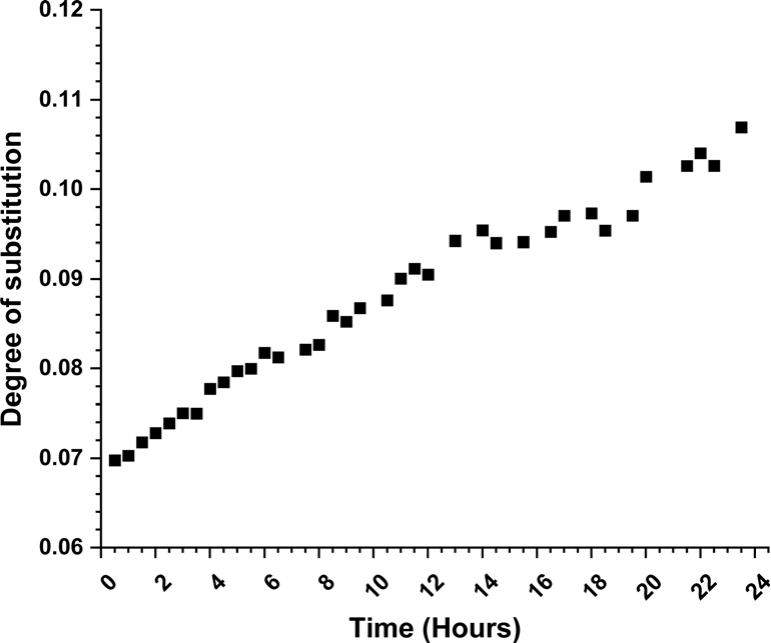
Plot of degree of substitution for cellulose carbamoylation with methyl *N*-phenylcarbamate at 80 °C. The DS values were determined from the diffusion edited ^1^H NMR utilizing a standard calibration curve.

Looking further for plausible clues of the reaction mechanism, we followed the progress of the reaction with diffusion-edited ^1^H NMR. The DS values (Fig. 1) from it were determined utilizing a standard calibration curve (see details in ESI†).

We monitored the increase of DS for 24 hours at 80 °C (experiment details in ESI†). The experiment was done in an NMR tube and in order to reach reasonable diffusion coefficient ranges, we used DMSO-*d*_6_ as an inert co-solvent. Since no mechanical stirring was applied, we can expect some deviations due to the limited reagent diffusion into the reaction mixture. The DS shows almost linear grow throughout the experiment time. The linear increase of the DS is in agreement with the associative nucleophilic attack pathway, but again it was not possible to exclude the dissociative pathway. The achieved DS at the end of the 24 hours is lower than expected (0.11 *vs.* 0.20), which is likely due to the experimental conditions. Due to the NMR instrument limitations, we were not able to perform the experiments at a higher temperature, which might provide us with more mechanistic insights. The same limitation applies for cellulose reactions with methyl *N*-butyl carbamate, where the reaction temperature should be even higher.

### Carbamate formation – TC/TU reaction

To screen the potential of the TC/TU transformation, we prepared cellulose carbamates with variable substituents and DS (Table 4). For this, we used commercially available methyl *N*-substituted carbamates, and the amount of reagent was varied (6, 9, and 12 equivalents). In the case of no commercial availability, the reagents were synthesized using known methods (see ESI† for details). To track if there are any trends in the reactivity of cellulose towards the methyl *N*-substituted carbamates, we selected substrates with varying properties. For the aliphatic substrates we chose such with an increasing *n*-carbon chain (0–4, Table 4 entries 1–5), with cationic functional group (Table 4 entry 6), and with branched chain (Table 4 entries 7–8). For the aromatic substrates, the electronic properties were varied, *i.e.*, electron neutral, electron rich and electron deficient (Table 4 entries 9–11). To evaluate possible side reactions of other functionalities, we included substrates carrying reactive functional groups, such as in the methyl *N*-styryl carbamate (Table 4 entry 12) which in turn enables further modifications (*e.g.*, crosslinking or click chemistry).^62,63^

**Table 4:** Substrate scope for cellulose transcarbamoylation^a^

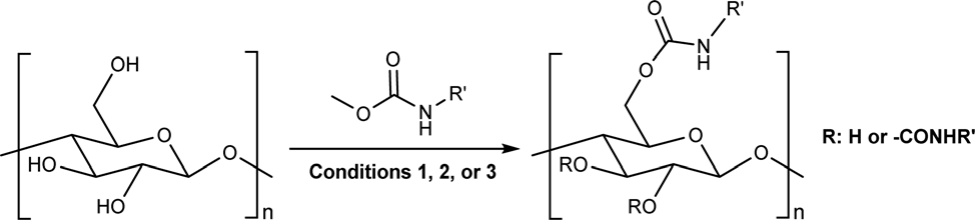			
Entry	R′	DS (condition 1/2/3)	
		^1^H NMR^b^	EA^c^
1	H	0.07/0.11/0.19	0.08/0.10/0.15
2	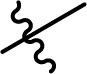	0.09/0.17/0.18	0.08/0.09/0.12
3	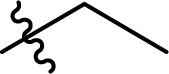	0.13/0.17/0.22	0.09/0.09/0.13
4	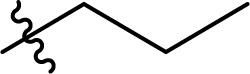	0.10/0.14/0.22	0.09/0.09/0.12
5	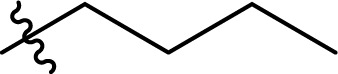	0.13/0.17/0.18	0.05/0.08/0.10
6	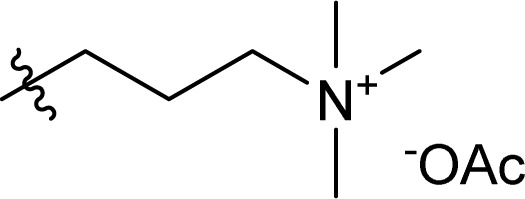	0.10/0.10/0.14	0.15/0.15/0.20
7	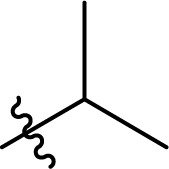	0.04/0.05/0.08	0.06/0.04/0.09
8	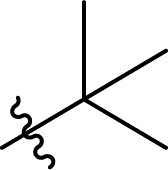	0.04/0.05/0.06	0.05/0.06/0.06
9	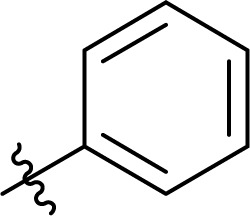	0.14/0.14/0.16	0.07/0.13/0.15
10	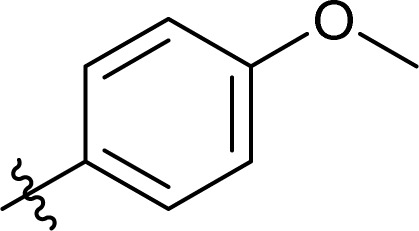	0.09/0.09/0.09	0.09/0.08/0.08
11	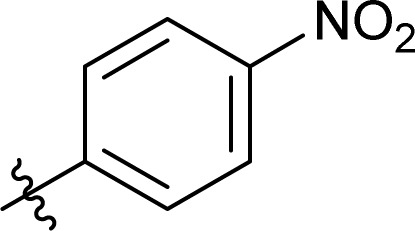	0.05/0.06/0.08	0.03/0.04/0.05
12	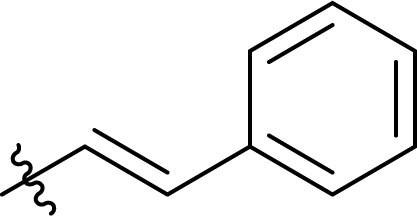	0.10/0.21/0.32	0.11/0.20/0.26

a Reactions were performed in 100 mL round bottom flask, where the corresponding reagent was added to 500 mg of MCC dissolved in 10 mL of [mTBNH][OAc], then the reaction was stirred at 80 °C (aromatic), 90 °C (conjugated) or 100 °C (aliphatic). Condition 1: 6 equivalents of reagent, 20 h reaction time. Condition 2: 9 equivalents of reagent, 20 h reaction time. Condition 3: 12 equivalents of reagent, 20 h reaction time.

b DS of isolated sample determined by ^1^H NMR at 65 °C in DMSO-*d*_6_/LiCl.

c DS of isolated sample determined by nitrogen content from elemental analysis (see ESI for details).

From the results (Table 4 entries 1–5), it is evident that the increasing number of carbon atoms in the aliphatic carbamates did not influence the cellulose TC/TU reaction. With sterically hindered carbamates, expectedly the reaction outcome was affected negatively, *i.e.*, yielding lower DS (Table 4 entries 7–8). On the other hand, the DS of the cationic cellulose carbamate (Table 4 entry 6) was comparable to the other aliphatic cellulose carbamates (Table 4 entries 1–5).

The results from the cellulose TC/TU reaction with the methyl *N*-substituted aromatic carbamates show that the changes in the electron properties influence the reaction outcome (Table 4 entries 9–11). The electron neutral phenyl carbamate gives higher DS compared to the electron rich (4-methoxy) and electron deficient (4-nitro). In comparison, the transesterification of cellulose with electron deficient substrates *e.g.*, methyl 4-nitrophenolate proceeds rather well.^57^ Quite unexpectedly, the corresponding cellulose carbamate formation with methyl *N*-4-nitrophenyl carbamate was considerably lower.

The formation of the cellulose *N*-conjugated carbamate through the TC/TU reaction proceeded well and cellulose materials with variable DS were prepared successfully (Table 4 entry 12).

Controlling the added amount of reagent allowed us to obtained cellulose carbamate materials with variable DS. However, in some cases, the DS of the prepared materials did not increase gradually with the increasing amount of the reagent (Table 4 entries 2, 5–6 and 9–11). This behaviour is likely due to an increasing aggregation of the cellulose (induced by the poorer solvent conditions), or possibly due to aggregation of the reagent itself. For the cationic cellulose carbamate (Table 4 entry 6), the internal electrostatic repulsions of the product may also contribute.

### Carbamate formation – isocyanate reaction

For comparison of using unactivated methyl *N*-substituted carbamates, we decided to prepare some similar cellulose carbamates using the common isocyanate route. For this, phenyl and butyl isocyanates were selected. To obtain comparable results, we kept the same reaction media ([mTBNH][OAc]), time (20 h), and variation (equivalents of regents *vs.* AGU) as for the TC/TU reaction (Table 5).

**Table 5:** Cellulose carbamoylation with isocyanates^a^

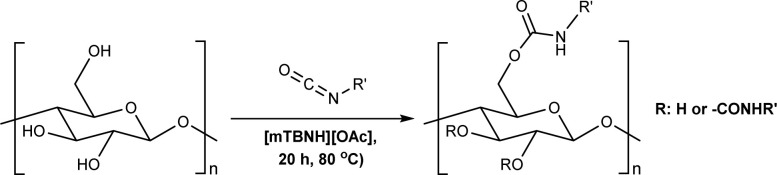					
Entry	R′	Equivalents	*T* (°C)	DS	
				^1^H NMR^b^	EA^c^
1	Phenyl	0.5	80	0.02	0.03
2	Phenyl	1	80	0.03	0.04
3^d^	Phenyl	1	80	0.08	0.07
4	Phenyl	1.5	80	0.07	0.06
5	Phenyl	2	80	0.08	0.08
6	Phenyl	2.5	80	0.10	0.09
7	Phenyl	3	80	0.11	0.11
8^d^	Phenyl	3	80	0.14	0.13
9	Phenyl	3	65	0.12	0.11
10	Phenyl	6	80	0.27	0.24
11^d^	Phenyl	6	80	0.29	0.28
12^d^	Phenyl	9	80	0.51	0.48
13^d^	Phenyl	12	80	0.65	0.74
14	Butyl	0.5	80	0.04	0.04
15	Butyl	1	80	0.11	0.08
16	Butyl	1.5	80	0.14	0.11
17	Butyl	2	80	0.14	0.11
18	Butyl	2.5	80	0.17	0.14
19	Butyl	3	80	0.23	0.16
20	Butyl	6	80	2.53	1.20

a Reactions were performed in 100 mL round bottom flask, where the corresponding amount of reagent was added to 500 mg of MCC dissolved in 10 mL of [mTBNH][OAc], then the reaction was stirred at the corresponding temperature for 20 h.

b DS of isolated sample determined by ^1^H NMR at 65 °C in DMSO-*d*_6_/LiCl.

c DS of isolated sample determined by nitrogen content from elemental analysis (see ESI for details).

d Reactions were performed in 50 mL round bottom flask, where the corresponding amount of reagent was added to 250 mg of MCC dissolved in 5 mL of [mTBNH][OAc], then the reaction was stirred at 80 °C for 20 h.

Somewhat surprisingly, the phenyl isocyanate was only slightly more reactive than the corresponding methyl *N*-phenyl carbamate (Table 5 entry 10 *vs.*Table 4 entry 9 condition 1). To validate this observation, additional experiments with varying reaction conditions were performed. The results from these experiments show that the increased amount of reagent (Table 5 entries 12–13) led to higher DS, which is still unexpectedly low for this activated reagent. One reason for the unexpected reactivity during this transformations could be a plausible side reaction of the used isocyanates with the acetate anion from the SB-IL.^64,65^ Other plausible reason for this behaviour might be the isocyanates side reaction with water, as we did not use water free conditions (note: due to the hygroscopicity of the used SB-ILs, trace amount of water could be present).^66–68^ In effort to limit this, we lower the reaction temperature to 65 °C, but the experiment did not provide any clear trend (Table 5 entries 9 *vs.* 7). The reactions with butyl isocyanate proceed similarly to phenyl isocyanate for up to 3 equivalents of reagent (Table 5 entries 14–19). However, the reaction with butyl isocyanate seems to proceed better with larger excess of the reagent yielding rather highly substituted cellulose (Table 5 entry 20). It seems that in this case the supressed reactivity of the butyl isocyanates is affected from the side reaction of the butyl isocyanate with water rather than the side reaction with the acetate anion. The unusual behaviour and low reactivities of isocyanates in SB-ILs deserve to be investigated in future studies.

**Scheme 3:**
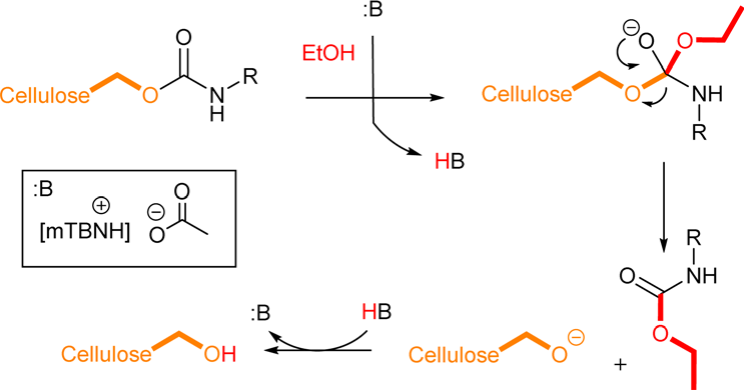
Plausible decomposition of the cellulose carbamates.

These findings prompted us to analyse the crude reaction mixtures further, which pointed out that there were differences arising during the work-up protocol with ethanol (Fig. S3 in ESI†). Evidently, a side reaction can occur, and reversibility of the main reaction is possible, resulting in a decrease of the DS (Scheme 3).

**Fig. 2:**
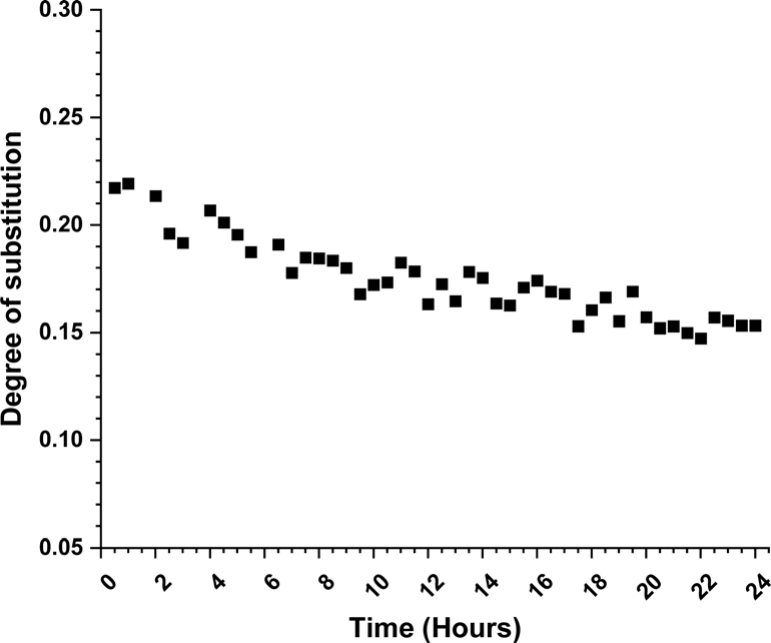
Plot of degree of substitution from NMR experiment for cellulose *N*-phenylcarbamate reacting with EtOH at 80 °C. The DS values were determined from the diffusion edited ^1^H NMR with standard calibration curve.

Indeed, cellulose *N*-phenyl carbamate derivative shows gradually declining DS in the presence of ethanol (Fig. 2, for Experimental details see ESI†). This finding strongly supports the reversibility of the utilized isocyanate reaction through TC/TU pathway.

A closer inspection of the results of the TC/TU reactions (crude reaction mixtures Tables 1 and 2*vs.* isolated materials Table 4) show some fluctuations. However, here we can also attribute these deviations to the small instability of the products in the presence of alcohols (or water). Simplest go around will be to utilize non-nucleophilic work-up (avoiding nucleophilic anti-solvents), but on the other hand, as it is shown in Fig. 2, the exchange reaction is rather slow, and it should not significantly contribute to the DS of the product. As an advantage of the ethanol precipitation/washing is the simultaneous removal of residual IL from the product. Overall, the current workup might not be fully optimal, but it is straightforward and simple. Clearly, there are several contributing factors, and the isolation process itself is certainly a critical step in the preparation of cellulose carbamates.

### Solubility of the cellulose carbamates

One parameter affecting the properties of the cellulose derivatives is the even and selective distribution of the introduced side chains.^69,70^ The distribution itself can be tedious to evaluate, but the solubility of the different types of substitution patterns is affected by the different solubilities of the derivatives with equal DS. Thus, we studied the solubility properties of the cellulose carbamates prepared through the TC/TU and isocyanate reaction. The results from the dissolution experiments are summarized in Tables 6 and S2.†

**Table 6:** Dissolution test of the prepared cellulosic materials^a^^,^^b^

Entry	Cellulose derivative	DS	DMSO	DMA	γ-VL	Cyrene™	H_2_O	1 M AcOH^c^	0.1 M NaOH^c^	1 M NaOH^c^	2 M NaOH^c^
1	Carbamate	0.07	−	−	−	−	−	−	−	*	+
		0.11	−	−	−	−	−	−	−	*	*
		0.19	+	−	−	−	−	−	−	*	*
2	*N*-Methyl carbamate	0.09	−	−	−	−	−	−	−	*	*
		0.17	−	−	−	−	−	−	−	*	*
		0.18	+	−	−	−	−	−	−	*	*
3	*N*-Ethyl carbamate	0.13	−	−	−	−	−	−	−	*	*
		0.17	−	−	−	−	−	−	−	*	*
		0.22	+	−	−	−	−	−	−	*	+
4	*N*-Propyl carbamate	0.10	−	−	−	−	−	−	−	*	*
		0.14	+	−	−	−	−	−	−	*	*
		0.22	+	−	−	−	−	−	−	*	*
5	*N*-Butyl carbamate	0.13	−	−	−	−	−	−	−	*	*
		0.17	−	−	−	−	−	−	−	*	*
		0.18	−	−	−	−	−	−	−	*	+
6	*N*-(*N*,*N*,*N*-Trimethylpropan-1-aminium acetate)carbamate	0.10	−	−	−	−	+	+	−	+	+
		0.10	−	−	−	−	+	+	−	+	+
		0.14	−	−	−	−	+	+	−	+	+
7	*N*-Isopropyl carbamate	0.04	−	−	−	−	−	−	−	*	+
		0.05	−	−	−	−	−	−	−	*	*
		0.08	−	−	−	−	−	−	−	*	+
8	*N-tert-*Butyl carbamate	0.04	−	−	−	−	−	−	−	*	+
		0.05	−	−	−	−	−	−	−	*	+
		0.06	−	−	−	−	−	−	−	*	*
9	*N*-Phenyl carbamate	0.14	−	−	−	−	−	−	−	*	*
		0.14	+	−	−	−	−	−	−	+	+
		0.16	+	−	−	−	−	−	−	+	+
10	*N*-4-Methoxyphenyl carbamate	0.09	−	−	−	−	−	−	−	+	+
		0.09	−	−	−	−	−	−	−	+	+
		0.09	−	−	−	−	−	−	−	+	+
11	*N*-4-Nitrophenyl carbamate	0.05	−	−	−	−	−	−	−	*	+
		0.06	−	−	−	−	−	−	−	*	+
		0.08	−	−	−	−	−	−	−	+	+
12	*N*-Styryl carbamate	0.10	+	−	−	−	−	−	−	+	+
		0.21	+	−	−	−	−	−	−	+	+
		0.32	+	−	−	−	−	−	−	+	+

a 10 mg of each cellulosic material was suspended in 1 mL of the corresponding solvent. After the suspensions were stirred at 100 °C for 20 h, the dissolution was confirmed.

b A chart depicting the structures of the prepared cellulosic materials could be found in the ESI.

c Dissolutions in these solvents were performed at room temperature utilizing the same quantities.^72^ − not dissolved, + dissolved, * opalescent (no visible particles were observed by eye, example optical microscope images could be seen in ESI).

In the cellulose *N*-substituted carbamates series prepared through the TC/TU reaction some of the materials with the highest DS were soluble in dimethyl sulfoxide (DMSO) (Table 6 entries 1–4, 9 and 12). A clear trend in the DMSO dissolution series could be observed; with the increase of the number of atoms in the aliphatic chain (0–3 carbon atoms) the dissolution increases, but upon further grow of the aliphatic chain the solubility then decreases (4 carbon atoms or sterically hindered derivatives). On the other hand, the cellulose *N*-styryl carbamate was soluble in DMSO in all of the prepared DS ranges. However, solubility trials with *N*,*N*-dimethyl acetamide (DMA, a ‘classical cellulose solvent’) and with the more sustainable alternatives gamma-valerolactone (γ-VL) or Cyrene™, were negative.^71^ The slow dissolution of the samples at elevated temperatures as well as the absence of such in most of the common organic solvents, together with previously obtained data, indicated that there was none to a minimal change of the degree of polymerisation of the prepared cellulose *N*-substituted carbamates.^57^

Apart from the cationic cellulose derivative (Table 6 entry 6), none of the prepared cellulose *N*-substituted carbamates dissolved in water (H_2_O) or 1 M acetic acid (AcOH) in water. On the contrast, alkaline water was rather good solvent for all of the *N*-substituted carbamates at higher NaOH concentrations (see Table 6).

Comparison of the results from the dissolution of the cellulose *N*-phenyl/butyl carbamates prepared through the isocyanate or the TC/TU reaction (Tables S2† and 6) shows some differences between samples with similar DS. This behavioural difference most likely originates from the different reactivity of the used reagents. Both reactions are in principle homogenous, and thus the functional group distribution should be similar, but the isocyanates are more reactive, which could lead to different substitution patterns (consequently, being less selective). The cellulose *N*-phenyl carbamates with DS in the range of 0.14–0.16 (Table 6 entry 9) prepared through TC/TU reaction were soluble in DMSO, 1 M and 2 M NaOH solution, but the ones prepared from isocyanates were not soluble at all (Table S2† entry 1 DS 0.11–0.14). Another similar discrepancy can be seen in the cellulose *N*-butyl carbamate series. The materials prepared through TC/TU reaction (Table 6 entry 5) with DS between 0.13–0.18 almost dissolved in 1 M and 2 M NaOH solution, while these prepared through the isocyanate reaction (Table S2† entry 2 DS 0.14–0.17) were insoluble. With cellulose derivatives it is known that selective distribution of the introduced sidechains usually improves the solubility, as the introduced sidechains can inhibit the inter- and intramolecular hydrogen bonding. Therefore, it seems that the somewhat slower reaction rate in the TC/TU reaction favours more selective distribution of the side chains and thus yields better solubility in organic solvents. To validate this, we per-*O*-acetylated the cellulose *N*-phenyl carbamates obtained from the different reactions (TC/TU *vs.* isocyanate pathway) and compared their acetyl substitution patterns. This experiment allows to indirectly observe which hydroxyl groups were carbamoylated initially. For the TC/TU reaction the experiments showed predominant carbamoylation at the cellulose's hydroxyl attached to the carbon 6 (C6) over C2 and C3. For the isocyanate reaction, there was less selectivity towards C6 (Experimental details and quantitative ^13^C NMR in ESI†). These findings correspond well with the observed solubility differences in these materials.

Looking into the solubility results further, we can see that cellulose *N*-phenyl carbamates (Table S2† entry 1) with DS 0.51 and 0.65 are soluble in DMSO and the latter one is also soluble in DMA. On the contrary, only the cellulose *N*-phenyl carbamates with DS 0.02–0.08 are somewhat soluble in 2 M NaOH solution. Notably, the cellulose *N*-butyl carbamate (Table S2† entry 2) with DS 2.53 dissolves well in the organic solvents but shows no dissolution in the used aqueous solvents.

## Experimental

Description of the general and the detailed synthetic procedures can be found in the ESI† associated with this article. Additionally, the used materials, methods and instrumentation are depicted in the ESI.†

## Conclusions

In summary, we have developed a sustainable protocol for the preparation of cellulose carbamates utilizing the superbase ionic liquid [mTBNH][OAc] as the reaction media and stable methyl *N*-substituted carbamates as the reagents. The reaction allowed for mild preparation of multiple cellulose carbamates with variable and controllable degree of substitution from readily available starting materials. The solubility of the obtained cellulose carbamates was tested in various organic and aqueous solvents, where some intrinsic trends were observed. The cellulose materials prepared from the TC/TU reaction possessed better solubility than those prepared using the traditional isocyanate reaction. Ultimately, this unprecedented TC/TU transformation of cellulose with methyl *N*-substituted carbamates presents an attractive and sustainable alternative in the preparation of cellulose carbamates with variable degrees of substitution.

## Data availability

The data supporting this article have been included as part of the ESI.†

## Author contributions

A. R. T. planned the work, executed the optimization studies, synthesized part of the needed starting materials and some of the cellulose carbamates, characterized the obtained materials, and wrote the manuscript. M. D. executed the cellulose reactions with isocyanates, contributed further to the characterization, and edited the manuscript. E. G. provide the superbase ionic liquids used in the studies, contributed for the optical microscopy, and edited the manuscript. M. P. synthesized some of the cellulose carbamates and contributed for the dissolution tests. I. K. conceived the project, and provided overall project guidance, and edited the manuscript together with A. R. T., M. D. and E. G.

## Conflicts of interest

There are no conflicts of interest to declare.

## Supplementary Material

RA-014-D4RA04521A-s001
