# Supplementary material for: Cellulose carbamates via transcarbamoylation/transurethanization of methyl carbamates in superbase–acid conjugate ionic liquids
Source: RSC Adv. 2024 Jul 22;14(32):23118–28. doi: 10.1039/d4ra04521a (PMC11262085; doi:10.1039/d4ra04521a)

Electronic Supplementary Material (ESI) for RSC Advances.
This journal is © The Royal Society of Chemistry 2024

Electronic Supplementary Information

Cellulose carbamates *via* transcarbamoylation/transurethanization of methyl carbamates in superbase-acid conjugate ionic liquids

Aleksandar R. Todorov,*[,a] Magdalena Dryś[a,b], Eva Gazagnaire[a], Manisha Podder[a] and Ilkka Kilpeläinen[a]

[a] Materials Chemistry Division, Department of Chemistry, University of Helsinki, 00560 Helsinki, Finland

[b] Natural Resources Institute Finland, Latokartanonkaari 9, 00790, Helsinki, Finland

Corresponding author: aleksandar.todorov@helsinki.fi

Contents

General information ... 4
Determination of the degree of substitution – isolated samples ... 4
By solution state ^{1}H NMR ... 4
By nitrogen content from elemental analysis ... 5
Determination of the degree of substitution – reaction optimization samples ... 5
Optimization studies ... 6
Reagent ... 7
Reagent amount ... 7
Reaction time ... 8
Reaction temperature and Ionic liquid ... 8
Ionic liquid composition ... 10
Time elapsed NMR experiments ... 11
Synthesis of cellulose *N*-phenyl carbamate ... 11
Decomposition of cellulose *N*-phenyl carbamate ... 11
Comparison of reaction mixtures and isolated materials for cellulose *N*-phenyl carbamate ... 12
Miscellaneous experiments ... 14
Synthesis of per-O-acetylated cellulose *N*-phenyl carbamate ... 14
Dissolution tests for the prepared cellulose materials through isocyanate pathway ... 16
Optical microscopy images ... 16
Cellulose materials – schematic representation ... 18
Synthesis of cellulose materials ... 19
General procedure - transcarbamoylation / transurethanization ... 19
Cellulose carbamate ... 19
Cellulose *N*-methyl carbamate ... 19
Cellulose *N*-ethyl carbamate ... 20
Cellulose *N*-propyl carbamate ... 20
Cellulose *N*-butyl carbamate ... 20
Cellulose *N*-(N,N,N-)trimethylpropan-1-aminium acetate carbamate ... 20
Cellulose *N*-*iso*-propyl carbamate ... 21
Cellulose *N*-*tert*-butyl carbamate ... 21
Cellulose *N*-phenyl carbamate ... 21
Cellulose *N*-4-methoxyphenyl carbamate ... 21
Cellulose *N*-4-nitrophenyl carbamate ... 22
Cellulose *N*-styryl carbamate ... 22
General procedure – isocyanate pathway ... 22

Cellulose *N*-phenyl carbamate 23
Cellulose *N*-butyl carbamate 24
Synthesis of methyl *N*-substituted carbamates 24
General procedure 24
Methyl-*N*-ethyl carbamate 25
Methyl-*N*-propyl carbamate 25
Methyl-*N*-butyl carbamate 25
Methyl-*N*-*iso*-propyl carbamate 25
Methyl-*N*-*tert*-butyl carbamate 26
Methyl-*N*-4-methoxyphenyl carbamate 26
Methyl-*N*-4-nitrophenyl carbamate 26
Methyl-*N*-styryl carbamate 27
***Iso*-propyl-*N*-phenyl carbamate** 27
References 28
Copy of spectral data 29

General information

All experimental procedures, the spectroscopic information for the prepared substrates and materials, and the utilized equipment are collected and presented below. All commercial chemicals were used as received. Microcrystalline cellulose (MCC) Avicel® PH-101 was purchased from Sigma Aldrich. Methyl *N*-methyl carbamate was purchased from FluoroChem, methyl *N*-phenyl carbamate from TCI Europe, and methyl carbamate from Sigma Aldrich. The used phenyl and butyl isocyanates were purchased from Sigma Aldrich. The rest of the utilised chemicals were obtained from Sigma Aldrich, TCI Europe, or BLDpharm. The used ionic liquids were prepared in our research group. Liquid state ^{1}H, diffusion-edited ^{1}H, and ^{13}C{^{1}H} NMR spectra were recorded at 25 or 65 °C using Bruker Avance Neo 400 [400.15 MHz], Bruker Avance Neo 500 [499.83 MHz], and Bruker Avance Neo 600 [599.69 MHz] spectrometers equipped with 5 mm triple resonance (^{1}H/^{19}F, ^{13}C, ^{31}P) inverse-detection probe with triple axis gradients; temperature range -20 - 80 °C. ^{1}H and ^{13}C{^{1}H} spectra were referenced to the residual solvent signals of DMSO-d_6 2.50 and 39.5 ppm, respectively, with no special notation for the equivalent carbons. Elemental analysis was performed with HANAU ElementarAnalysensysteme GmbH, Germany, vario MICRO cube with serial number 15082023, equipped with TCD-detector.

Determination of the degree of substitution – isolated samples

By solution state ^{1}H NMR

The degree of substitution (DS) of the prepared and isolated cellulose carbamate materials was determined from the liquid state quantitative ^{1}H NMR spectra at 65 °C in DMSO-d_6/LiCl. Samples were prepared utilising the following procedure: 0.0175 g of the analysed carbamate material was measured into an 8 mL vial. The vial was equipped with a cylindrical stirrer bar (10 x 6 mm) and was charged with 1.25 mL DMSO-d_6. Then the suspension was stirred for 2 hours at 130 °C, after which the temperature was decreased to 90 °C. After the addition of 0.0875 g of dry LiCl at 90 °C the heating was ceased, and the mixture was stirred for 18 furthermore, during which the sample fully dissolved. After the full dissolution of the cellulose carbamate sample, 1 mL of the mixture was transferred into NMR tube and was analysed by NMR.

The crude NMR data was analysed following scheme S1. Firstly, the crude NMR data was processed, and phase corrected in MestReNova. Secondly, the phase corrected data was exported as .txt format for further analysis in Fityk.[1,2]

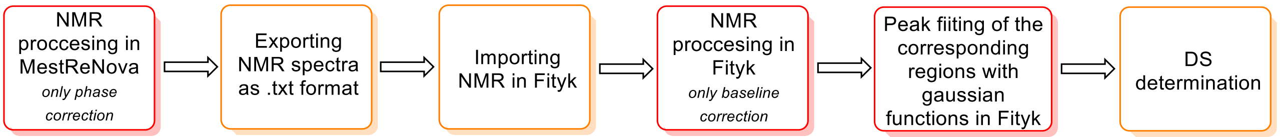

Scheme S1. Analysis of the NMR data

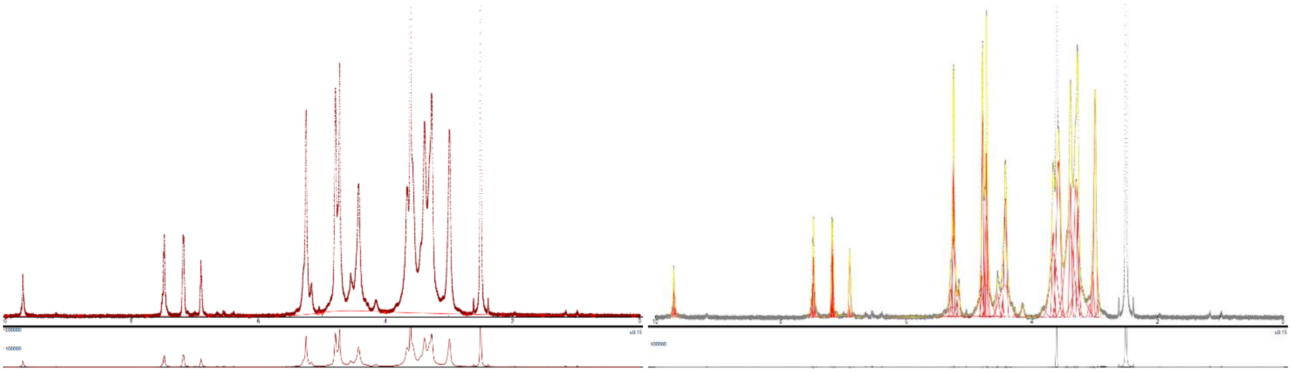

Figure S1 Example of spline baseline correction (left) and Gaussian peak fitting (right) in Fityk for cellulose N-phenyl carbamate with DS 0.14

After the NMR data is imported into Fityk, it is further processed (spline baseline corrected, figure S1 left). Then the corresponding cellulose and substituent regions were peak fitted with Gaussian functions (figure S1 right).

The DS of the samples were determined following equation 1:

$$DS = \frac{\frac{I_{(substituent)}}{x}}{\frac{I_{(cellulose)}}{10}} \quad \text{Eq. 1}$$

where $I_{(substituent)}$ is the peak area of the combined substituent signal, $I_{(cellulose)}$ is the peak area of the combined cellulose backbone signals, ***X*** (dependable from the substituent, e.g., 6 for *N*-phenyl carbamate) and ***10*** (7 from cellulose backbone signals and 3 from cellulose OH signals) are the total number of protons for the above-mentioned fragments. N.B! In DMSO-d_6/LiCl the cellulose OH signals were present in the ^{1}H NMR, so they were considered during the calculation of the DS.

By nitrogen content from elemental analysis

Cellulose carbamate samples were measured in 4x4x11mm tin boats on a Mettler Toledo MX5 balance. Then, the samples were subjected to elemental analysis determination. Devise's 1st combustion tube was filled with quartz wool, tungsten(VI) oxide-granulate, corundum balls, and an ash crucible with a bottom of Al_2O_3 –wool. The 2nd reduction tube was filled with quartz wool, copper, corundum balls, and silver wool. The calibration of the device was performed with sulphanilamide (Elementar, Germany). The measurements were triplicated, and the DS was determined as an average from them.

The DS of the samples were determined following equation 2:

$$DS = \frac{Mw(AGU) * N(\%)}{(Mw(N) * 100) - (Mw(Carb) * N(\%))} \quad \text{Eq. 2}$$

where ***Mw (AGU)*** is the molecular mass of cellulose 162.14 g/mol, ***N*** is the determined nitrogen content from the elemental analysis in percentage, ***Mw (N)*** is the molecular mass of nitrogen 14.01 g/mol, ***Mw (Carb)*** is the molecular mass of the carbamate group (dependable from each substituent, e.g., 120.13 g/mol for *N*-phenyl carbamate).[3,4]

Determination of the degree of substitution – reaction optimization samples

A standard calibration curve method was used to determine the DS of the reaction optimization samples.[1] The standard calibration curve was prepared from duplicated series of cellulose *N*-phenyl and *N*-butyl carbamates with known DS (determined from ^{1}H NMR in DMSO-d_6/LiCl at 65 °C) plotted against the corresponding integral ratios obtained from the diffusion-edited ^{1}H NMR in [mTBNH][OAc] at 65 °C. The standard calibration curve samples were measured in as similar as possible conditions (e.g., concentration, solvents, dilutions, NMR tubes, NMR instrumentation etc.,) as the analysed samples, to avoid substantial errors. The integral regions were set as follows: for cellulose between 4.00 and 5.50 ppm, for the phenyl group (in cellulose *N*-phenyl carbamate) between 6.50 and 8.00 ppm, and for the butyl group (in cellulose *N*-butyl carbamate) between 0.50 and 1.50 ppm. The used tabulated data as well as the constructed calibration curves for cellulose *N*-phenyl and *N*-butyl carbamates are shown in table S1 and in figure S2.

Table S1 Cellulose N-phenyl and N-butyl carbamate samples with known DS for preparation of the standart calibration curves

Cellulose *N*-phenyl carbamate	Average integral ratio (Carbamate/Cellulose)	DS
1	0.484884166	0.08
2	1.184108213	0.14
3	2.224139237	0.29
4	3.604595679	0.51
5	3.720140011	0.65

Cellulose *N*-butyl carbamate	Average integral ratio (Carbamate/Cellulose)	DS
1	0.241231534	0.04
2	1.026278740	0.11
3	1.750046298	0.14
4	1.730085306	0.14
5	2.013209539	0.17
6	2.609225036	0.23
7	9.730256562	2.53

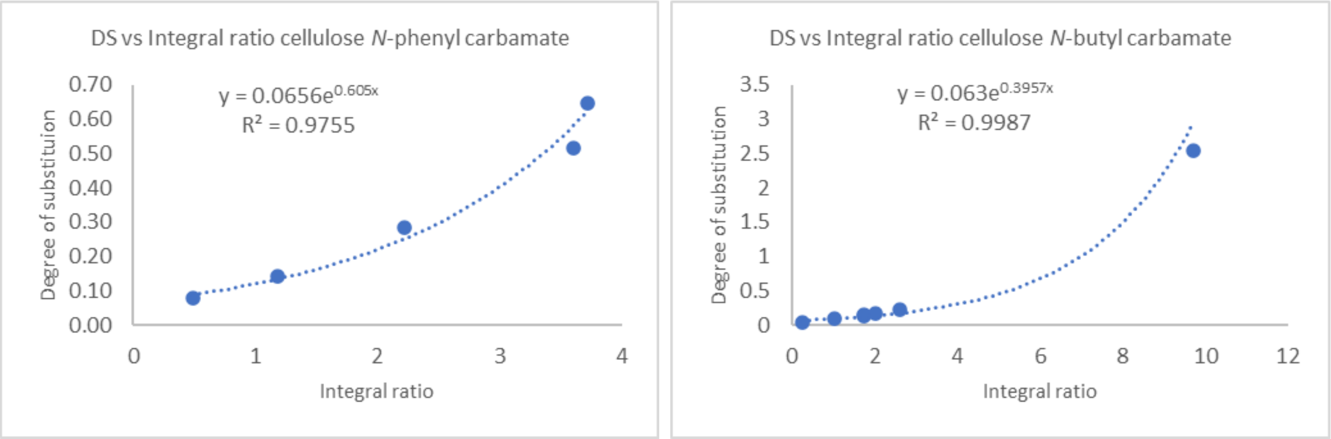

Figure S2 Standard calibration curve with exponential fit for cellulose N-phenyl (left) and N-butyl (right) carbamates

The standard calibration curves were exponentially fitted, and equations 3 and 4 were obtained. They were used further to determine the DS from the crude reaction optimization samples in both the aromatic and aliphatic series.

$$DS(phenyl) = 0.0656e^{0.605x} \quad \text{Eq.3}$$

$$DS(butyl) = 0.063e^{0.3957x} \quad \text{Eq.4}$$

In equation 3, ***X*** is the integral ratio between the *N*-phenyl carbamate group signals (6.50 - 8.00 ppm) and the cellulose group signal (4.00 - 5.50 ppm) from the diffusion-edited ^{1}H spectra. In equation 4, the ***X*** is the integral ratio of the *N*-butyl carbamate signals (0.50 - 1.50 ppm) and the cellulose group signal (4.00 - 5.50 ppm) from the diffusion-edited ^{1}H spectra. The reported DS values in tables 1, 2, and 3 are the average of two independent reactions performed in the same conditions. The shown method for determination of the DS works well for cellulose *N*-phenyl and *N*-butyl carbamate samples in the calibrated region. The lower detection limit of the described standard calibration curve method was found to be DS of 0.066 (for phenyl) and 0.063 (for butyl) or an integral ratio of 0.01 (for both).

Optimization studies

Microcrystalline cellulose (0.0500 g, 0.3084 mmol) was placed into an 8 mL vial. Then, a cylindrical stirrer bar (10 x 6 mm) was added, followed by 1 mL [mTBNH][OAc] (or [mTBDH][OAc] when applicable), and the vial was sealed. The viscous reaction mixture was suspended with vortex genie 2 (scientific industries), and then it was immersed into a preheated to the desired reaction temperature (typically 80 °C or 120 °C) silicon bath. The suspended MCC was stirred at this temperature until complete dissolution occurs (typically 30 minutes). After that, the vial was taken out from the silicon bath, and the desired amount of

the used reagent was added to the dissolved MCC. For reactions with methyl *N*-phenyl carbamate after the reagent, 0.5 mL of gamma-valerolactone (γ-VL) was added to reduce the viscosity of the reaction mixture. Then the vial was resealed, and the reaction mixture was immersed again into the silicon bath and was stirred for the corresponding time (typically 20 h). After that, the reaction mixture was diluted with 1 mL of DMSO-d_6 and was analysed by diffusion-edited ^{1}H NMR. Then the DS was determined from the crude reaction mixtures using the standard calibration curves, and the results can be found in tables 1, 2, and 3.

Reagent

The titled parameter was varied following the optimization studies procedure. Results are the average of two independent reactions performed in the same conditions. This parameter optimization was performed only for the cellulose *N*-phenyl carbamate series (table 1 entries 1-4)

Methyl *N*-phenyl carbamate: Starting from MCC (0.0500 g, 0.3084 mmol) and MeOCONHPh (0.4196 g, 2.7756 mmol). Reaction time was 20 h and the temperature was set at 80 °C. **Obtained DS: 0.18**

Ethyl *N*-phenyl carbamate: Starting from MCC (0.0500 g, 0.3084 mmol) and EtOCONHPh (0.4585 g, 2.7756 mmol). Reaction time was 20 h and the temperature was set at 80 °C. **Obtained DS: 0.11**

***i*-Propyl *N*-phenyl carbamate:** Starting from MCC (0.0500 g, 0.3084 mmol) and *i*-PrOCONHPh (0.4974 g, 2.7756 mmol). Reaction time was 20 h and the temperature was set at 80 °C. **Obtained DS: 0.10**

***t*-Butyl *N*-phenyl carbamate:** Starting from MCC (0.0500 g, 0.3084 mmol) and *t*-BuOCONHPh (0.5364 g, 2.7756 mmol). Reaction time was 20 h and the temperature was set at 80 °C. **Obtained DS: 0.11**

Reagent amount

The titled parameter was varied following the optimization studies procedure. Results are the average of two independent reactions performed in the same conditions. This parameter optimization was performed for both cellulose *N*-phenyl and *N*-butyl carbamate series (table 1 entries 5-9 and table 2 entries 1-5).

Methyl *N*-phenyl carbamate – 3 equivalents to AGU: Starting from MCC (0.0500 g, 0.3084 mmol) and MeOCONHPh (0.1399 g, 0.9252 mmol). Reaction time was 20 h and the temperature was set at 80 °C. **Obtained DS: 0.10**

Methyl *N*-phenyl carbamate – 6 equivalents to AGU: Starting from MCC (0.0500 g, 0.3084 mmol) and MeOCONHPh (0.2797 g, 1.8504 mmol). Reaction time was 20 h and the temperature was set at 80 °C. **Obtained DS: 0.14**

Methyl *N*-phenyl carbamate – 9 equivalents to AGU: Starting from MCC (0.0500 g, 0.3084 mmol) and MeOCONHPh (0.4196 g, 2.7756 mmol). Reaction time was 20 h and the temperature was set at 80 °C. **Obtained DS: 0.18**

Methyl *N*-phenyl carbamate – 12 equivalents to AGU: Starting from MCC (0.0500 g, 0.3084 mmol) and MeOCONHPh (0.5594 g, 3.7008 mmol). Reaction time was 20 h and the temperature was set at 80 °C. **Obtained DS: 0.20**

Methyl *N*-phenyl carbamate – 15 equivalents to AGU: Starting from MCC (0.0500 g, 0.3084 mmol) and MeOCONHPh (0.6993 g, 4.6260 mmol). Reaction time was 20 h and the temperature was set at 80 °C. **Obtained DS: 0.26**

Methyl *N*-butyl carbamate – 3 equivalents to AGU: Starting from MCC (0.0500 g, 0.3084 mmol) and MeOCONHBu (0.1214 g, 0.9252 mmol). Reaction time was 20 h and the temperature was set at 120 °C. **Obtained DS: 0.10**

Methyl *N*-butyl carbamate – 6 equivalents to AGU: Starting from MCC (0.0500 g, 0.3084 mmol) and MeOCONHBu (0.2427 g, 1.8504 mmol). Reaction time was 20 h and the temperature was set at 120 °C. **Obtained DS: 0.16**

Methyl *N*-butyl carbamate – 9 equivalents to AGU: Starting from MCC (0.0500 g, 0.3084 mmol) and MeOCONHBu (0.3641 g, 2.7756 mmol). Reaction time was 20 h and the temperature was set at 120 °C. **Obtained DS: 0.23**

Methyl *N*-butyl carbamate – 12 equivalents to AGU: Starting from MCC (0.0500 g, 0.3084 mmol) and MeOCONHBu (0.4855 g, 3.7008 mmol). Reaction time was 20 h and the temperature was set at 120 °C. **Obtained DS: 0.32**

Methyl *N*-butyl carbamate – 15 equivalents to AGU: Starting from MCC (0.0500 g, 0.3084 mmol) and MeOCONHBu (0.6068 g, 4.6260 mmol). Reaction time was 20 h and the temperature was set at 120 °C. **Obtained DS: 0.36**

Reaction time

The titled parameter was varied following the optimization studies procedure. Results are the average of two independent reactions performed in the same conditions. This parameter optimization was performed for both cellulose *N*-phenyl and *N*-butyl carbamate series (table 1 entries 10-12 and table 2 entries 6-8).

Methyl *N*-phenyl carbamate – 24 h: Starting from MCC (0.0500 g, 0.3084 mmol) and MeOCONHPh (0.4196 g, 2.7756 mmol). Reaction time was 24 h and the temperature was set at 80 °C. **Obtained DS: 0.20**

Methyl *N*-phenyl carbamate – 48 h: Starting from MCC (0.0500 g, 0.3084 mmol) and MeOCONHPh (0.4196 g, 2.7756 mmol). Reaction time was 48 h and the temperature was set at 80 °C. **Obtained DS: 0.19**

Methyl *N*-phenyl carbamate – 72 h: Starting from MCC (0.0500 g, 0.3084 mmol) and MeOCONHPh (0.4196 g, 2.7756 mmol). Reaction time was 72 h and the temperature was set at 80 °C. **Obtained DS: 0.21**

Methyl *N*-butyl carbamate – 24 h: Starting from MCC (0.0500 g, 0.3084 mmol) and MeOCONHBu (0.2427 g, 1.8504 mmol). Reaction time was 24 h and the temperature was set at 120 °C. **Obtained DS: 0.16**

Methyl *N*-butyl carbamate – 48 h: Starting from MCC (0.0500 g, 0.3084 mmol) and MeOCONHBu (0.2427 g, 1.8504 mmol). Reaction time was 48 h and the temperature was set at 120 °C. **Obtained DS: 0.09**

Methyl *N*-butyl carbamate – 72 h: Starting from MCC (0.0500 g, 0.3084 mmol) and MeOCONHBu (0.2427 g, 1.8504 mmol). Reaction time was 72 h and the temperature was set at 120 °C. **Obtained DS: 0.08**

Reaction temperature and Ionic liquid

The titled parameters were varied following the optimization studies procedure. The reaction performed in [mTBDH][OAc] required the addition of gamma-valerolactone before the dissolution. Results are the average of two independent reactions performed in the same conditions. These parameter optimizations were performed for both cellulose *N*-phenyl and *N*-butyl carbamate series (table 1 entries 13-20 and table 2 entries 9-20).

Methyl *N*-phenyl carbamate – [mTBNH][OAc] + *γ*-VL and 60 °C: Starting from MCC (0.0500 g, 0.3084 mmol) and MeOCONHPh (0.4196 g, 2.7756 mmol). Reaction time was 20 h and the temperature was set at 60 °C. **Obtained DS: 0.07**

Methyl *N*-phenyl carbamate – [mTBDH][OAc] + *γ*-VL and 60 °C: Starting from MCC (0.0500 g, 0.3084 mmol) and MeOCONHPh (0.4196 g, 2.7756 mmol). Reaction time was 20 h and the temperature was set at 60 °C. **Obtained DS: 0.07**

Methyl *N*-phenyl carbamate – [mTBNH][OAc] + *γ*-VL and 80 °C: Starting from MCC (0.0500 g, 0.3084 mmol) and MeOCONHPh (0.4196 g, 2.7756 mmol). Reaction time was 20 h and the temperature was set at 80 °C. **Obtained DS: 0.18**

Methyl *N*-phenyl carbamate – [mTBDH][OAc] + *γ*-VL and 80 °C: Starting from MCC (0.0500 g, 0.3084 mmol) and MeOCONHPh (0.4196 g, 2.7756 mmol). Reaction time was 20 h and the temperature was set at 80 °C. **Obtained DS: 0.14**

Methyl *N*-phenyl carbamate – [mTBNH][OAc] + *γ*-VL and 100 °C: Starting from MCC (0.0500 g, 0.3084 mmol) and MeOCONHPh (0.4196 g, 2.7756 mmol). Reaction time was 20 h and the temperature was set at 100 °C. **Obtained DS: 0.14**

Methyl *N*-phenyl carbamate – [mTBDH][OAc] + *γ*-VL and 100 °C: Starting from MCC (0.0500 g, 0.3084 mmol) and MeOCONHPh (0.4196 g, 2.7756 mmol). Reaction time was 20 h and the temperature was set at 100 °C. **Obtained DS: 0.14**

Methyl *N*-phenyl carbamate – [mTBNH][OAc] + *γ*-VL and 120 °C: Starting from MCC (0.0500 g, 0.3084 mmol) and MeOCONHPh (0.4196 g, 2.7756 mmol). Reaction time was 20 h and the temperature was set at 100 °C. **Obtained DS: 0.07**

Methyl *N*-phenyl carbamate – [mTBDH][OAc] + *γ*-VL and 120 °C: Starting from MCC (0.0500 g, 0.3084 mmol) and MeOCONHPh (0.4196 g, 2.7756 mmol). Reaction time was 20 h and the temperature was set at 100 °C. **Obtained DS: 0.07**

Methyl *N*-butyl carbamate – [mTBNH][OAc] and 60 °C: Starting from MCC (0.0500 g, 0.3084 mmol) and MeOCONHBu (0.2427 g, 1.8504 mmol). Reaction time was 20 h and the temperature was set at 60 °C. **Obtained DS: 0.06**

Methyl *N*-butyl carbamate – [mTBNH][OAc] + *γ*-VL and 60 °C: Starting from MCC (0.0500 g, 0.3084 mmol) and MeOCONHBu (0.2427 g, 1.8504 mmol). Reaction time was 20 h and the temperature was set at 60 °C. **Obtained DS: 0.06**

Methyl *N*-butyl carbamate – [mTBDH][OAc] + *γ*-VL and 60 °C: Starting from MCC (0.0500 g, 0.3084 mmol) and MeOCONHBu (0.2427 g, 1.8504 mmol). Reaction time was 20 h and the temperature was set at 60 °C. **Obtained DS: 0.06**

Methyl *N*-butyl carbamate – [mTBNH][OAc] and 80 °C: Starting from MCC (0.0500 g, 0.3084 mmol) and MeOCONHBu (0.2427 g, 1.8504 mmol). Reaction time was 20 h and the temperature was set at 80 °C. **Obtained DS: 0.09**

Methyl *N*-butyl carbamate – [mTBNH][OAc] + *γ*-VL and 80 °C: Starting from MCC (0.0500 g, 0.3084 mmol) and MeOCONHBu (0.2427 g, 1.8504 mmol). Reaction time was 20 h and the temperature was set at 80 °C. **Obtained DS: 0.08**

Methyl *N*-butyl carbamate – [mTBDH][OAc] + *γ*-VL and 80 °C: Starting from MCC (0.0500 g, 0.3084 mmol) and MeOCONHBu (0.2427 g, 1.8504 mmol). Reaction time was 20 h and the temperature was set at 80 °C. **Obtained DS: 0.08**

Methyl *N*-butyl carbamate – [mTBNH][OAc] and 100 °C: Starting from MCC (0.0500 g, 0.3084 mmol) and MeOCONHBu (0.2427 g, 1.8504 mmol). Reaction time was 20 h and the temperature was set at 100 °C. **Obtained DS: 0.17**

Methyl *N*-butyl carbamate – [mTBNH][OAc] + *γ*-VL and 100 °C: Starting from MCC (0.0500 g, 0.3084 mmol) and MeOCONHBu (0.2427 g, 1.8504 mmol). Reaction time was 20 h and the temperature was set at 100 °C. **Obtained DS: 0.13**

Methyl *N*-butyl carbamate – [mTBDH][OAc] + *γ*-VL and 100 °C: Starting from MCC (0.0500 g, 0.3084 mmol) and MeOCONHBu (0.2427 g, 1.8504 mmol). Reaction time was 20 h and the temperature was set at 100 °C. **Obtained DS: 0.13**

Methyl *N*-butyl carbamate – [mTBNH][OAc] and 120 °C: Starting from MCC (0.0500 g, 0.3084 mmol) and MeOCONHBu (0.2427 g, 1.8504 mmol). Reaction time was 20 h and the temperature was set at 120 °C. **Obtained DS: 0.15**

Methyl *N*-butyl carbamate – [mTBNH][OAc] + *γ*-VL and 120 °C: Starting from MCC (0.0500 g, 0.3084 mmol) and MeOCONHBu (0.2427 g, 1.8504 mmol). Reaction time was 20 h and the temperature was set at 120 °C. **Obtained DS: 0.14**

Methyl *N*-butyl carbamate – [mTBDH][OAc] + *γ*-VL and 120 °C: Starting from MCC (0.0500 g, 0.3084 mmol) and MeOCONHBu (0.2427 g, 1.8504 mmol). Reaction time was 20 h and the temperature was set at 120 °C. **Obtained DS: 0.10**

Ionic liquid composition

The titled parameter was varied following the optimization studies procedure. Results are the average of two independent reactions performed in the same conditions. This parameter optimization was performed for both cellulose *N*-phenyl and *N*-butyl carbamate series (table 2 entries 1-12).

Methyl *N*-phenyl carbamate – [mTBNH][OAc] [1:1.5]: Starting from MCC (0.0500 g, 0.3084 mmol) and MeOCONHPh (0.4196 g, 2.7756 mmol). Reaction time was 20 h and the temperature was set at 80 °C. **Obtained DS: 0.15**

Methyl *N*-phenyl carbamate – [mTBNH][OAc] [1:1]: Starting from MCC (0.0500 g, 0.3084 mmol) and MeOCONHPh (0.4196 g, 2.7756 mmol). Reaction time was 20 h and the temperature was set at 80 °C. **Obtained DS: 0.21**

Methyl *N*-phenyl carbamate – [mTBNH][OAc] [1.5:1]: Starting from MCC (0.0500 g, 0.3084 mmol) and MeOCONHPh (0.4196 g, 2.7756 mmol). Reaction time was 20 h and the temperature was set at 80 °C. **Obtained DS: 0.19**

Methyl *N*-phenyl carbamate – [mTBNH][OAc] [2:1]: Starting from MCC (0.0500 g, 0.3084 mmol) and MeOCONHPh (0.4196 g, 2.7756 mmol). Reaction time was 20 h and the temperature was set at 80 °C. **Obtained DS: 0.19**

Methyl *N*-phenyl carbamate – [mTBNH][OAc] [5:1]: Starting from MCC (0.0500 g, 0.3084 mmol) and MeOCONHPh (0.4196 g, 2.7756 mmol). Reaction time was 20 h and the temperature was set at 80 °C. **Obtained DS: 0.20**

Methyl *N*-phenyl carbamate – [mTBNH][OAc] [10:1]: Starting from MCC (0.0500 g, 0.3084 mmol) and MeOCONHPh (0.4196 g, 2.7756 mmol). Reaction time was 20 h and the temperature was set at 80 °C. **Obtained DS: 0.21**

Methyl *N*-butyl carbamate – [mTBNH][OAc] [1:1.5]: Starting from MCC (0.0500 g, 0.3084 mmol) and MeOCONHBu (0.2427 g, 1.8504 mmol). Reaction time was 20 h and the temperature was set at 120 °C. **Obtained DS: 0.11**

Methyl *N*-butyl carbamate – [mTBNH][OAc] [1:1]: Starting from MCC (0.0500 g, 0.3084 mmol) and MeOCONHBu (0.2427 g, 1.8504 mmol). Reaction time was 20 h and the temperature was set at 120 °C. **Obtained DS: 0.12**

Methyl *N*-butyl carbamate – [mTBNH][OAc] [1.5:1]: Starting from MCC (0.0500 g, 0.3084 mmol) and MeOCONHBu (0.2427 g, 1.8504 mmol). Reaction time was 20 h and the temperature was set at 120 °C. **Obtained DS: 0.10**

Methyl *N*-butyl carbamate – [mTBNH][OAc] [2:1]: Starting from MCC (0.0500 g, 0.3084 mmol) and MeOCONHBu (0.2427 g, 1.8504 mmol). Reaction time was 20 h and the temperature was set at 120 °C. **Obtained DS: 0.09**

Methyl *N*-butyl carbamate – [mTBNH][OAc] [5:1]: Starting from MCC (0.0500 g, 0.3084 mmol) and MeOCONHBu (0.2427 g, 1.8504 mmol). Reaction time was 20 h and the temperature was set at 120 °C. **Obtained DS: 0.08**

Methyl *N*-butyl carbamate – [mTBNH][OAc] [10:1]: Starting from MCC (0.0500 g, 0.3084 mmol) and MeOCONHBu (0.2427 g, 1.8504 mmol). Reaction time was 20 h and the temperature was set at 120 °C. **Obtained DS: 0.08**

Time elapsed NMR experiments

Synthesis of cellulose *N*-phenyl carbamate

Microcrystalline cellulose (0.0500 g, 0.3084 mmol) was placed into an 8 mL vial. Then a cylindrical stirrer bar (10 x 6 mm) was added, followed by 1 mL [mTBNH][OAc], and the vial was sealed. The viscous reaction mixture was suspended with vortex genie 2 (scientific industries) and then it was immersed into a preheated to 80 °C silicon bath. After 30 minutes of stirring, the MCC was completely dissolved, and the vial was removed from the silicon bath. After cooling the reaction mixture, MeOCONHPh (0.4196 g, 2.7756 mmol) was added, followed by 1 mL of DMSO-d_6. After that, 1 mL from the well-stirred reaction mixture was transferred to an NMR tube. Then, the NMR tube was placed into the preheated to 80 °C NMR spectrometer and was subjected to NMR analysis. The diffusion-edited ^{1}H data was collected every 30 minutes for 24 hours resulting in 48 individual points. The DS was determined using the standard calibration curve. N.B! Due to operational procedures, the first obtained data point is 30 minutes after the insertion of the sample. **Observed DS: 0.07 – 0.11**

Decomposition of cellulose *N*-phenyl carbamate

Cellulose *N*-phenyl carbamate (0.0500 g, 0.2620 mmol) was placed into an 8 mL vial. Then a cylindrical stirrer bar (10 x 6 mm) was added followed, by 1 mL [mTBNH][OAc], and the vial was sealed. The viscous reaction mixture was suspended with vortex genie 2 (scientific industries) and then it was immersed into a preheated to 80 °C silicon bath. After 18 hours of stirring, the cellulose *N*-phenyl carbamate was completely dissolved, and the vial was removed from the silicon bath. To the solution was added 1 mL DMSO-d_6 and the reaction mixture was split into two equal parts (1 mL each).

The first part (containing only cellulose *N*-phenyl carbamate - 0.0250 g, 0.1310 mmol) was subjected to NMR analysis utilising the diffusion-edited ^{1}H and the standard calibration curve. **Observed DS: 0.27**

To the second portion (containing cellulose *N*-phenyl carbamate - 0.0250 g, 0.1310 mmol) was added EtOH (0.0382 mL, 0.6550 mmol). After that, the well-stirred reaction mixture was transferred to an NMR tube. Then, the NMR tube was placed into the preheated to 80 °C NMR spectrometer and was subjected to NMR analysis. The diffusion-edited ^{1}H data was collected every 30 minutes for 24 hours resulting in 48 individual points. The DS was determined using the standard calibration curve. N.B! Due to operational procedures, the first obtained data point is 30 minutes after the insertion of the sample. **Observed DS: 0.22 – 0.15**

Comparison of reaction mixtures and isolated materials for cellulose *N*-phenyl carbamate

The following diffusion-edited stacked ^{1}H NMR spectra were used to visually compare the existing differences before and after the isolation of the corresponding cellulose *N*-phenyl carbamates obtained through the isocyanate pathway (figure S3). The reaction conditions and the calculated DS can be found in table 5 entries 3, 8, 11-13.

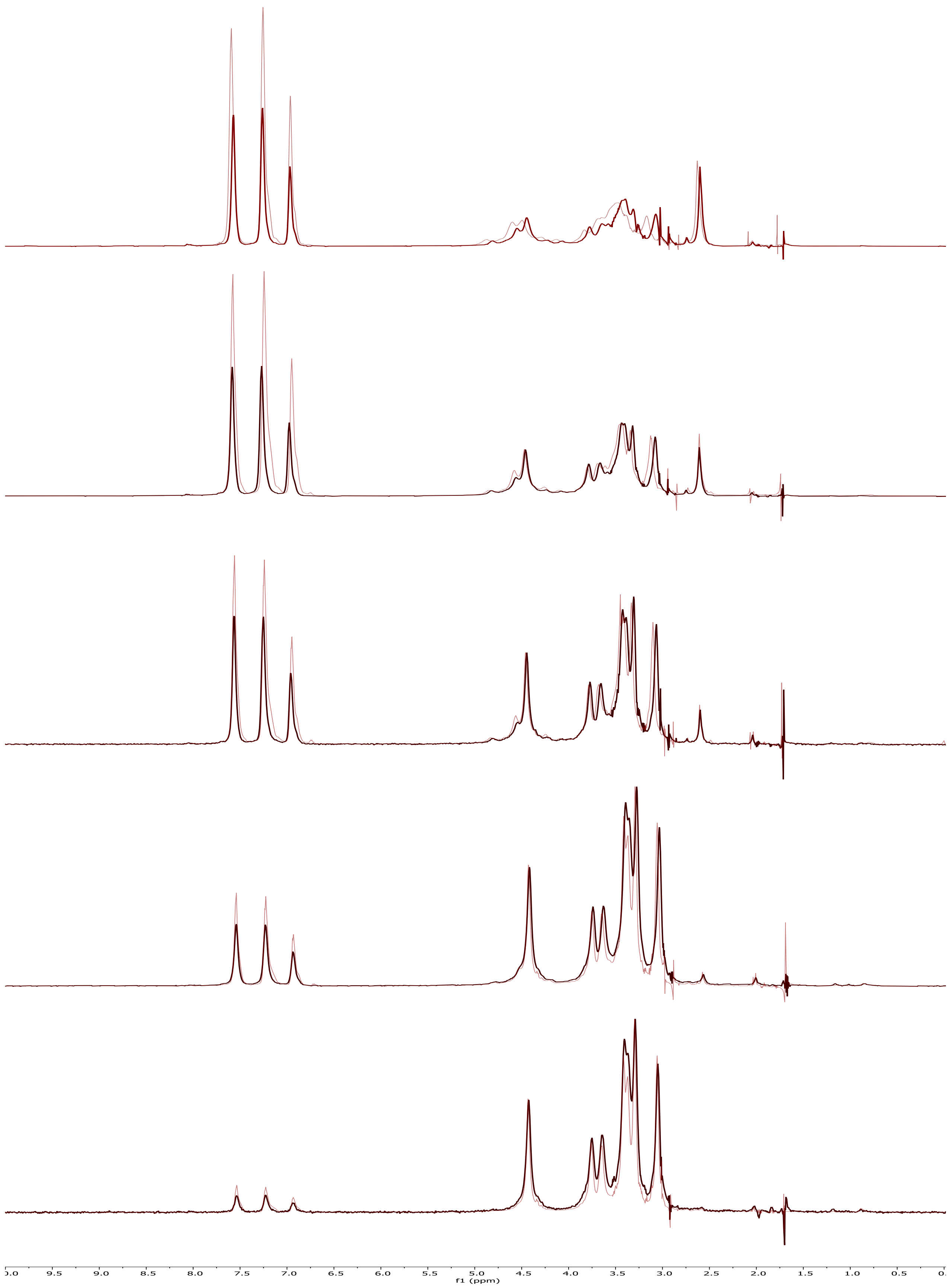

Figure S3 Comparison of diffusion-edited ¹H NMR spectra of five different reactions before isolation (pale) and after isolation of the cellulose N-phenyl carbamate (solid). DS of isolated sample (solid) from bottom to top: 0.08; 0.14; 0.29; 0.51; 0.65.

Miscellaneous experiments

Synthesis of per-O-acetylated cellulose *N*-phenyl carbamate

The following synthetic procedure was adapted from Tezuka and Tsuchiya.[5]

Sample A: Starting from cellulose *N*-phenyl carbamate from **TC/TU reaction** with DS 0.16.

Sample B: Starting from cellulose *N*-phenyl carbamate from **isocyanate reaction** with DS 0.12.

Sample A or B cellulose *N*-phenyl carbamate (0.0500 g, 0.3084 mmol) was placed into an 8 mL vial. Then a cylindrical stirrer bar (10 x 6 mm) was added, followed by 4-dimethylaminopyridine (0.0377 g, 0.3084 mmol), pyridine (0.4968 mL, 6.1680 mmol) and acetic anhydride (0.8754 mL, 9.2520 mmol), and the vial was sealed. The reaction mixture was suspended with vortex genie 2 (scientific industries) and then it was immersed into a preheated to 100 °C silicon bath. Then the reaction mixture was stirred for 60 minutes at this temperature. The reaction mixture was quenched with 5 mL ethanol while it was still hot, and then the reaction mixture was transferred into 95 mL acetone to suspend. After stirring for a few hours, the formed cellulosic solids were filtrated off and were washed with ethanol (3 times). The solids were transferred back into 100 mL ethanol, and the suspension was allowed to stir overnight at room temperature. This step was repeated one more time, but instead of stirring overnight, it was stirred for few hours. After the final filtration, the obtained cellulose carbamate material was dried in a vacuum.

N.B! Work up at elevated temperatures helps the dispersion of the regenerated cellulose material and minimizes its capability of retention of reagent and or SB-IL.

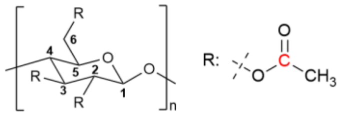

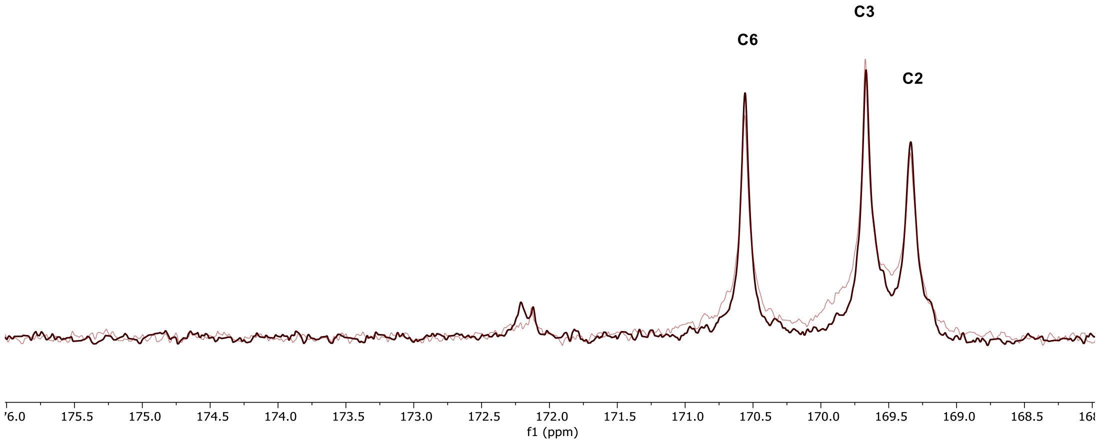

Figure S4 Comparison of the carbonyl region from quantitative ^{13}C NMR spectra of ***Sample A*** *(solid) and* ***Sample B*** *(pale) in DMSO-d_6.*

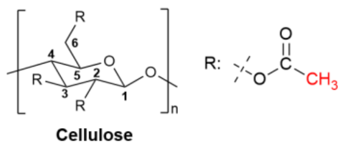

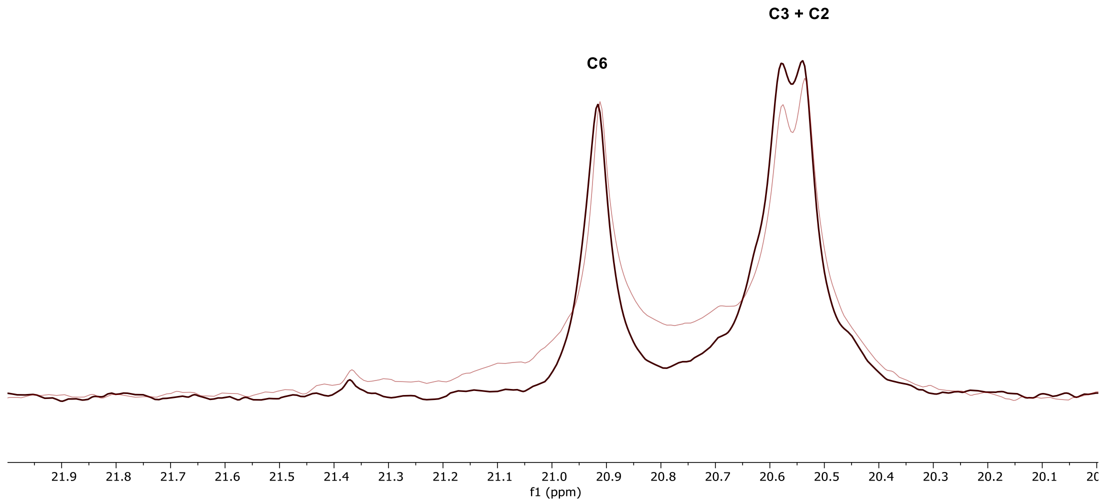

Figure S5 Comparison of the acetyl region from quantitative 13C NMR spectra of ***Sample A*** *(solid) and* ***Sample B*** *(pale) in DMSO-d_6.*

Dissolution tests for the prepared cellulose materials through isocyanate pathway

Table S2 Dissolution test of the prepared cellulosic materials through isocyanate reaction[a,b]

Entry	Cellulose derivate	DS	DMSO	DMA	γ-VL	Cyrene™	H_2O	1M AcOH[c]	0.1M NaOH[c]	1M NaOH[c]	2M NaOH[c]
		0.02	-	-	-	-	-[d]	-	-[d]	-[d]	*[d]
		0.03	-	-	-	-	-	-	-	-	*
		0.07	-	-	-	-	-	-	-	-	*
		0.08	-	-	-	-	-	-	-	-	*
		0.08	-	-	-	-	-	-	-	-	*
		0.10	-	-	-	-	-	-	-	-	-
**1**	*N*-phenyl carbamate	0.11	-	-	-	-	-	-	-	-	-
		0.12	-	-	-	-	-	-	-	-	-
		0.14	-	-	-	-	-	-	-	-	-
		0.27	-	-	-	-	-	-	-	-	-
		0.29	-	-	-	-	-	-	-	-	-
		0.51	+	-	-	-	-	-	-	-	-
		0.65	+	+	-	-	-	-	-	-	-
		0.04	-	-	-	-	-	-	-	-	-
		0.11	-	-	-	-	-	-	-	-	-
		0.14	-	-	-	-	-	-	-	-	-
**2**	*N*-butyl carbamate	0.14	-	-	-	-	-	-	-	-	-
		0.17	-	-	-	-	-	-	-	-	-
		0.23	-	-	-	-	-	-	-	-	-
		2.53	+	+	+	+	-	-	-	-	-

[a] 10 mg of each cellulosic material was suspended in 1 mL of the corresponding solvent. After the suspensions were stirred at 100 °C for 20 h, the dissolution was confirmed. [b] A chart depicting the structures of the prepared cellulosic materials could be found in the next section of the ESI. [c] Dissolutions in these solvents were performed at room temperature utilizing the same quantities. - not dissolved, + dissolved, * opalescent (no visible particles were observed by eye) [d] Optical microscope images can be found in the next section of ESI.

Optical microscopy images

Selected samples were studied with the help of an optical microscope (Olympus BX51TF microscope, equipped with DP70 colour camera and adjustable polarized lenses). The Cross-polarizer angle was optimized for better image contrast, producing differences in colours.

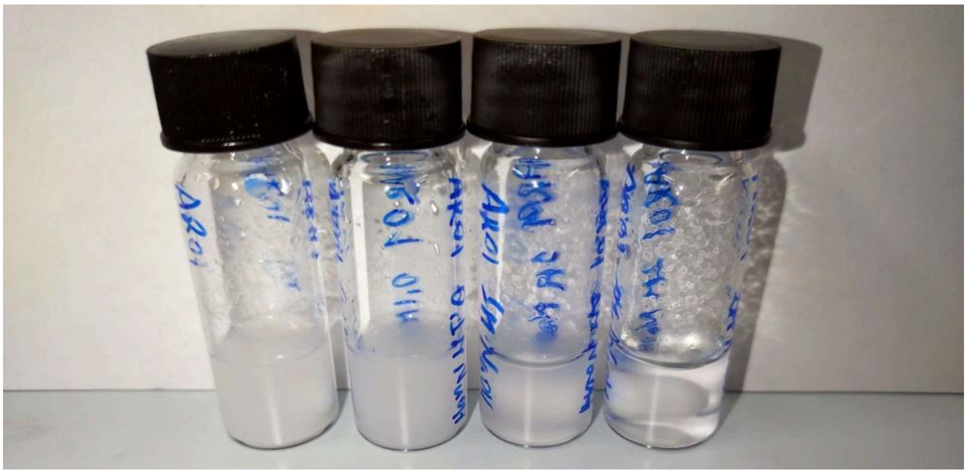

Figure S6 Cellulose N-phenyl carbamates (DS 0.03) in different solvents. From left to right H_2O, 0.1M NaOH, 1M NaOH and 2M NaOH.

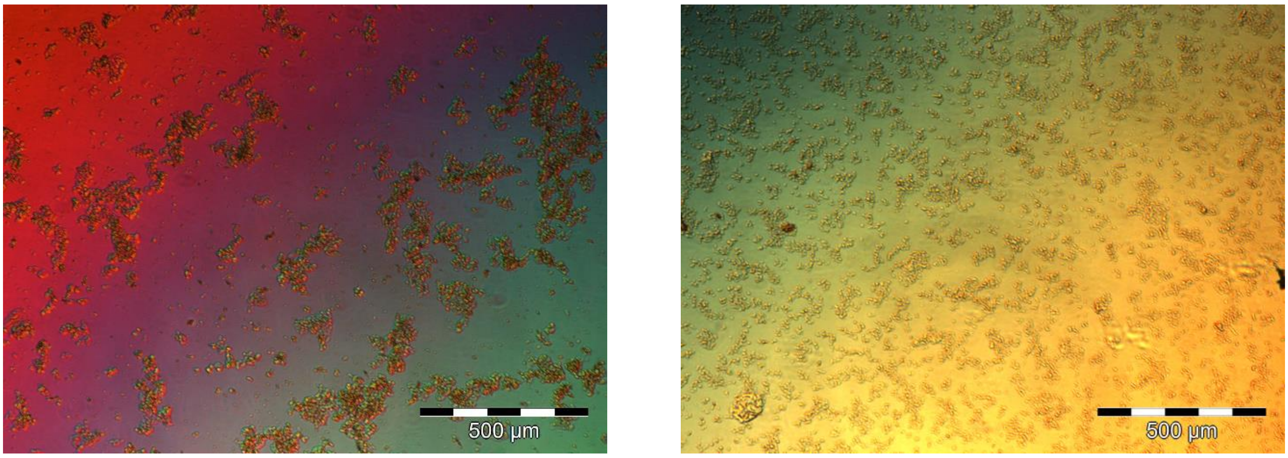

Figure S7 Microscopy images of cellulose N-phenyl carbamate with DS 0.03 in: H_2O (left) and 0.1M NaOH (right)

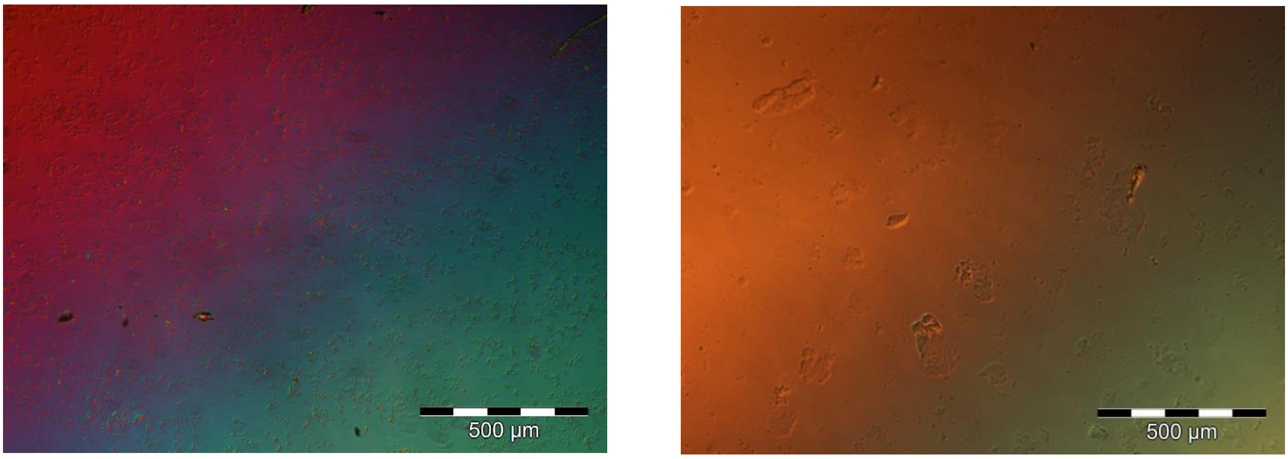

Figure S8 Microscopy images of cellulose N-phenyl carbamate with DS 0.03 in: 1M NaOH (left) and 2M NaOH (right)

Cellulose materials – schematic representation

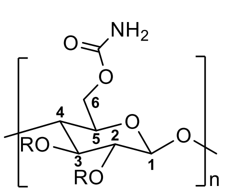

R: H or $-CONH_2$

Cellulose carbamate

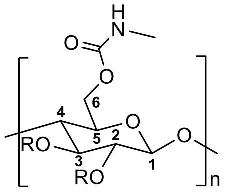

R: H or $-CONHCH_3$

Cellulose *N*-methyl carbamate

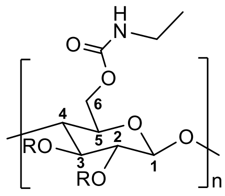

R: H or $-CONHCH_2CH_3$

Cellulose *N*-ethyl carbamate

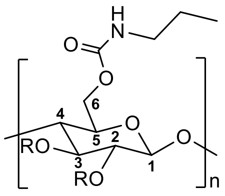

R: H or $-CONHCH_2CH_2CH_3$

Cellulose *N*-propyl carbamate

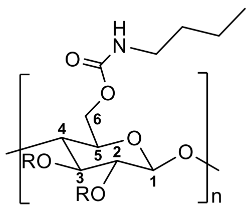

R: H or $-CONHCH_2(CH_2)_2CH_3$

Cellulose *N*-butyl carbamate

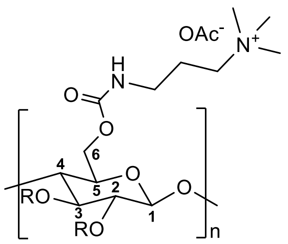

R: H or $-CONHCH_2CH_2CH_2N^+(CH_3)_3\ O^-Ac$

Cellulose *N*-(*N*,*N*,*N*-) trimethylpropan-1-aminium acetate carbamate

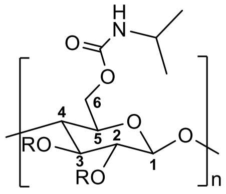

R: H or $-CONHCH(CH_3)_2$

Cellulose *N*-*iso*-propyl carbamate

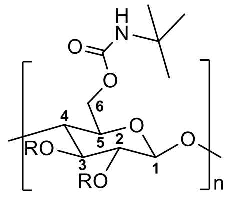

R: H or $-CONHC(CH_3)_3$

Cellulose *N*-*tert*-Butyl carbamate

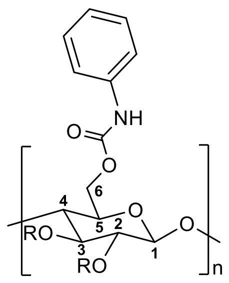

R: H or -CONHPh

Cellulose *N*-phenyl carbamate

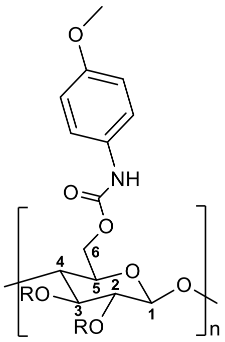

R: H or -CONH(4-MeO)-Ph

Cellulose *N*-4-methoxyphenyl carbamate

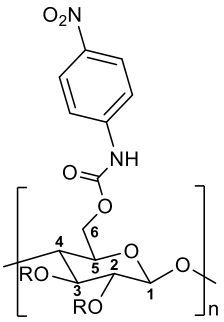

R: H or $-CONH(4\text{-}NO_2)\text{-}Ph$

Cellulose *N*-4-nitrophenyl carbamate

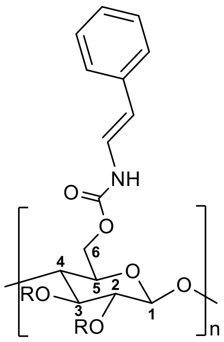

R: H or -CONHCH=CH-Ph

Cellulose *N*-styryl carbamate

Chart S1. Schematic representation of the prepared cellulose carbamate materials

Synthesis of cellulose materials

General procedure - transcarbamoylation / transurethanization

Condition 1: Microcrystalline cellulose (0.5000 g, 3.0837 mmol) was charged in a 100 mL round bottom flask followed by an oval stirrer bar (30 x 16 mm) and 10 mL [mTBNH][OAc]. The viscous reaction mixture was suspended with vortex genie 2 (scientific industries), and then it was immersed into a preheated to 80, 90, or 100 °C silicon bath. MCC was stirred for 30 minutes until it was completely dissolved. After the dissolution, 6 equivalents of the desired reagent were added to the reaction mixture. Then, the reaction mixture was stirred for 20 h at the desired temperature (80, 90, or 100 °C). After that, the reaction mixture was quenched with 80 mL EtOH while it was still hot, and then the heating was ceased. After cooling, the diluted reaction mixture was transferred into 500 mL EtOH to suspend. After stirring for a few hours, the formed cellulosic solids were filtrated off and were washed with EtOH (3 times). The solids were transferred back into 500 mL EtOH, and the suspension was allowed to stir overnight at room temperature. This step was repeated one more time, but instead of stirring overnight, it was stirred for few hours. After the final filtration, the obtained cellulose carbamate material was dried in a vacuum.

Condition 2: Following the same reaction conditions as in Condition 1 with an exception in the used amount of the desired reagent. Here, 9 equivalents of the desired reagent were used.

Condition 3: Following the same reaction conditions as in Conditions 1 with an exception in the used amount of the desired reagent. Here, 12 equivalents of the desired reagent were used.

N.B! Work up at elevated temperatures helps the dispersion of the regenerated cellulose material and minimizes its capability of retention of reagent and or SB-IL.

Cellulose carbamate

DS 0.07: The material was obtained by following the general procedure – **condition 1**. Starting from MCC (0.5000 g, 3.0837 mmol), methyl carbamate (1.3890 g, 18.5025 mmol) and 10 mL [mTBNH][OAc]. Reaction time was 20 h and the temperature was set at 100 °C.

DS 0.11: The material was obtained by following the general procedure – **condition 2**. Starting from MCC (0.5000 g, 3.0837 mmol), methyl carbamate (2.0835 g, 27.7537 mmol) and 10 mL [mTBNH][OAc]. Reaction time was 20 h and the temperature was set at 100 °C.

DS 0.19: The material was obtained by following the general procedure – **condition 3**. Starting from MCC (0.5000 g, 3.0837 mmol), methyl carbamate (2.7780 g, 37.0049 mmol) and 10 mL [mTBNH][OAc]. Reaction time was 20 h and the temperature was set at 100 °C.

Cellulose *N*-methyl carbamate

DS 0.09: The material was obtained by following the general procedure – **condition 1**. Starting from MCC (0.5000 g, 3.0837 mmol), methyl *N*-methyl carbamate (1.6484 g, 18.5025 mmol) and 10 mL [mTBNH][OAc]. Reaction time was 20 h and the temperature was set at 100 °C.

DS 0.17: The material was obtained by following the general procedure – **condition 2**. Starting from MCC (0.5000 g, 3.0837 mmol), methyl *N*-methyl carbamate (2.4726 g, 27.7537 mmol) and 10 mL [mTBNH][OAc]. Reaction time was 20 h and the temperature was set at 100 °C.

DS 0.18: The material was obtained by following the general procedure – **condition 3**. Starting from MCC (0.5000 g, 3.0837 mmol), methyl *N*-methyl carbamate (3.2968 g, 37.0049 mmol) and 10 mL [mTBNH][OAc]. Reaction time was 20 h and the temperature was set at 100 °C.

Cellulose *N*-ethyl carbamate

DS 0.13: The material was obtained by following the general procedure – **condition 1**. Starting from MCC (0.5000 g, 3.0837 mmol), methyl *N*-ethyl carbamate (1.9080 g, 18.5025 mmol) and 10 mL [mTBNH][OAc]. Reaction time was 20 h and the temperature was set at 100 °C.

DS 0.17: The material was obtained by following the general procedure – **condition 2**. Starting from MCC (0.5000 g, 3.0837 mmol), methyl *N*-ethyl carbamate (2.8620 g, 27.7537 mmol) and 10 mL [mTBNH][OAc]. Reaction time was 20 h and the temperature was set at 100 °C.

DS 0.22: The material was obtained by following the general procedure – **condition 3**. Starting from MCC (0.5000 g, 3.0837 mmol), methyl *N*-ethyl carbamate (3.8159 g, 37.0049 mmol) and 10 mL [mTBNH][OAc]. Reaction time was 20 h and the temperature was set at 100 °C.

Cellulose *N*-propyl carbamate

DS 0.10: The material was obtained by following the general procedure – **condition 1**. Starting from MCC (0.5000 g, 3.0837 mmol), methyl *N*-propyl carbamate (2.1676 g, 18.5025 mmol) and 10 mL [mTBNH][OAc]. Reaction time was 20 h and the temperature was set at 100 °C.

DS 0.14: The material was obtained by following the general procedure – **condition 2**. Starting from MCC (0.5000 g, 3.0837 mmol), methyl *N*-propyl carbamate (3.2513 g, 27.7537 mmol) and 10 mL [mTBNH][OAc]. Reaction time was 20 h and the temperature was set at 100 °C.

DS 0.22: The material was obtained by following the general procedure – **condition 3**. Starting from MCC (0.5000 g, 3.0837 mmol), methyl *N*-propyl carbamate (4.3351 g, 37.0049 mmol) and 10 mL [mTBNH][OAc]. Reaction time was 20 h and the temperature was set at 100 °C.

Cellulose *N*-butyl carbamate

DS 0.13: The material was obtained by following the general procedure – **condition 1**. Starting from MCC (0.5000 g, 3.0837 mmol), methyl *N*-butyl carbamate (2.4272 g, 18.5025 mmol) and 10 mL [mTBNH][OAc]. Reaction time was 20 h and the temperature was set at 100 °C.

DS 0.17: The material was obtained by following the general procedure – **condition 2**. Starting from MCC (0.5000 g, 3.0837 mmol), methyl *N*-butyl carbamate (3.6407 g, 27.7537 mmol) and 10 mL [mTBNH][OAc]. Reaction time was 20 h and the temperature was set at 100 °C.

DS 0.18: The material was obtained by following the general procedure – **condition 3**. Starting from MCC (0.5000 g, 3.0837 mmol), methyl *N*-butyl carbamate (4.8543 g, 37.0049 mmol) and 10 mL [mTBNH][OAc]. Reaction time was 20 h and the temperature was set at 100 °C.

Cellulose *N*-(N,N,N-)trimethylpropan-1-aminium acetate carbamate

DS 0.10: The material was obtained by following the general procedure – **condition 1**. Starting from MCC (0.5000 g, 3.0837 mmol), methyl *N*-butyl carbamate (4.3351 g, 18.5025 mmol) and 10 mL [mTBNH][OAc]. Reaction time was 20 h and the temperature was set at 100 °C.

DS 0.10: The material was obtained by following the general procedure – **condition 2**. Starting from MCC (0.5000 g, 3.0837 mmol), methyl *N*-butyl carbamate (6.5027 g, 27.7537 mmol) and 10 mL [mTBNH][OAc]. Reaction time was 20 h and the temperature was set at 100 °C.

DS 0.14: The material was obtained by following the general procedure – **condition 3**. Starting from MCC (0.5000 g, 3.0837 mmol), methyl *N*-butyl carbamate (8.6702 g, 37.0049 mmol) and 10 mL [mTBNH][OAc]. Reaction time was 20 h and the temperature was set at 100 °C.

Cellulose *N-iso*-propyl carbamate

DS 0.04: The material was obtained by following the general procedure – **condition 1**. Starting from MCC (0.5000 g, 3.0837 mmol), methyl *N-iso*-propyl carbamate (2.1676 g, 18.5025 mmol) and 10 mL [mTBNH][OAc]. Reaction time was 20 h and the temperature was set at 100 °C.

DS 0.05: The material was obtained by following the general procedure – **condition 2**. Starting from MCC (0.5000 g, 3.0837 mmol), methyl *N-iso*-propyl carbamate (3.2513 g, 27.7537 mmol) and 10 mL [mTBNH][OAc]. Reaction time was 20 h and the temperature was set at 100 °C.

DS 0.08: The material was obtained by following the general procedure – **condition 3**. Starting from MCC (0.5000 g, 3.0837 mmol), methyl *N-iso*-propyl carbamate (4.3351 g, 37.0049 mmol) and 10 mL [mTBNH][OAc]. Reaction time was 20 h and the temperature was set at 100 °C.

Cellulose *N-tert*-butyl carbamate

DS 0.04: The material was obtained by following the general procedure – **condition 1**. Starting from MCC (0.5000 g, 3.0837 mmol), methyl *N-tert*-butyl carbamate (2.4272 g, 18.5025 mmol) and 10 mL [mTBNH][OAc]. Reaction time was 20 h and the temperature was set at 100 °C.

DS 0.05: The material was obtained by following the general procedure – **condition 2**. Starting from MCC (0.5000 g, 3.0837 mmol), methyl *N-tert*-butyl carbamate (3.6407 g, 27.7537 mmol) and 10 mL [mTBNH][OAc]. Reaction time was 20 h and the temperature was set at 100 °C.

DS 0.06: The material was obtained by following the general procedure – **condition 3**. Starting from MCC (0.5000 g, 3.0837 mmol), methyl *N-tert*-butyl carbamate (4.8543 g, 37.0049 mmol) and 10 mL [mTBNH][OAc]. Reaction time was 20 h and the temperature was set at 100 °C.

Cellulose *N*-phenyl carbamate

DS 0.14: The material was obtained by following the general procedure – **condition 1**. Starting from MCC (0.5000 g, 3.0837 mmol), methyl *N*-phenyl carbamate (2.7970 g, 18.5025 mmol) and 10 mL [mTBNH][OAc]. Reaction time was 20 h and the temperature was set at 80 °C.

DS 0.14: The material was obtained by following the general procedure – **condition 2**. Starting from MCC (0.5000 g, 3.0837 mmol), methyl *N*-phenyl carbamate (4.1955 g, 27.7537 mmol) and 10 mL [mTBNH][OAc]. Reaction time was 20 h and the temperature was set at 80 °C.

DS 0.16: The material was obtained by following the general procedure – **condition 3**. Starting from MCC (0.5000 g, 3.0837 mmol), methyl *N*-phenyl carbamate (5.5940 g, 37.0049 mmol) and 10 mL [mTBNH][OAc]. Reaction time was 20 h and the temperature was set at 80 °C.

Cellulose *N*-4-methoxyphenyl carbamate

DS 0.09: The material was obtained by following the general procedure – **condition 1**. Starting from MCC (0.5000 g, 3.0837 mmol), methyl *N*-4-methoxyphenyl carbamate (3.3525 g, 18,5025 mmol) and 10 mL [mTBNH][OAc]. Reaction time was 20 h and the temperature was set at 80 °C.

DS 0.09: The material was obtained by following the general procedure – **condition 2**. Starting from MCC (0.5000 g, 3.0837 mmol), methyl *N*-4-methoxyphenyl carbamate (5.0287 g, 27.7537 mmol) and 10 mL [mTBNH][OAc]. Reaction time was 20 h and the temperature was set at 80 °C.

DS 0.09: The material was obtained by following the general procedure – **condition 3**. Starting from MCC (0.5000 g, 3.0837 mmol), methyl *N*-4-methoxyphenyl carbamate (6.7049 g, 37.0049 mmol) and 10 mL [mTBNH][OAc]. Reaction time was 20 h and the temperature was set at 80 °C.

Cellulose *N*-4-nitrophenyl carbamate

DS 0.05: The material was obtained by following the general procedure – **condition 1**. Starting from MCC (0.5000 g, 3.0837 mmol), methyl *N*-4-nitrophenyl carbamate (3.6294 g, 18,5025 mmol) and 10 mL [mTBNH][OAc]. Reaction time was 20 h and the temperature was set at 80 °C.

DS 0.06: The material was obtained by following the general procedure – **condition 2**. Starting from MCC (0.5000 g, 3.0837 mmol), methyl *N*-4-nitrophenyl carbamate (5.4441 g, 27.7537 mmol) and 10 mL [mTBNH][OAc]. Reaction time was 20 h and the temperature was set at 80 °C.

DS 0.08: The material was obtained by following the general procedure – **condition 3**. Starting from MCC (0.5000 g, 3.0837 mmol), methyl *N*-4-nitrophenyl carbamate (7.2589 g, 37.0049 mmol) and 10 mL [mTBNH][OAc]. Reaction time was 20 h and the temperature was set at 80 °C.

Cellulose *N*-styryl carbamate

DS 0.10: The material was obtained by following the general procedure – **condition 1**. Starting from MCC (0.5000 g, 3.0837 mmol), methyl *N*-styryl carbamate (3.2786 g, 18,5025 mmol) and 10 mL [mTBNH][OAc]. Reaction time was 20 h and the temperature was set at 90 °C.

DS 0.21: The material was obtained by following the general procedure – **condition 2**. Starting from MCC (0.5000 g, 3.0837 mmol), methyl *N*-styryl carbamate (4.9180 g, 27.7537 mmol) and 10 mL [mTBNH][OAc]. Reaction time was 20 h and the temperature was set at 90 °C.

DS 0.32: The material was obtained by following the general procedure – **condition 3**. Starting from MCC (0.5000 g, 3.0837 mmol), methyl *N*-styryl carbamate (6.5573 g, 37.0049 mmol) and 10 mL [mTBNH][OAc]. Reaction time was 20 h and the temperature was set at 90 °C.

General procedure – isocyanate pathway

Condition A: Microcrystalline cellulose (0.5000 g, 3.0837 mmol) was charged in a 100 mL round bottom flask followed by an oval stirrer bar (30 x 16 mm) and 10 mL [mTBNH][OAc]. The viscous reaction mixture was suspended with vortex genie 2 (scientific industries) and then it was immersed into a preheated to 80 °C silicon bath. MCC was stirred for 30 minutes until it was completely dissolved. After the dissolution, the desired amount of reagent was added to the reaction mixture. Then, the reaction mixture was stirred further for 20 h at 80 °C. After that, the reaction mixture was quenched with 80 mL EtOH while it was still hot, and then the heating was ceased. After cooling, the diluted reaction mixture was transferred into 500 mL EtOH to suspend. After stirring for a few hours, the formed cellulosic solids were filtrated off and were washed with EtOH (x 3 times). The solids were transferred back into 500 mL EtOH, and the suspension was allowed to stir overnight at room temperature. This step was repeated one more time, but instead of stirring overnight, it was stirred for few hours. After the final filtration, the obtained cellulose carbamate material was dried in a vacuum.

Condition B: Microcrystalline cellulose (0.2500 g, 1.5419 mmol) was charged in a 50 mL round bottom flask followed by an oval stirrer bar (20 x 10 mm) and 5 mL [mTBNH][OAc]. The viscous reaction mixture was suspended with vortex genie 2 (scientific industries) and then it was immersed into a preheated to 80 °C silicon bath. MCC was stirred for 30 minutes until it was completely dissolved. After the dissolution, the desired amount of reagent was added to the reaction mixture. Then, the reaction mixture was stirred

further for 20 h at 80 °C. After that, the reaction mixture was quenched with 40 mL EtOH while it was still hot, and then the heating was ceased. After cooling the diluted reaction mixture was transferred into 250 mL EtOH to suspend. After stirring for a few hours, the formed cellulosic solids were filtrated off and were washed with EtOH (x 3 times). The solids were transferred back into 250 mL EtOH, and the suspension was allowed to stir overnight at room temperature. This step was repeated one more time, but instead of stirring overnight, it was stirred for few hours. After the final filtration, the obtained cellulose carbamate material was dried in a vacuum.

Cellulose *N*-phenyl carbamate

DS 0.02: The material was obtained by following the general procedure – **condition A**. Starting from MCC (0.5000 g, 3.0837 mmol), phenyl isocyanate (0.1685 mL, 1.5419 mmol) and 10 mL [mTBNH][OAc]. Reaction time was 20 h and the temperature was set at 80 °C.

DS 0.03: The material was obtained by following the general procedure – **condition A**. Starting from MCC (0.5000 g, 3.0837 mmol), phenyl isocyanate (0.3370 mL, 3.0837 mmol) and 10 mL [mTBNH][OAc]. Reaction time was 20 h and the temperature was set at 80 °C.

DS 0.08: The material was obtained by following the general procedure – **condition B**. Starting from MCC (0.2500 g, 1.5419 mmol), phenyl isocyanate (0.1685 mL, 1.5419 mmol) and 5 mL [mTBNH][OAc]. Reaction time was 20 h and the temperature was set at 80 °C.

DS 0.07: The material was obtained by following the general procedure – **condition A**. Starting from MCC (0.5000 g, 3.0837 mmol), phenyl isocyanate (0.5056 mL, 4.6256 mmol) and 10 mL [mTBNH][OAc]. Reaction time was 20 h and the temperature was set at 80 °C.

DS 0.08: The material was obtained by following the general procedure – **condition A**. Starting from MCC (0.5000 g, 3.0837 mmol), phenyl isocyanate (0.6741 mL, 6.1674 mmol) and 10 mL [mTBNH][OAc]. Reaction time was 20 h and the temperature was set at 80 °C.

DS 0.10: The material was obtained by following the general procedure – **condition A**. Starting from MCC (0.5000 g, 3.0837 mmol), phenyl isocyanate (0.8426 mL, 7.7093 mmol) and 10 mL [mTBNH][OAc]. Reaction time was 20 h and the temperature was set at 80 °C.

DS 0.11: The material was obtained by following the general procedure – **condition A**. Starting from MCC (0.5000 g, 3.0837 mmol), phenyl isocyanate (1.0111 mL, 9.2511 mmol) and 10 mL [mTBNH][OAc]. Reaction time was 20 h and the temperature was set at 80 °C.

DS 0.14: The material was obtained by following the general procedure – **condition B**. Starting from MCC (0.2500 g, 1.5419 mmol), phenyl isocyanate (0.5056 mL, 4.6257 mmol) and 5 mL [mTBNH][OAc]. Reaction time was 20 h and the temperature was set at 80 °C.

DS 0.12: The material was obtained by following the general procedure – **condition A** with exception of the used reaction temperature. Starting from MCC (0.5000 g, 3.0837 mmol), phenyl isocyanate (1.0111 mL, 9.2511 mmol) and 10 mL [mTBNH][OAc]. Reaction time was 20 h and the temperature was set at 65 °C.

DS 0.27: The material was obtained by following the general procedure – **condition A**. Starting from MCC (0.5000 g, 3.0837 mmol), phenyl isocyanate (2.0223 mL, 18.5022 mmol) and 10 mL [mTBNH][OAc]. Reaction time was 20 h and the temperature was set at 80 °C.

DS 0.29: The material was obtained by following the general procedure – **condition B**. Starting from MCC (0.2500 g, 1.5419 mmol), phenyl isocyanate (1.0111 mL, 9.2511 mmol) and 5 mL [mTBNH][OAc]. Reaction time was 20 h and the temperature was set at 80 °C.

DS 0.51: The material was obtained by following the general procedure – **condition B**. Starting from MCC (0.2500 g, 1.5419 mmol), phenyl isocyanate (1.5168 mL, 13.8771 mmol) and 5 mL [mTBNH][OAc]. Reaction time was 20 h and the temperature was set at 80 °C.

DS 0.65: The material was obtained by following the general procedure – **condition B**. Starting from MCC (0.2500 g, 1.5419 mmol), phenyl isocyanate (2.0224 mL, 18.5028 mmol) and 5 mL [mTBNH][OAc]. Reaction time was 20 h and the temperature was set at 80 °C.

Cellulose *N*-butyl carbamate

DS 0.04: The material was obtained by following the general procedure – **condition A**. Starting from MCC (0.5000 g, 3.0837 mmol), butyl isocyanate (0.1736 mL, 1.5419 mmol) and 10 mL [mTBNH][OAc]. Reaction time was 20 h and the temperature was set at 80 °C.

DS 0.11: The material was obtained by following the general procedure – **condition A**. Starting from MCC (0.5000 g, 3.0837 mmol), butyl isocyanate (0.3472 mL, 3.0837 mmol) and 10 mL [mTBNH][OAc]. Reaction time was 20 h and the temperature was set at 80 °C.

DS 0.14: The material was obtained by following the general procedure – **condition A**. Starting from MCC (0.5000 g, 3.0837 mmol), butyl isocyanate (0.5208 mL, 4.6256 mmol) and 10 mL [mTBNH][OAc]. Reaction time was 20 h and the temperature was set at 80 °C.

DS 0.14: The material was obtained by following the general procedure – **condition A**. Starting from MCC (0.5000 g, 3.0837 mmol), butyl isocyanate (0.6944 mL, 6.1674 mmol) and 10 mL [mTBNH][OAc]. Reaction time was 20 h and the temperature was set at 80 °C.

DS 0.17: The material was obtained by following the general procedure – **condition A**. Starting from MCC (0.5000 g, 3.0837 mmol), butyl isocyanate (0.8681 mL, 7.7093 mmol) and 10 mL [mTBNH][OAc]. Reaction time was 20 h and the temperature was set at 80 °C.

DS 0.23: The material was obtained by following the general procedure – **condition A**. Starting from MCC (0.5000 g, 3.0837 mmol), butyl isocyanate (1.0417 mL, 9.2511 mmol) and 10 mL [mTBNH][OAc]. Reaction time was 20 h and the temperature was set at 80 °C.

DS 2.53: The material was obtained by following the general procedure – **condition A**. Starting from MCC (0.5000 g, 3.0837 mmol), butyl isocyanate (2.0833 mL, 18.5022 mmol) and 10 mL [mTBNH][OAc]. Reaction time was 20 h and the temperature was set at 80 °C.

Synthesis of methyl *N*-substituted carbamates

General procedure

The corresponding amine (200 mmol) was charged in a 250 mL round bottom flask followed by an oval stirrer bar (30 x 16 mm) and 40 mL of MeOH. To the clear solution, 1000 mmol of dimethyl carbonate (DMC) was added, followed by 10 mol% of base (mTBN) or Lewis acid ($Zn(OAc)_2*2H_2O$) catalyst. Then, the reaction mixture was immersed into a preheated to 80 °C silicon oil bath and was stirred for a specific time (typically 24 hours). After that, the excess of DMC was removed by distillation (N.B! If the DMC should be reused a fractional distillation is required). The obtained crude reaction mixtures were purified by one of the following methods: 1) For the methyl *N*-aliphatic carbamates, typically the purification includes vacuum distillation. 2) For the methyl *N*-aromatic carbamates, the crude products were re-dissolved in acetone and were precipitated with water. The formed solids were filtrated off and were washed thoroughly with water. Then the solids were dried in a vacuum.

Methyl-*N*-ethyl carbamate

The titled compound was synthesized following the general procedure. Exceptionally, due to the low boiling point of the starting material, the reaction was performed in a sealed autoclave vessel. Starting from ethyl amine (15.0300 mL, 100 mmol, used as 30 w.% MeOH solution), DMC (42.0935 mL, 500 mmol), mTBN (1.3920 g, 10 mmol) and 20 mL MeOH. Reaction time was 24 hours, and the temperature was set at 80 °C. The crude was purified by vacuum distillation (temperature of vapours: 47 °C at 6 mbar). Yield 65.3 % (6.7346 g, 65.3084 mmol). The characterization data of the obtained compound agrees with the literature values.[6]

Characterization data: Colourless liquid; **^{1}H NMR** (400 MHz; DMSO-*d₆*) δ_h 7.06 (s, 1H), 3.51 (s, 3H), 3.00 (qd, *J* = 7.2, 5.7 Hz, 2H), 1.00 (t, *J* = 7.2 Hz, 3H); **^{13}C NMR** (101 MHz, DMSO-d_6) δ_c 157.0, 51.5, 35.5, 15.5.

Methyl-*N*-propyl carbamate

The titled compound was synthesized following the general procedure. Starting from propyl amine (16.4423 mL, 200 mmol), DMC (84.1869 mL, 1000 mmol), mTBN (2.7840 g, 20 mmol) and 40 mL MeOH. Reaction time was 24 hours, and the temperature was set at 80 °C. The crude was purified by vacuum distillation (temperature of vapours: 55 °C at 1.5 mbar). Yield 74.4 % (17.4370 g, 148.8434 mmol). The characterization data of the obtained compound agrees with the literature values.[7,8]

Characterization data: Colourless liquid; **^{1}H NMR** (400 MHz; DMSO-d_6) δ_h 7.08 (s, 1H), 3.51 (s, 3H), 2.93 (td, *J* = 7.1, 5.8 Hz, 2H), 1.40 (h, *J* = 7.4 Hz, 2H), 0.83 (t, *J* = 7.4 Hz, 3H); **^{13}C NMR** (101 MHz, DMSO-d_6) δ_c 157.2, 51.5, 42.5, 23.1, 11.6.

Methyl-*N*-butyl carbamate

The titled compound was synthesized following the general procedure. Starting from butyl amine (19.7946 mL, 200 mmol), DMC (84.1869 mL, 1000 mmol), mTBN (2.7840 g, 20 mmol) and 40 mL MeOH. Reaction time was 24 hours, and the temperature was set at 80 °C. The crude was purified by vacuum distillation (temperature of vapours: 47-48 °C at 0.1 mbar). Yield 77.8 % (20.4130 g, 155.6106 mmol). The characterization data of the obtained compound agrees with the literature values.[7,9]

Characterization data: Colourless liquid; **^{1}H NMR** (400 MHz; DMSO-d_6) δ_h 7.05 (s, 1H), 3.51 (s, 3H), 2.97 (td, *J* = 6.9, 5.7 Hz, 2H), 1.44 – 1.32 (m, 2H), 1.31 – 1.18 (m, 2H), 0.86 (t, *J* = 7.3 Hz, 3H); **^{13}C NMR** (101 MHz, DMSO-d_6) δ_c 157.1, 51.5, 40.3, 32.0, 19.8, 14.1.

Methyl-*N*-*iso*-propyl carbamate

Synthesis 1: The titled compound was synthesized following the general procedure. Exceptionally, due to the low boiling point of the starting material, the reaction was performed in a sealed autoclave vessel. Starting from *iso*-propyl amine (8.5916 mL, 100 mmol), DMC (42.0935 mL, 500 mmol), mTBN (1.3920 g, 10 mmol) and 20 mL MeOH. Reaction time was 24 hours, and the temperature was set at 80 °C. The crude was purified by vacuum distillation (temperature of vapours: 42 °C at 0.1 mbar). Yield 32.8 % (3.840 g, 32.8126 mmol).

Synthesis 2: The titled compound was synthesized following the general procedure. Exceptionally, due to the low boiling point of the starting material, the reaction was performed in a sealed autoclave vessel. Starting from *iso*-propyl amine (8.5916 mL, 100 mmol), DMC (42.0935 mL, 500 mmol), $Zn(OAc)_2*2H_2O$ (2.1950 g, 10 mmol) and 20 mL MeOH. Reaction time was 24 hours, and the temperature was set at 80 °C. The crude was purified by vacuum distillation (temperature of vapours: 42 °C at 0.1 mbar). Yield 35.0 % (4.0960 g, 34.9637 mmol).

Synthesis 3: The *iso*-propyl amine (3.0000 g, 50.7528 mmol) was charged in a 50 mL round bottom flask followed by an oval stirrer bar (20 x 10 mm), K_2CO_3 (7.0143 g, 50.7528 mmol) and 20 mL of DCM. The

formed suspension was immersed into an ice bath. To the ice-cold suspension, methyl chloroformate (3.93 mL, 50.7528 mmol) was added dropwise for 1 hour. After the addition, the reaction mixture was refluxed for 5 hours furthermore before it was allowed to cool to room temperature. Then the crude reaction mixture was diluted with 30 mL of deionised water and was extracted with DCM (2 x 25 mL). The combined organic layers were dried over Na_2SO_4, and then the crude product was concentrated under reduced pressure.[10] The crude was purified by vacuum distillation (temperature of vapours: 42 °C at 0.1 mbar). Yield 60.5 % (3.5958 g, 30.6940 mmol).

The characterization data of the obtained compound agrees with the literature values.[7,10,11]

Characterization data: Colourless liquid; ^{1}**H NMR** (500 MHz; DMSO-*d₆*) δ_h 6.97 (s, 1H), 3.59 (dq, *J* = 13.0, 6.5 Hz, 1H), 3.50 (s, 3H), 1.04 (d, *J* = 6.6 Hz, 6H); ^{13}**C NMR** (126 MHz, DMSO-d_6) δ_c 156.3, 51.4, 42.7, 23.0.

Methyl-*N*-*tert*-butyl carbamate

The *tert*-butyl amine (8.8119 mL, 83.8542 mmol) was charged in a 250 mL round bottom flask followed by an oval stirrer bar (30 x 15 mm), K_2CO_3 (11.5891 g, 83.8542 mmol) and 80 mL of DCM. The formed suspension was immersed into an ice bath. To the ice-cold suspension, methyl chloroformate (8.8119 mL, 83.8542 mmol) was added dropwise for 1/2 hour. After the addition, the reaction mixture was stirred at room temperature for 72 hours furthermore. Then, the crude reaction mixture was diluted with 50 mL of deionised water and was extracted with DCM (2 x 25 mL). The combined organic layers were dried over Na_2SO_4, and then the crude product was concentrated under reduced pressure. The crude was purified by vacuum distillation (temperature of vapours: 37 °C at 1.9 mbar). Yield 79.3 % (8.7240 g, 66.5040 mmol). The characterization data of the obtained compound agrees with the literature values.[11,12]

Characterization data: Colourless liquid; ^{1}**H NMR** (400 MHz; DMSO-d_6) δ_h 6.85 (s, 1H), 3.47 (s, 3H), 1.21 (s, 9H); ^{13}**C NMR** (101 MHz, DMSO-d_6) δ_c 155.6, 51.0, 49.7, 29.1.

Methyl-*N*-4-methoxyphenyl carbamate

Synthesis 1: The titled compound was synthesized following the general procedure. Exceptionally, Dean-Stark apparatus was used to remove excess of MeOH during the reaction. Starting from 4-methoxy aniline (12.3150 g, 100 mmol), DMC (42.0935 mL, 500 mmol), and mTBN (1.3920 g, 10 mmol). Reaction time was 48 hours, and the temperature was set at 100 °C. The crude product was purified by flash chromatography (SiO_2) using DCM as eluent, followed by further recrystallization from heptane. Yield 51.7 % (9.3620 g, 51.6695 mmol).

Synthesis 2: The titled compound was synthesized following the general procedure. Exceptionally, Dean-Stark apparatus was used to remove excess of MeOH during the reaction. Starting from 4-methoxy aniline (12.3150 g, 100 mmol), DMC (42.0935 mL, 500 mmol), and $Zn(OAc)_2$*$2H_2O$ (2.1950 g, 10 mmol). Reaction time was 48 hours, and the temperature was set at 100 °C. The crude product was purified by re-dissolution in acetone and precipitation from water. The formed solids were filtrated off and were washed thoroughly with water. After that, the solids were dried in a vacuum. Yield 72.4 % (13.1163 g, 72.3898 mmol).

The characterization data of the obtained compound agrees with the literature values.[13,14]

Characterization data: Off-white solid; ^{1}**H NMR** (500 MHz; DMSO-d_6) δ_h 9.42 (s, 1H), 7.35 (d, *J* = 8.4 Hz, 2H), 6.86 (d, *J* = 9.0 Hz, 2H), 3.71 (s, 3H), 3.64 (s, 3H); ^{13}**C NMR** (126 MHz, DMSO-d_6) δ_c 155.2, 154.6, 132.6, 120.3, 114.4, 55.6, 51.9.

Methyl-*N*-4-nitrophenyl carbamate

The titled compound was synthesized following the general procedure. Exceptionally, Dean-Stark apparatus was used to remove excess of MeOH during the reaction. Starting from 4-nitro aniline (13.8130 g, 100

mmol), DMC (42.0935 mL, 500 mmol), and mTBN (1.3920 g, 10 mmol). Reaction time was 48 hours, and the temperature was set at 100 °C. The crude product was purified by re-dissolution in acetone and precipitation from water. The formed solids were filtrated off and were washed thoroughly with water. After that the solids were dried in a vacuum. Yield 84.4 % (16.5500 g, 84.3699 mmol). The characterization data of the obtained compound agrees with the literature values.[11,13]

Characterization data: Yellow solid; **^{1}H NMR** (400 MHz; DMSO-d_6) δ_h 10.39 (s, 1H), 8.20 (d, J = 9.3 Hz, 2H), 7.69 (d, J = 9.3 Hz, 2H), 3.73 (s, 3H); **^{13}C NMR** (101 MHz, DMSO-d_6) δ_c 154.2, 146.1, 142.1, 125.5, 118.1, 52.6.

Methyl-*N*-styryl carbamate

Trans-3-phenylacrylamide (7.3585 g, 50.0000 mmol) was charged in a 500 mL round bottom flask followed by an oval stirrer bar (30 x 15 mm) and 250 mL of MeOH. The formed suspension was immersed into a preheated to 70 °C silicone oil bath until the suspension becomes clear. After that, the clear solution was cooled with an ice bath, and to the cold solution was added dropwise NaOCl (29.7760 mL, 40.0000 mmol, used as 10 w.% in water). After the addition, the reaction mixture was stirred for 18 hours at 70 °C. Then the reaction mixture was poured over 2.5 L of water, and the formed solids were filtrated off and were washed with plenty of water. The crude product was dissolved in EtOH and was precipitated with heptane. Yield 69.7 % (6.1730 g, 34.8363 mmol). The characterization data of the obtained compound agrees with the literature values.[15]

Characterization data: White-yellowish solid; **^{1}H NMR** (400 MHz; DMSO-d_6) δ_h 9.75 (d, J = 10.1 Hz, 1H), 7.37 – 7.22 (m, 4H), 7.21 – 7.06 (m, 2H), 6.04 (d, J = 14.6 Hz, 1H), 3.67 (s, 3H); **^{13}C NMR** (101 MHz, DMSO-d_6) δ_c 154.8, 137.1, 129.1, 126.2, 125.8, 125.3, 110.1, 52.5.

Iso-propyl-*N*-phenyl carbamate

Phenyl isocyanate (5.4644 mL, 50.0000 mmol) was charged in a 100 mL round bottom flask followed by an oval stirrer bar (20 x 10 mm) and *i*-PrOH (38.2290 mL, 500.0000mmol). Then, the reaction mixture was immersed into a preheated to 90 °C silicone oil bath for 2 hours. After that, the reaction mixture was evaporated to dryness, and the crude product was used as such without further purification. Yield 99.8 % (8.9431 g, 49.9000 mmol). The characterization data of the obtained compound agrees with the literature values.[16,17]

Characterization data: White solid; **^{1}H NMR** (400 MHz; DMSO-d_6) δ_h 9.53 (s, 1H), 7.47 (d, J = 7.3 Hz, 2H), 7.26 (t, J = 7.4 Hz, 2H), 6.97 (t, J = 7.3 Hz, 1H), 4.90 (hept, J = 6.2 Hz, 1H), 1.26 (d, J = 6.3 Hz, 6H); **^{13}C NMR** (101 MHz, DMSO-d_6) δ_c 153.6, 139.8, 129.1, 122.6, 118.6, 67.8, 22.4.

References

1 A. R. Todorov, A. W. T. King and I. Kilpeläinen, *RSC Adv.*, 2023, **13**, 5983–5992.
2 L. Fliri, K. Heise, T. Koso, A. R. Todorov, D. R. del Cerro, S. Hietala, J. Fiskari, I. Kilpeläinen, M. Hummel and A. W. T. King, *Nat. Protoc.*, 2023, **18**, 2084–2123.
3 A. F. Tarchoun, D. Trache, T. M. Klapötke, B. Krumm and M. Kofen, *Fuel*, 2021, **292**, 120347.
4 L. T. T. Vo, B. Široká, A. P. Manian and T. Bechtold, *Carbohydr. Polym.*, 2010, **82**, 1191–1197.
5 Y. Tezuka and Y. Tsuchiya, *Carbohydr. Res.*, 1995, **273**, 83–91.
6 Y. Imada, Y. Mitsue, K. Ike, K. Washizuka and S.-I. Murahashi, *Bull. Chem. Soc. Jpn.*, 1996, **69**, 2079–2090.
7 Q. Gu, J. Fang, Z. Xu, W. Ni, K. Kong and Z. Hou, *New J. Chem.*, 2018, **42**, 13054–13064.
8 D. Margetić, I. Z. Antonac, Z. Glasovac, M. Eckert-Maksić and L. Maksimović, *Synth. Commun.*, 2011, **41**, 2283–2289.
9 R. Zeng, L. Bao, H. Sheng, L. Sun, M. Chen, Y. Feng and M. Zhu, *RSC Adv.*, 2016, **6**, 78576–78584.
10 A. Millet, D. Dailler, P. Larini and O. Baudoin, *Angew. Chemie Int. Ed.*, 2014, **53**, 2678–2682.
11 A. Yoshimura, M. W. Luedtke and V. V. Zhdankin, *J. Org. Chem.*, 2012, **77**, 2087–2091.
12 M. Hutchby, C. E. Houlden, J. G. Ford, S. N. G. Tyler, M. R. Gagné, G. C. Lloyd-Jones and K. I. Booker-Milburn, *Angew. Chemie Int. Ed.*, 2009, **48**, 8721–8724.
13 Q. Zhang, H.-Y. Yuan, N. Fukaya, H. Yasuda and J.-C. Choi, *Green Chem.*, 2017, **19**, 5614–5624.
14 Y. Wei, H. Ding, S. Lin and F. Liang, *Org. Lett.*, 2011, **13**, 1674–1677.
15 Z. Zhuangyu, P. Yi, H. Honwen and K. Tsi-yu, *Synthesis (Stuttg).*, 1991, 539–542.
16 N. V. Reddy, K. R. Prasad, P. S. Reddy, M. Lakshmi Kantam and K. R. Reddy, *Org. Biomol. Chem.*, 2014, **12**, 2172–2175.
17 I. Dindarloo Inaloo and S. Majnooni, *New J. Chem.*, 2018, **42**, 13249–13255.

Copy of spectral data

^{1}H and diffusion-edited ^{1}H spectra of **Cellulose carbamate DS 0.07** in DMSO-d_6/LiCl solution. (R: H or $-CONH_2$)

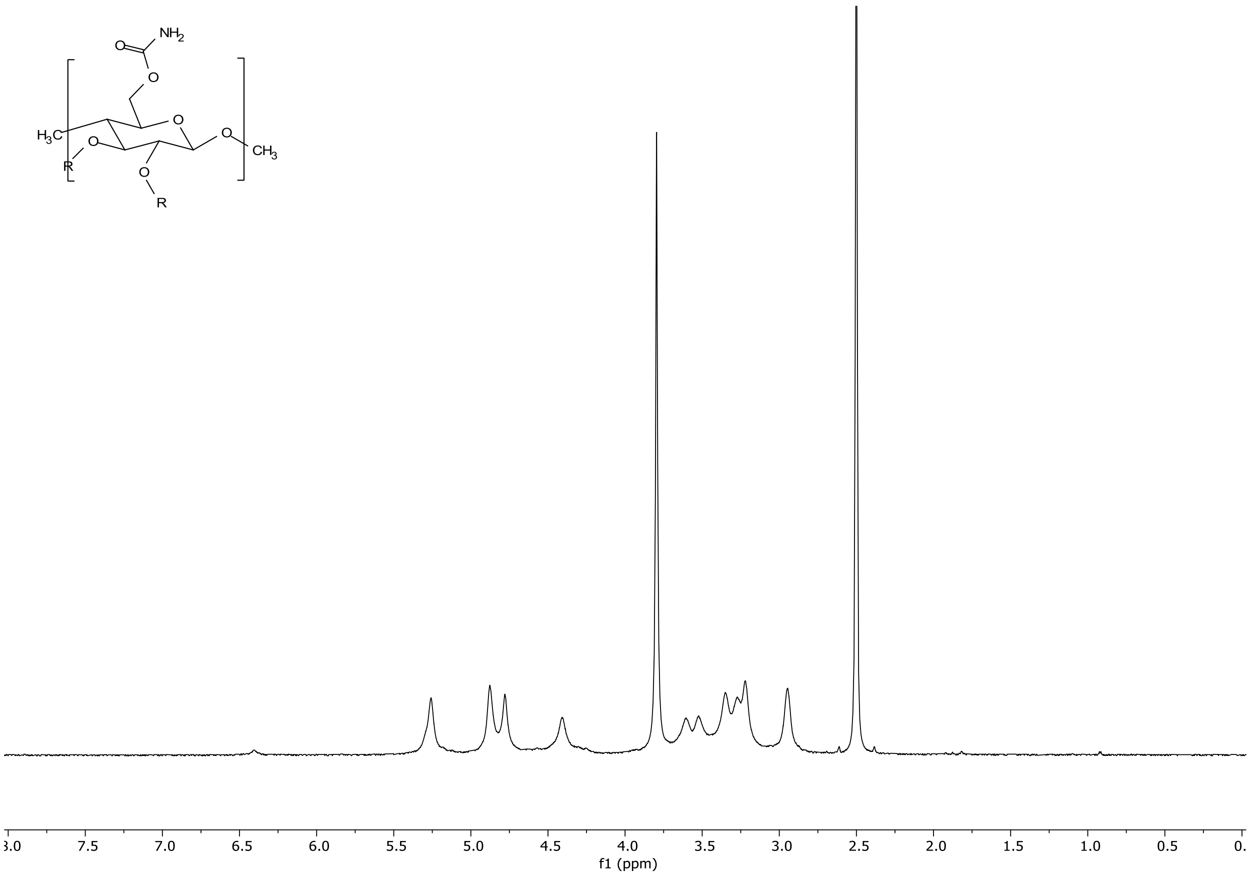

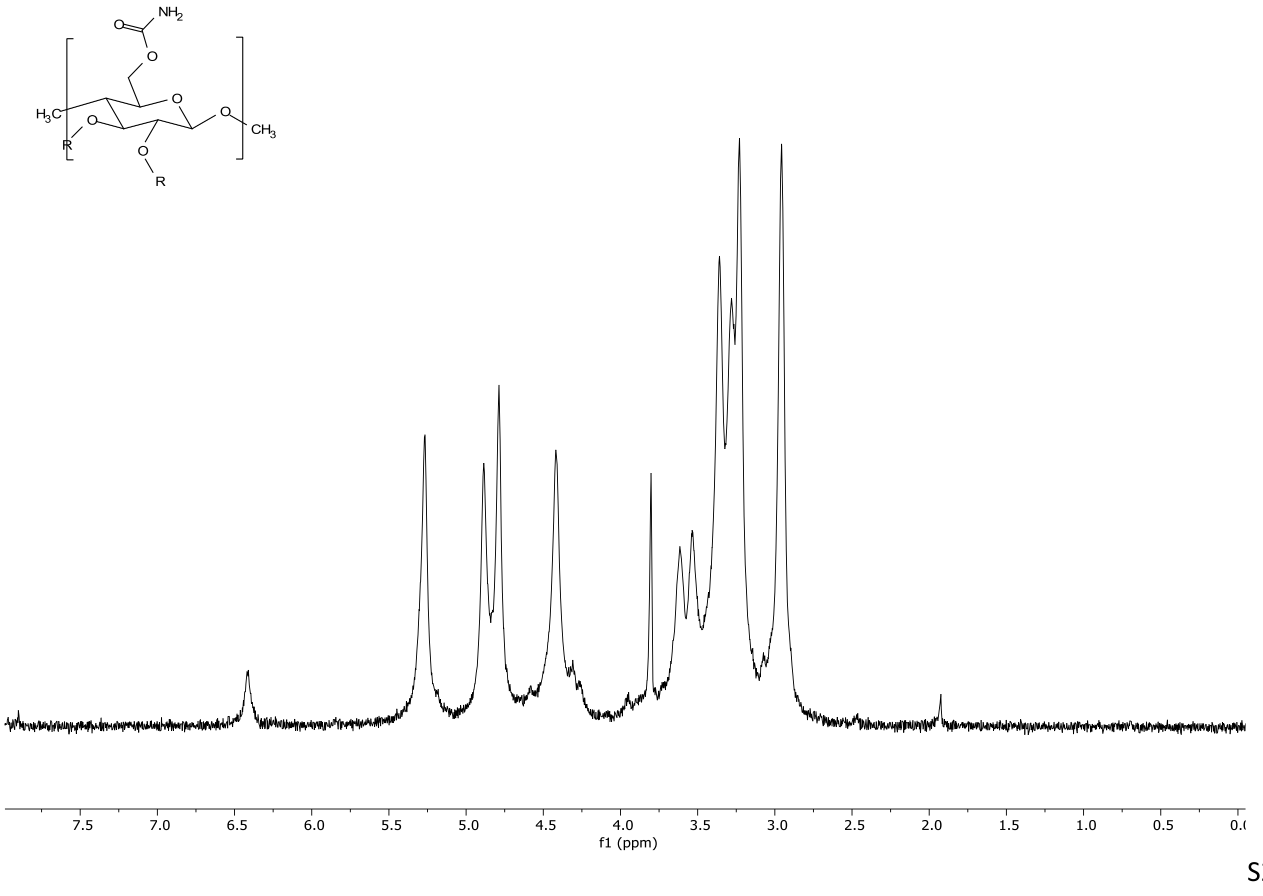

^{1}H and diffusion-edited ^{1}H spectra of **Cellulose carbamate DS 0.11** in DMSO-d_6/LiCl solution. (R: H or $-CONH_2$)

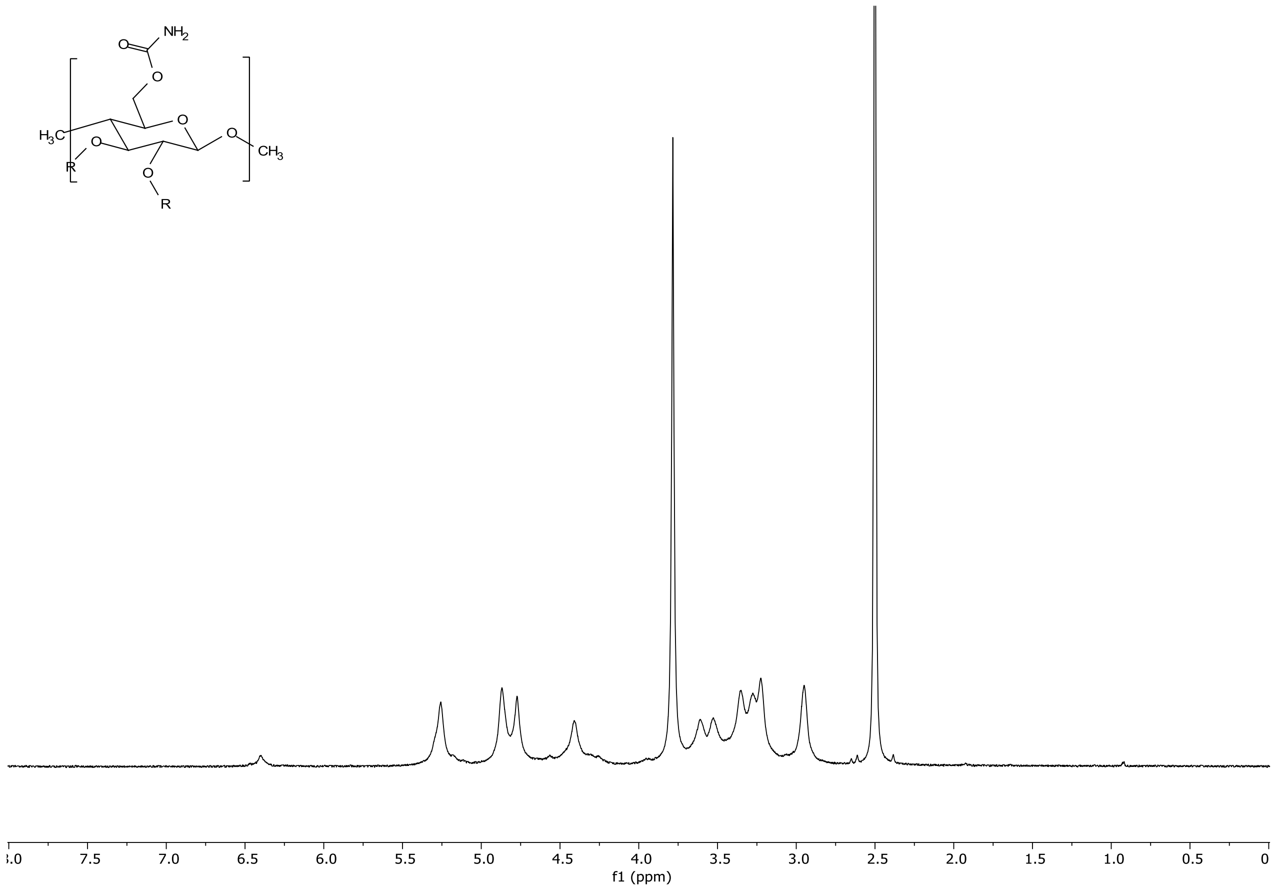

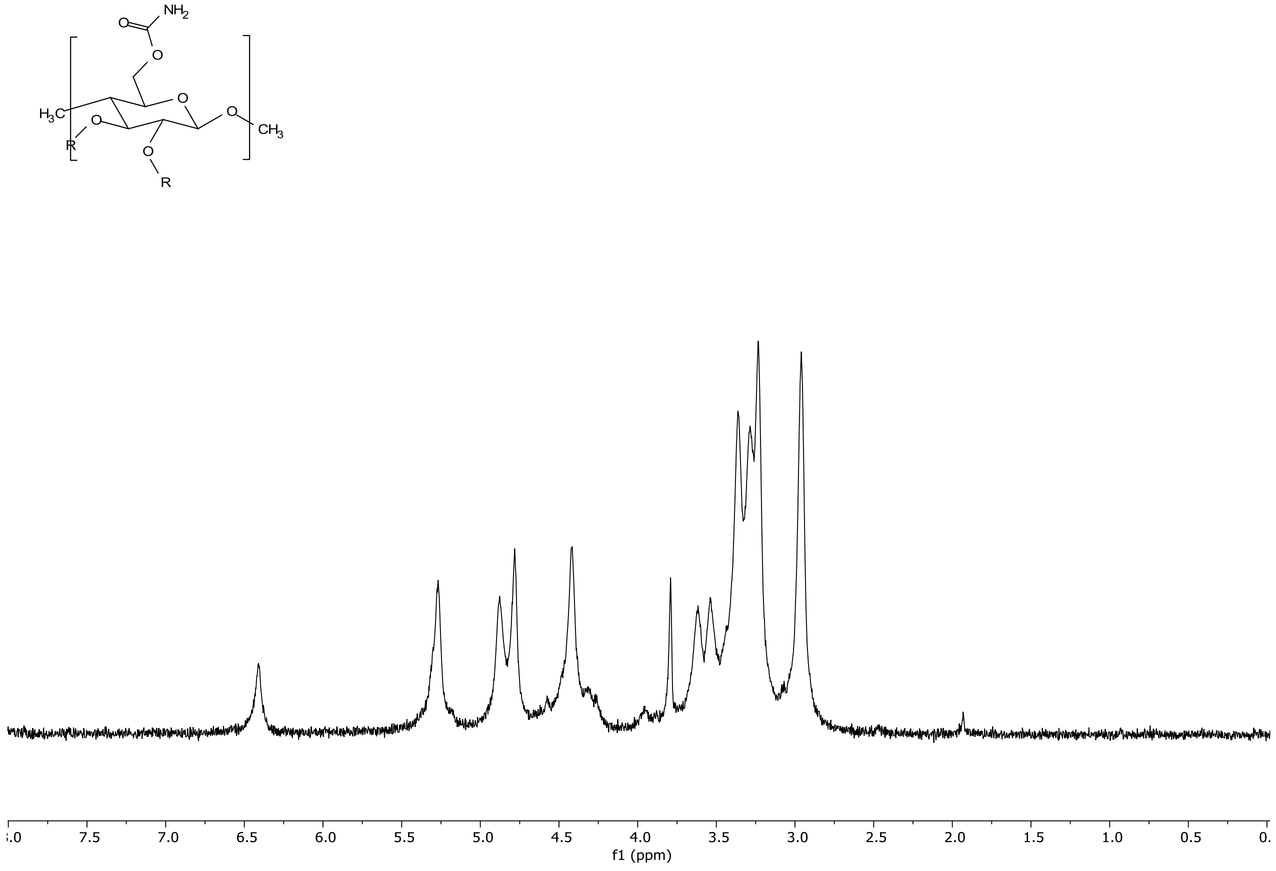

^{1}H and diffusion-edited ^{1}H spectra of **Cellulose carbamate DS 0.19** in DMSO-d_6/LiCl solution. (R: H or -CONH$_2$)

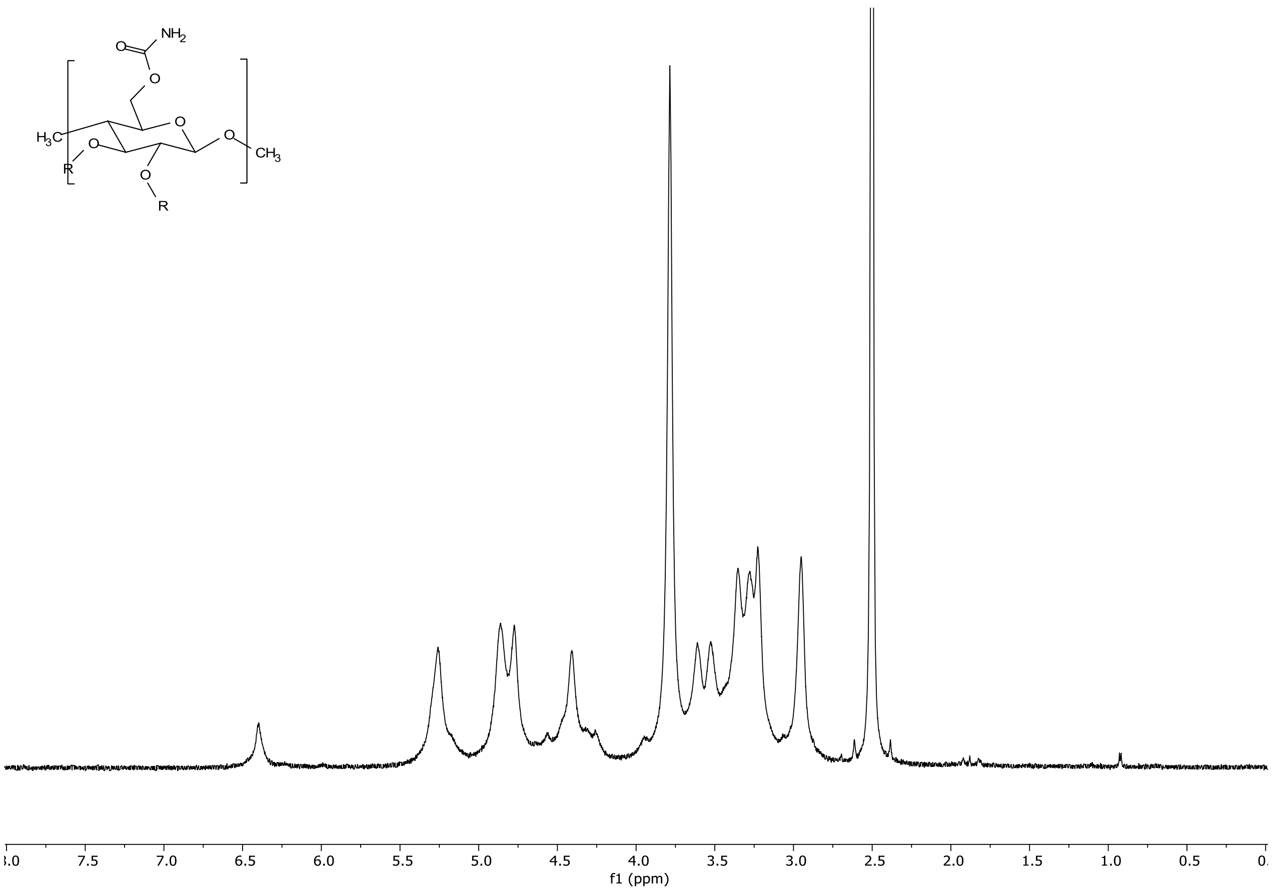

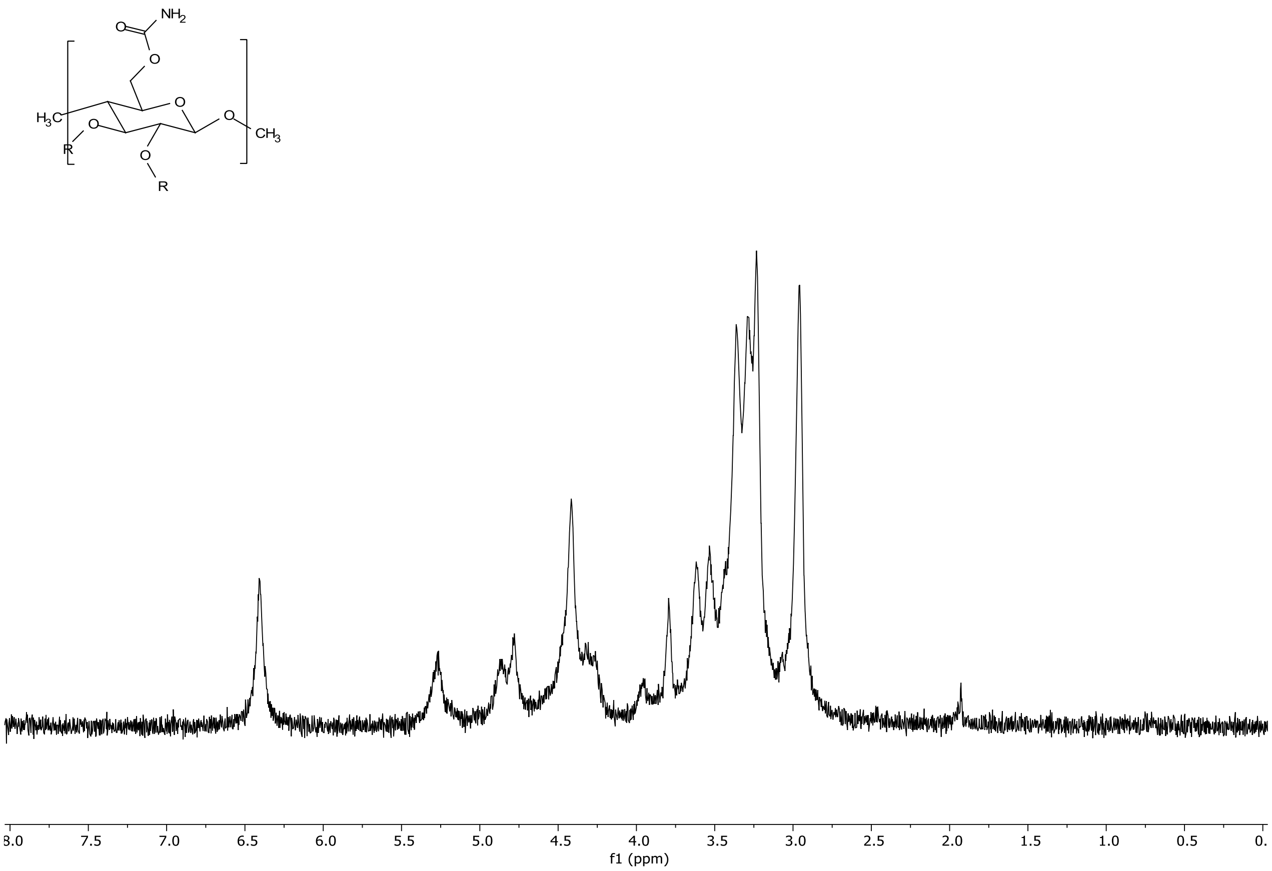

^{1}H and diffusion-edited ^{1}H spectra of **Cellulose *N*-methyl carbamate DS 0.09** in DMSO-d_6/LiCl solution. (R: H or -CONHCH$_3$)

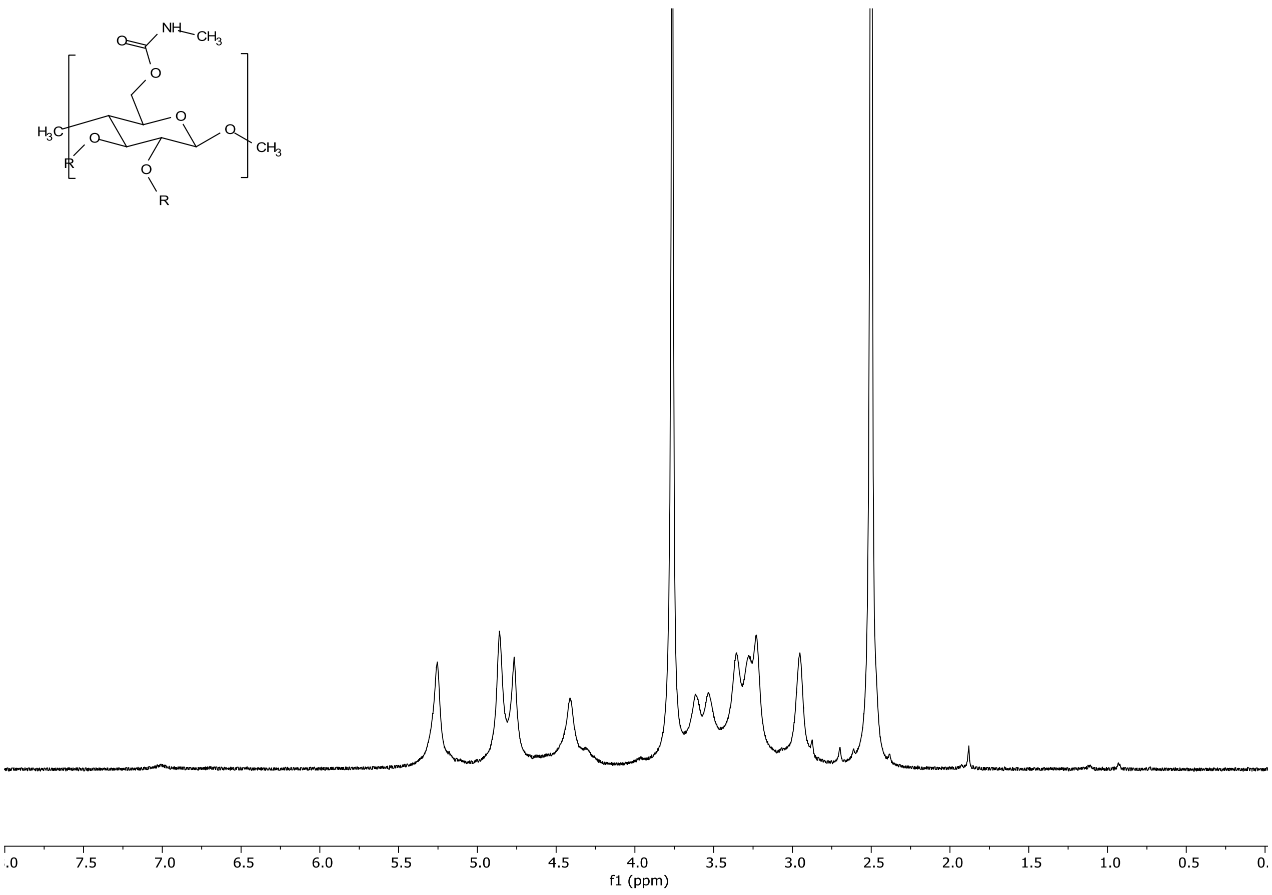

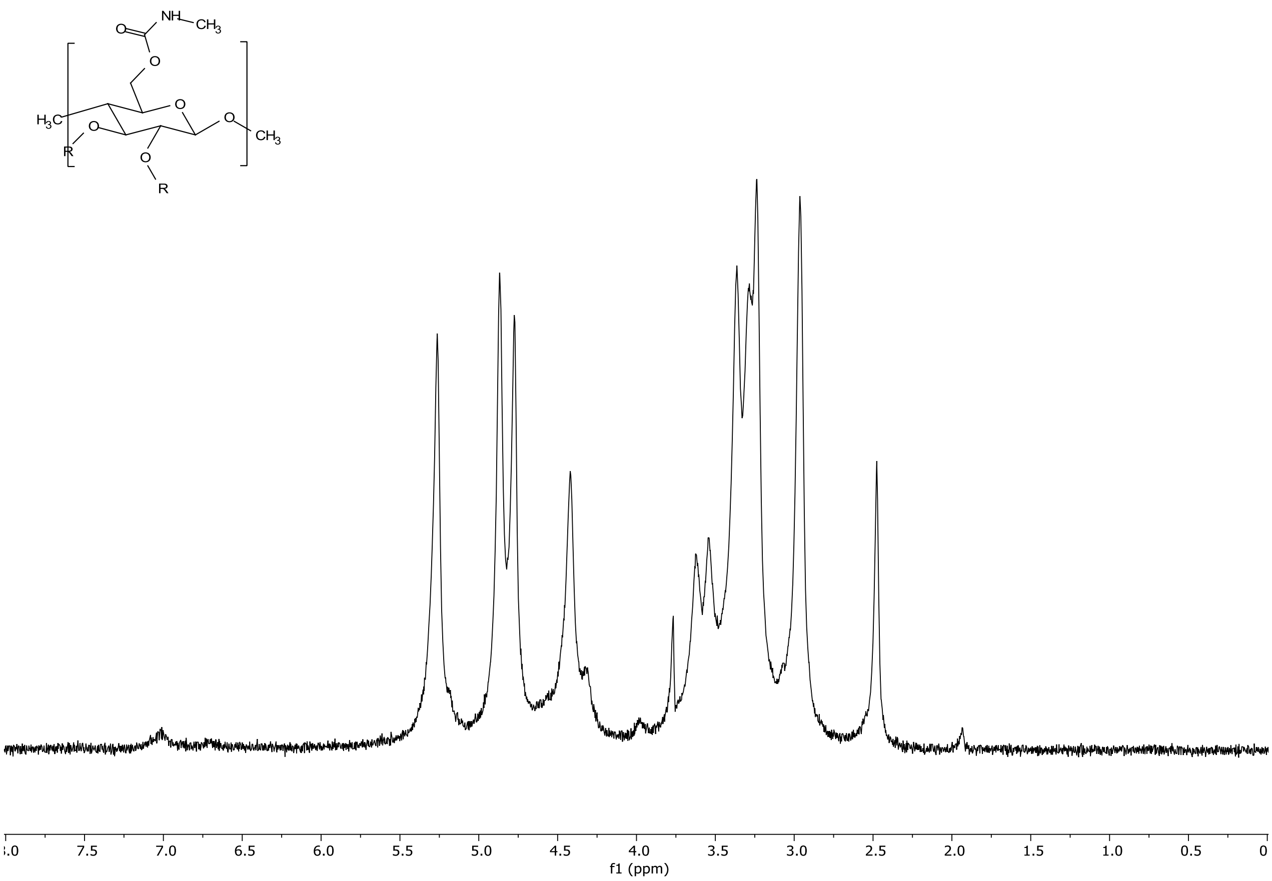

^{1}H and diffusion-edited ^{1}H spectra of **Cellulose *N*-methyl carbamate DS 0.17** in DMSO-d_6/LiCl solution. (R: H or -CONHCH$_3$)

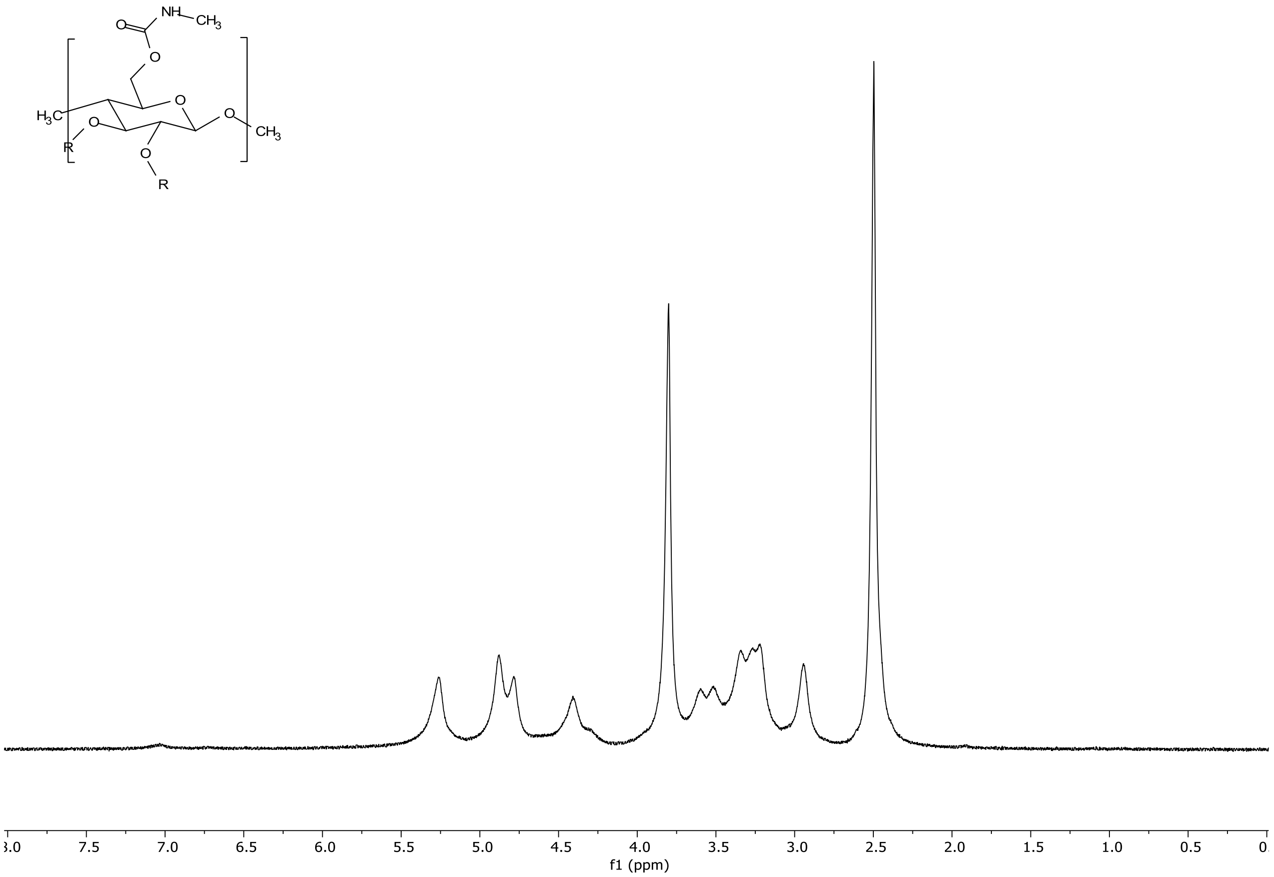

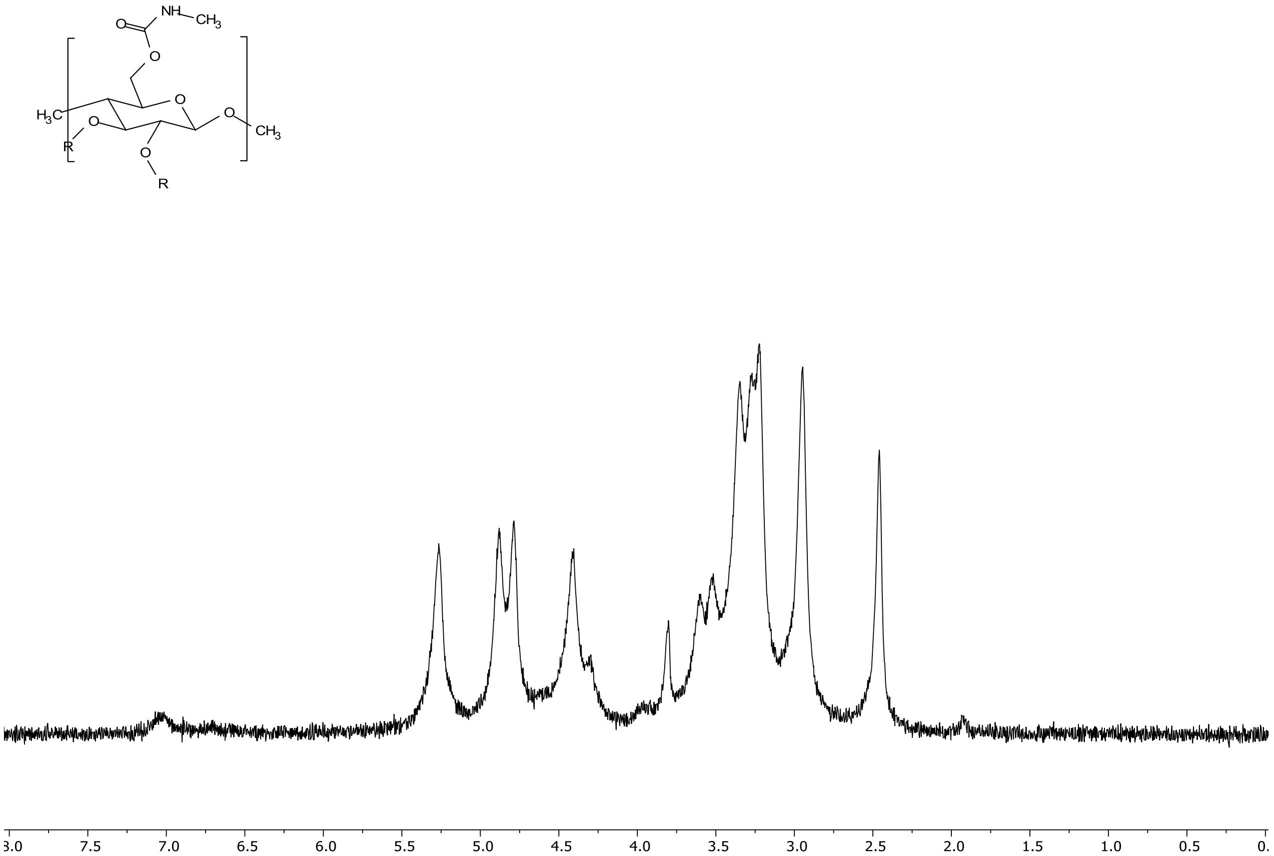

^{1}H and diffusion-edited ^{1}H spectra of **Cellulose *N*-methyl carbamate DS 0.18** in DMSO-d_6/LiCl solution. (R: H or -CONHCH$_3$)

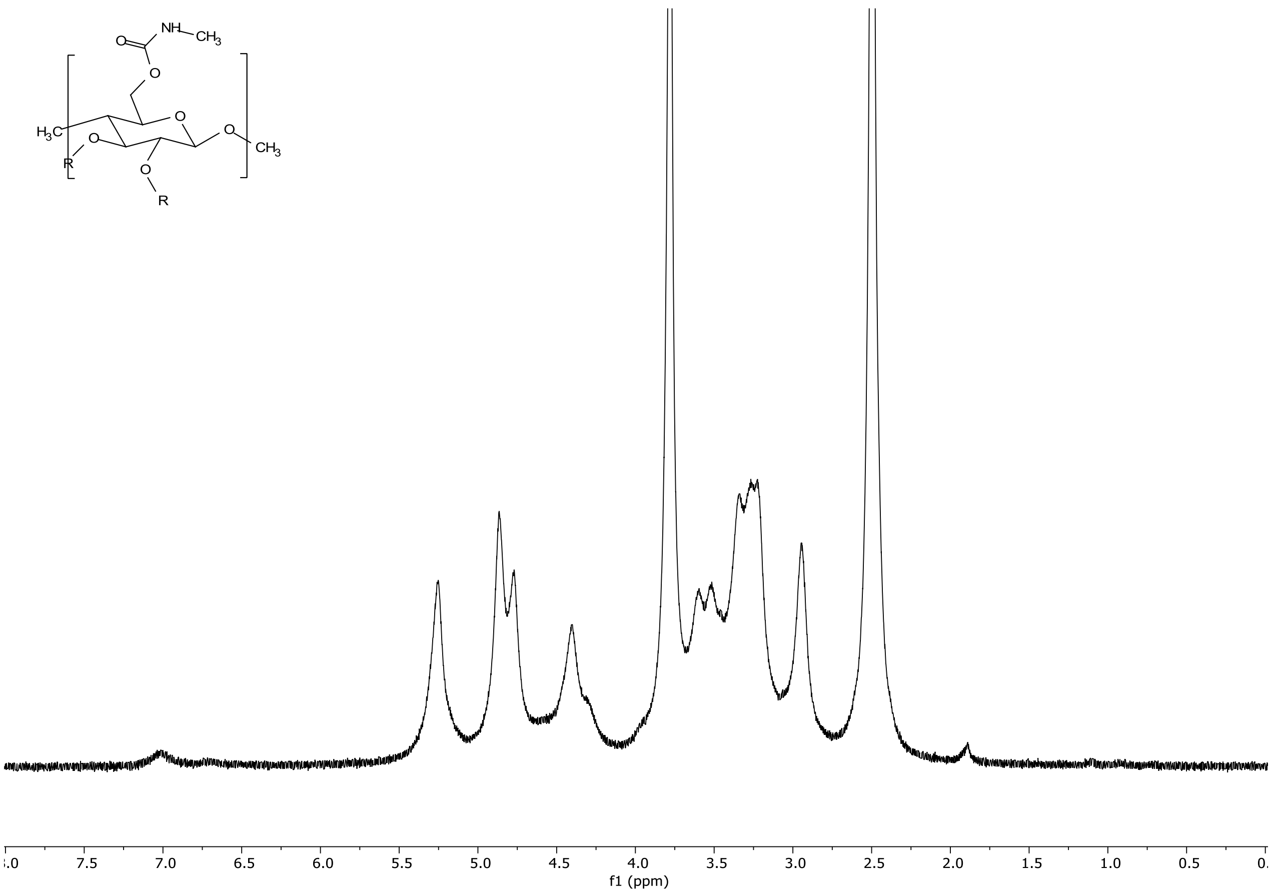

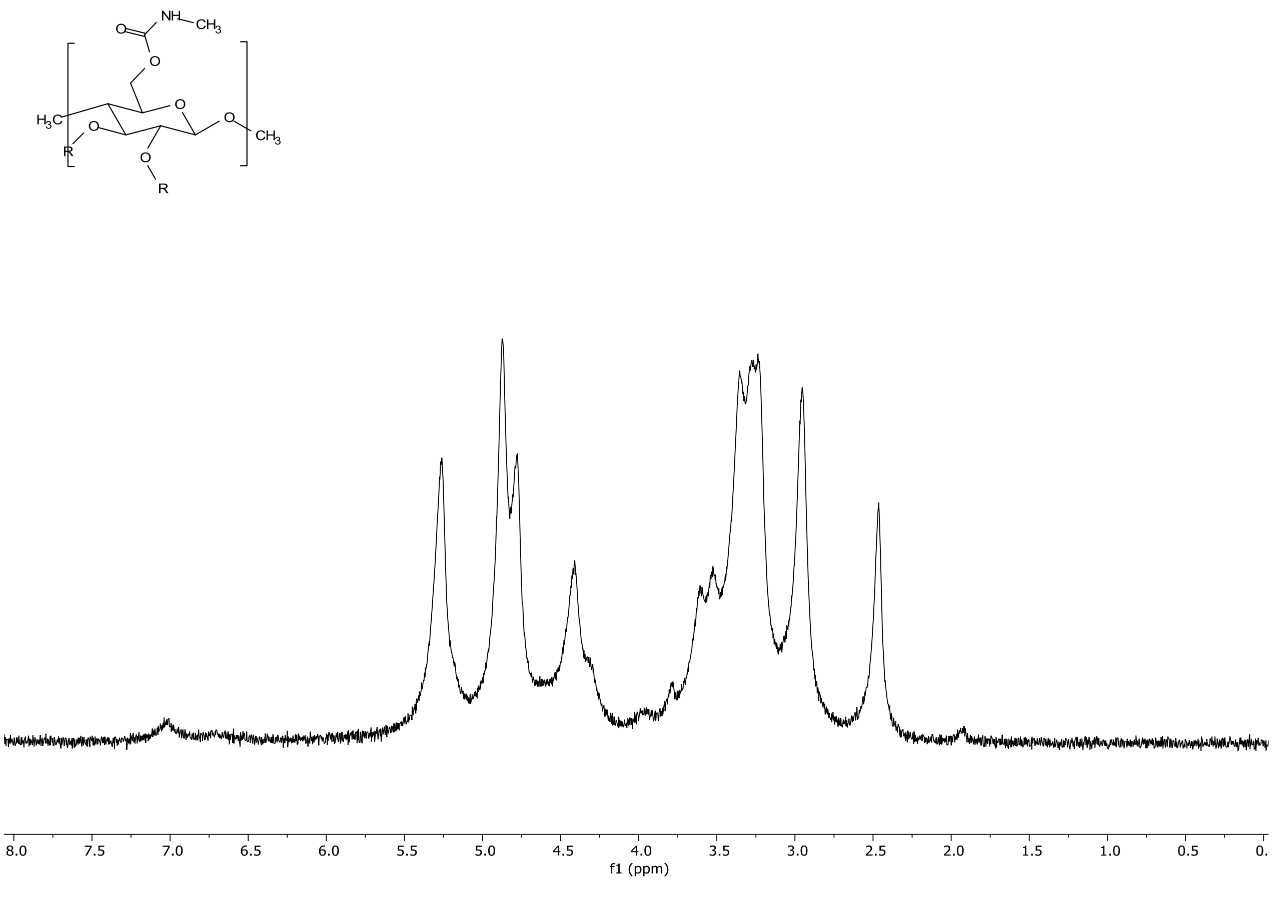

^{1}H and diffusion-edited ^{1}H spectra of **Cellulose *N*-ethyl carbamate DS 0.13** in DMSO-d_6/LiCl solution. (R: H or $-CONHCH_2CH_3$)

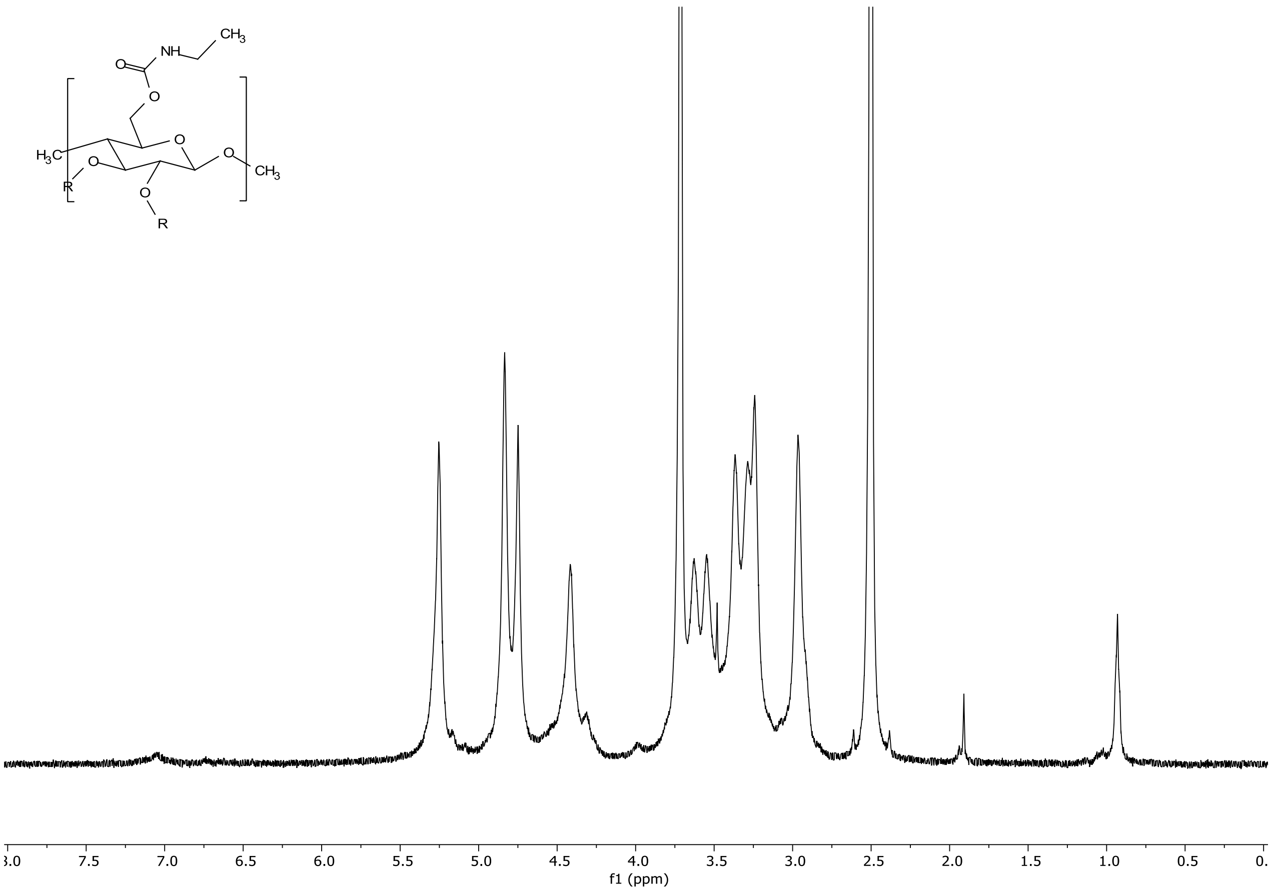

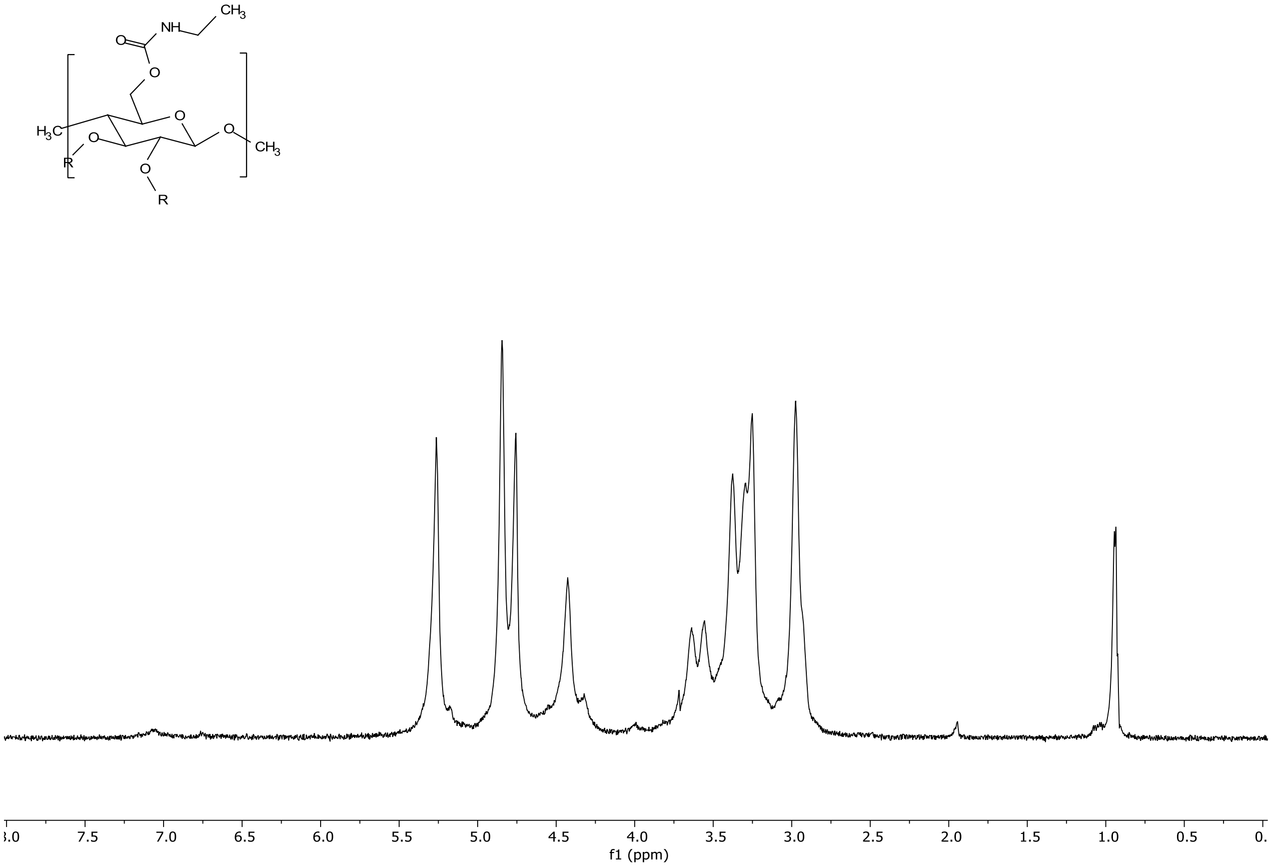

^{1}H and diffusion-edited ^{1}H spectra of **Cellulose *N*-ethyl carbamate DS 0.17** in DMSO-d_6/LiCl solution. (R: H or -CONHCH$_2$CH$_3$)

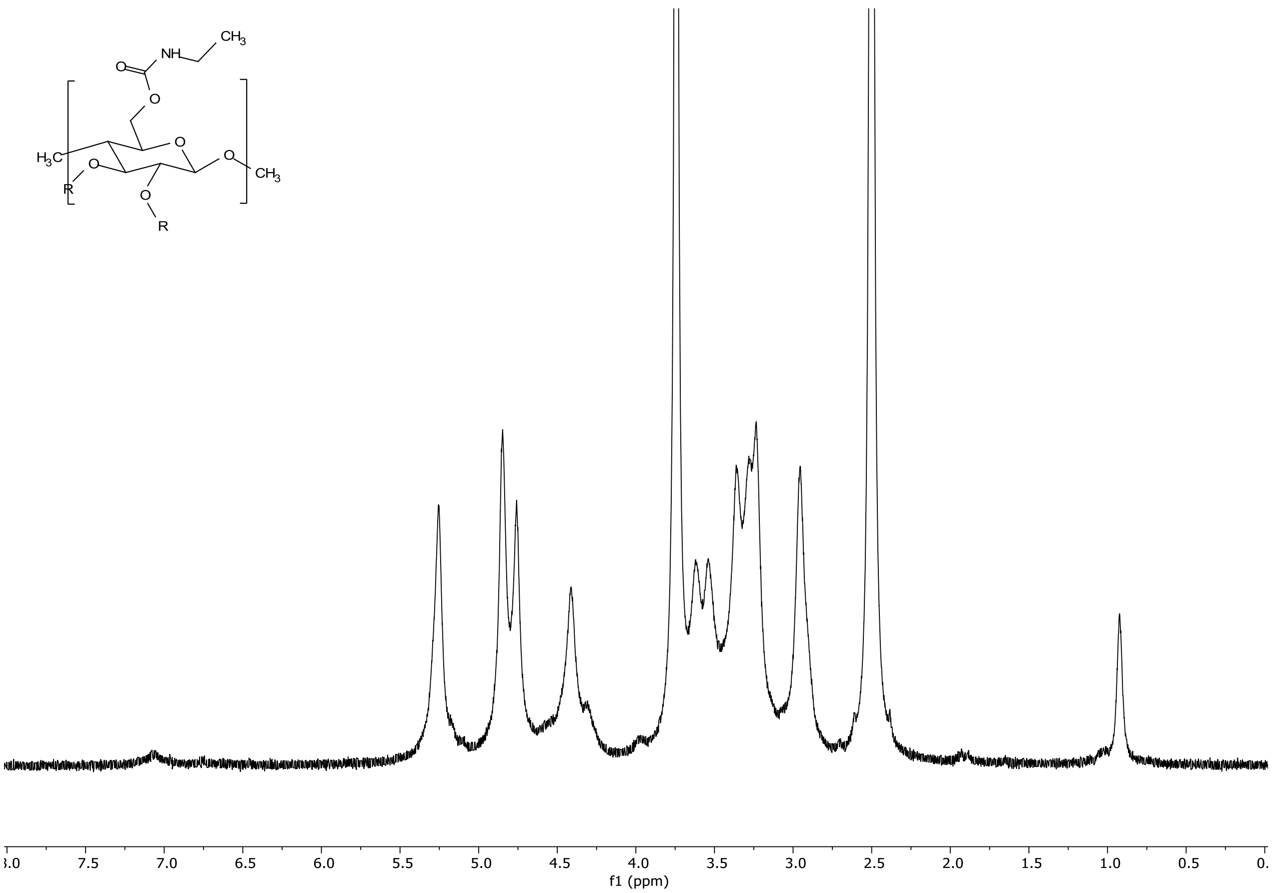

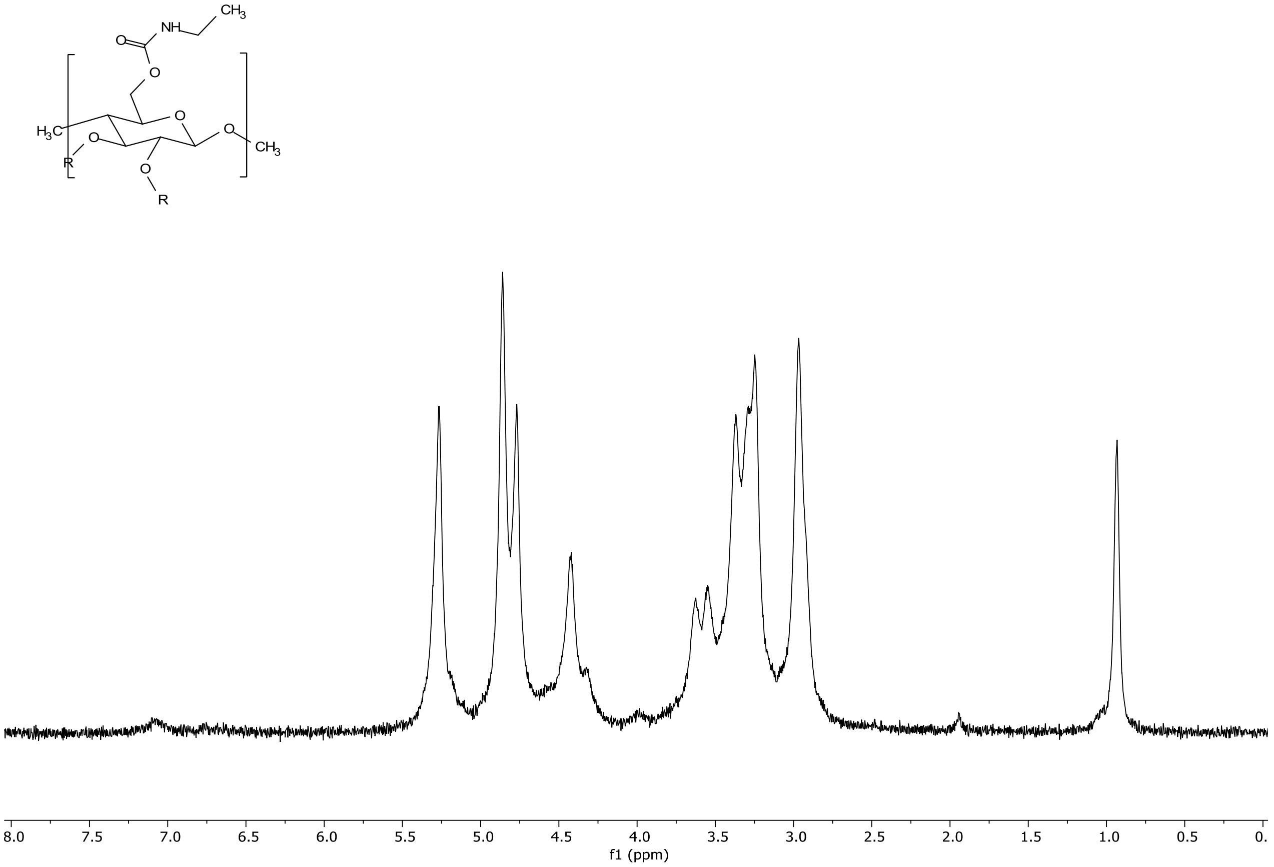

^{1}H and diffusion-edited ^{1}H spectra of **Cellulose *N*-ethyl carbamate DS 0.22** in DMSO-d_6/LiCl solution. (R: H or $-CONHCH_2CH_3$)

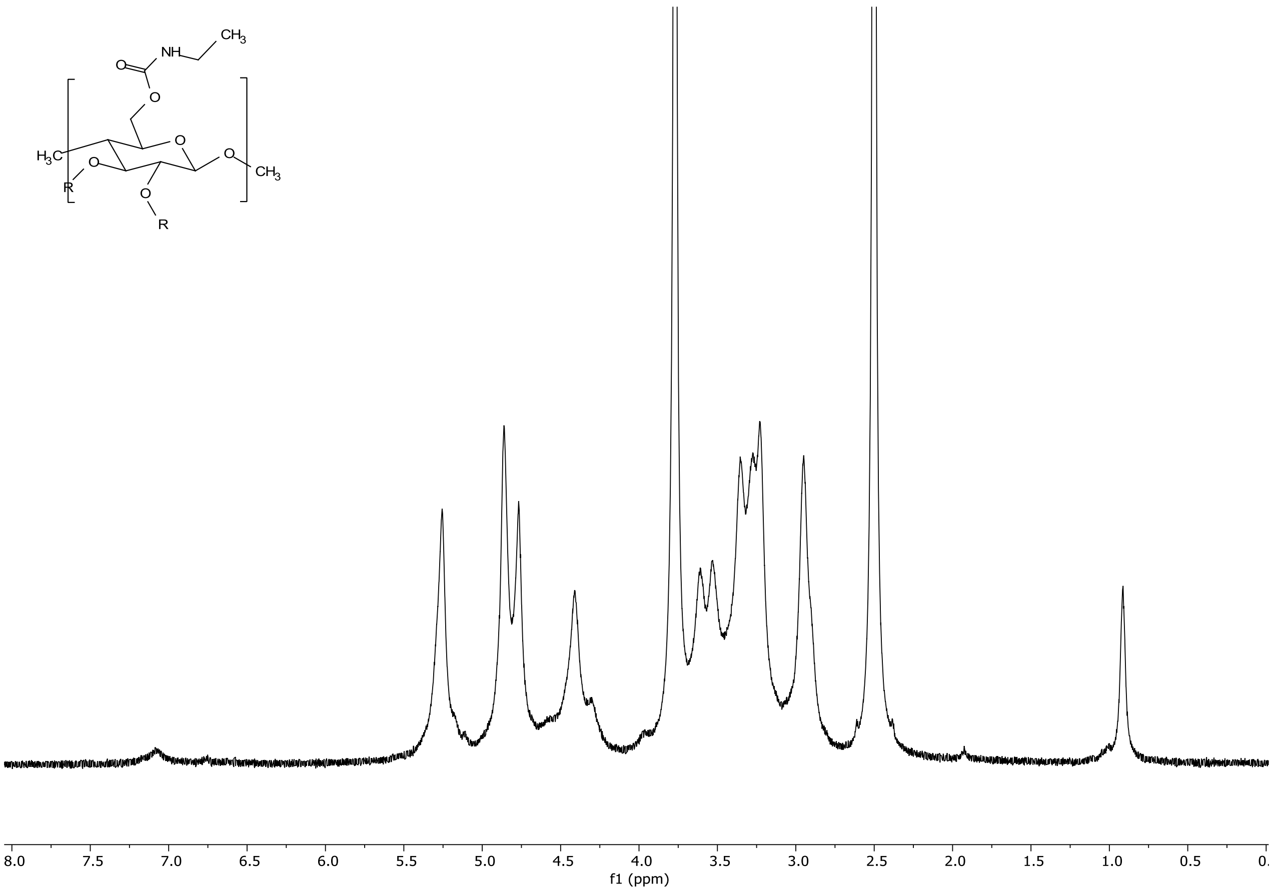

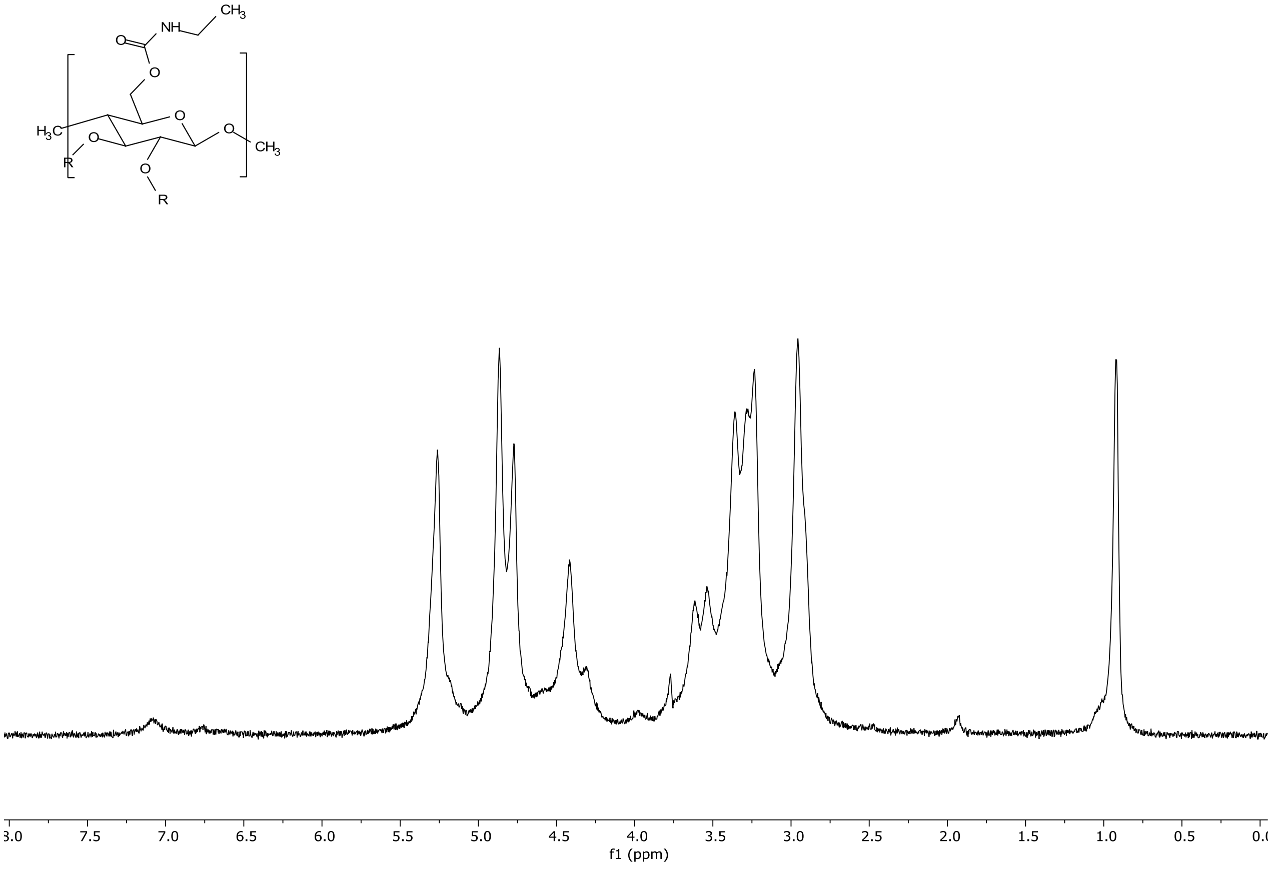

^{1}H and diffusion-edited ^{1}H spectra of **Cellulose *N*-propyl carbamate DS 0.10** in DMSO-d_6/LiCl solution. (R: H or -$CONHCH_2CH_2CH_3$)

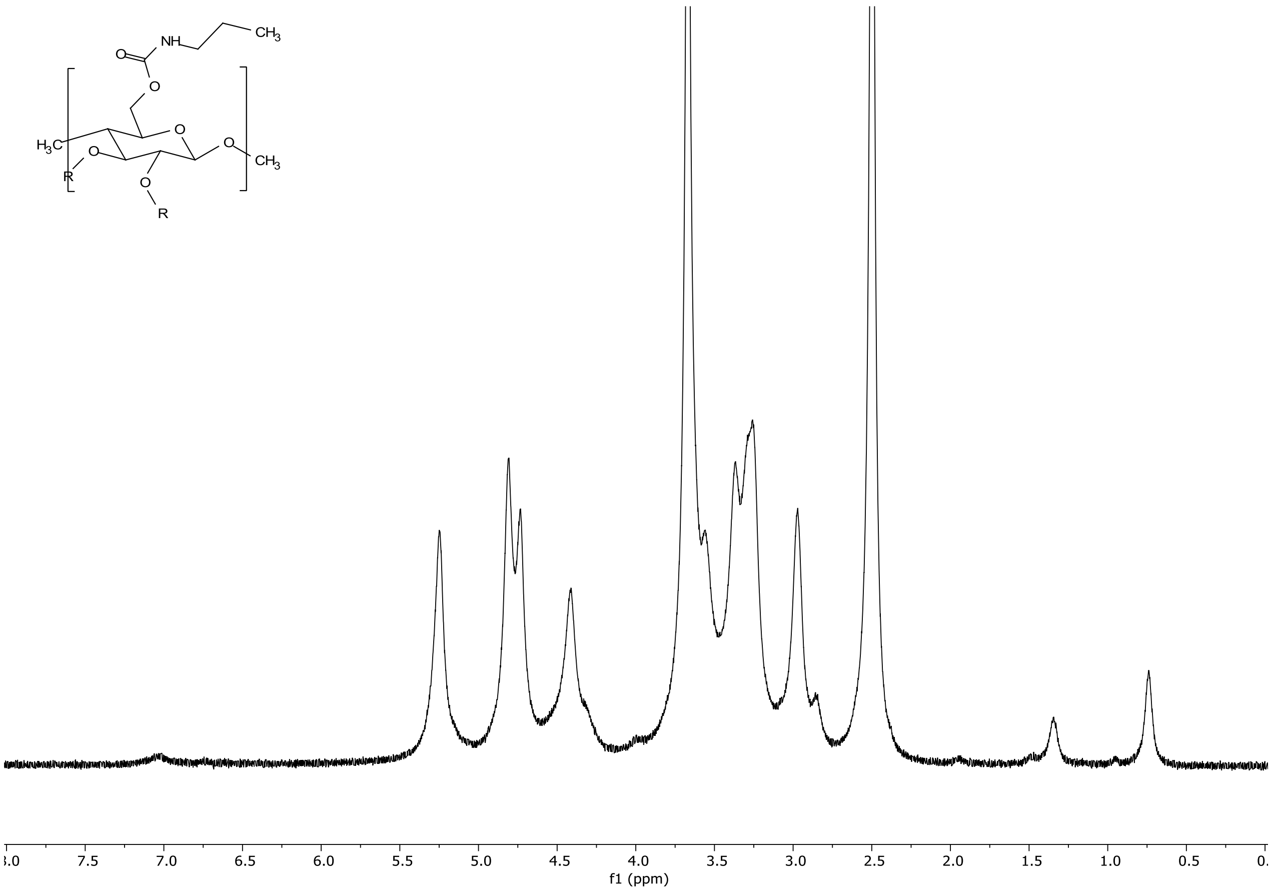

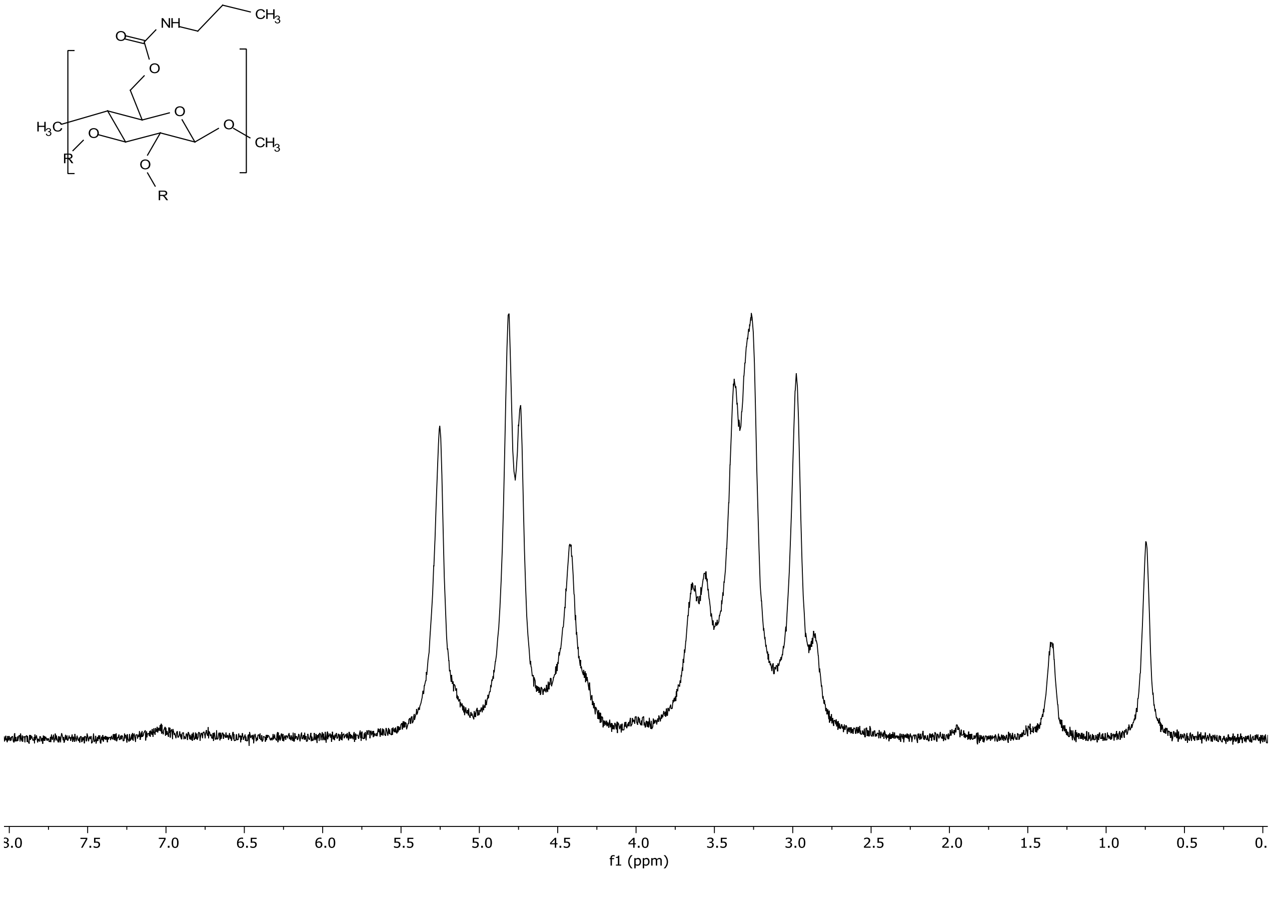

^{1}H and diffusion-edited ^{1}H spectra of **Cellulose *N*-propyl carbamate DS 0.14** in DMSO-d_6/LiCl solution. (R: H or $-CONHCH_2CH_2CH_3$)

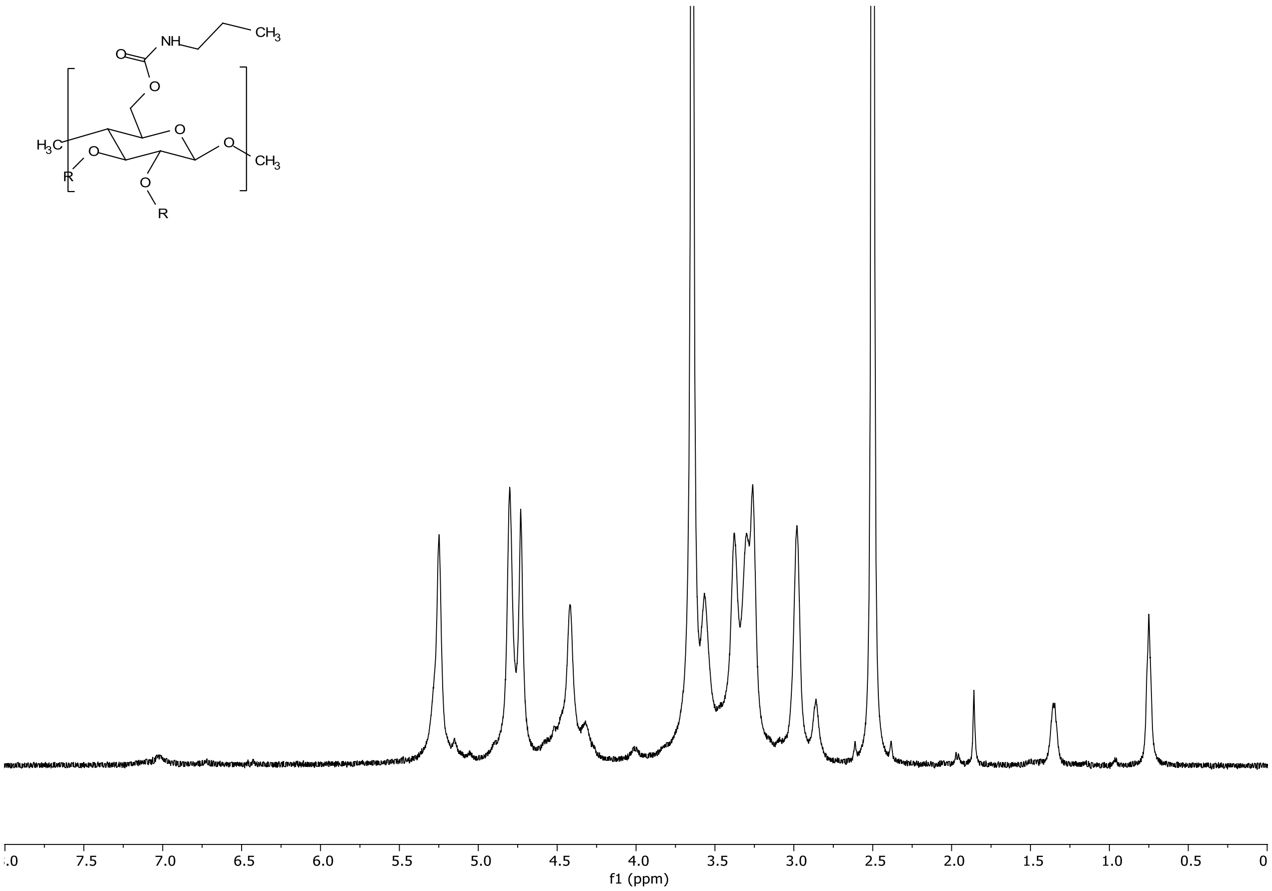

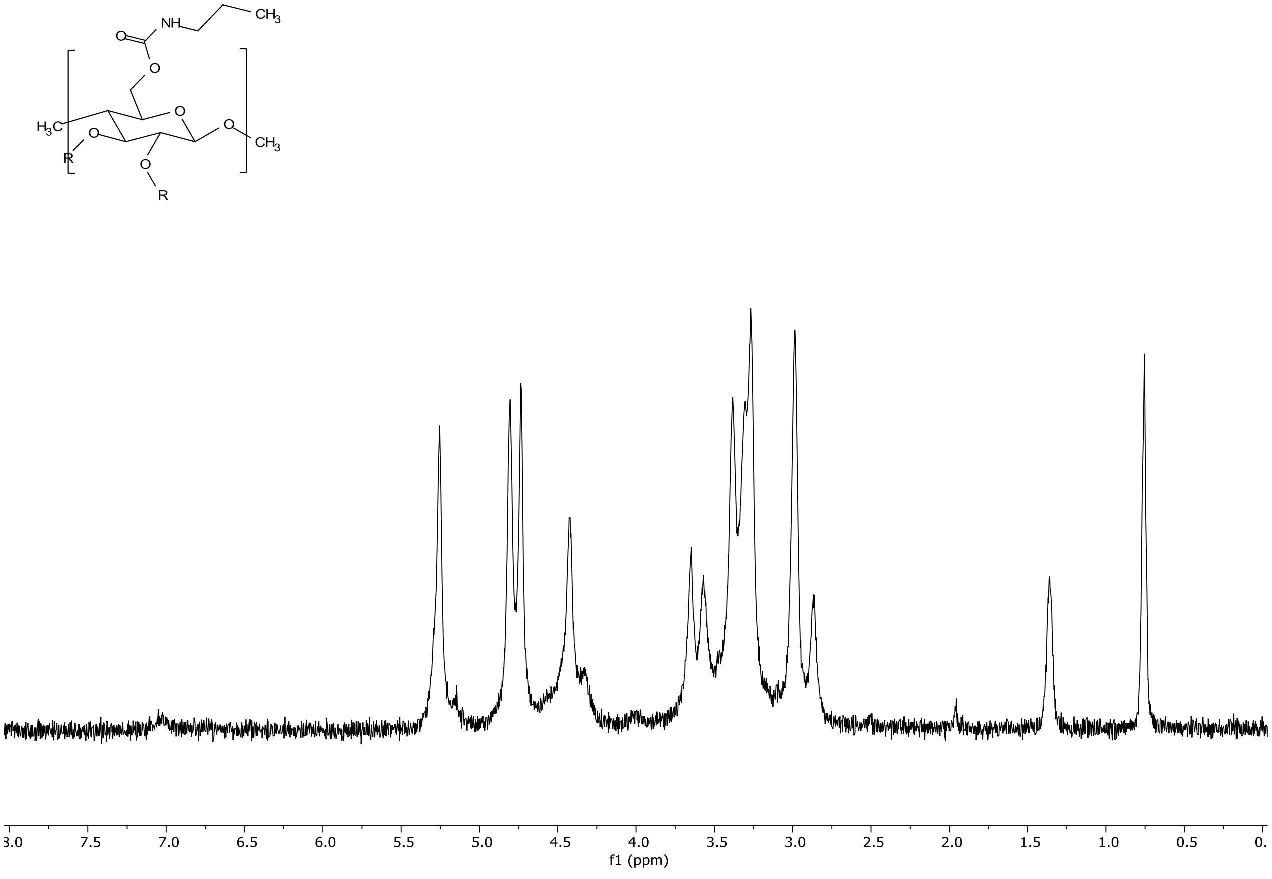

^{1}H and diffusion-edited ^{1}H spectra of **Cellulose *N*-propyl carbamate DS 0.22** in DMSO-d_6/LiCl solution. (R: H or -$CONHCH_2CH_2CH_3$)

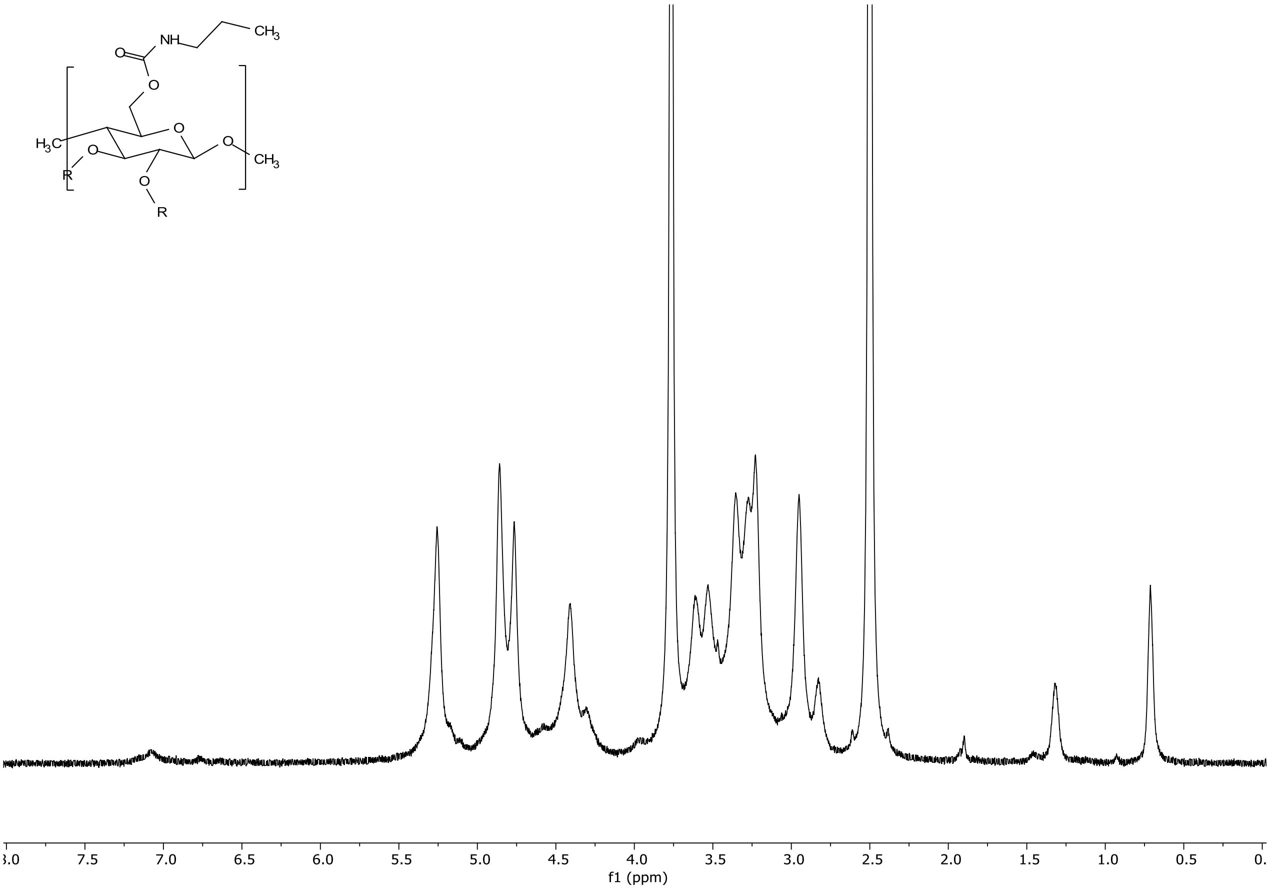

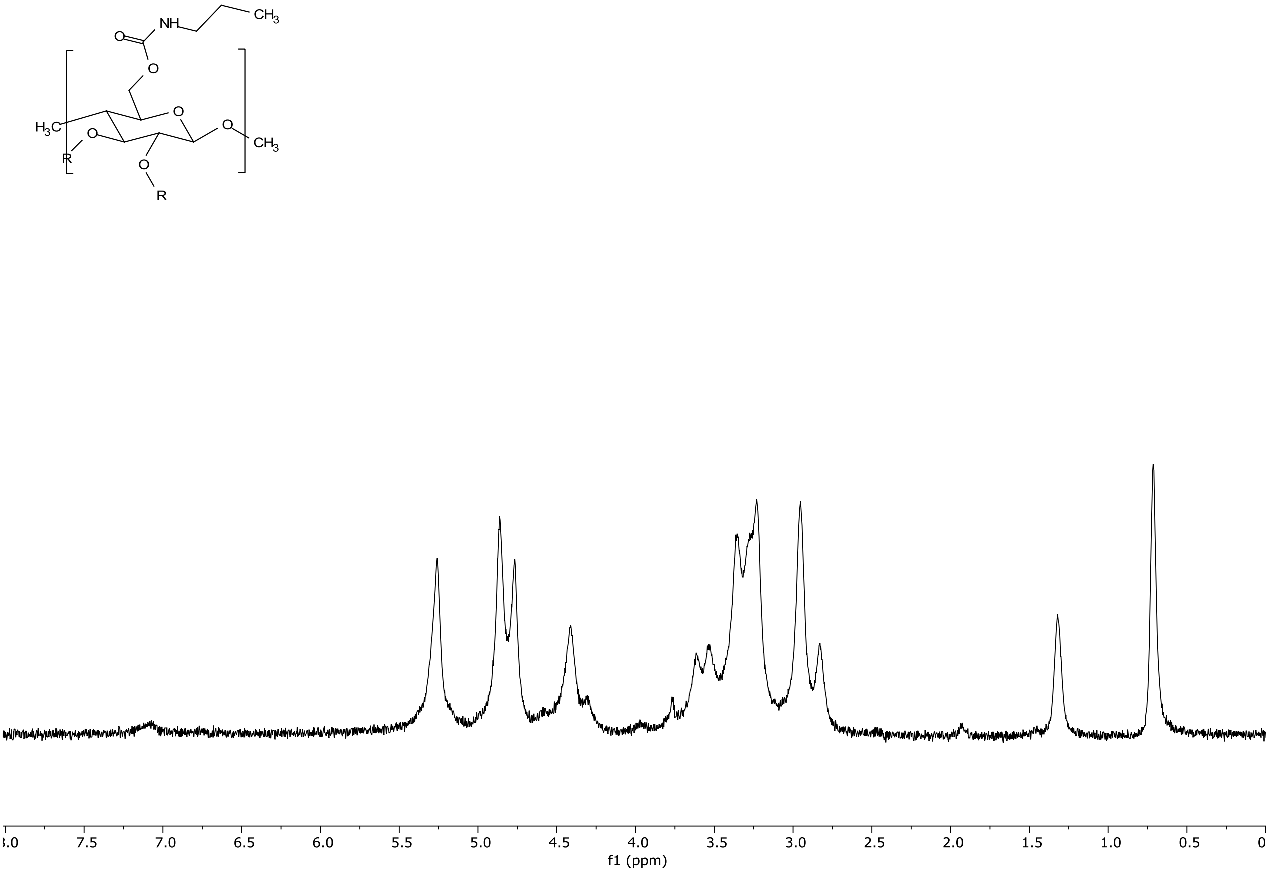

^{1}H and diffusion-edited ^{1}H spectra of **Cellulose *N*-butyl carbamate DS 0.13** in DMSO-d_6/LiCl solution. (R: H or -$CONHCH_2CH_2CH_2CH_3$)

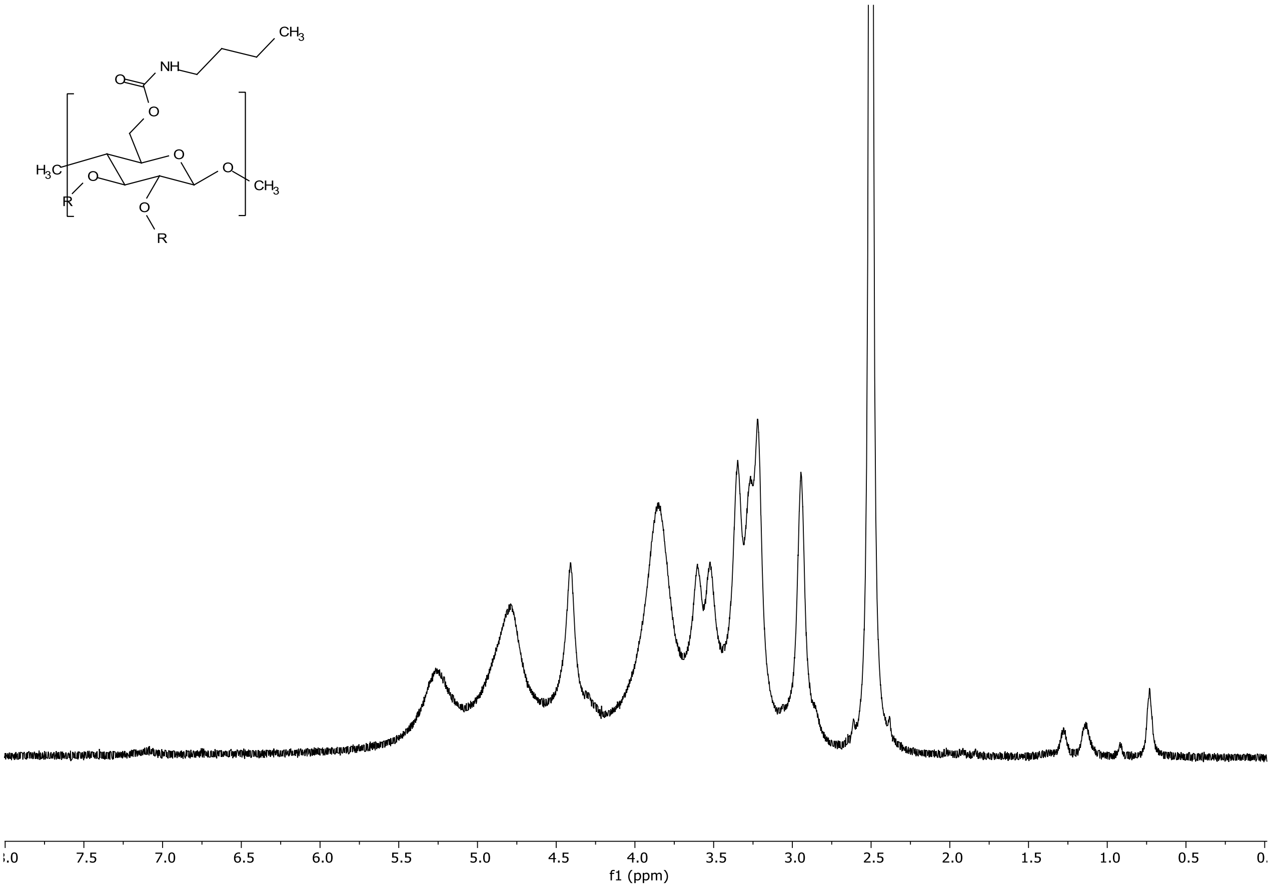

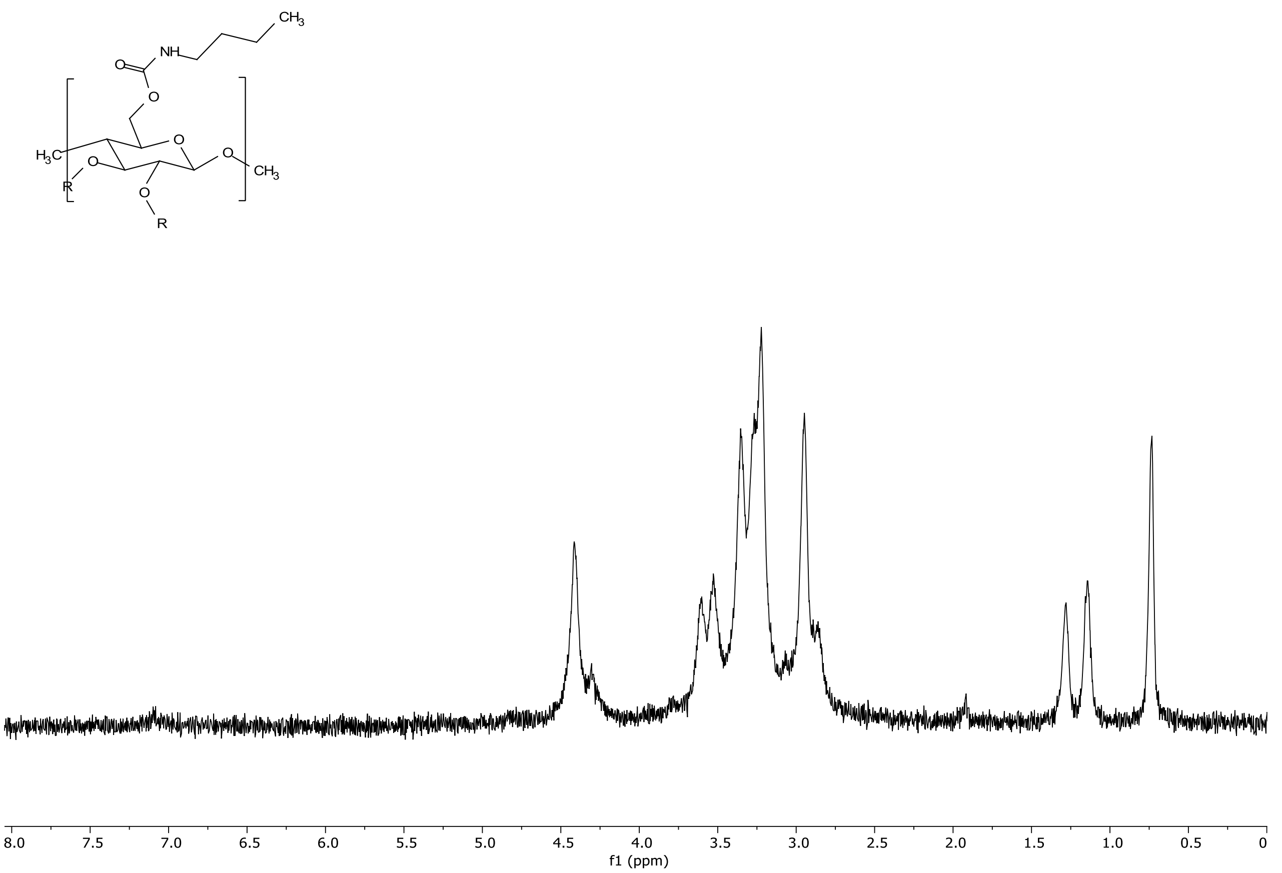

^1H and diffusion-edited ^1H spectra of **Cellulose *N*-butyl carbamate DS 0.17** in DMSO-d_6/LiCl solution. (R: H or $-CONHCH_2CH_2CH_2CH_3$)

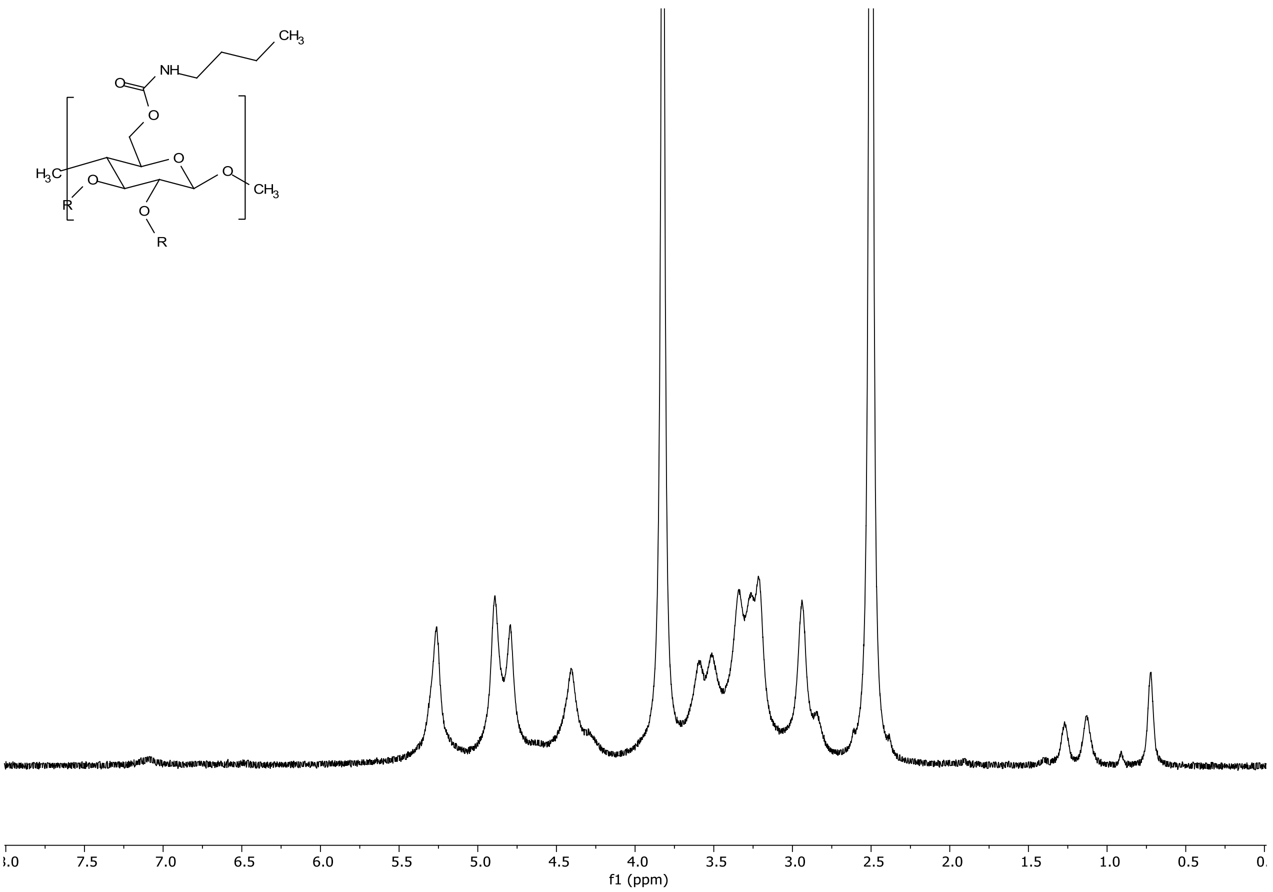

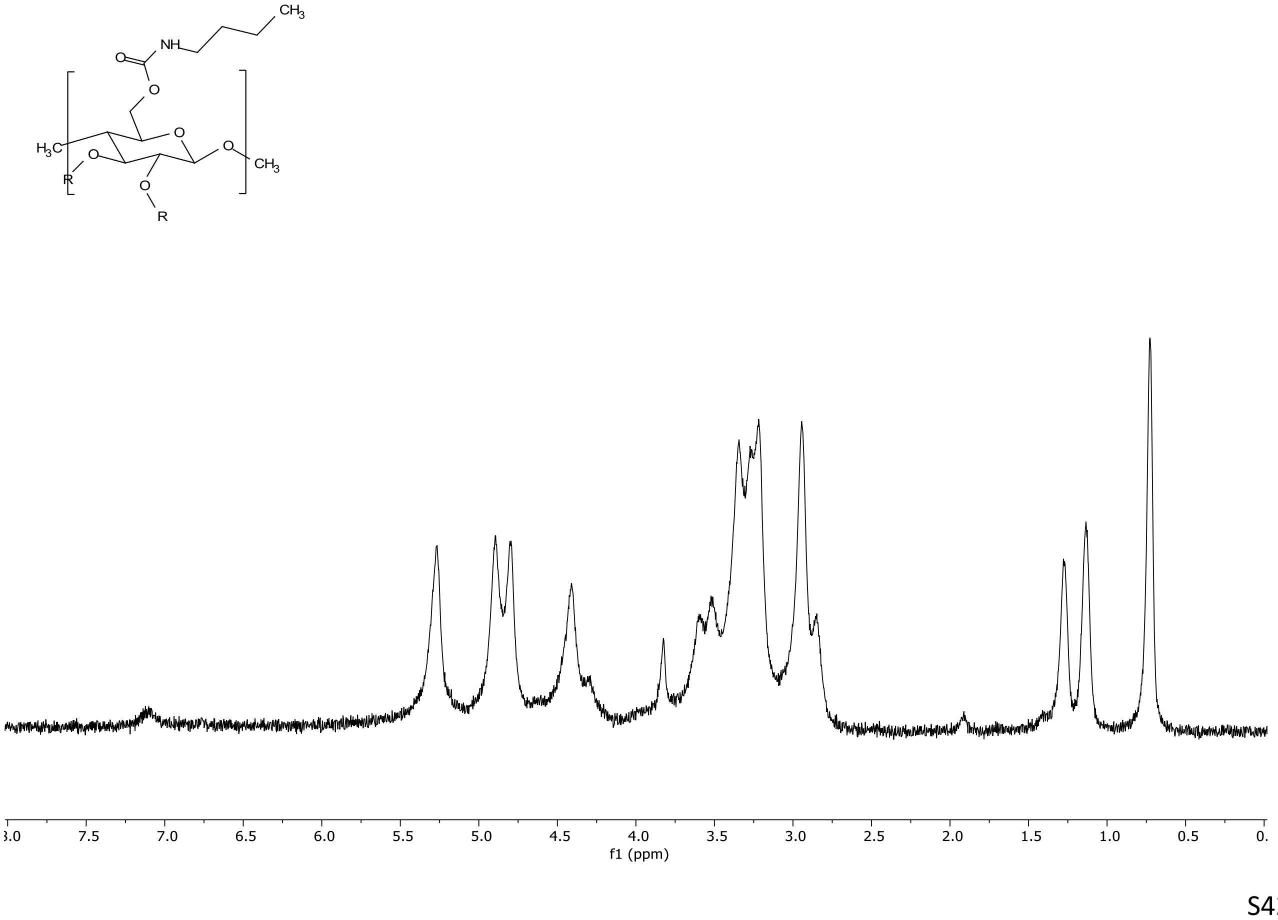

^{1}H and diffusion-edited ^{1}H spectra of **Cellulose *N*-butyl carbamate DS 0.18** in DMSO-d_6/LiCl solution. (R: H or -$CONHCH_2CH_2CH_2CH_3$)

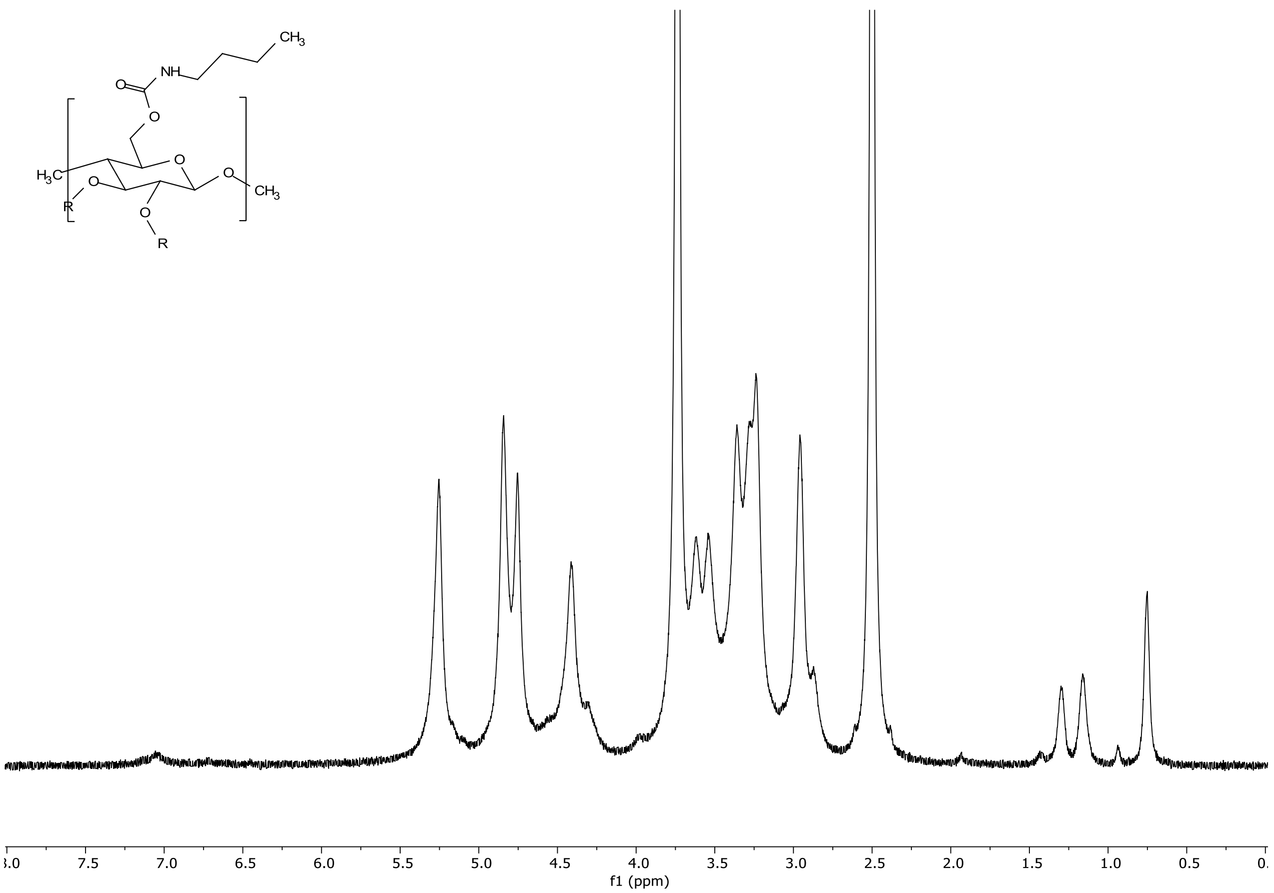

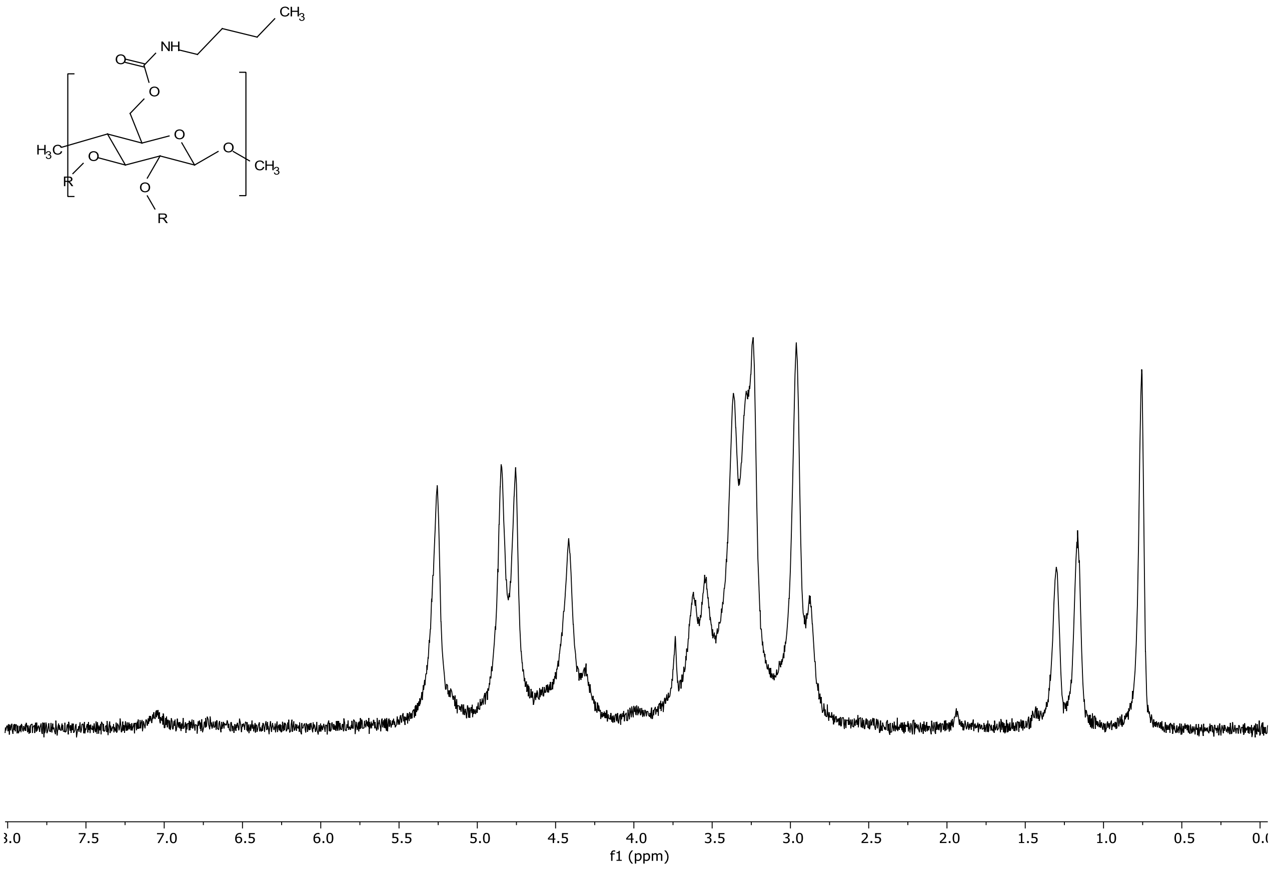

^{1}H and diffusion-edited ^{1}H spectra of **Cellulose N-(N,N,N-)trimethylpropan-1-aminium acetate carbamate DS 0.10** in DMSO-d_6/LiCl solution. (R: H or $-CONHCH_2CH_2CH_2N^{+}(CH_3)_3$ $O^{-}Ac$)

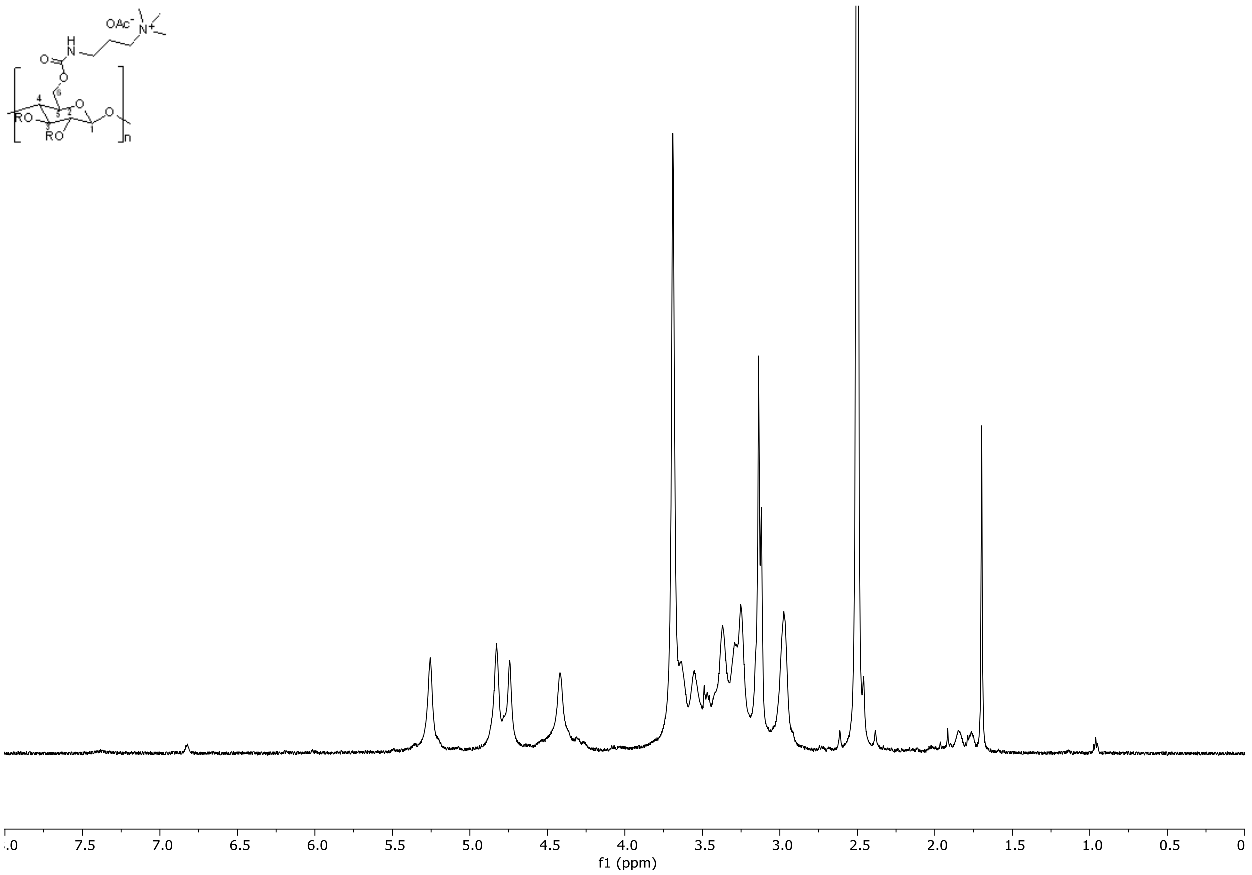

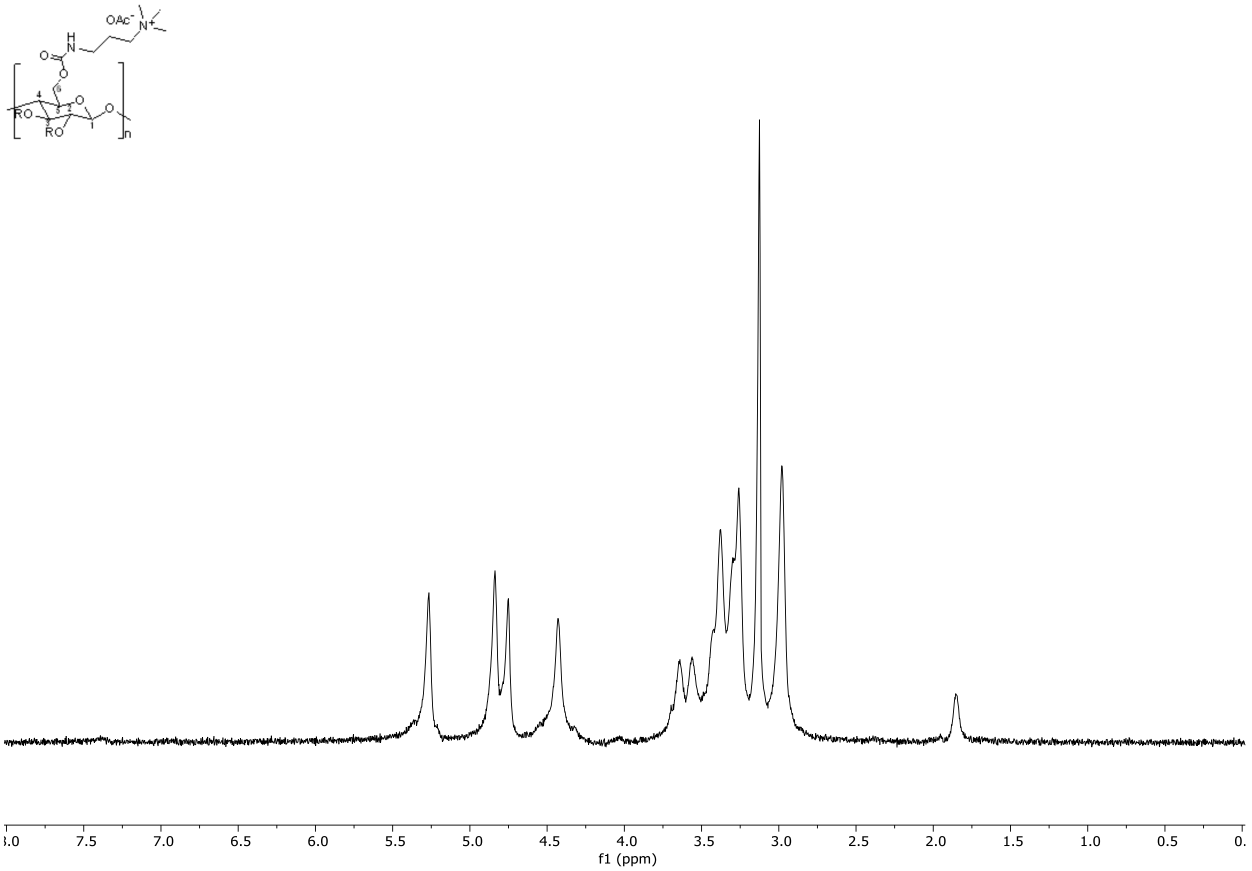

^{1}H and diffusion-edited ^{1}H spectra of **Cellulose N-(N,N,N-)trimethylpropan-1-aminium acetate carbamate DS 0.10** in DMSO-d_6/LiCl solution. (R: H or -$CONHCH_2CH_2CH_2N^+(CH_3)_3$ O^-Ac)

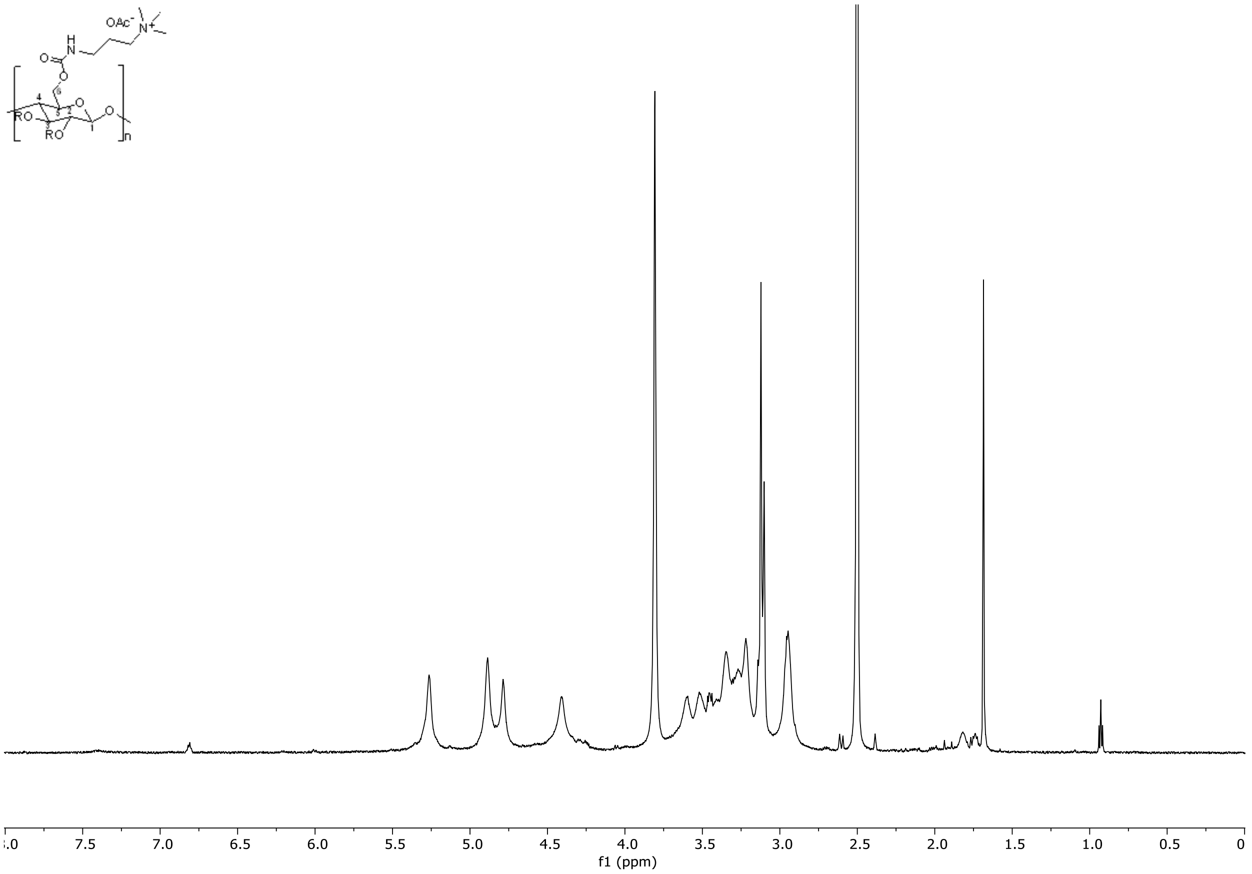

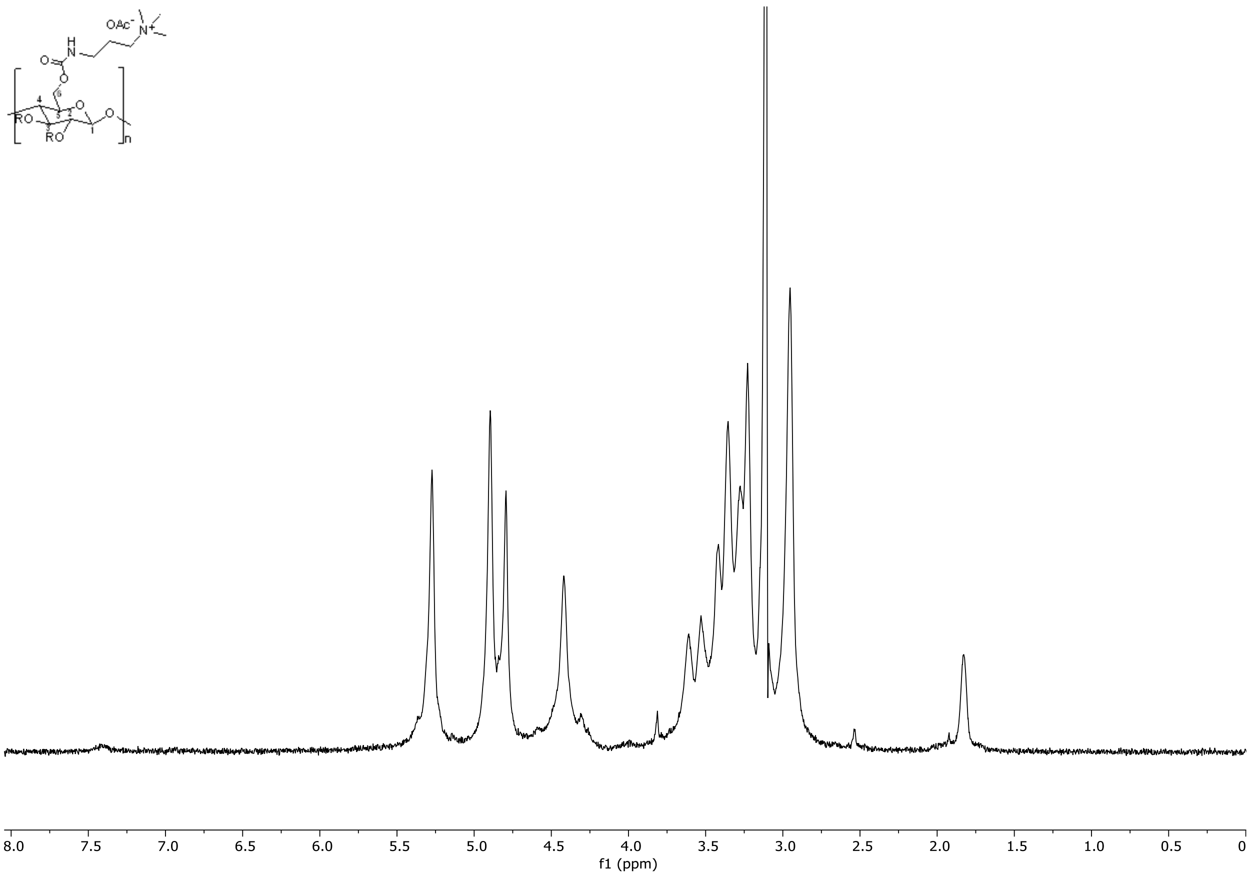

^1H and diffusion-edited ^1H spectra of **Cellulose N-(N,N,N-)trimethylpropan-1-aminium acetate carbamate DS 0.14** in DMSO-d_6/LiCl solution. (R: H or $-CONHCH_2CH_2CH_2N^+(CH_3)_3\ O^-Ac$)

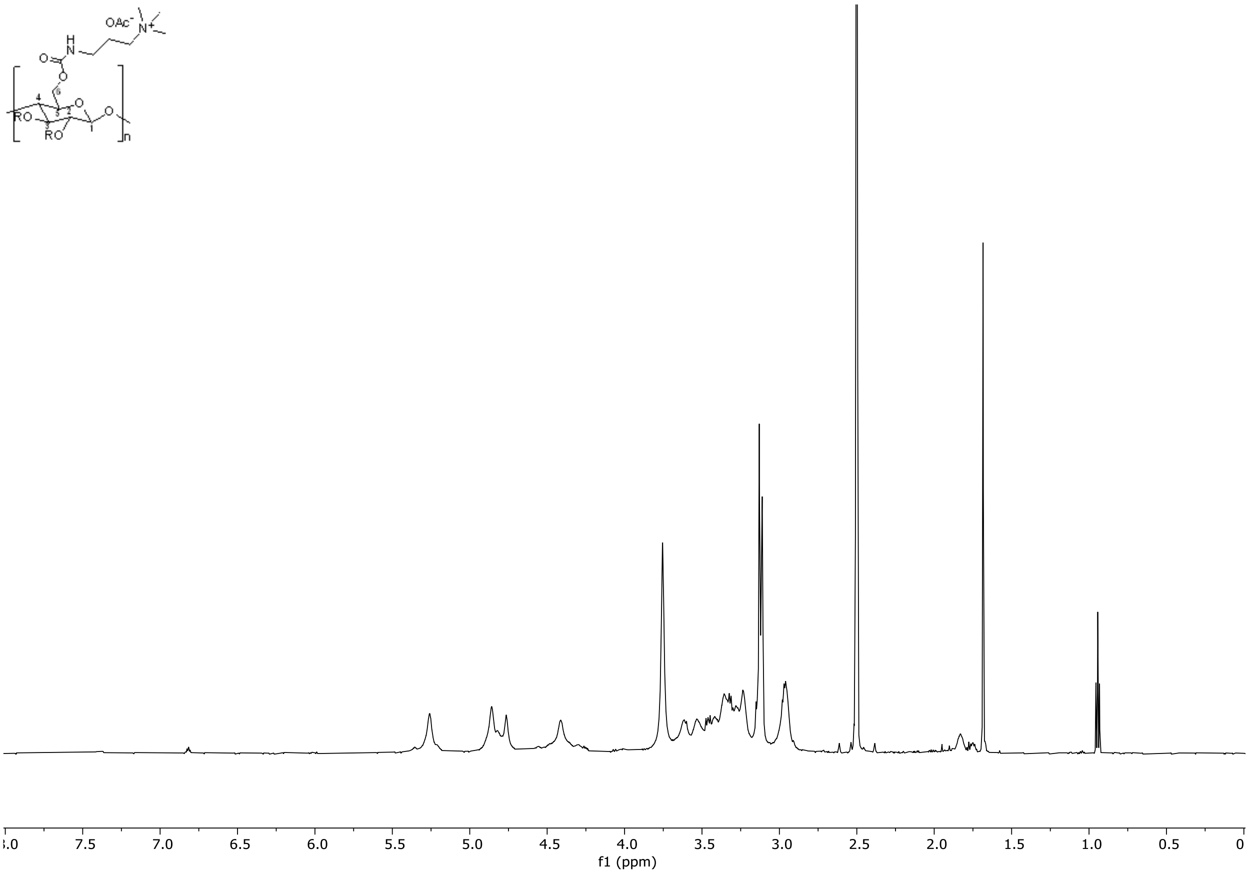

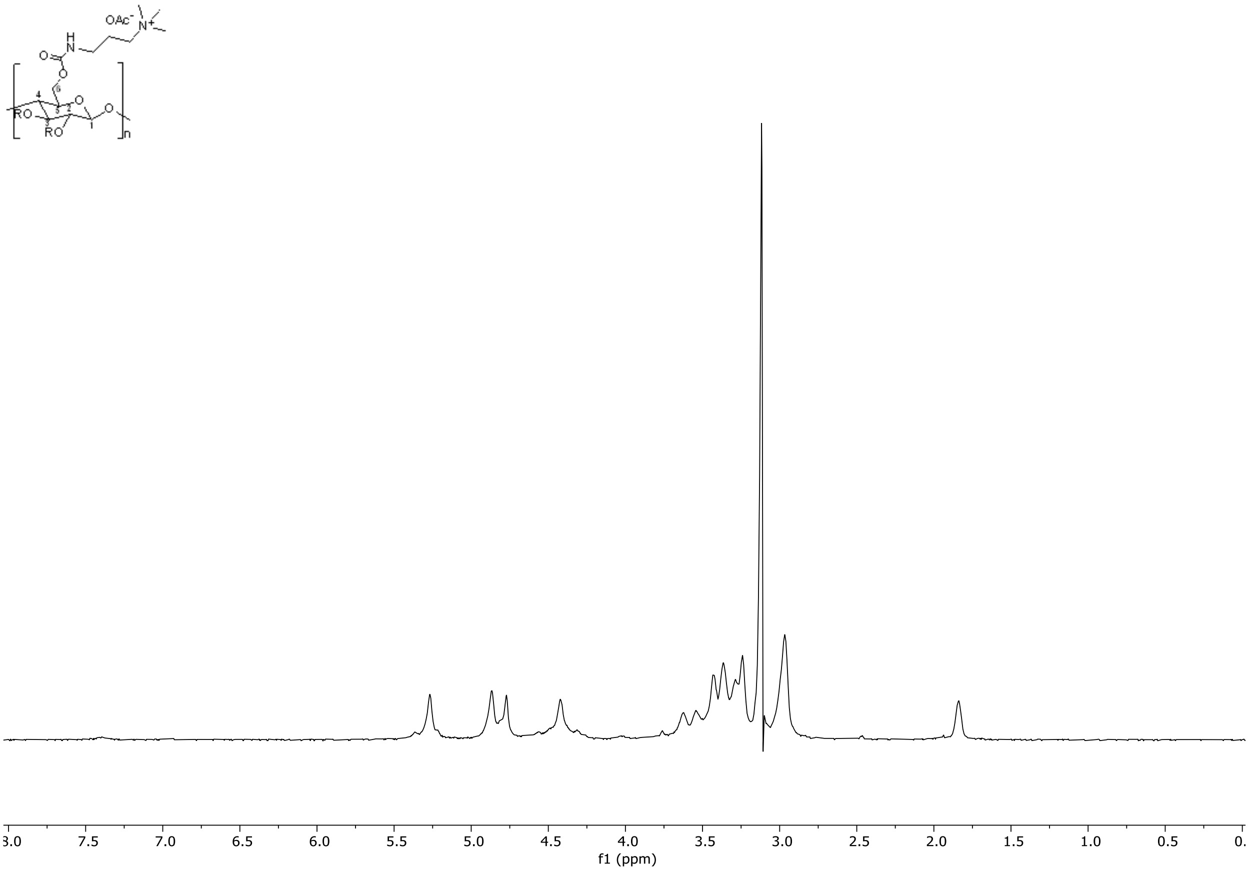

^{1}H and diffusion-edited ^{1}H spectra of **Cellulose *N-iso*-propyl carbamate DS 0.04** in DMSO-d_6/LiCl solution. (R: H or $-CONHCH(CH_3)_2$)

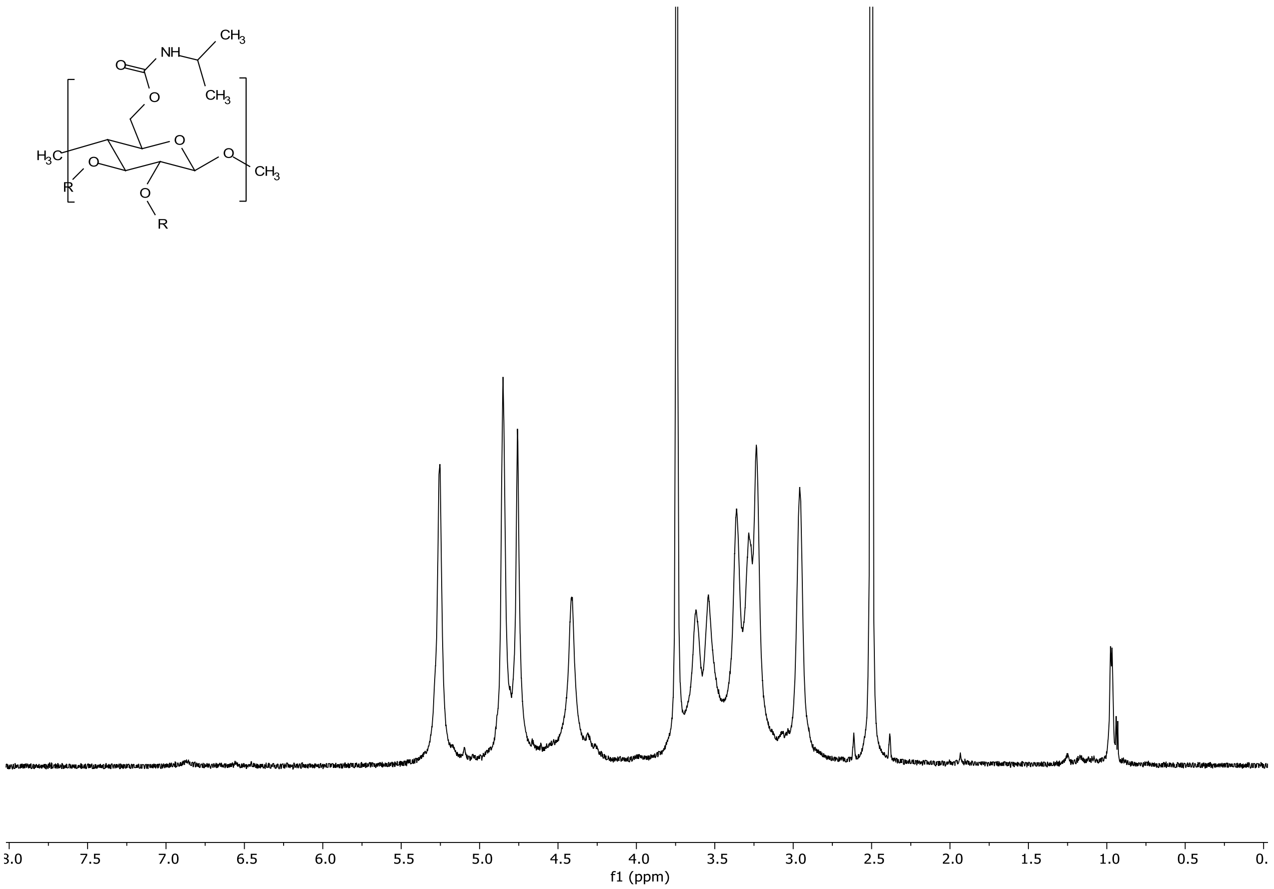

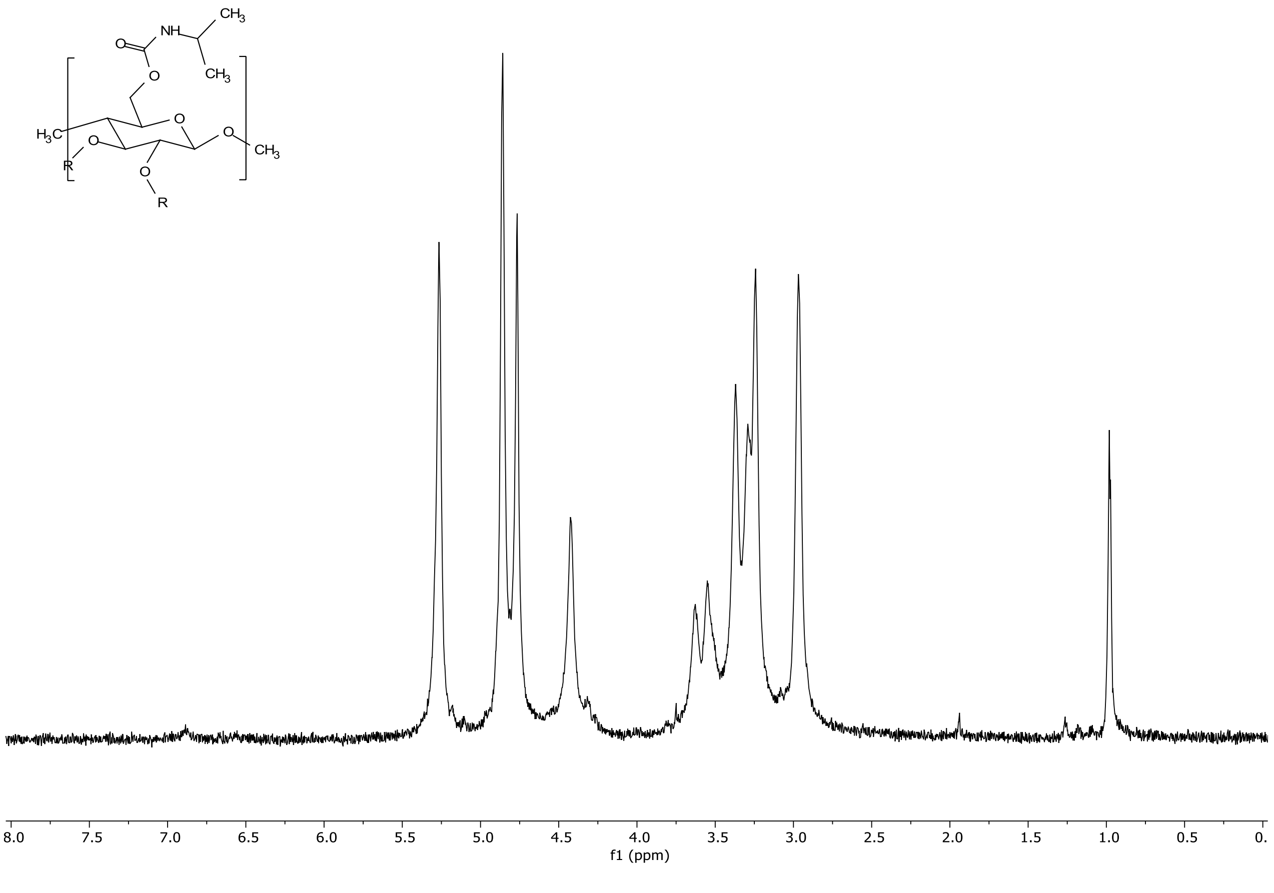

^{1}H and diffusion-edited ^{1}H spectra of **Cellulose *N-iso*-propyl carbamate DS 0.05** in DMSO-d_6/LiCl solution. (R: H or -CONHCH(CH_3)$_2$)

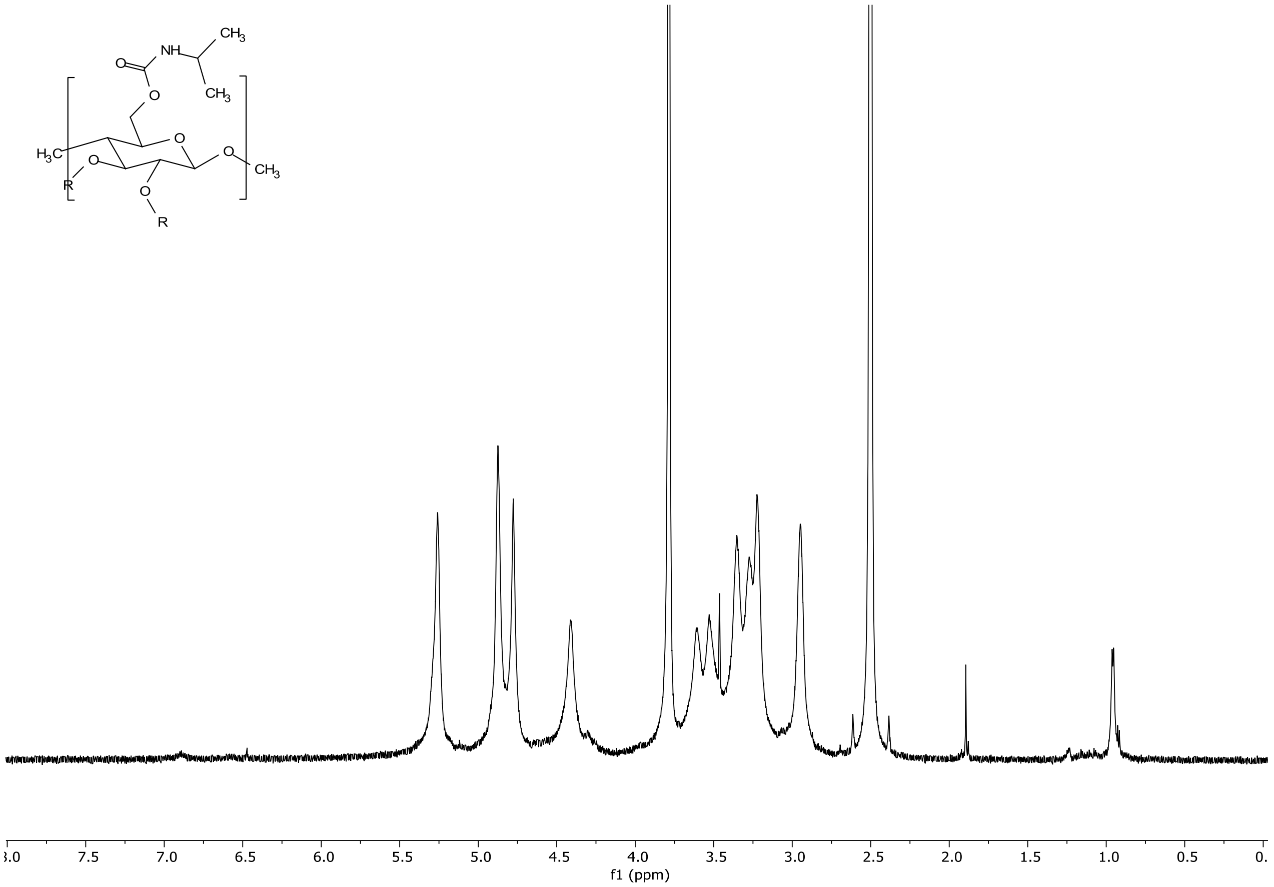

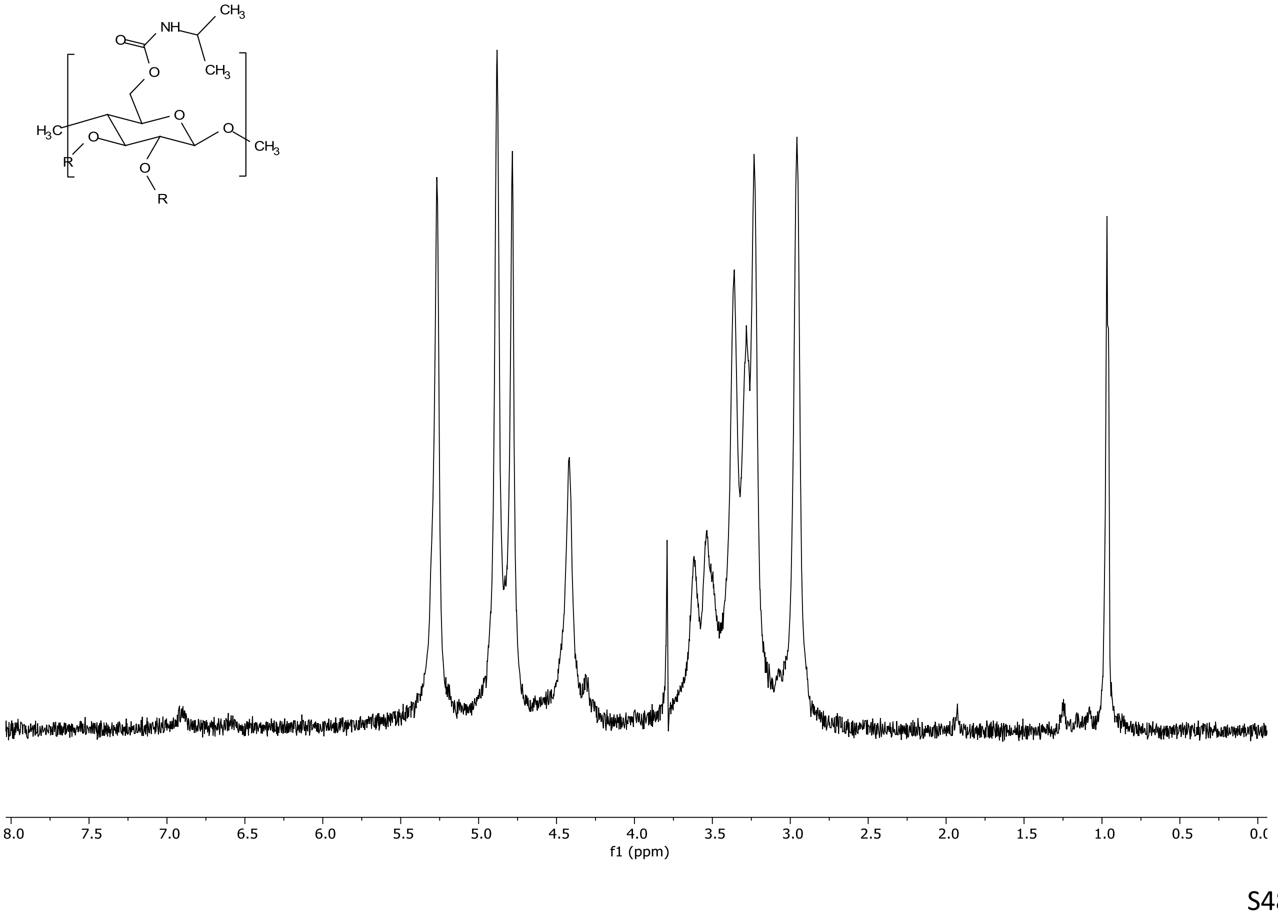

^{1}H and diffusion-edited ^{1}H spectra of **Cellulose *N-iso*-propyl carbamate DS 0.08** in DMSO-d_6/LiCl solution. (R: H or -CONHCH(CH_3)$_2$)

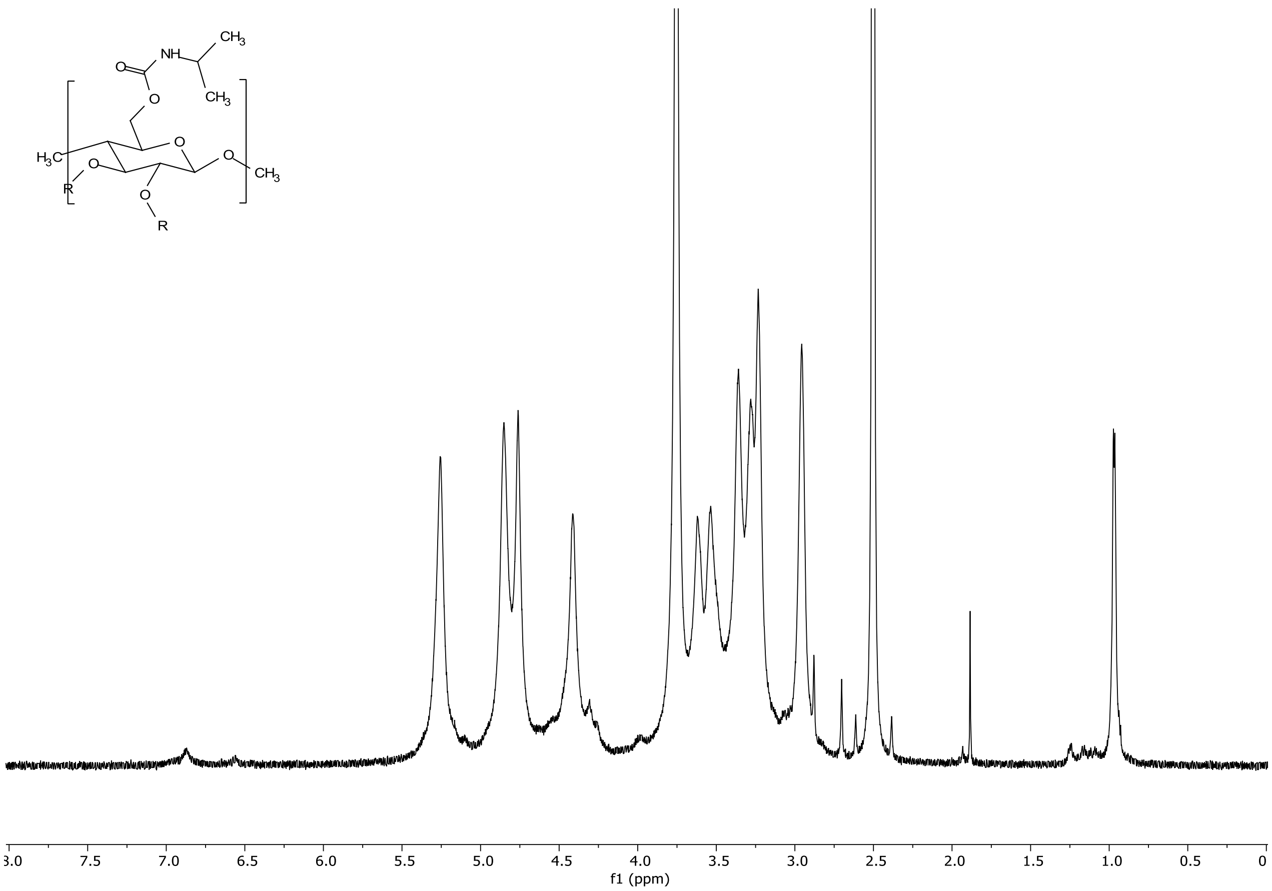

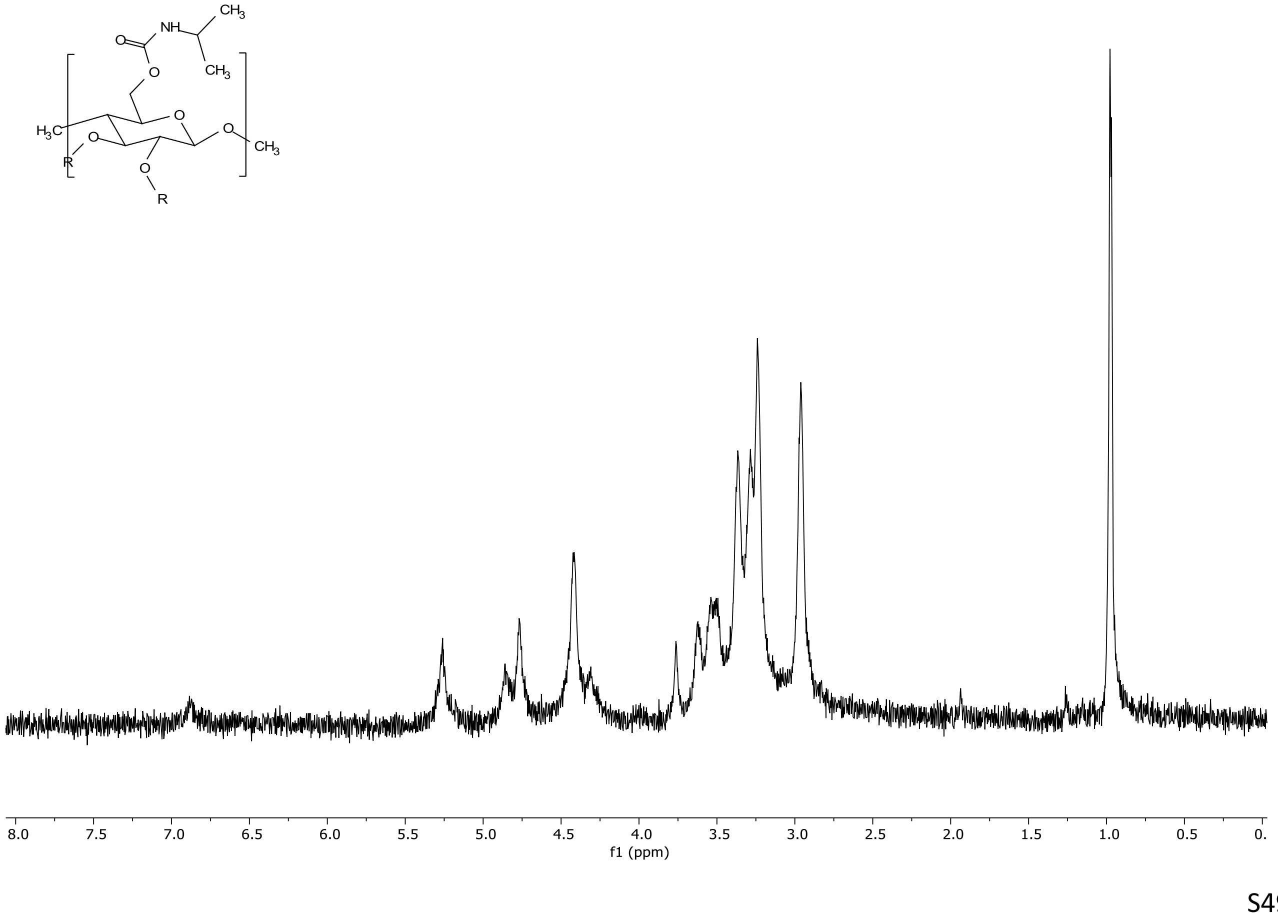

^{1}H and diffusion-edited ^{1}H spectra of **Cellulose *N-tert*-butyl carbamate DS 0.04** in DMSO-d_6/LiCl solution. (R: H or -CONHC(CH_3)$_3$)

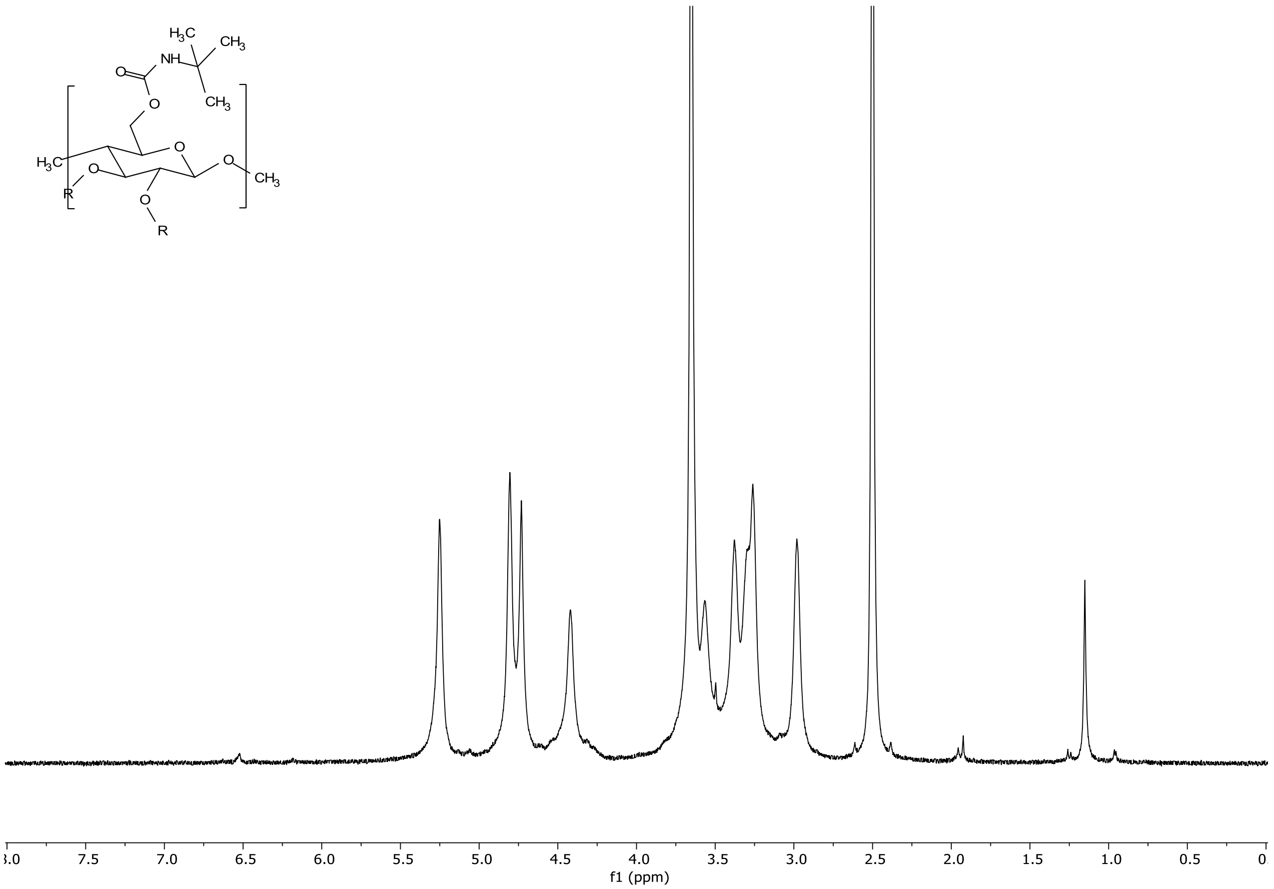

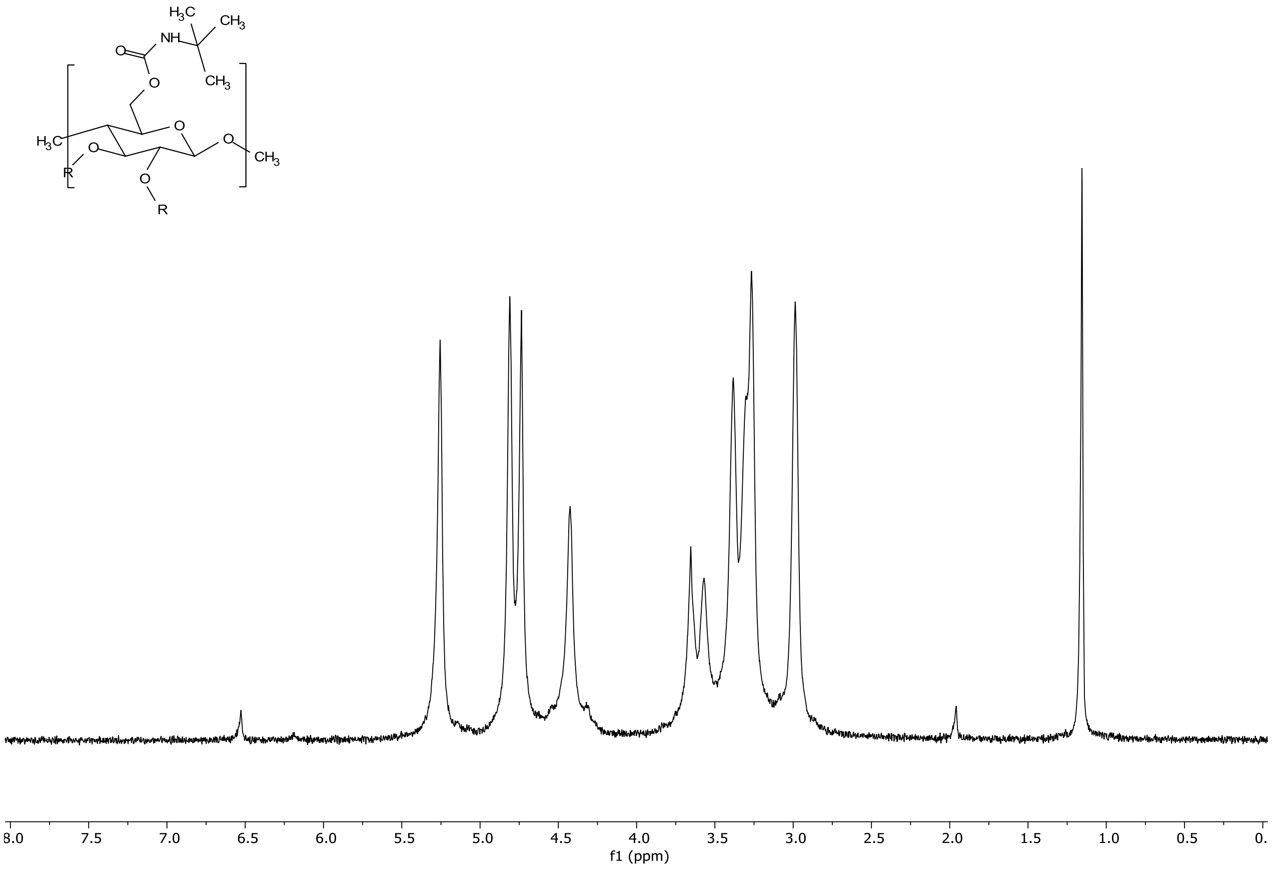

^{1}H and diffusion-edited ^{1}H spectra of **Cellulose *N-tert*-butyl carbamate DS 0.05** in DMSO-d_6/LiCl solution. (R: H or -CONHC(CH_3)$_3$)

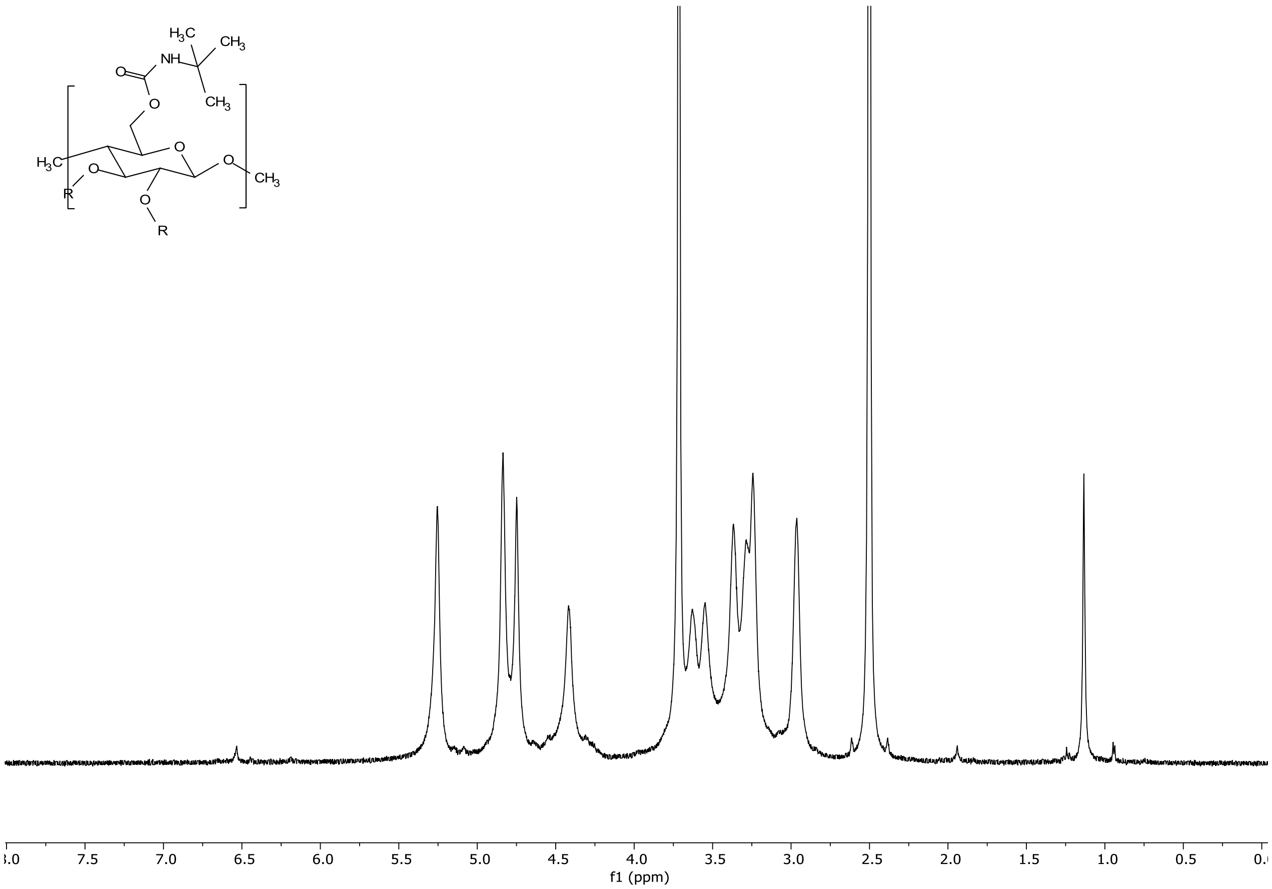

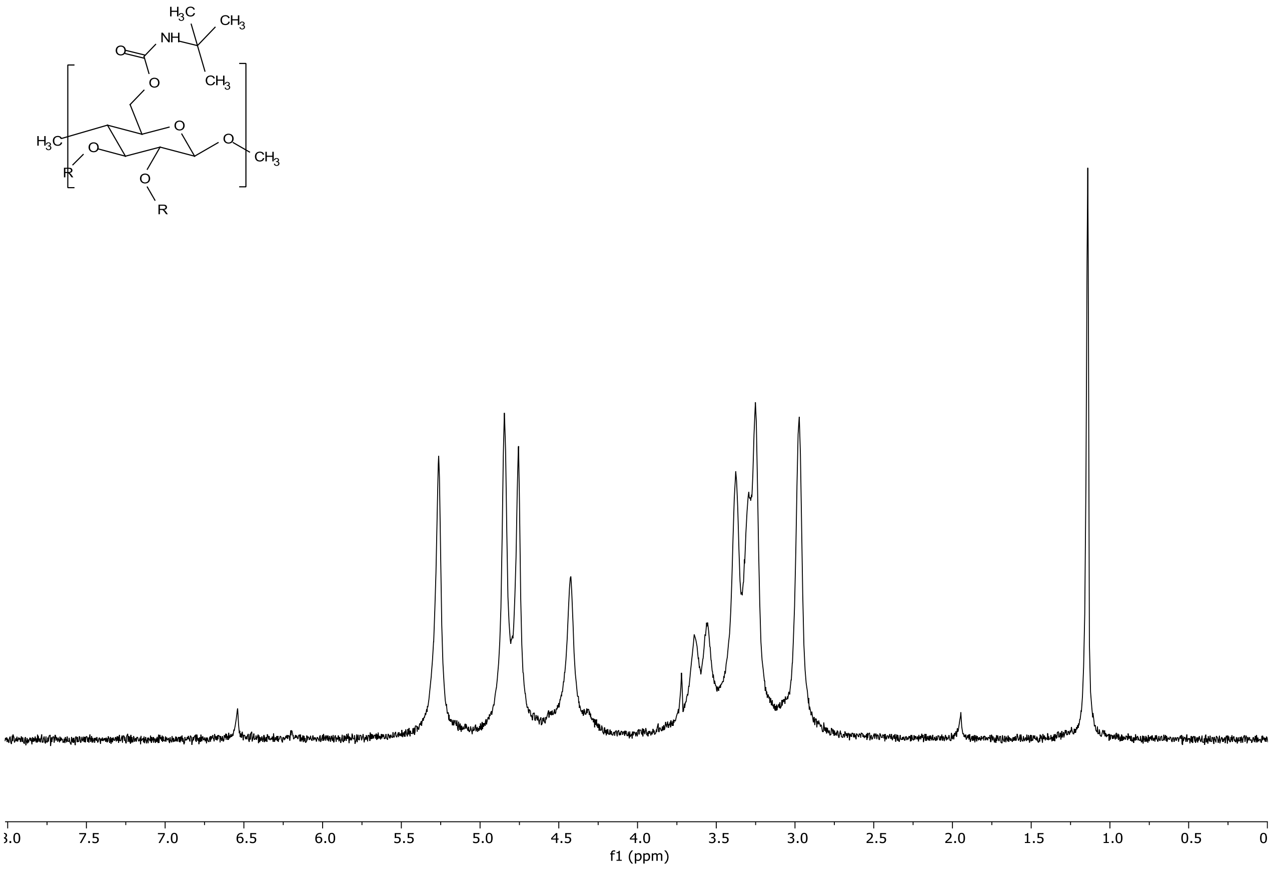

^{1}H and diffusion-edited ^{1}H spectra of **Cellulose *N-tert*-butyl carbamate DS 0.06** in DMSO-d_6/LiCl solution. (R: H or -CONHC(CH_3)$_3$)

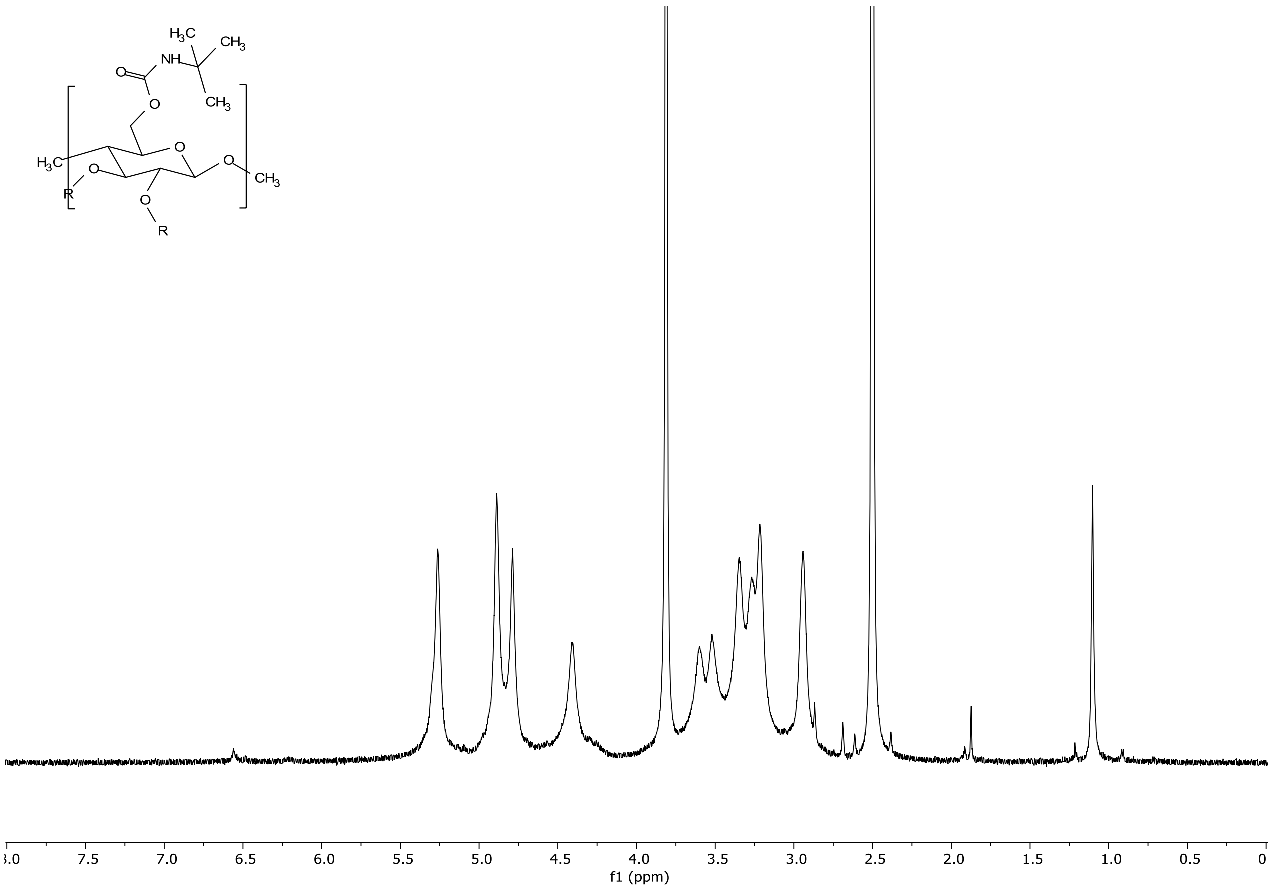

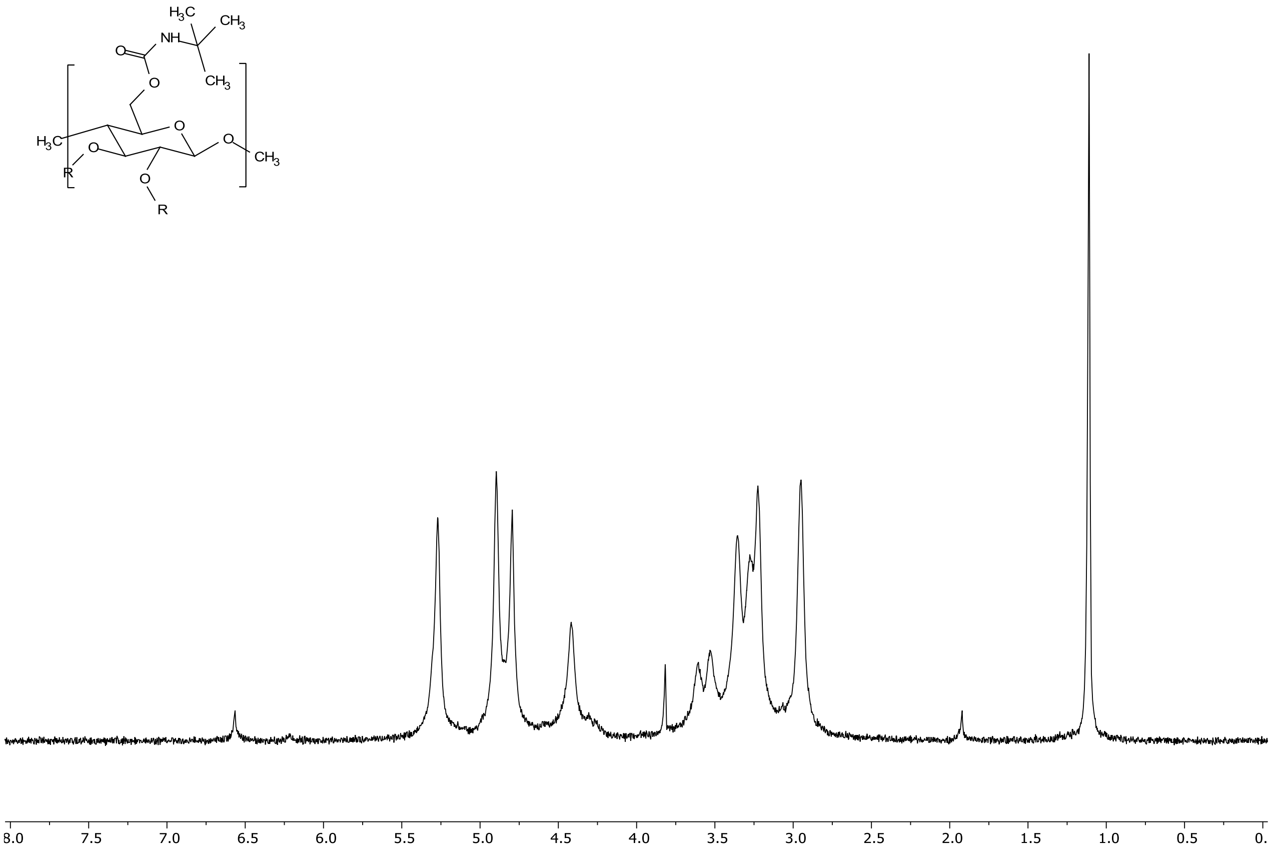

^{1}H and diffusion-edited ^{1}H spectra of **Cellulose *N*-phenyl carbamate DS 0.14** in DMSO-d_6/LiCl solution. (R: H or -CONHPh)

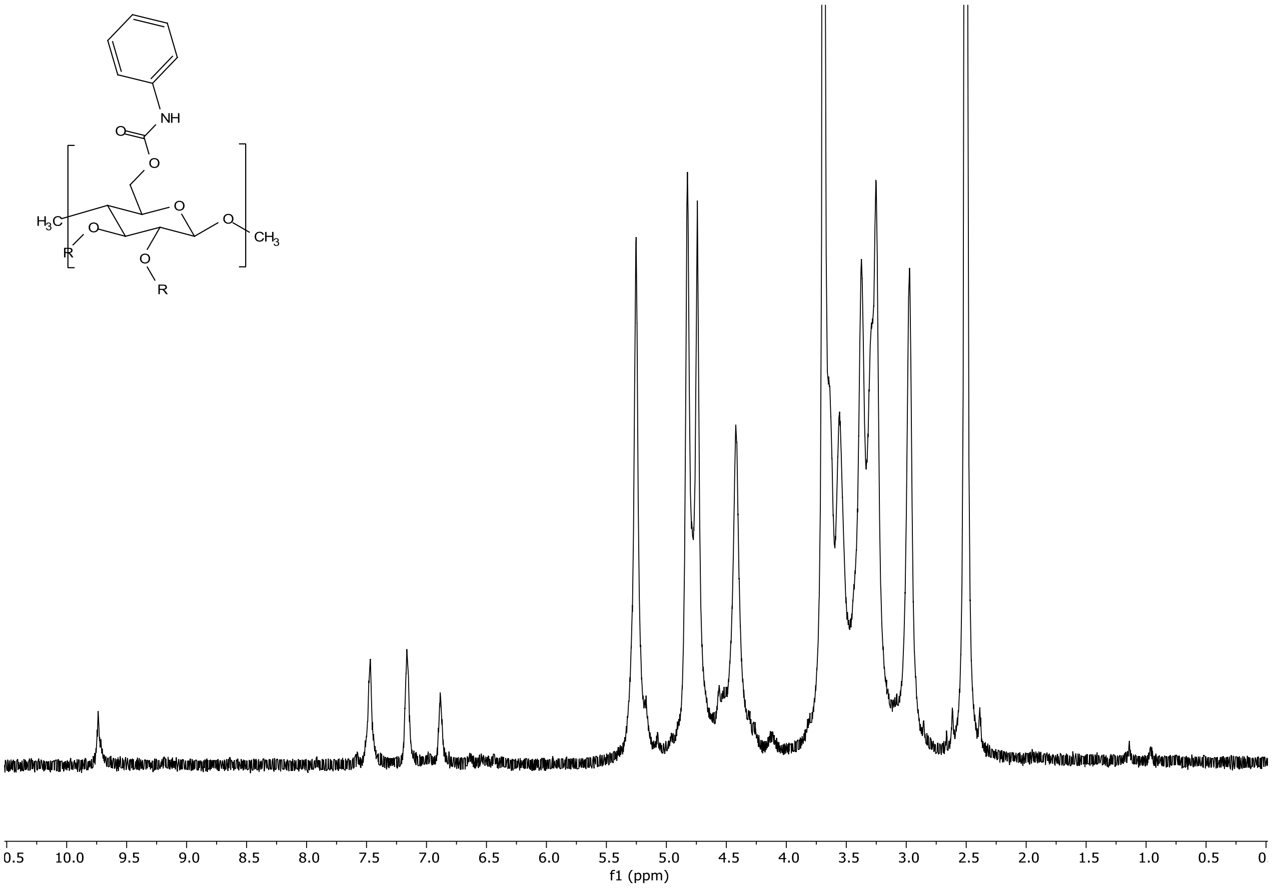

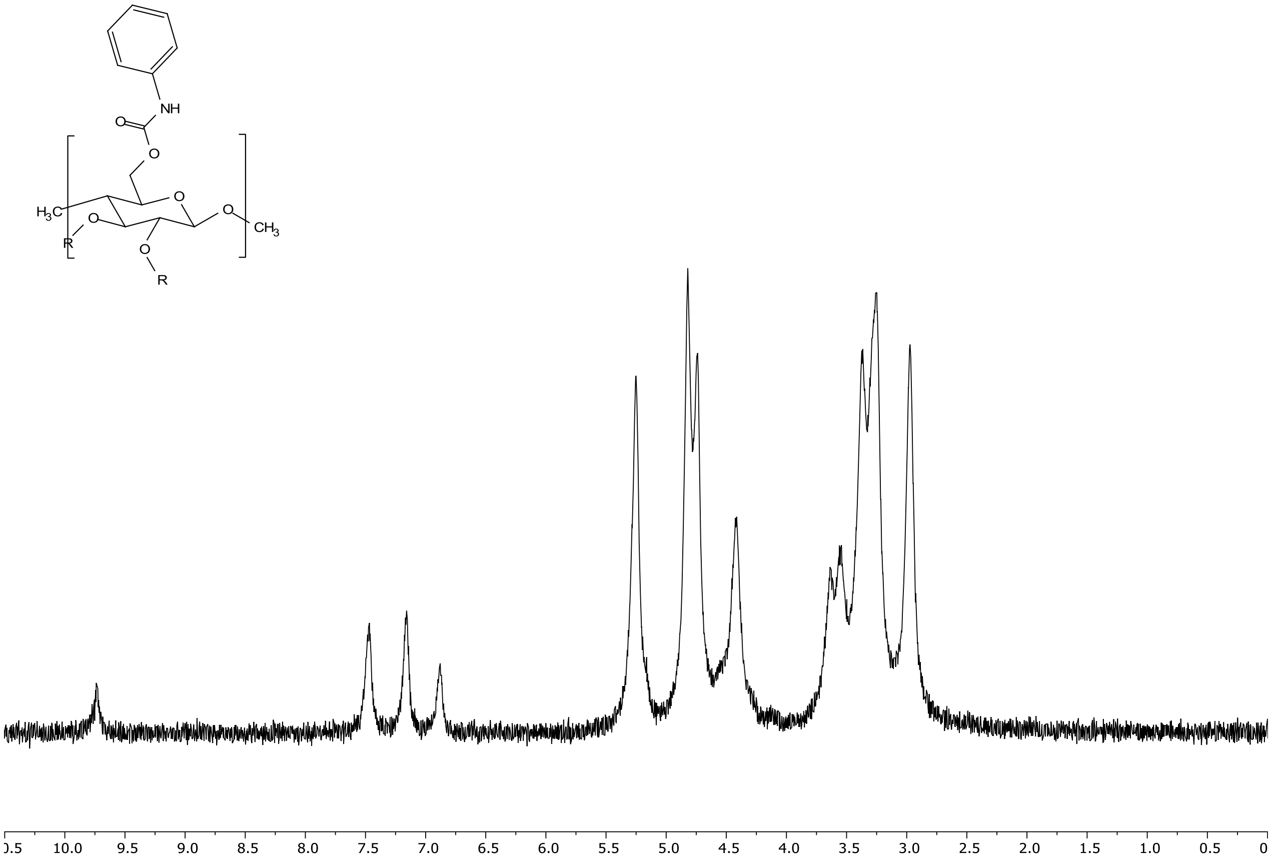

^{1}H and diffusion-edited ^{1}H spectra of **Cellulose *N*-phenyl carbamate DS 0.14** in DMSO-d_6/LiCl solution. (R: H or -CONHPh)

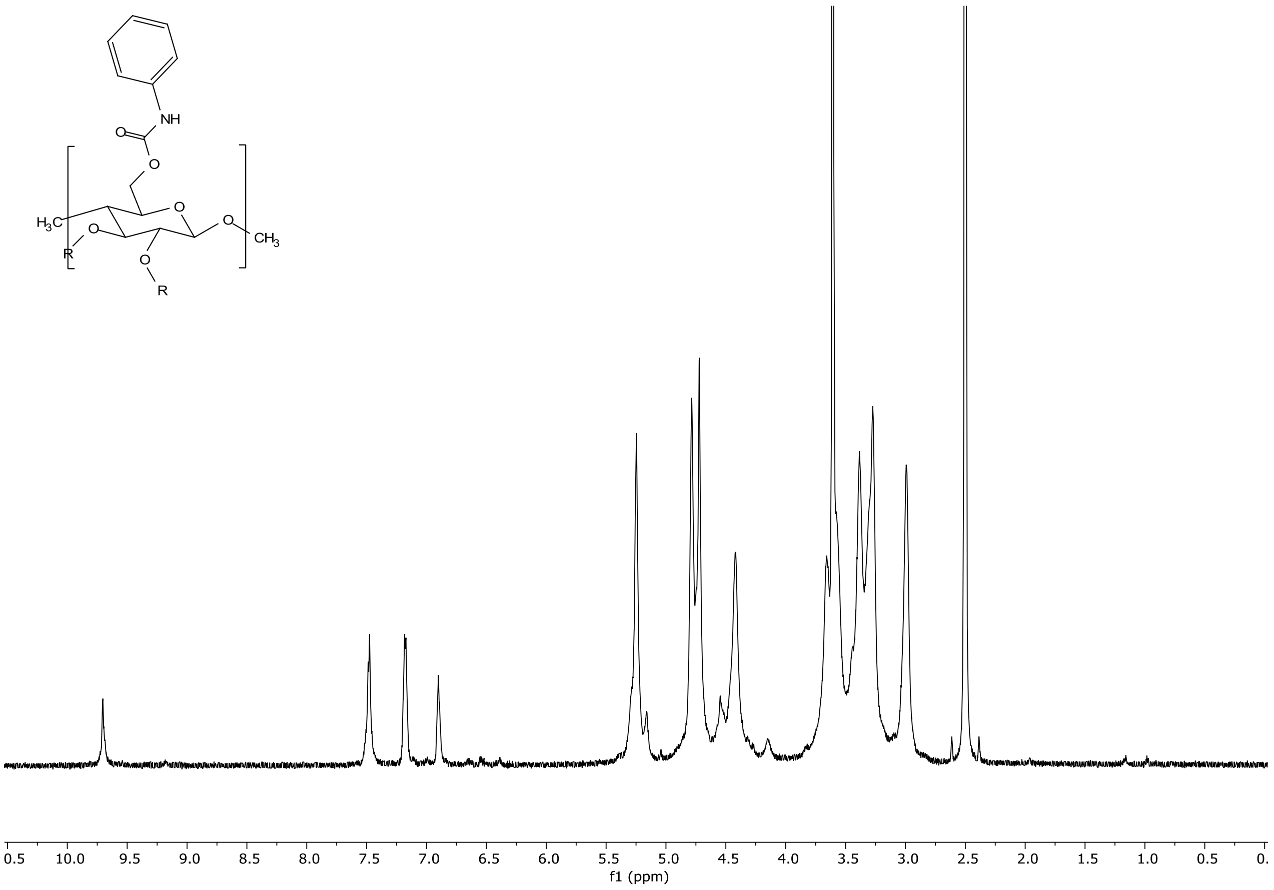

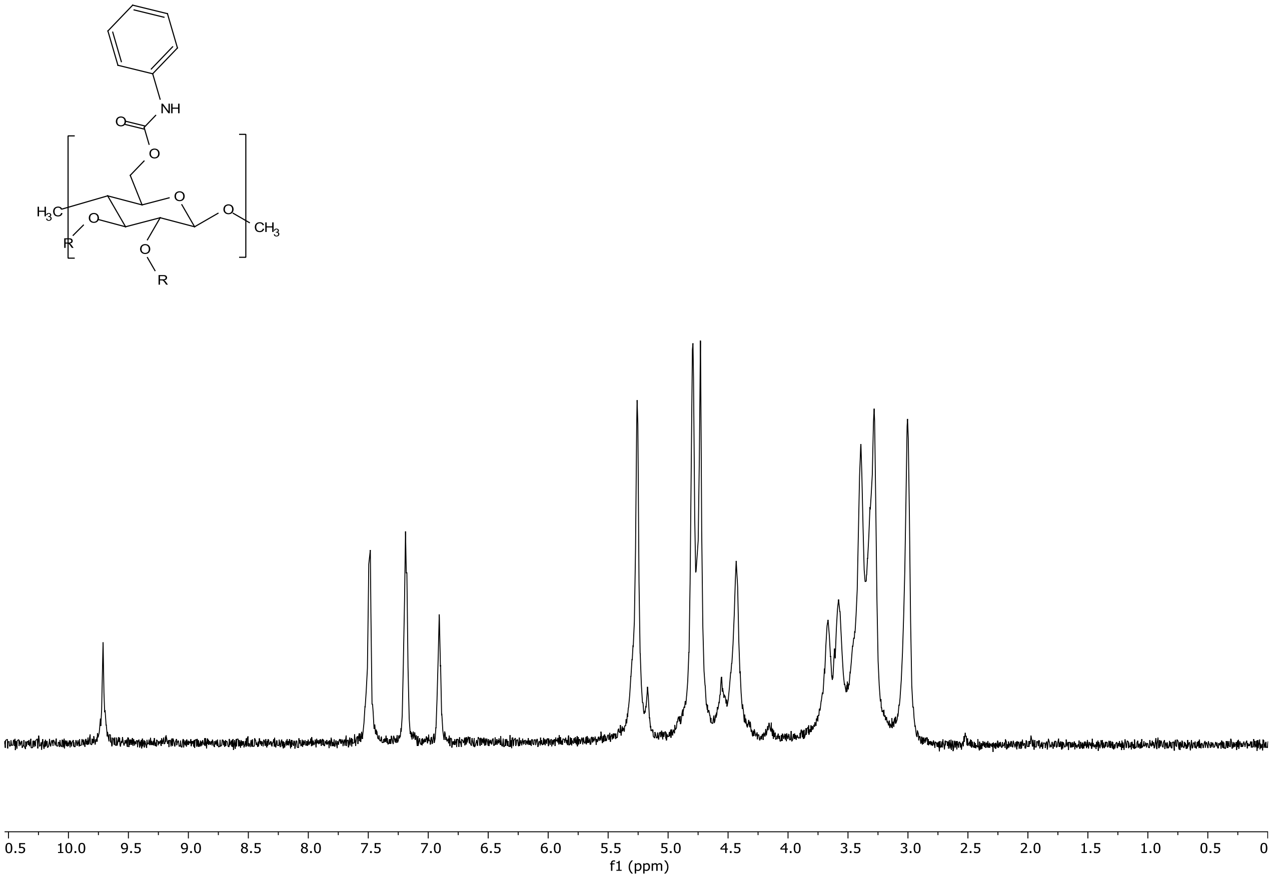

^{1}H and diffusion-edited ^{1}H spectra of **Cellulose *N*-phenyl carbamate DS 0.16** in DMSO-d_6/LiCl solution. (R: H or -CONHPh)

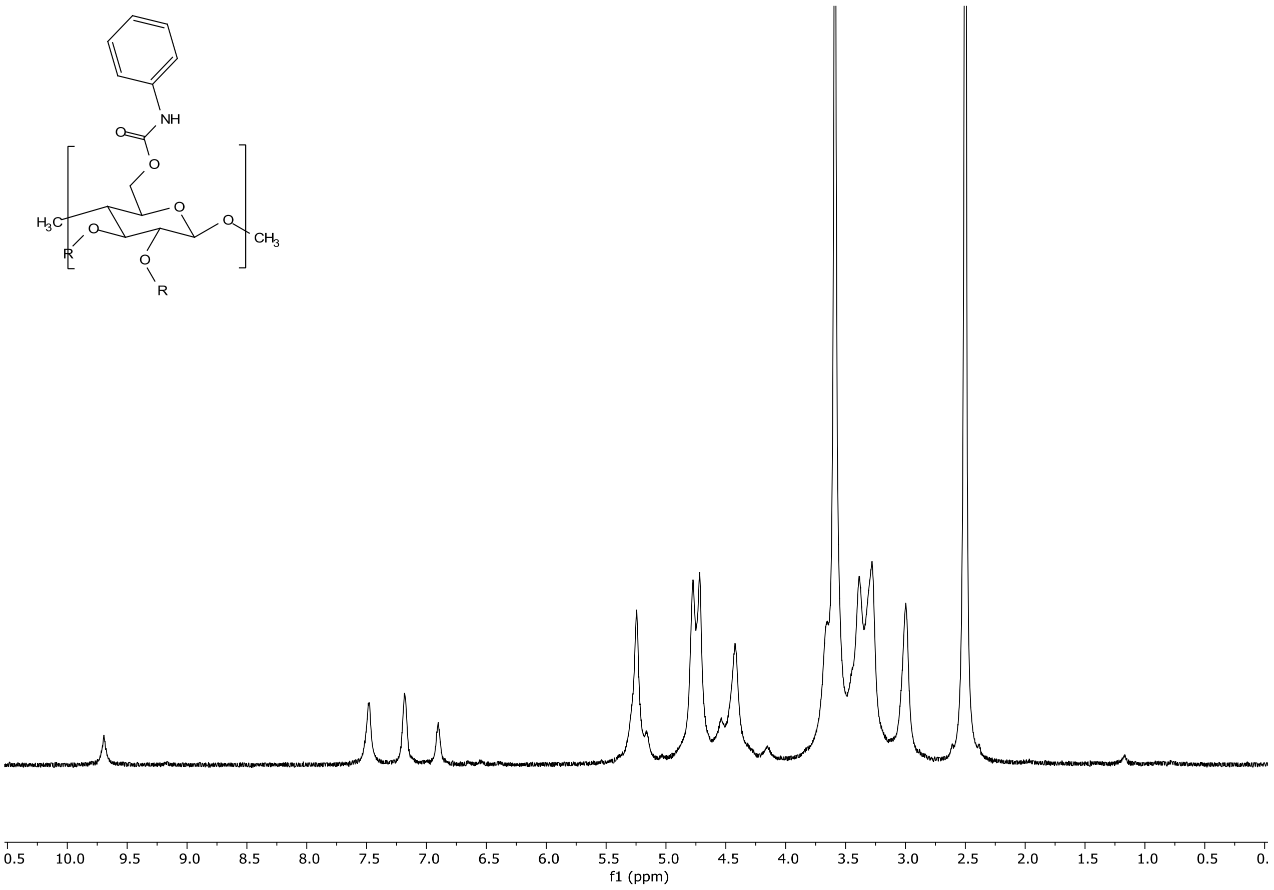

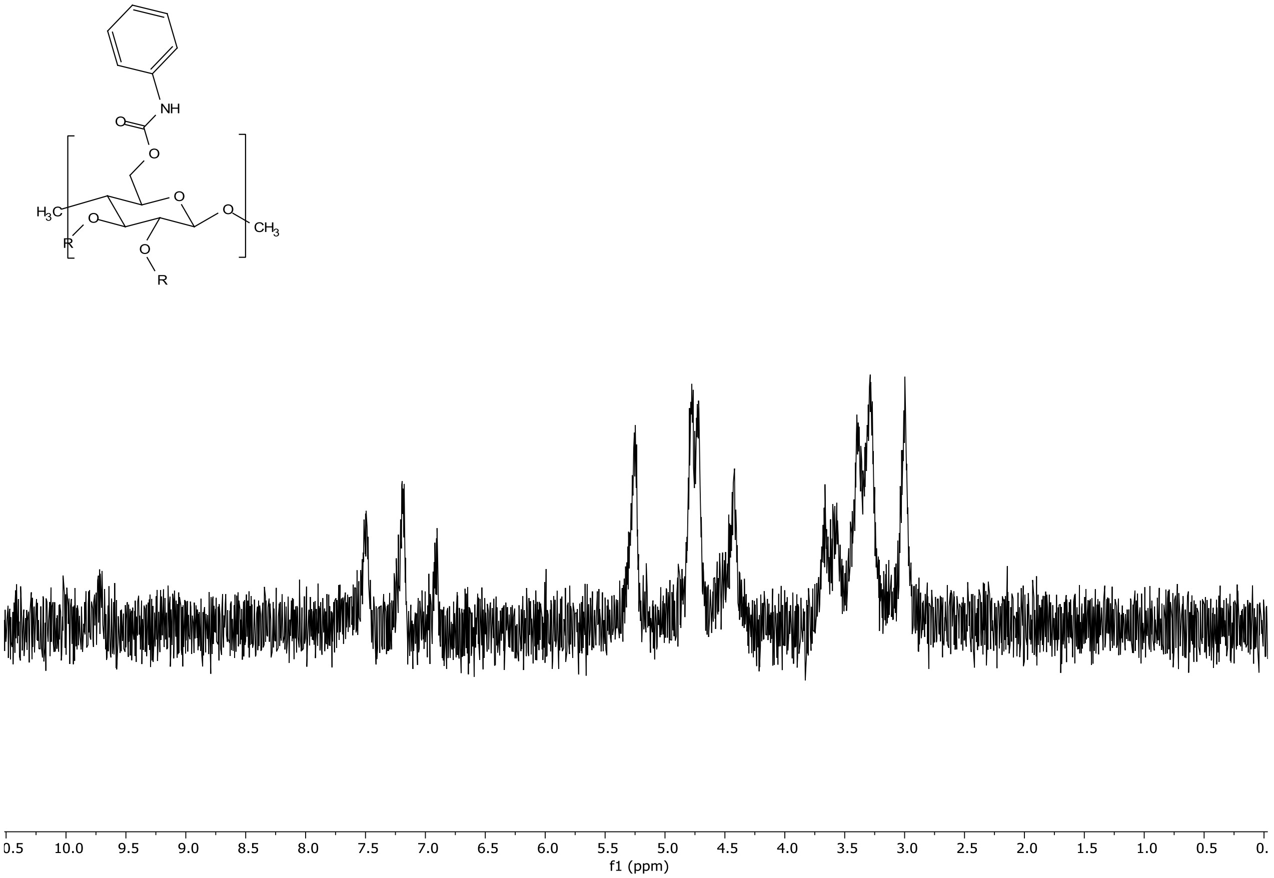

^{1}H and diffusion-edited ^{1}H spectra of **Cellulose *N*-4-methoxyphenyl carbamate DS 0.09** in DMSO-d_6/LiCl solution. (R: H or -CONH(4-MeO)Ph)

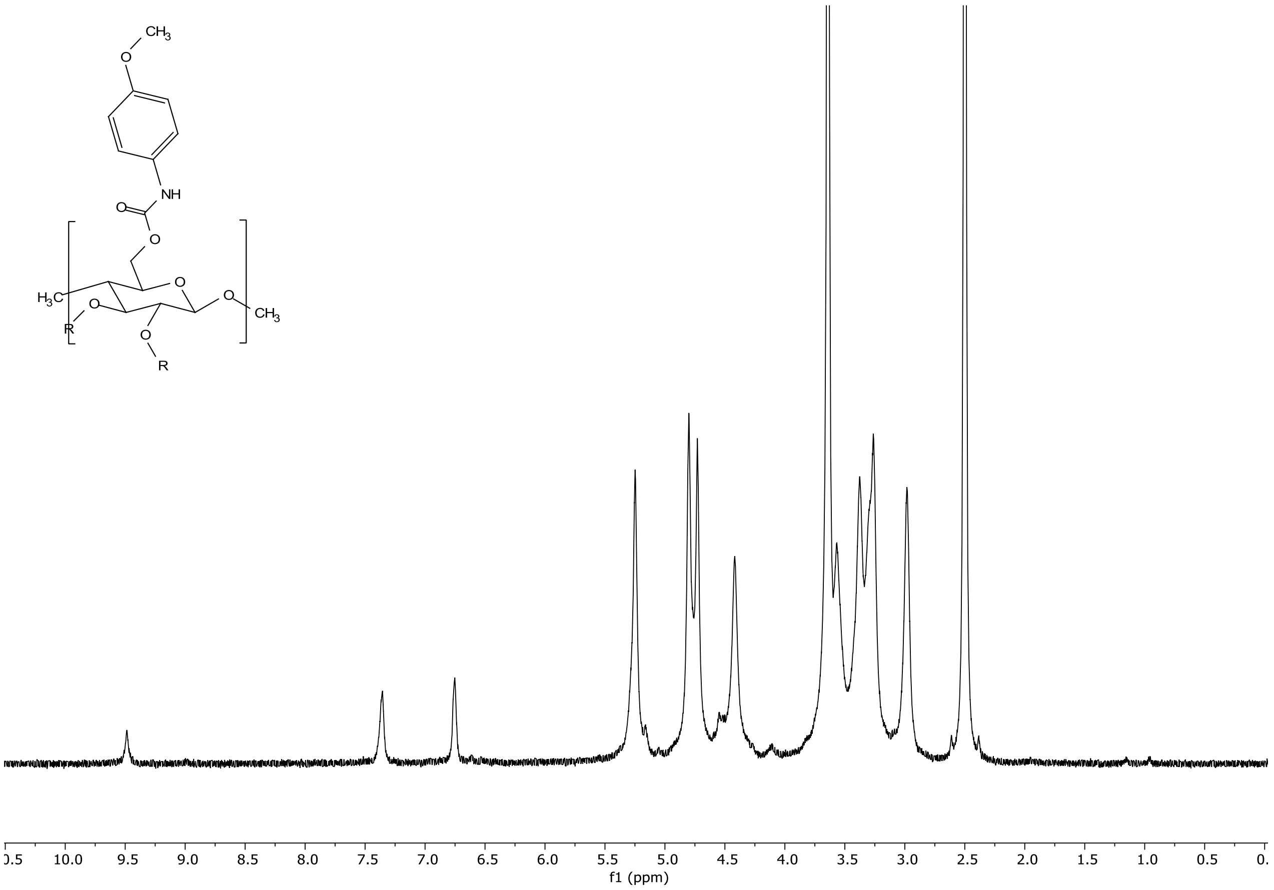

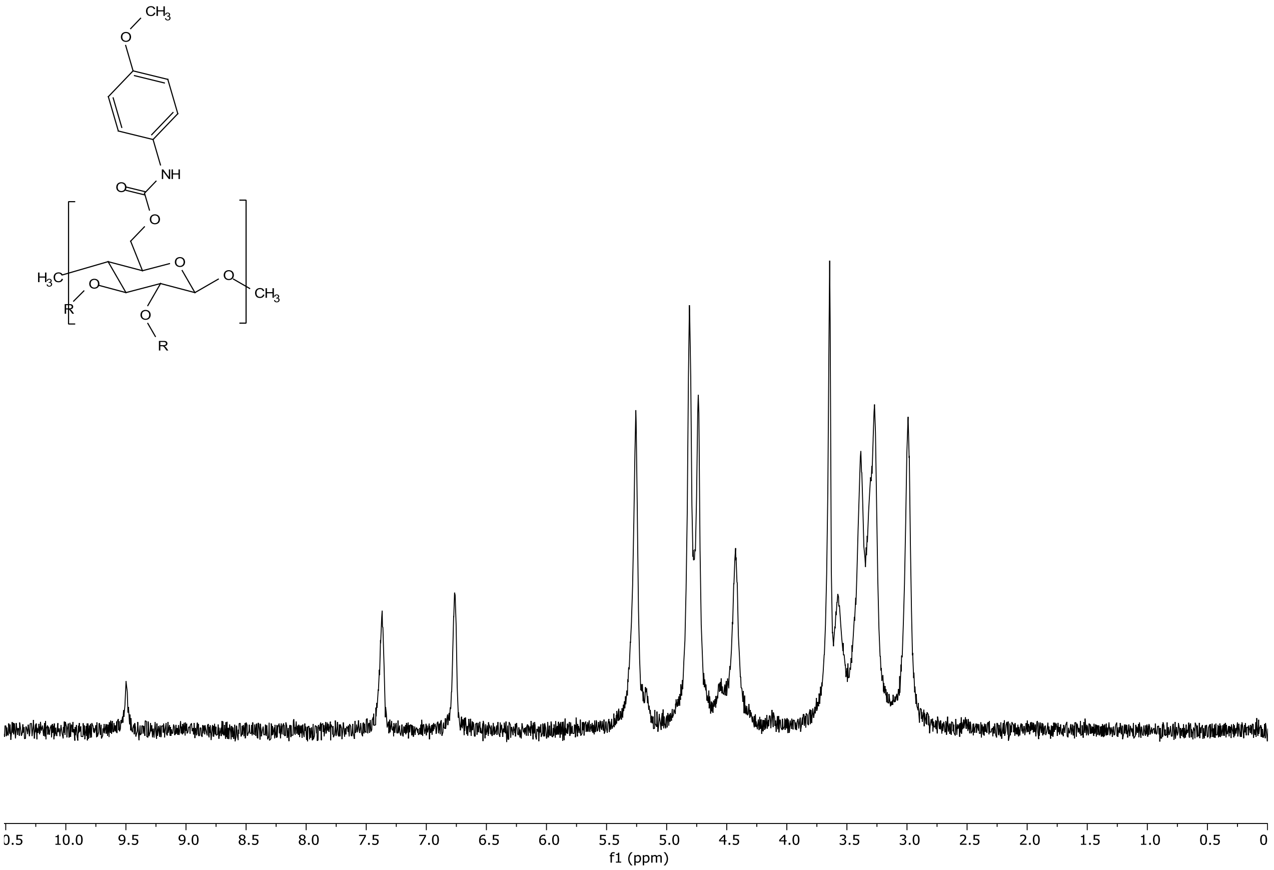

^{1}H and diffusion-edited ^{1}H spectra of **Cellulose *N*-4-methoxyphenyl carbamate DS 0.09** in DMSO-d_6/LiCl solution. (R: H or -CONH(4-MeO)Ph)

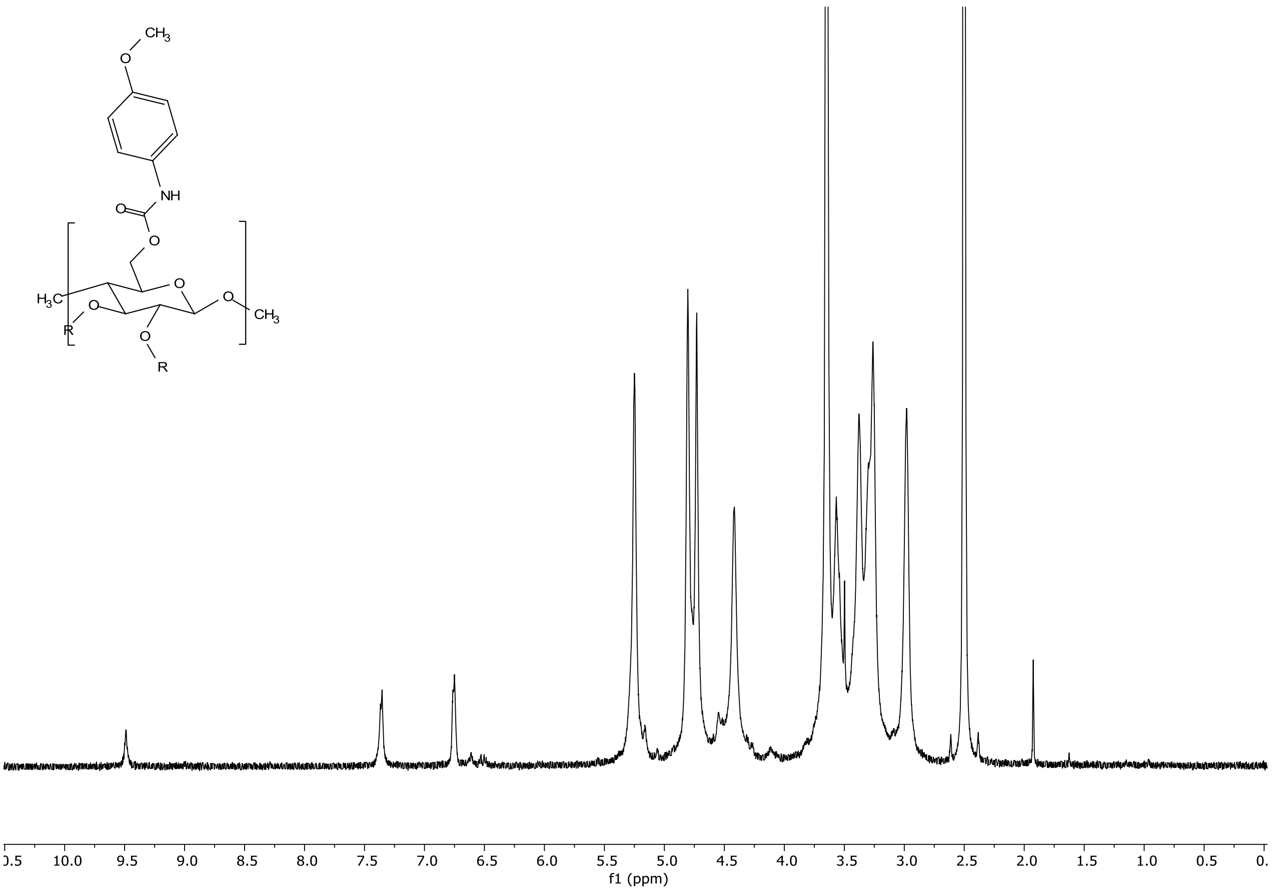

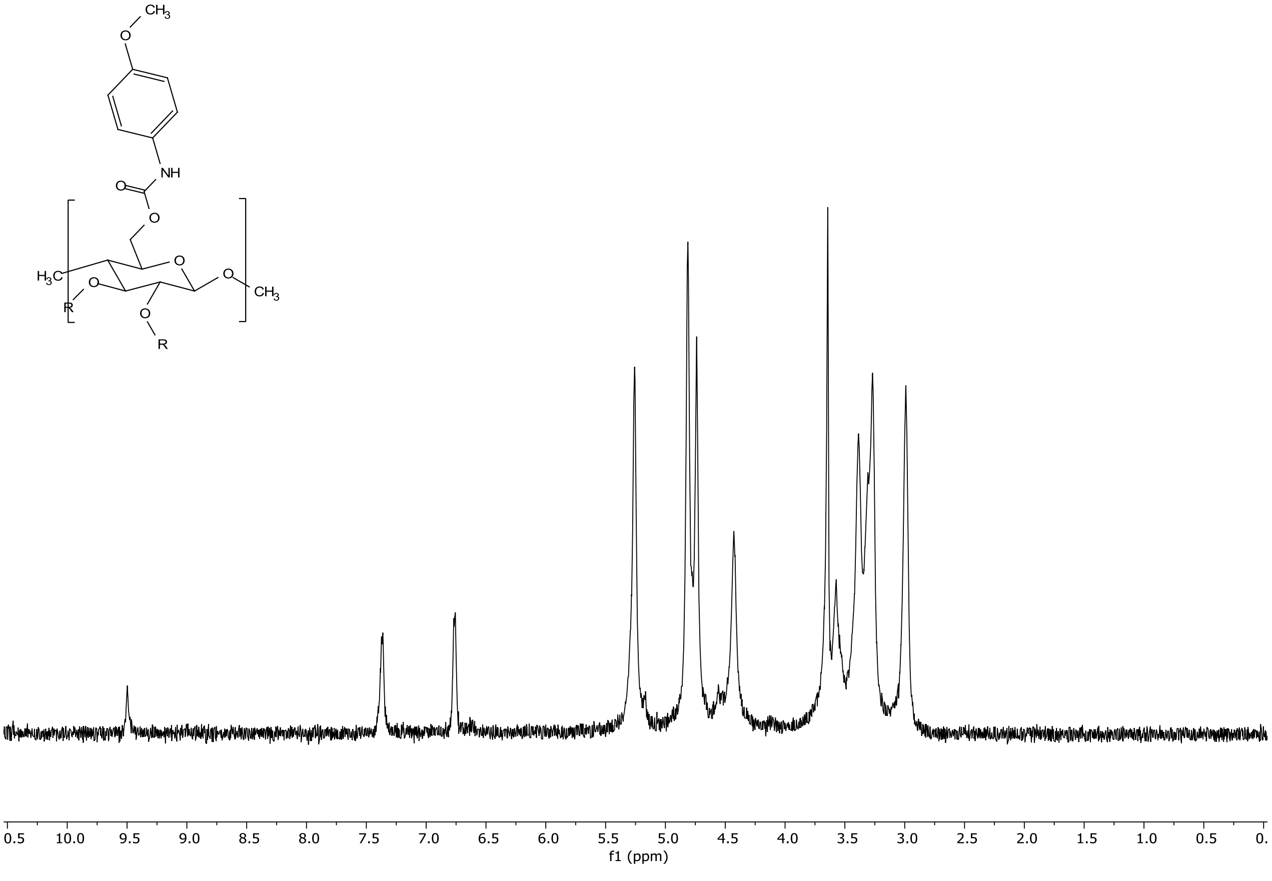

^{1}H and diffusion-edited ^{1}H spectra of **Cellulose *N*-4-methoxyphenyl carbamate DS 0.09** in DMSO-d_6/LiCl solution. (R: H or -CONH(4-MeO)Ph)

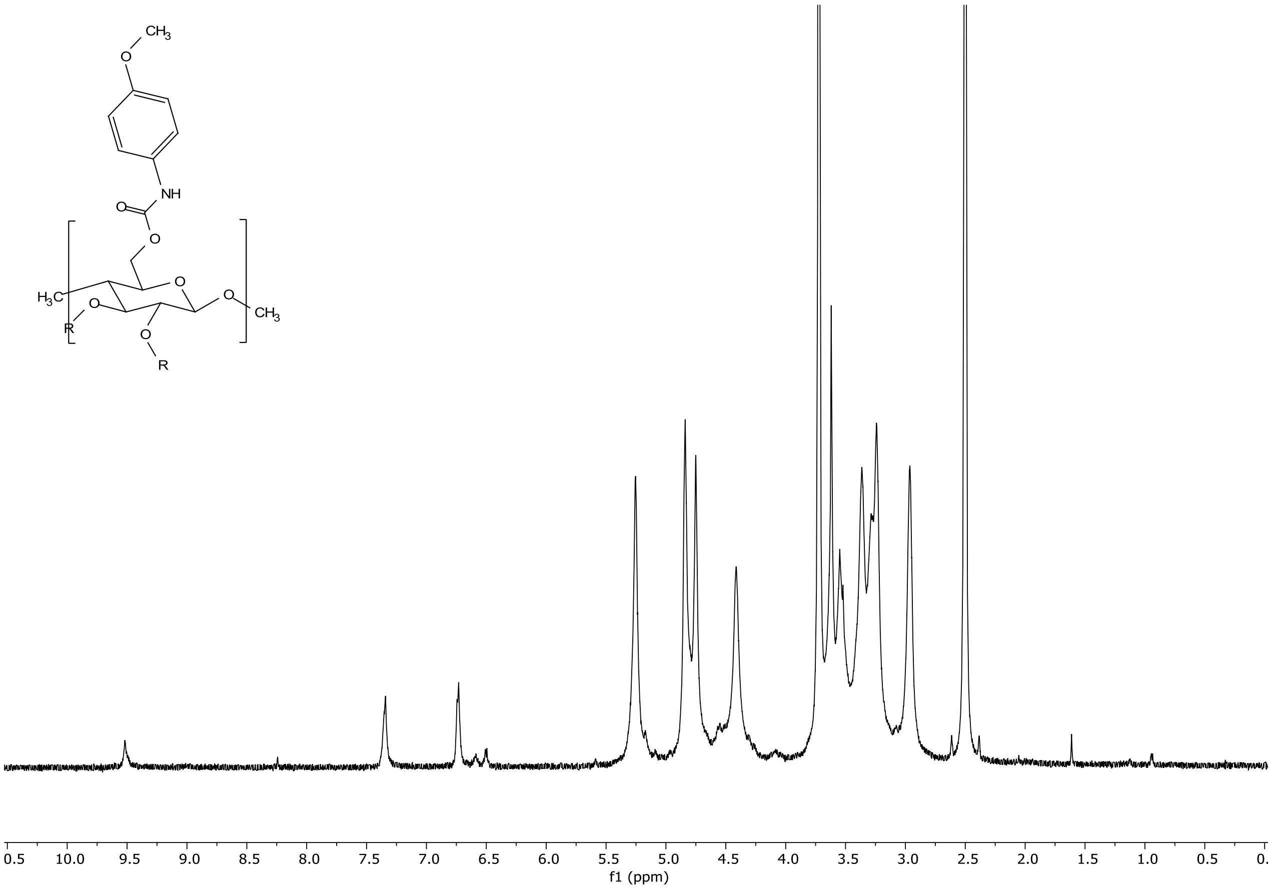

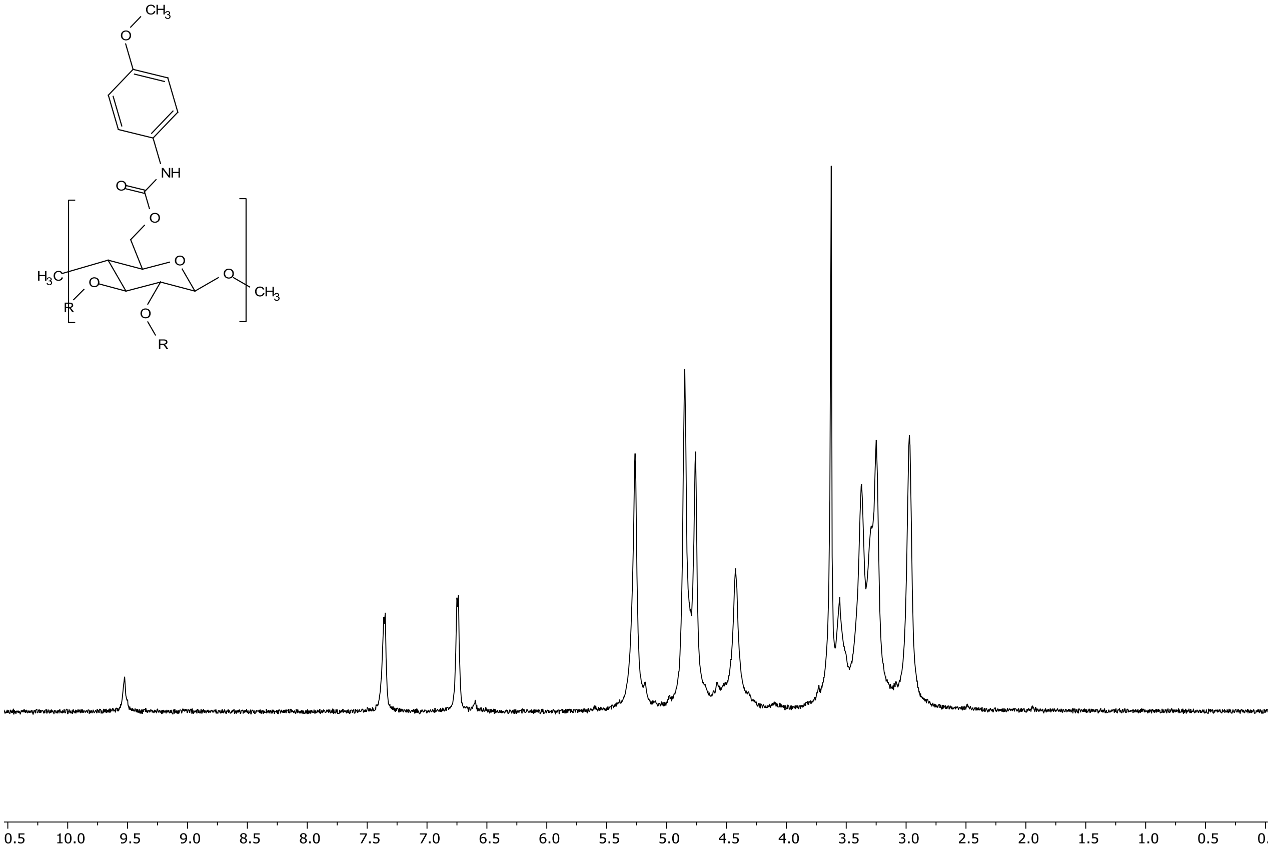

^1H and diffusion-edited ^1H spectra of **Cellulose *N*-4-nitrophenyl carbamate DS 0.05** in DMSO-d_6/LiCl solution. (R: H or -CONH(4-NO_2)Ph)

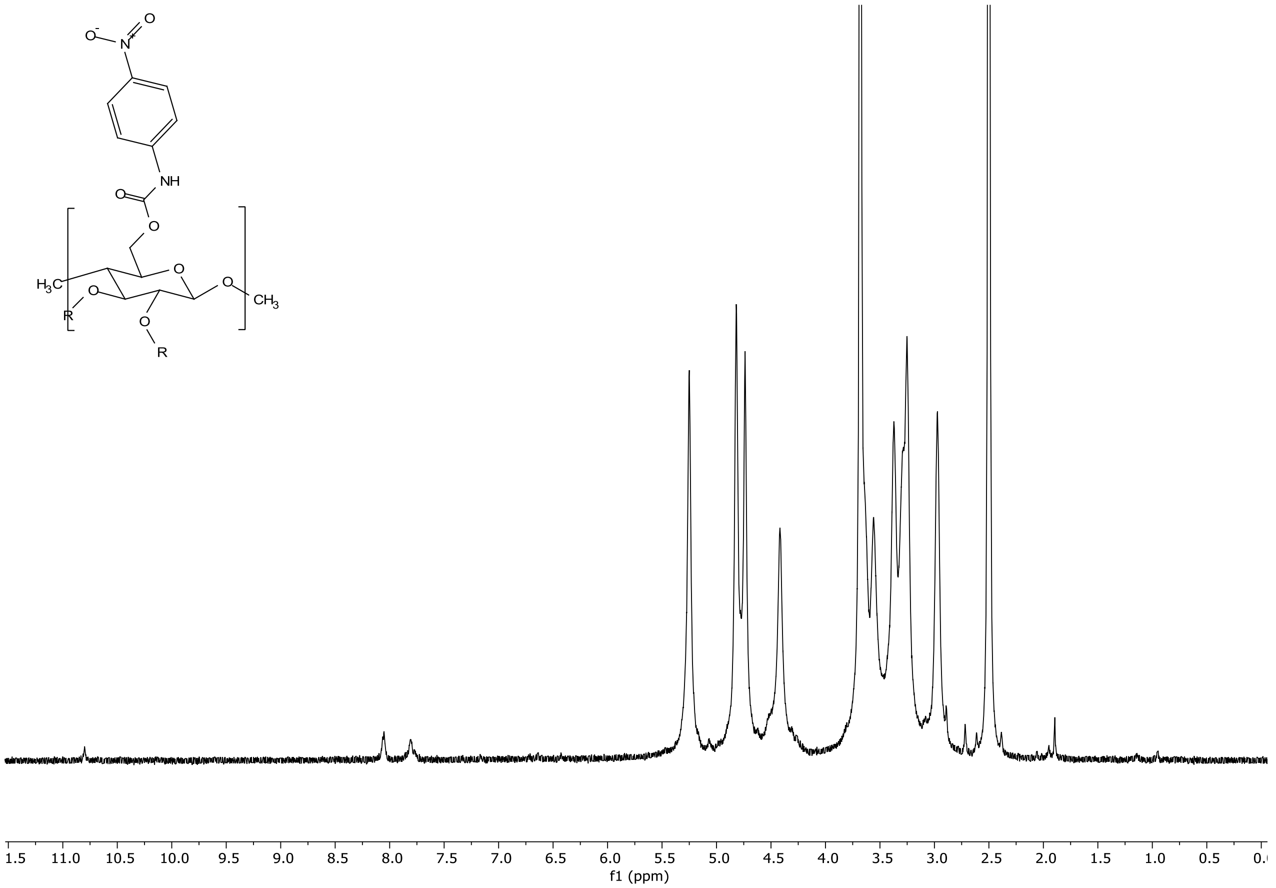

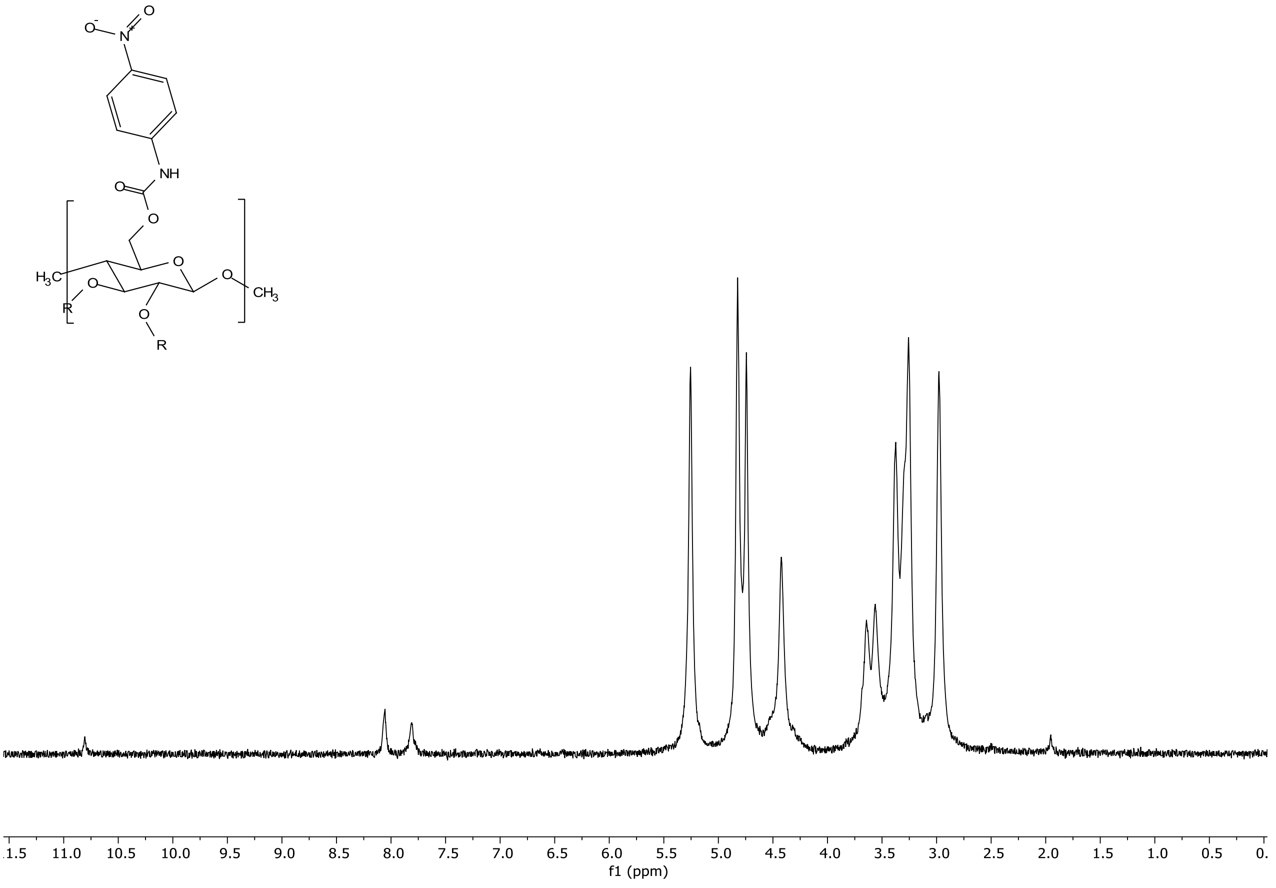

^1H and diffusion-edited ^1H spectra of **Cellulose *N*-4-nitrophenyl carbamate DS 0.06** in DMSO-d_6/LiCl solution. (R: H or -CONH(4-NO_2)Ph)

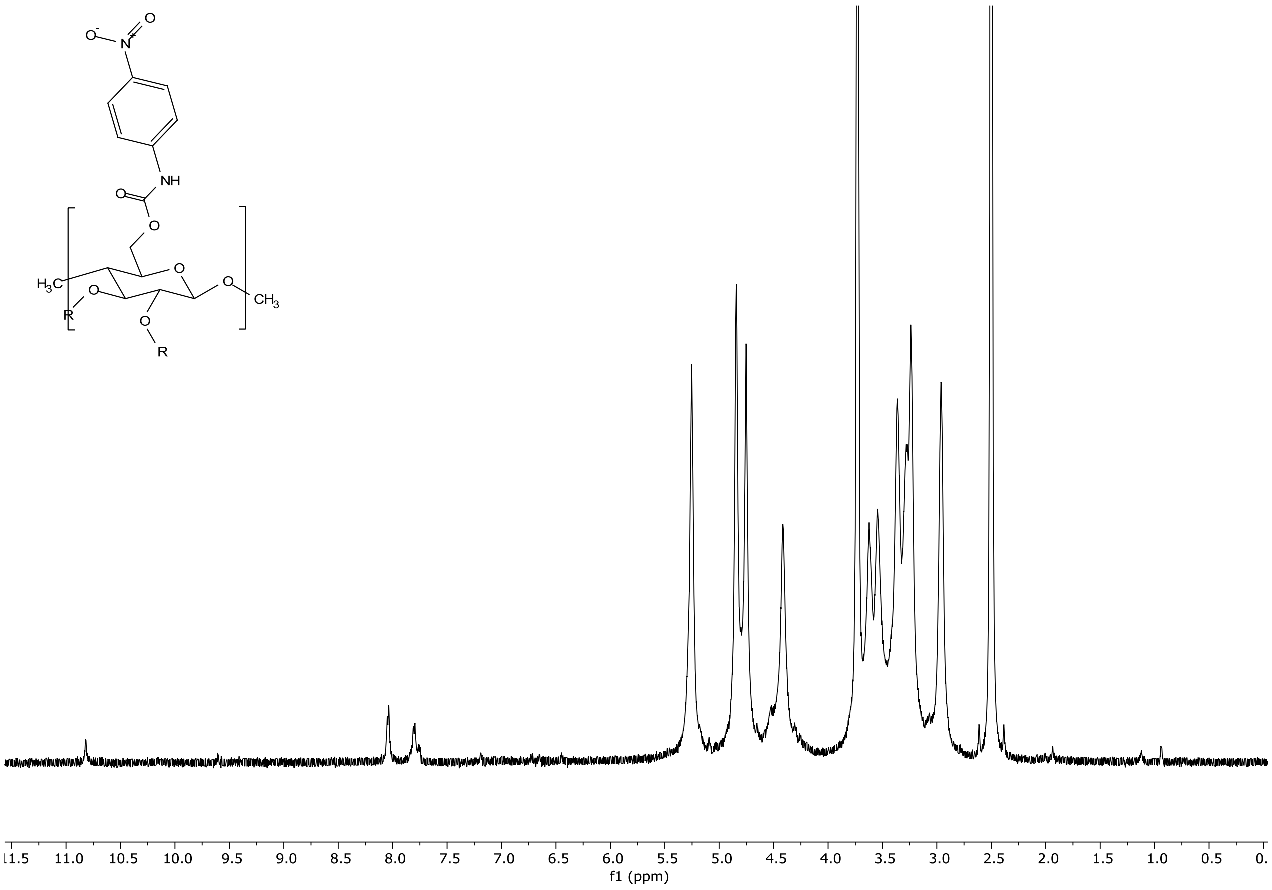

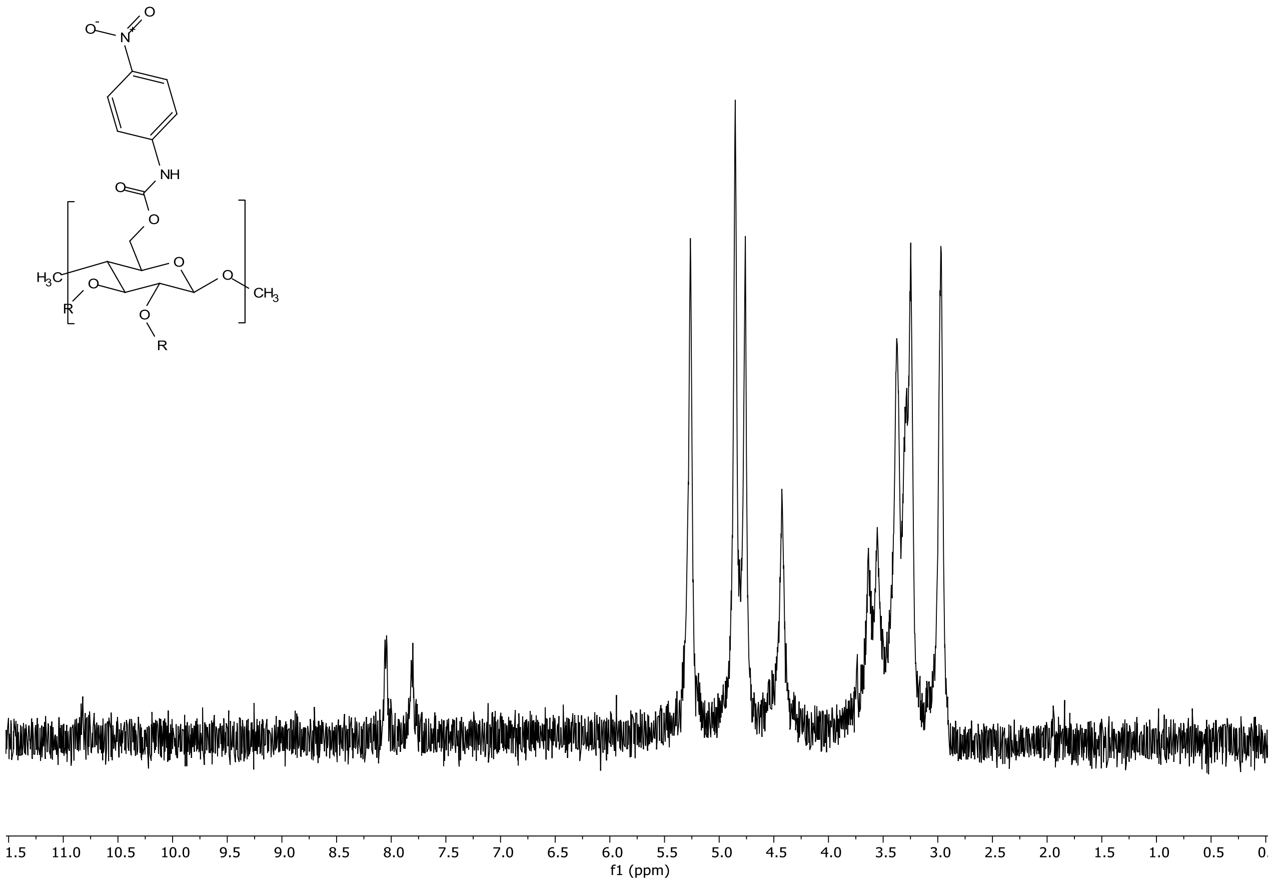

^1H and diffusion-edited ^1H spectra of **Cellulose *N*-4-nitrophenyl carbamate DS 0.08** in DMSO-d_6/LiCl solution. (R: H or -CONH(4-NO_2)Ph)

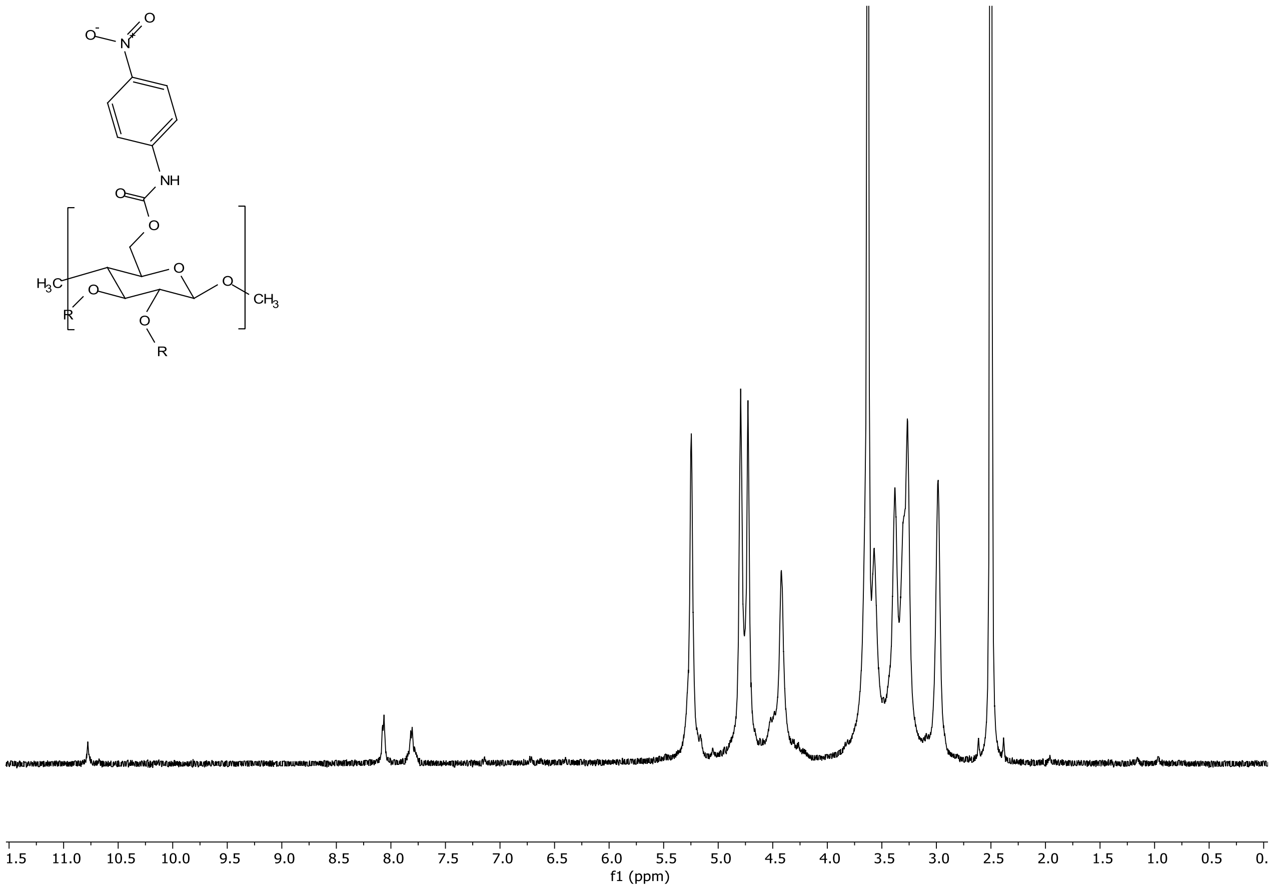

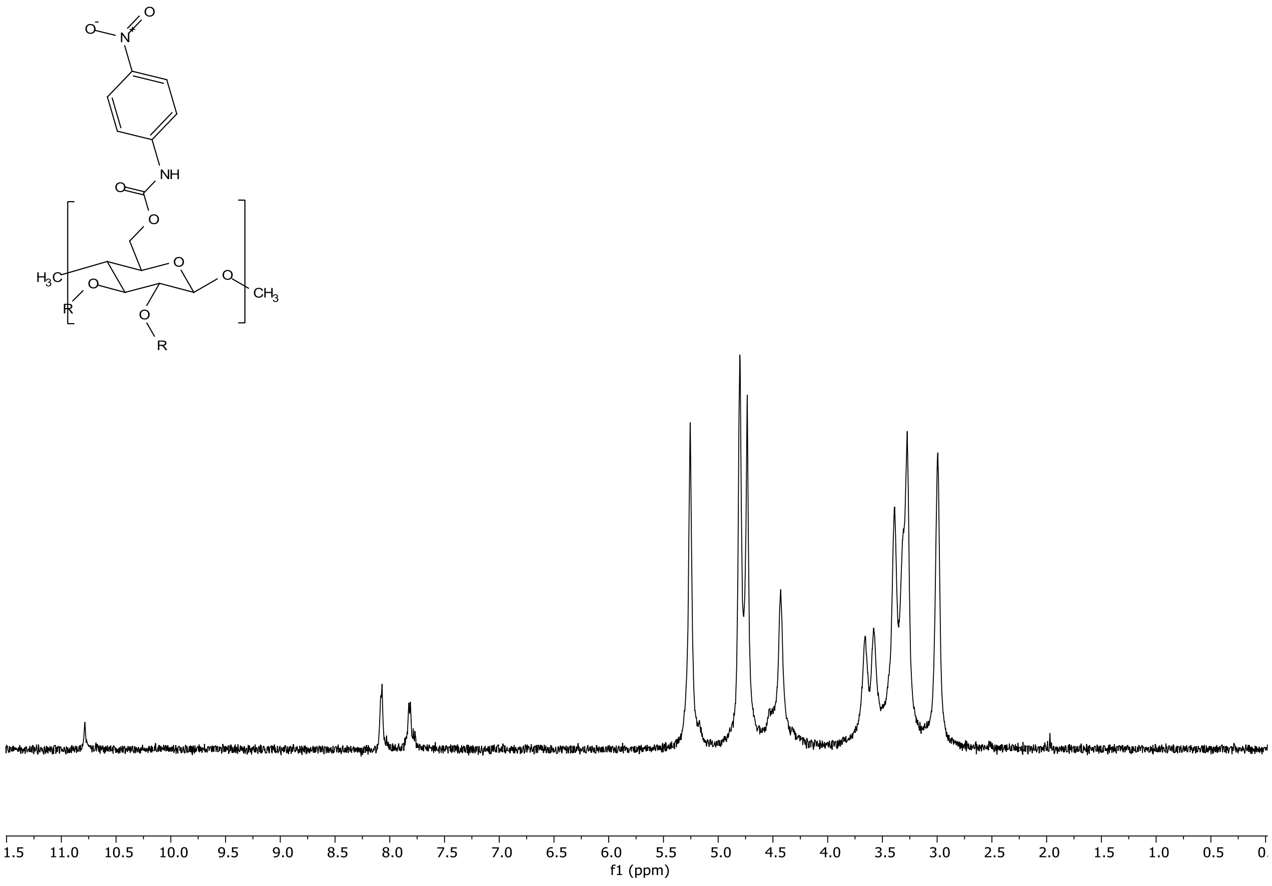

^{1}H and diffusion-edited ^{1}H spectra of **Cellulose *N*-styryl carbamate DS 0.10** in DMSO-d_6/LiCl solution. (R: H or -CONHCH=CHPh)

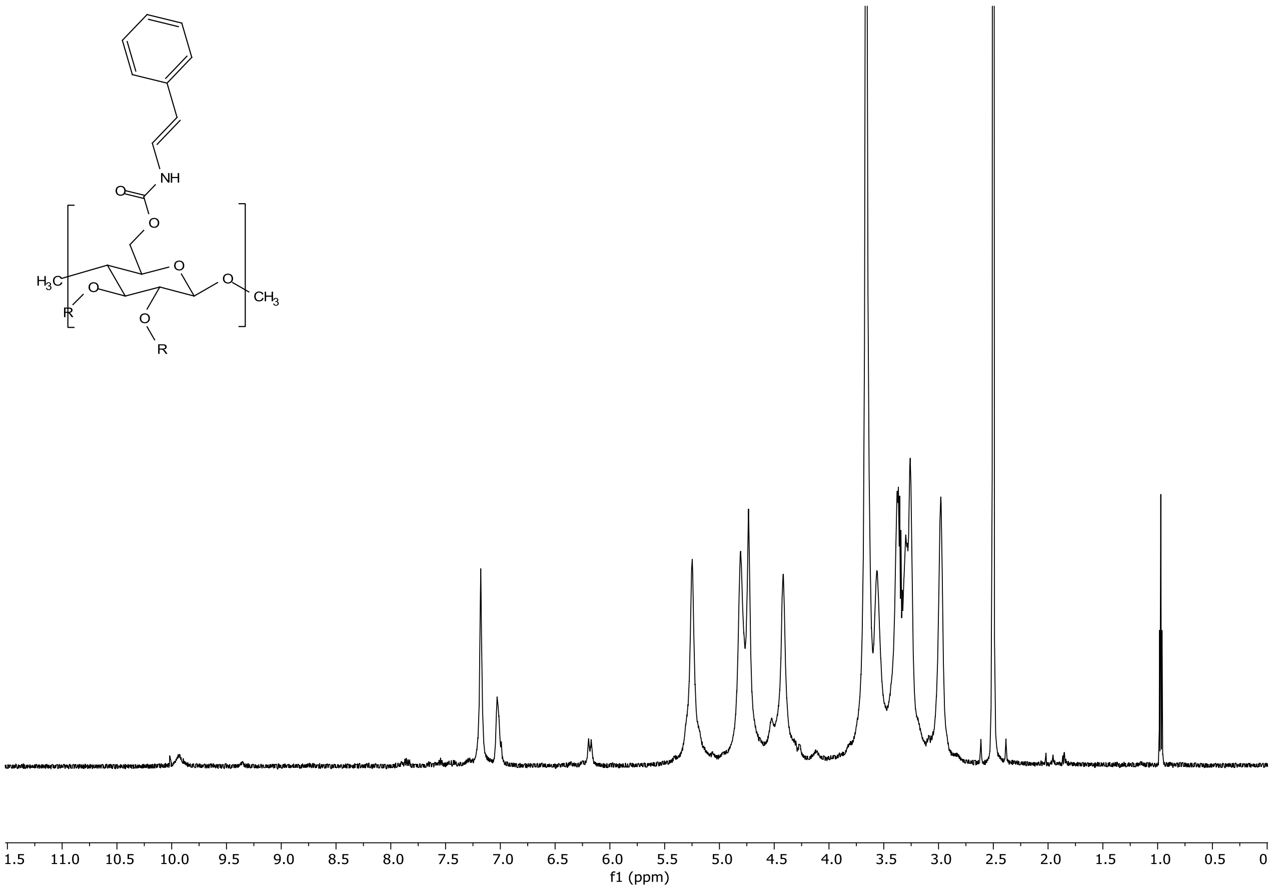

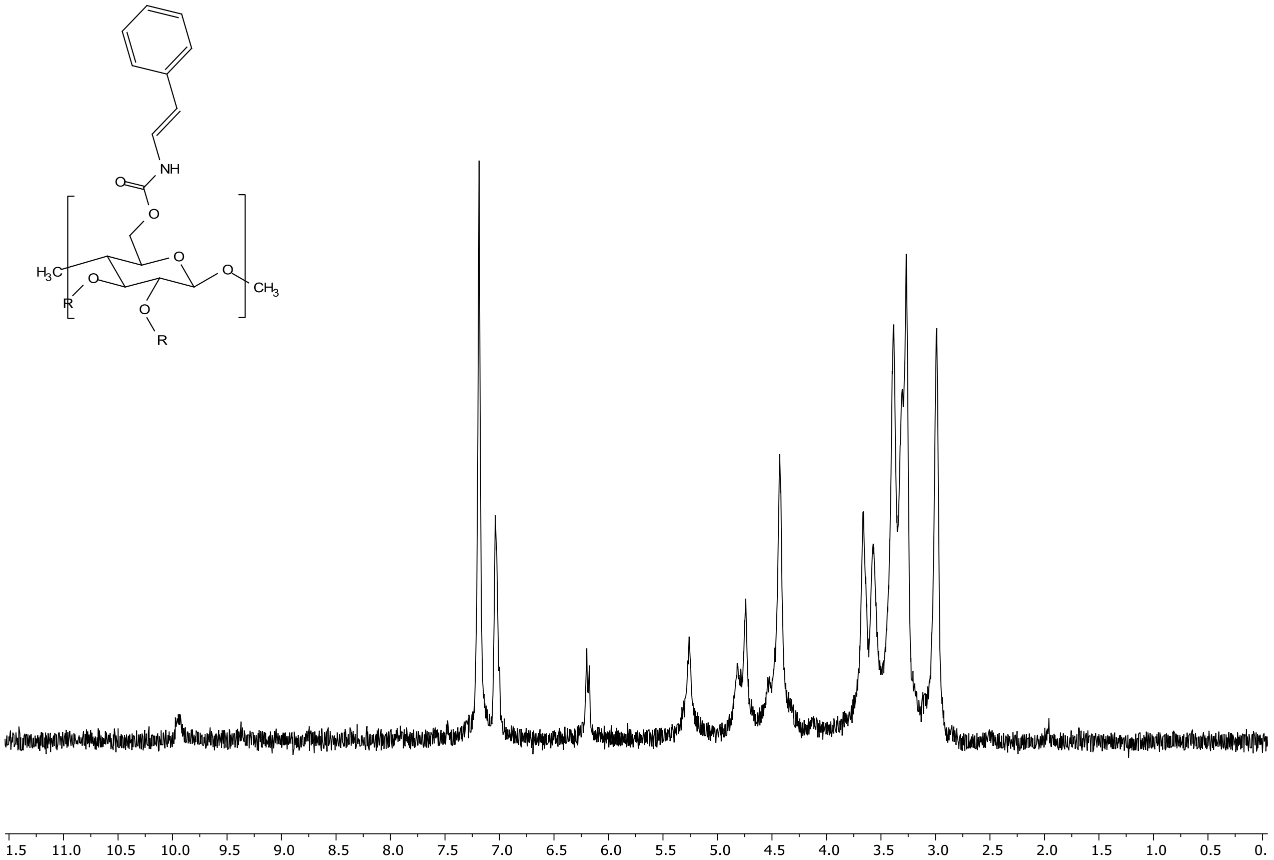

^1H and diffusion-edited ^1H spectra of **Cellulose *N*-styryl carbamate DS 0.21** in DMSO-d_6/LiCl solution. (R: H or -CONHCH=CHPh)

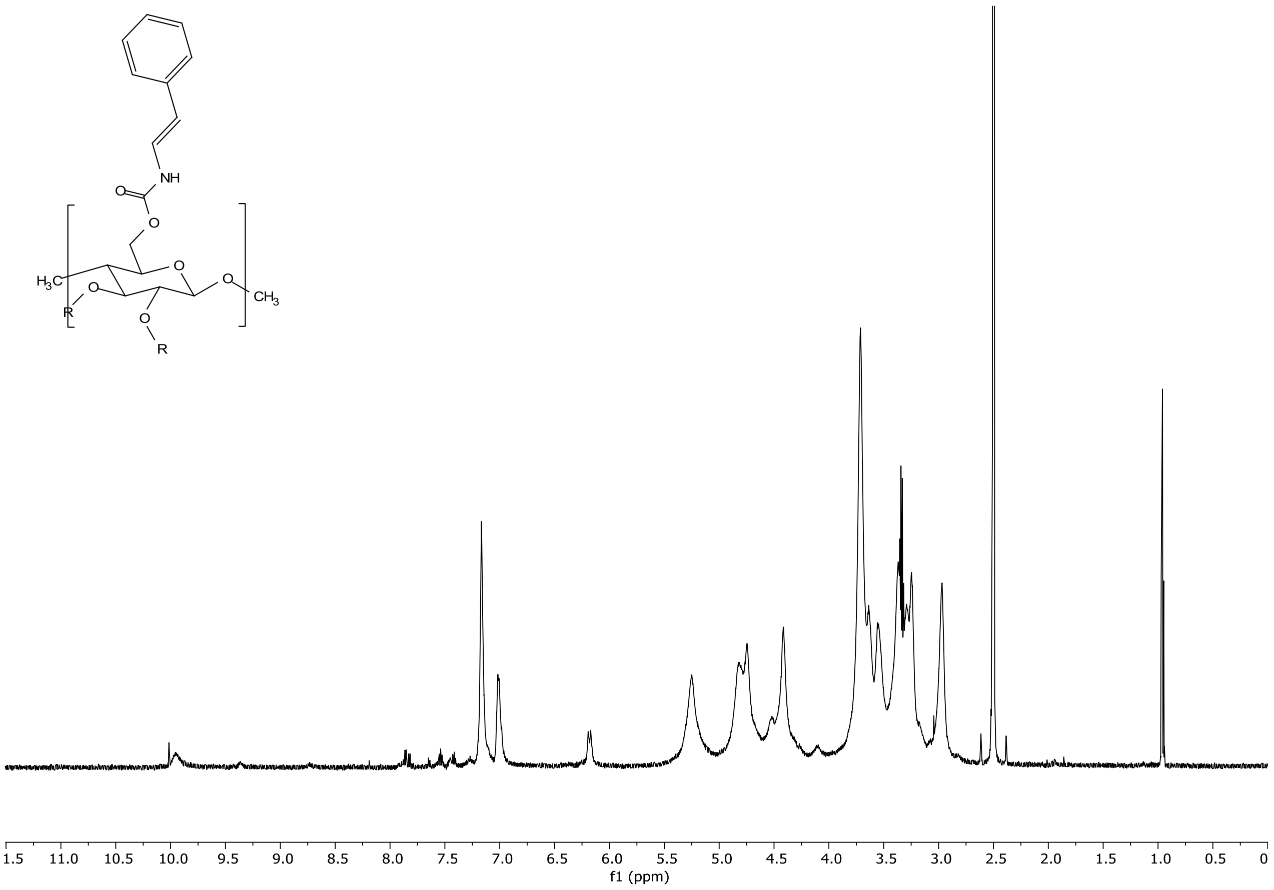

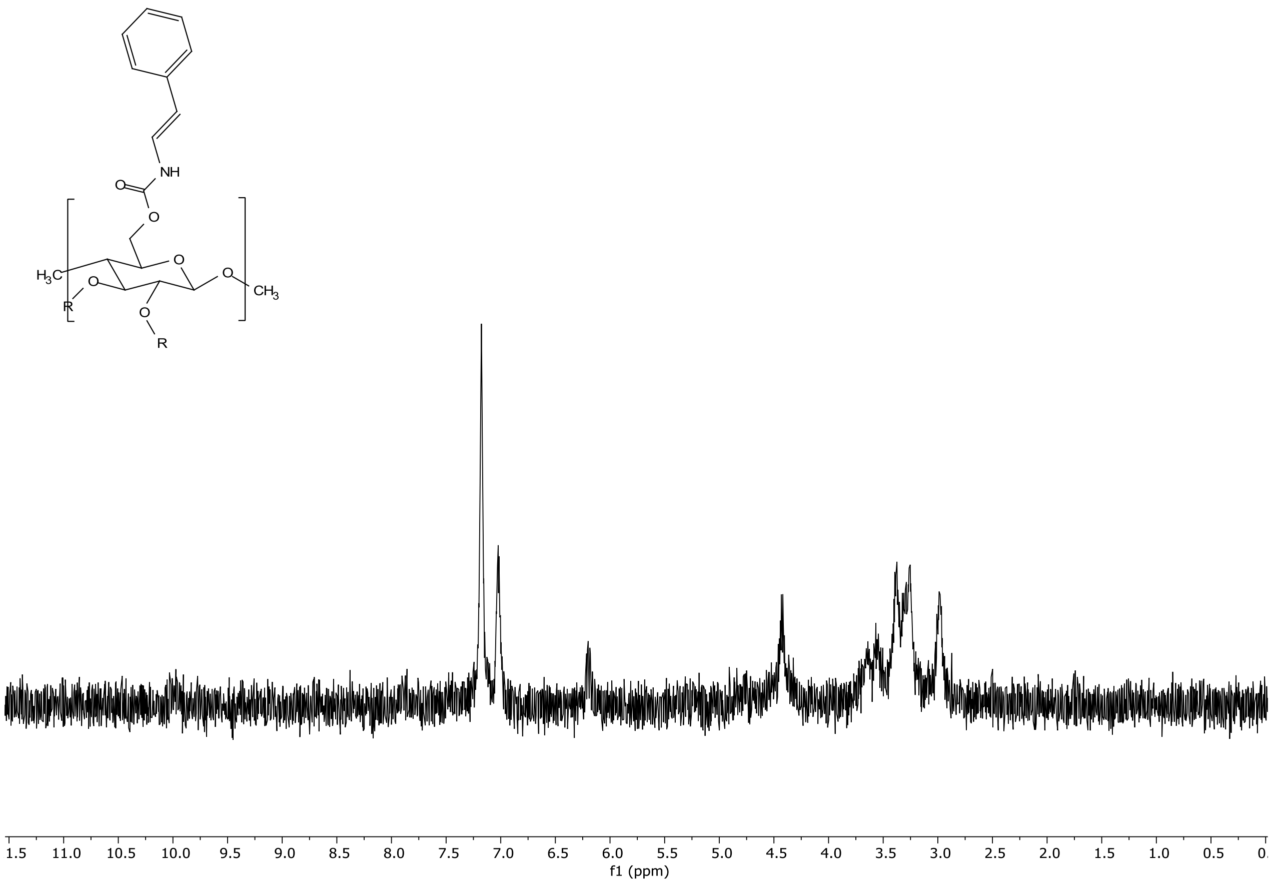

^{1}H and diffusion-edited ^{1}H spectra of **Cellulose *N*-styryl carbamate DS 0.32** in DMSO-d_6/LiCl solution. (R: H or -CONHCH=CHPh)

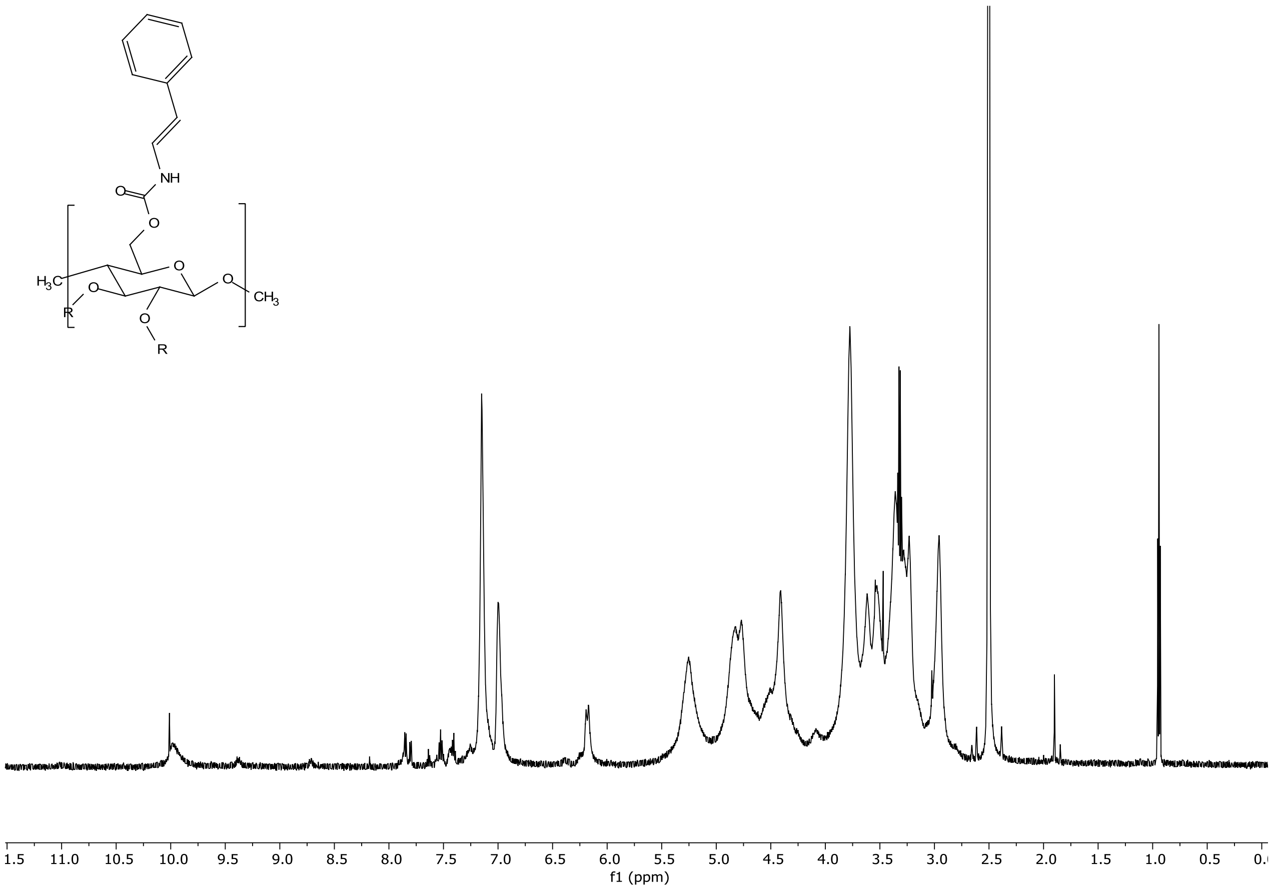

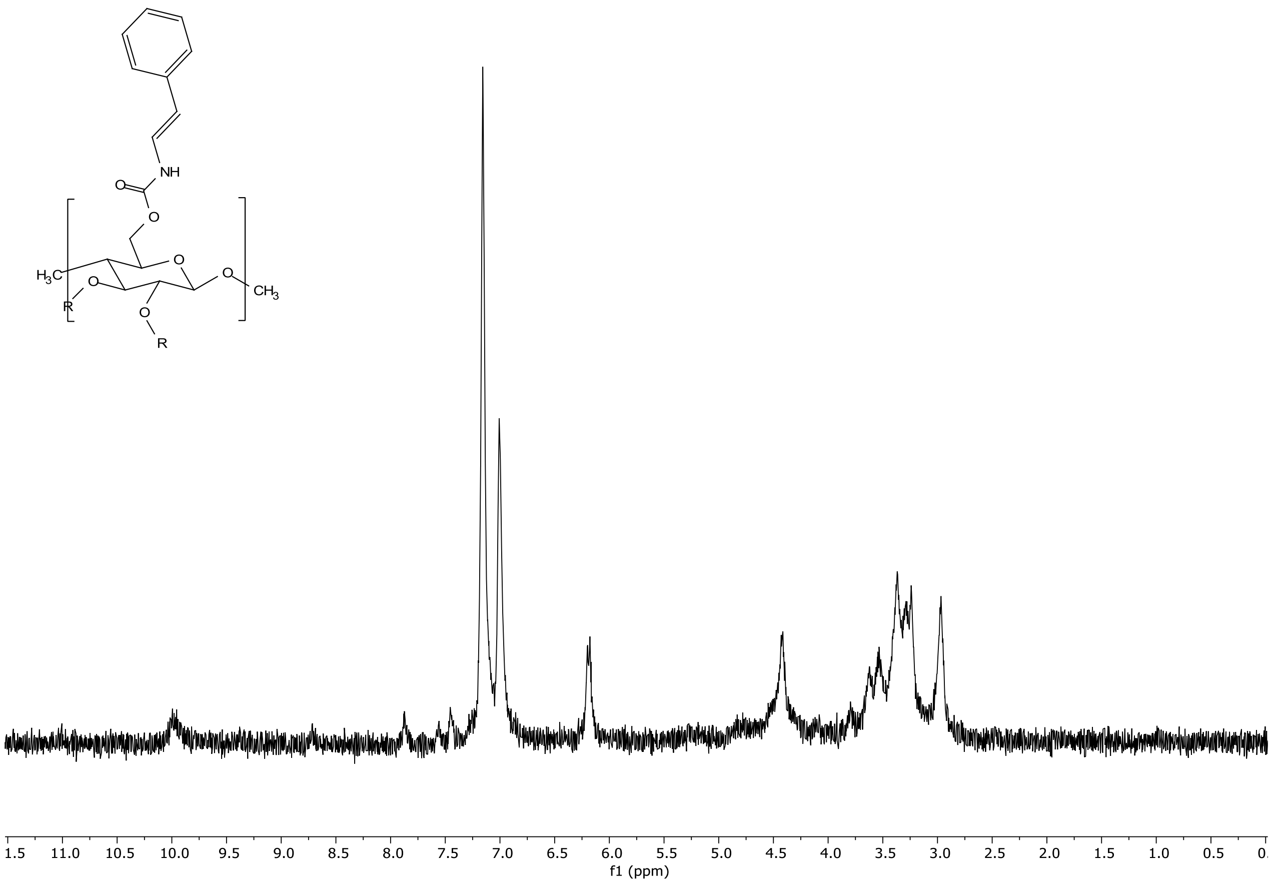

^{1}H and diffusion-edited ^{1}H spectra of **Cellulose *N*-phenyl carbamate DS 0.02** in DMSO-d_6/LiCl solution. (R: H or -CONHPh)

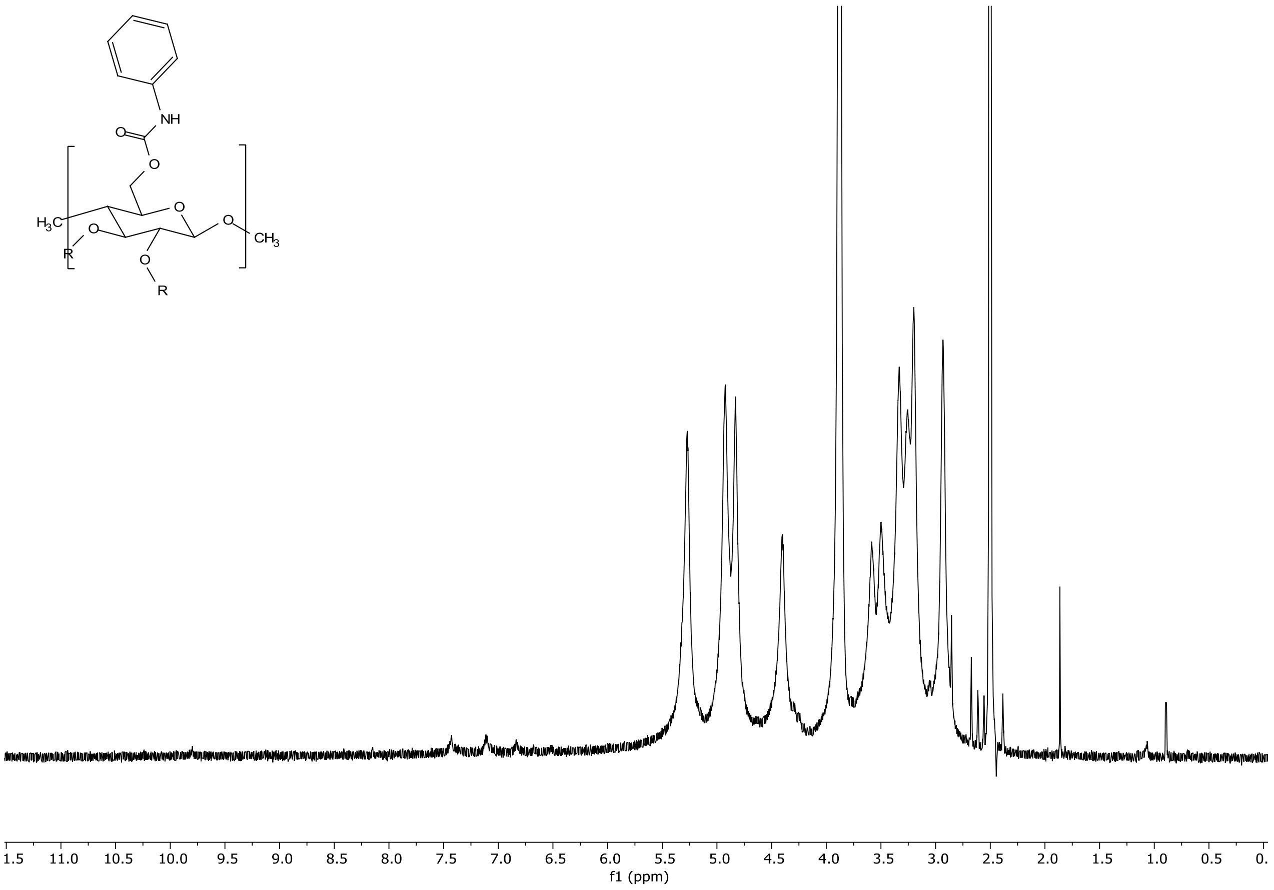

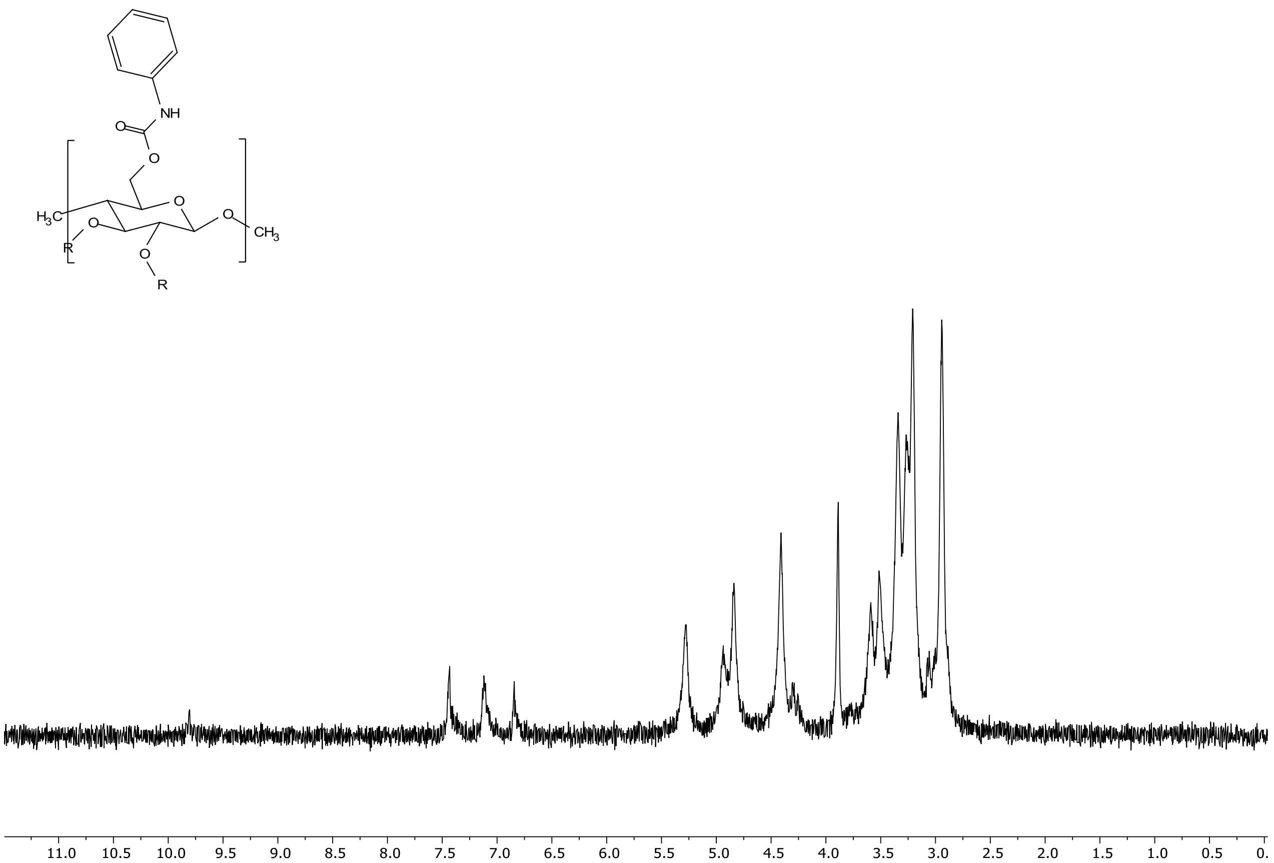

^{1}H and diffusion-edited ^{1}H spectra of **Cellulose *N*-phenyl carbamate DS 0.03** in DMSO-d_6/LiCl solution. (R: H or -CONHPh)

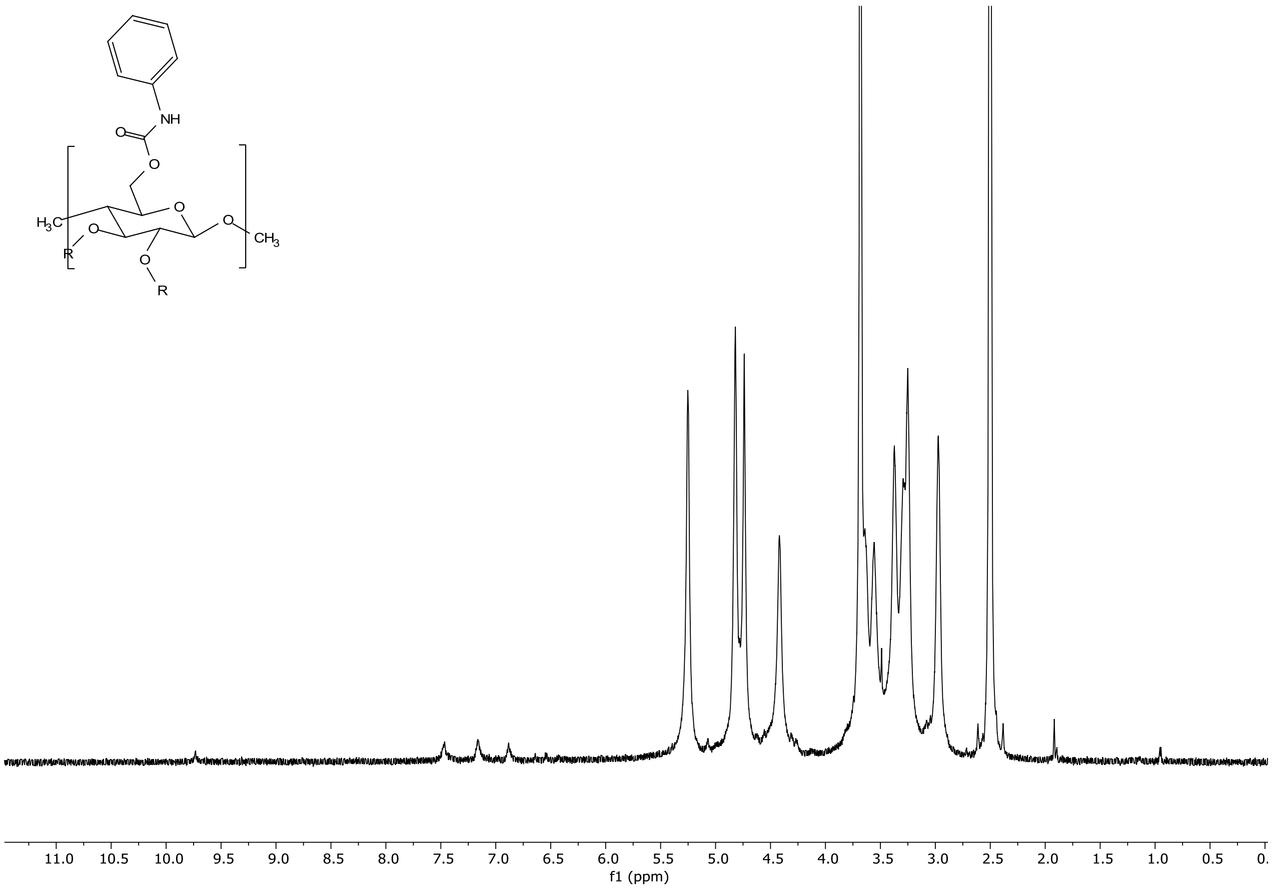

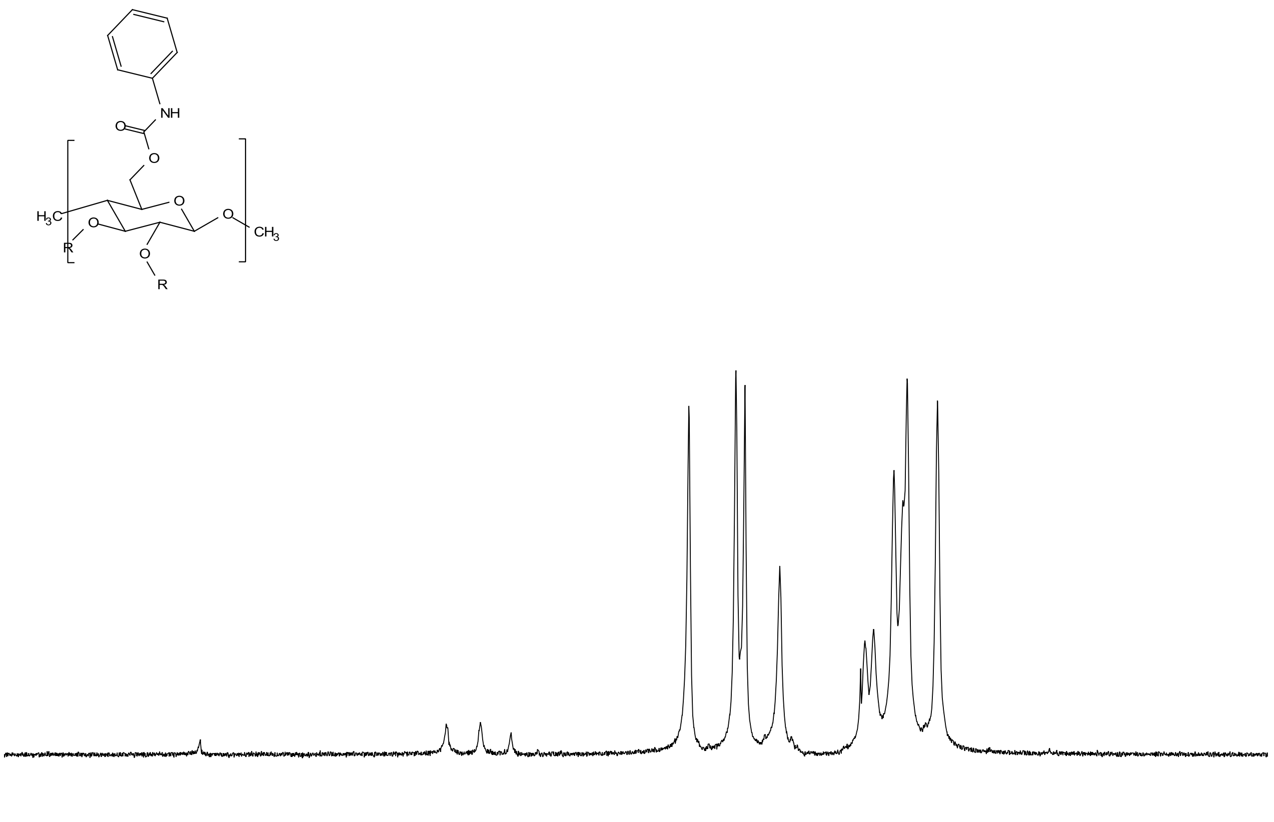

^{1}H and diffusion-edited ^{1}H spectra of **Cellulose *N*-phenyl carbamate DS 0.08** in DMSO-d_6/LiCl solution. (R: H or -CONHPh)

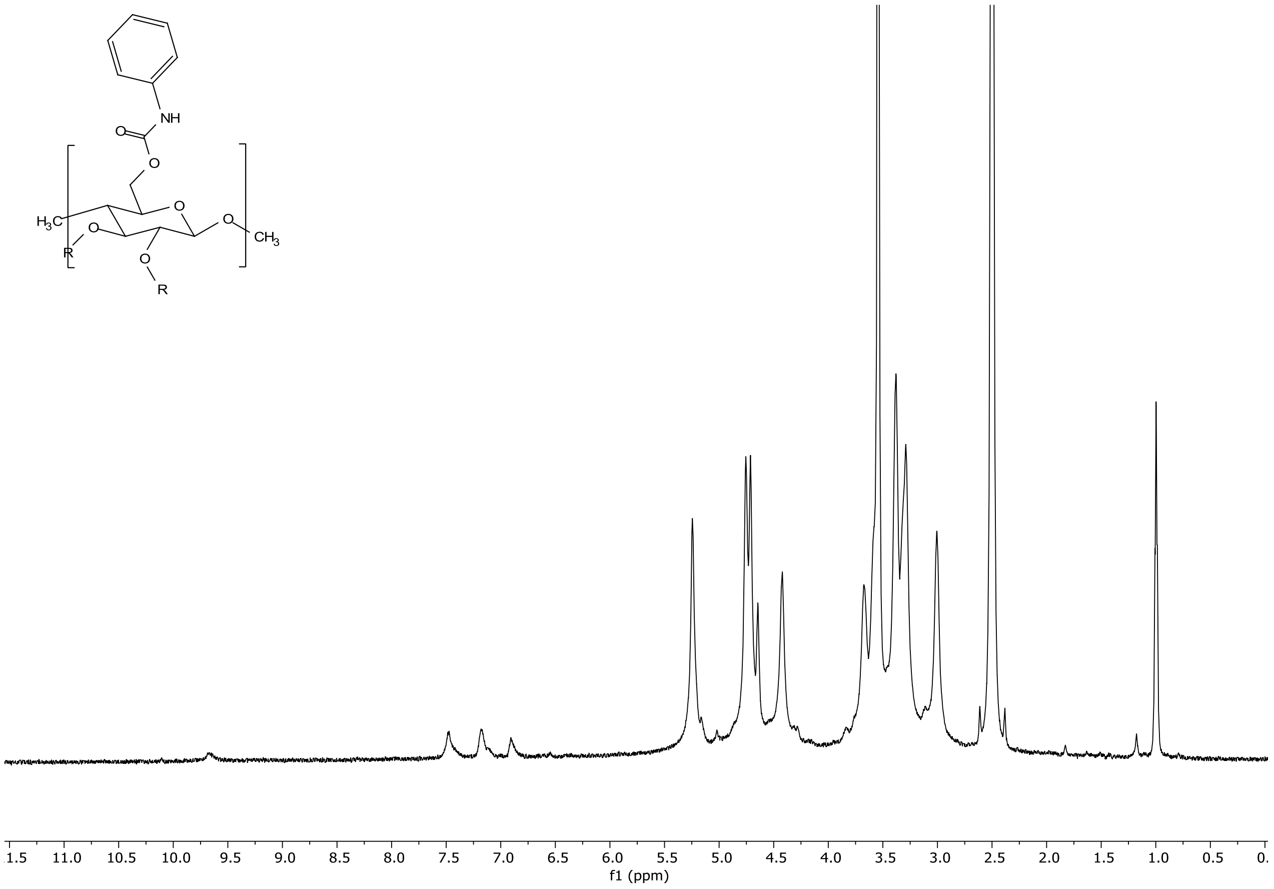

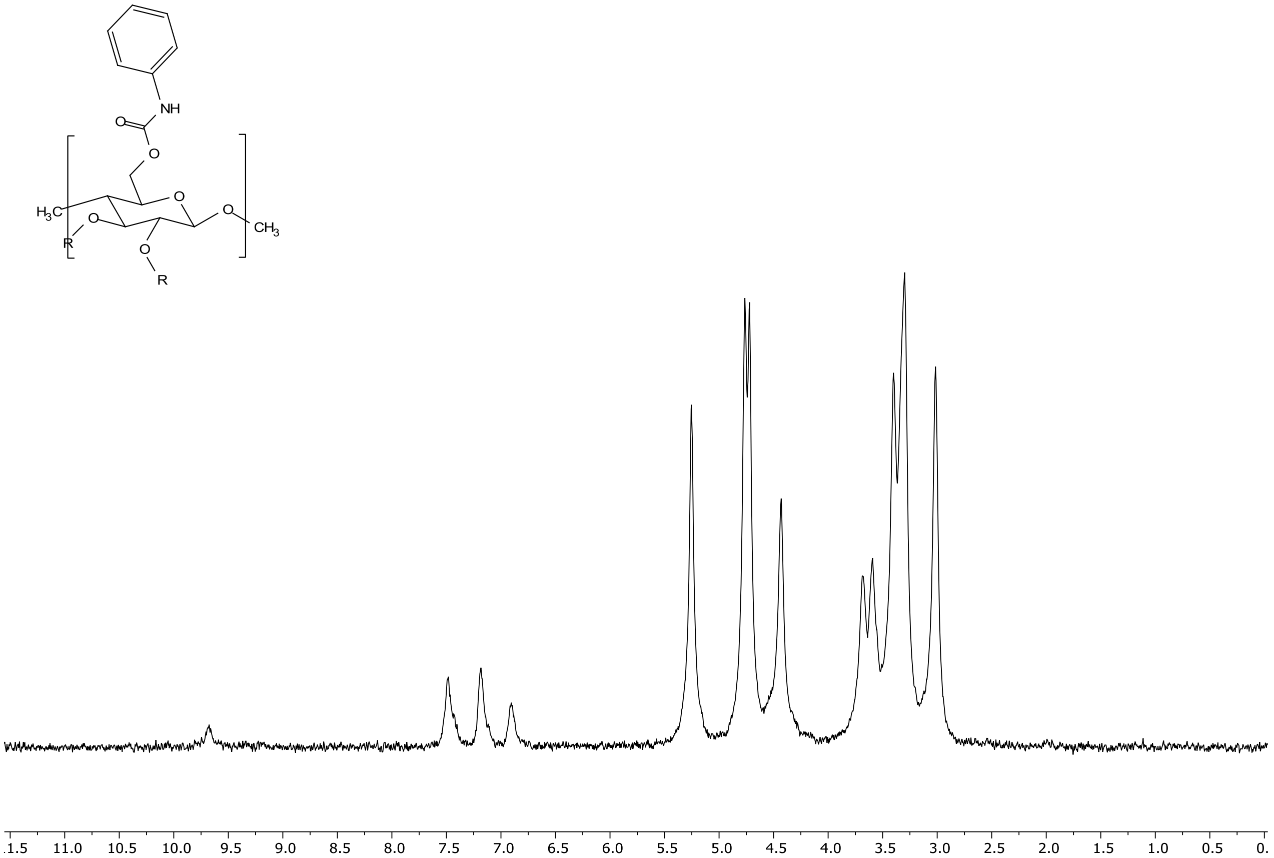

^{1}H and diffusion-edited ^{1}H spectra of **Cellulose *N*-phenyl carbamate DS 0.07** in DMSO-d_6/LiCl solution. (R: H or -CONHPh)

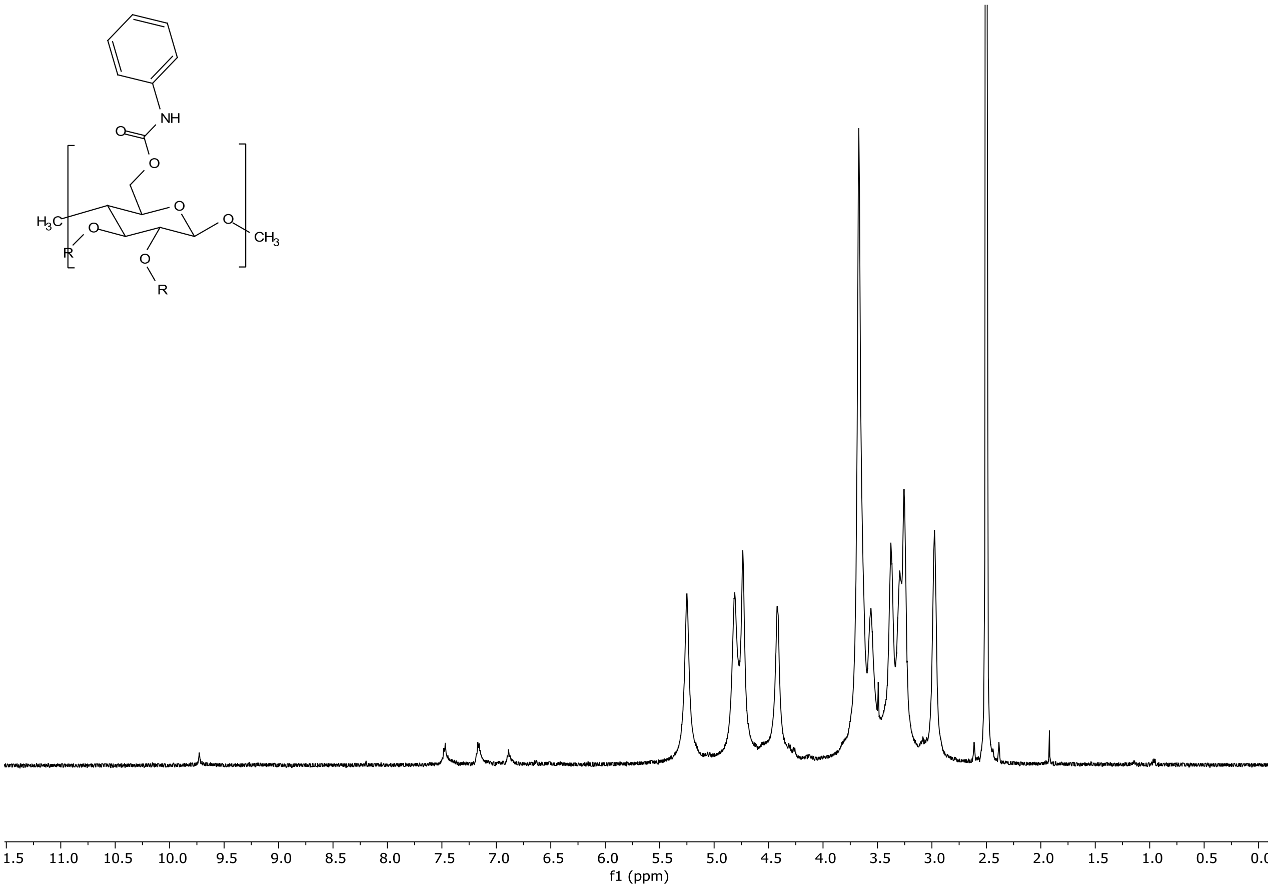

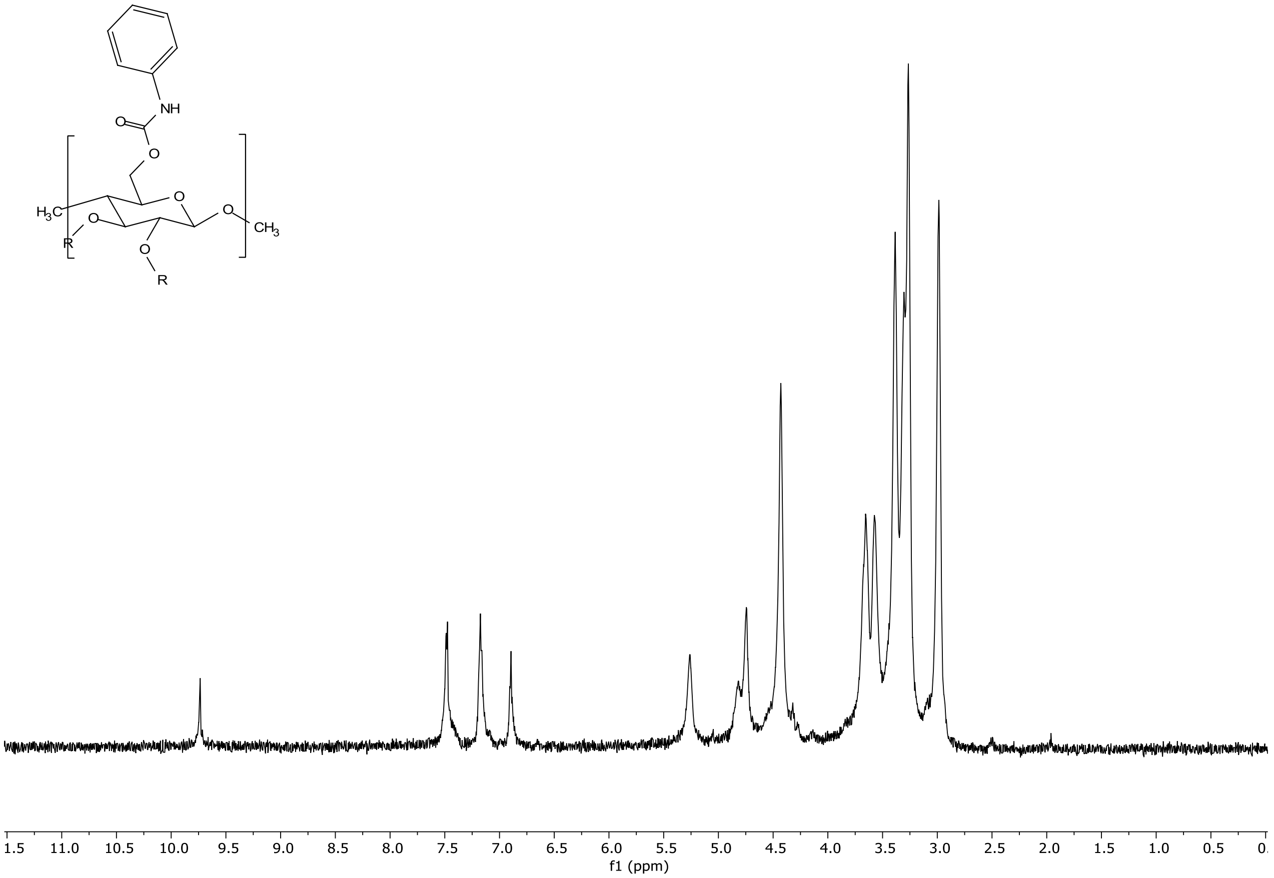

^{1}H and diffusion-edited ^{1}H spectra of **Cellulose *N*-phenyl carbamate DS 0.08** in DMSO-d_6/LiCl solution. (R: H or -CONHPh)

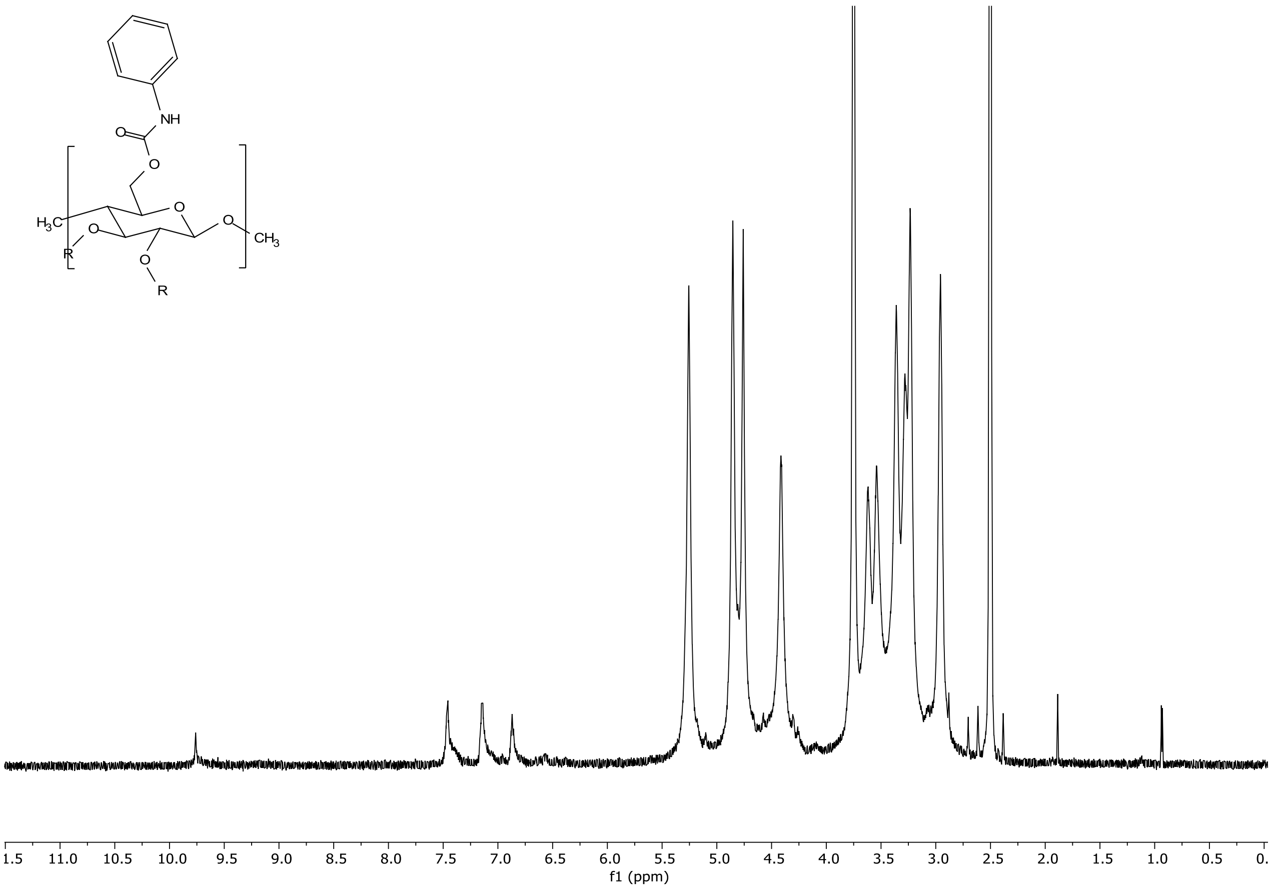

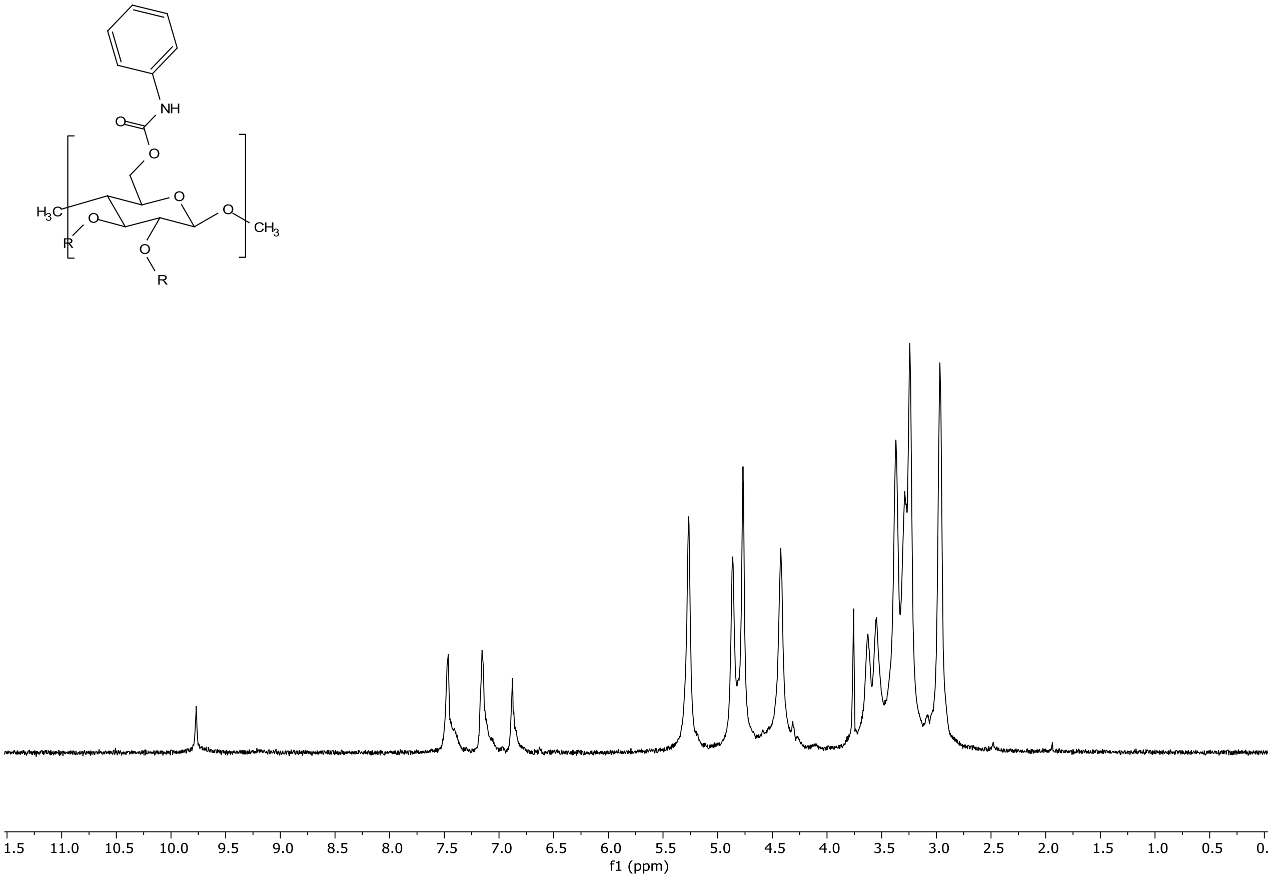

^{1}H and diffusion-edited ^{1}H spectra of **Cellulose *N*-phenyl carbamate DS 0.10** in DMSO-d_6/LiCl solution. (R: H or -CONHPh)

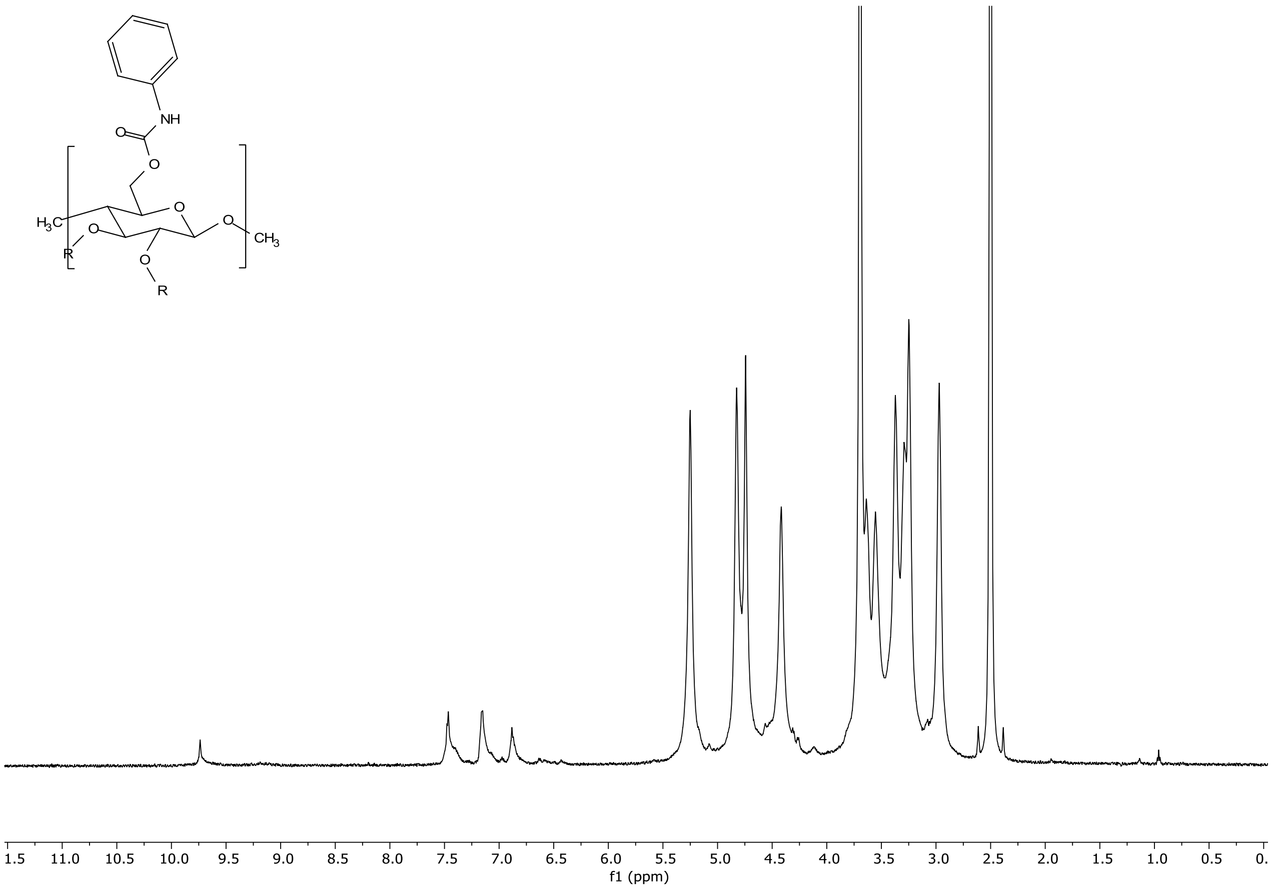

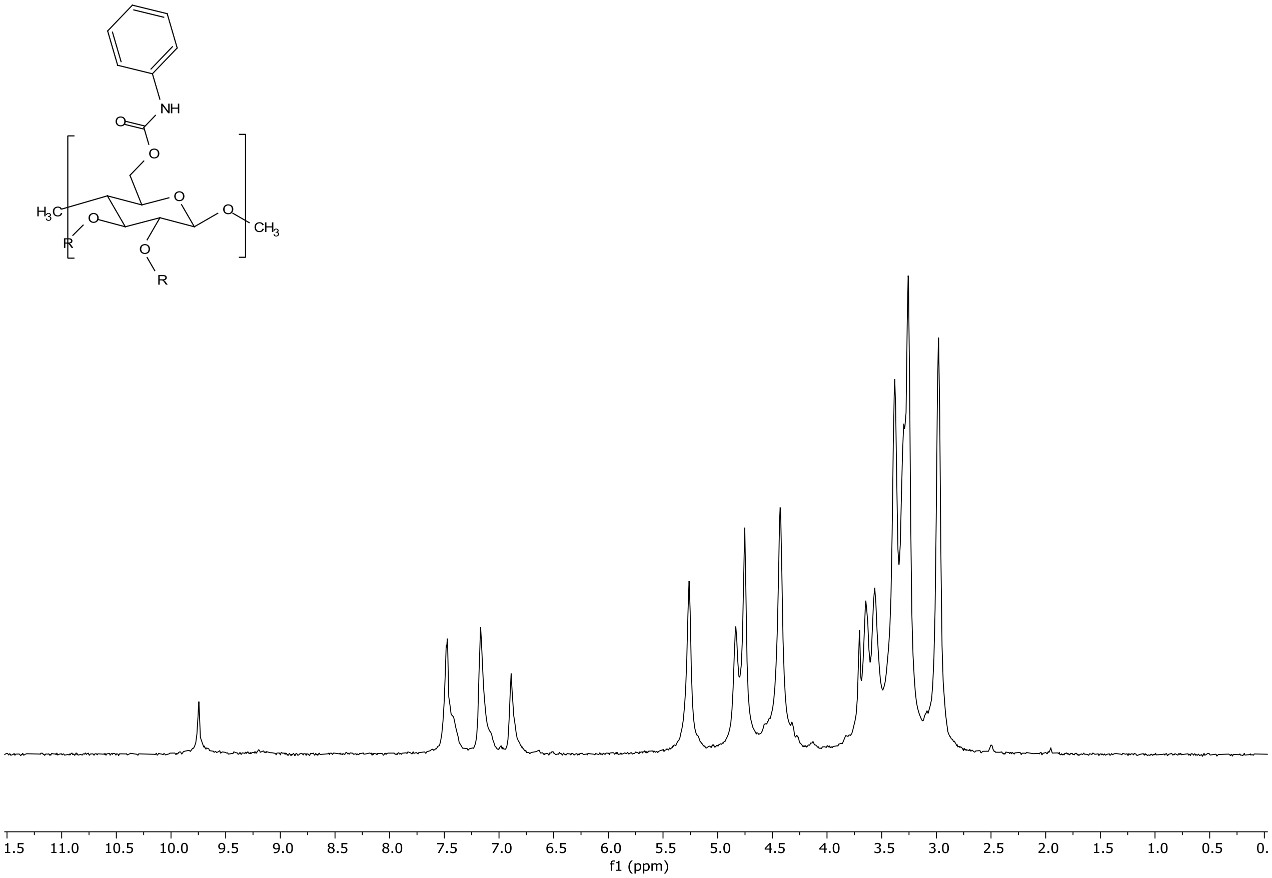

^{1}H and diffusion-edited ^{1}H spectra of **Cellulose *N*-phenyl carbamate DS 0.11** in DMSO-d_6/LiCl solution. (R: H or -CONHPh)

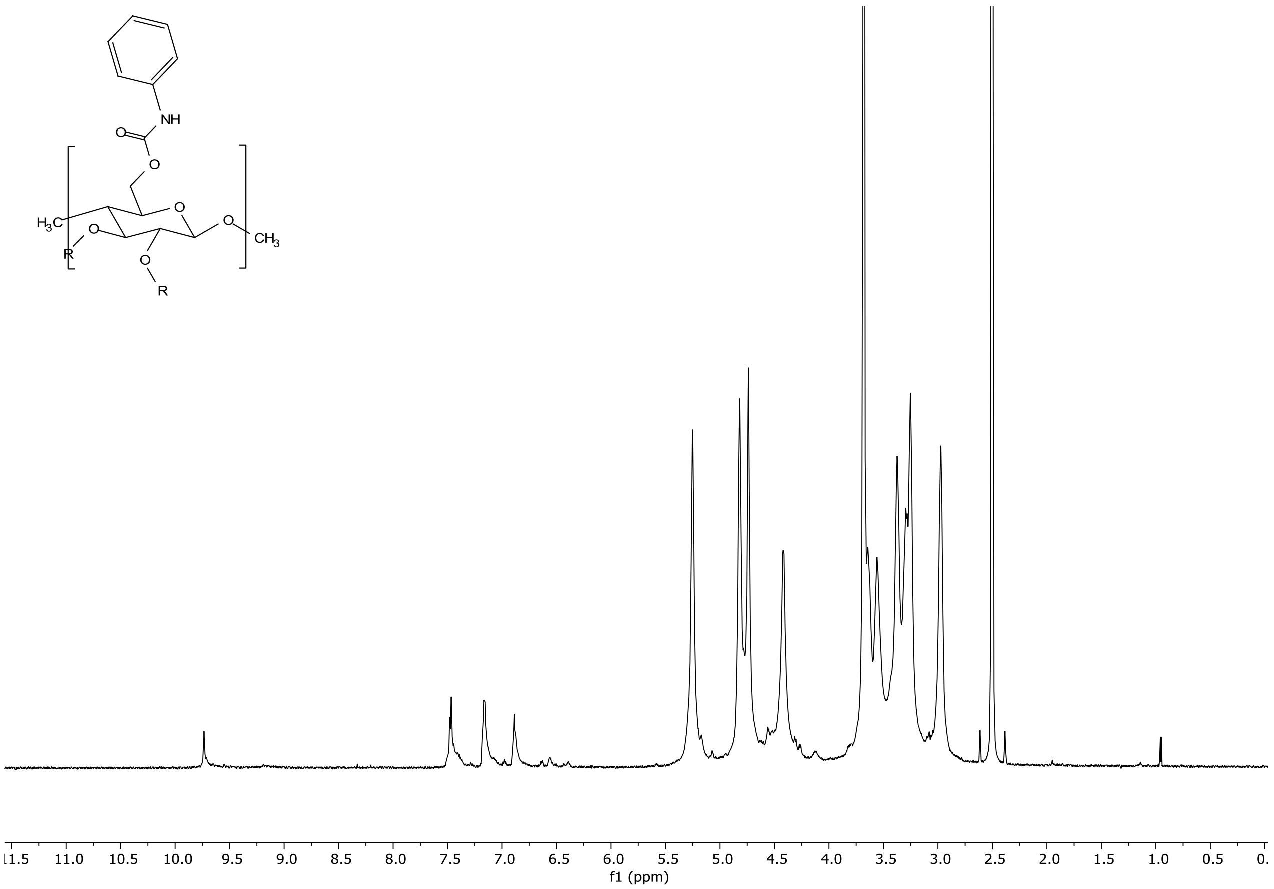

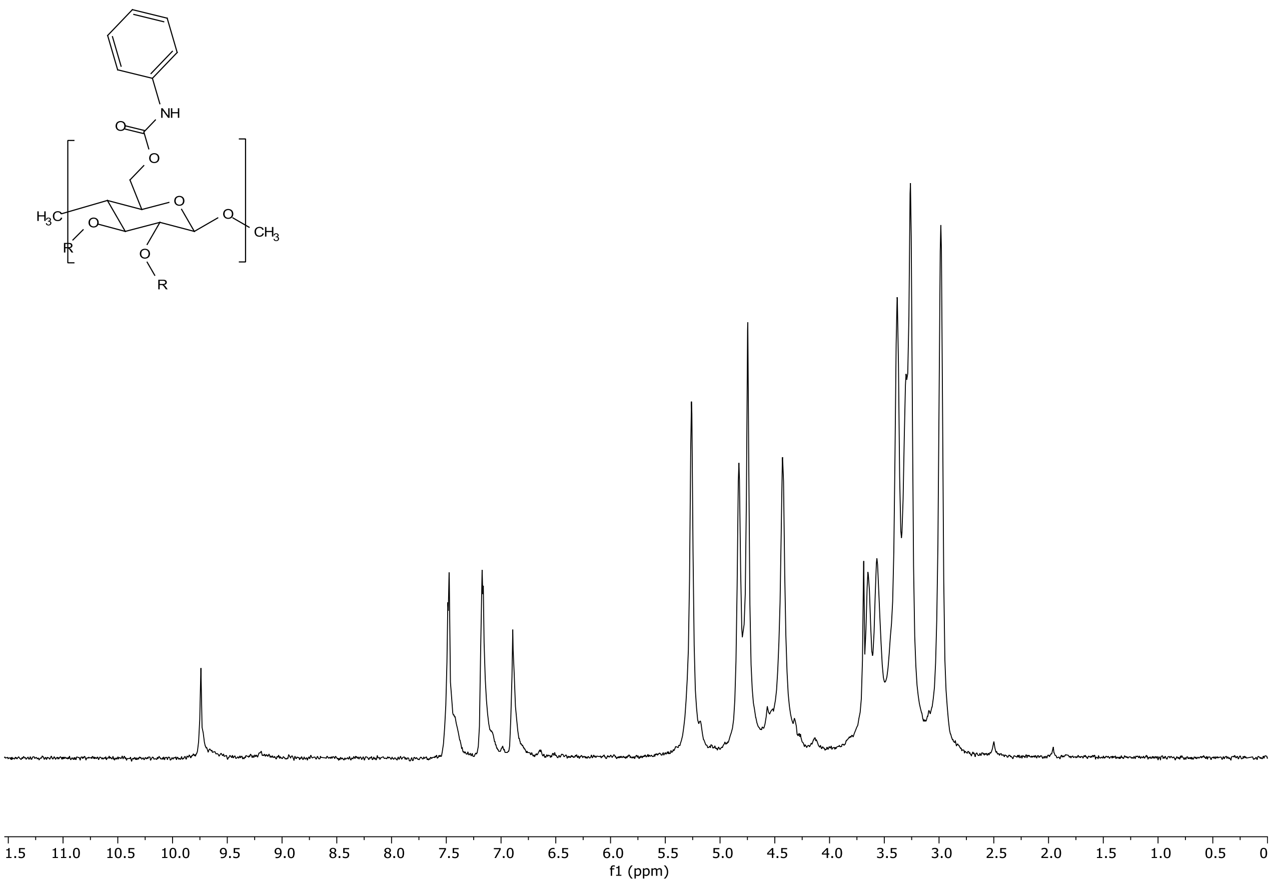

^{1}H and diffusion-edited ^{1}H spectra of **Cellulose *N*-phenyl carbamate DS 0.14** in DMSO-d_6/LiCl solution. (R: H or -CONHPh)

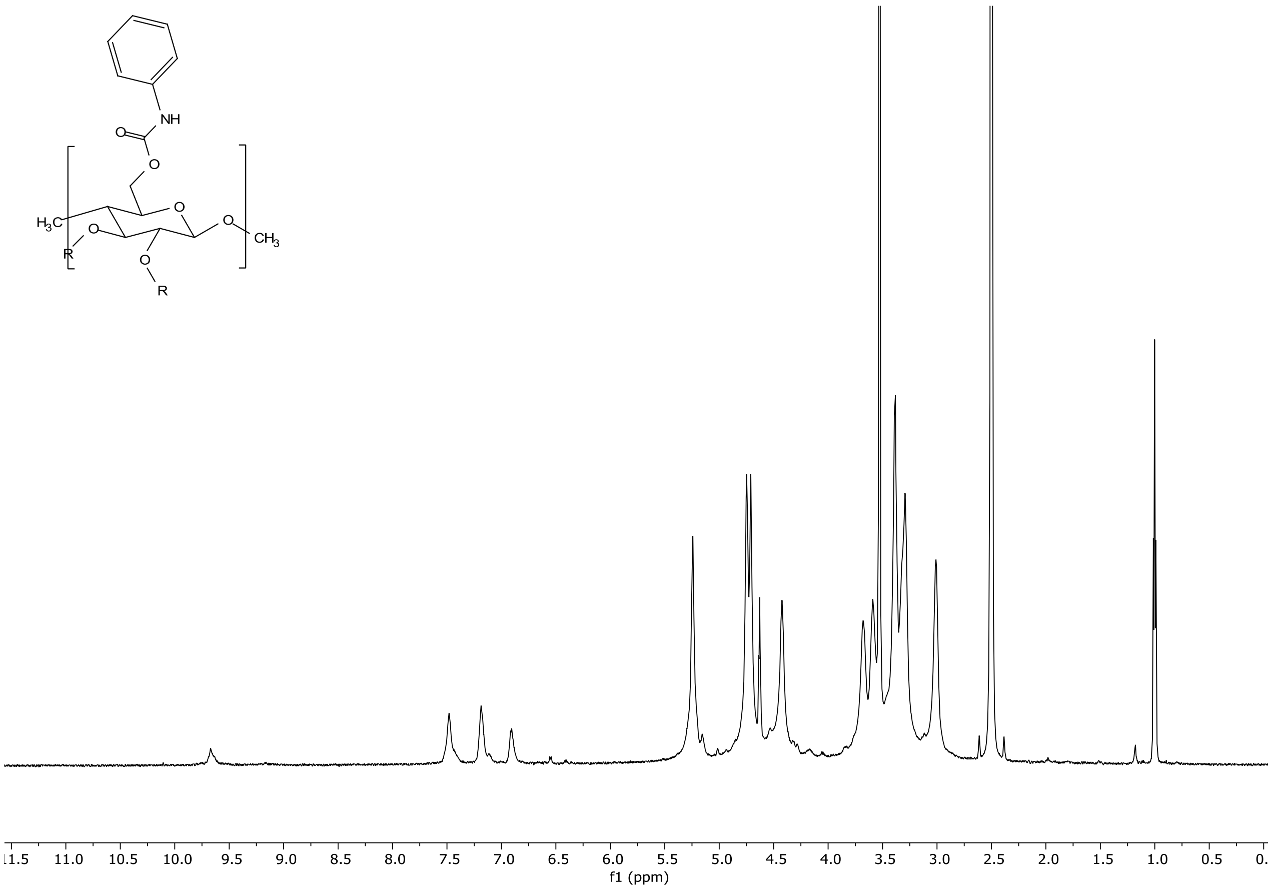

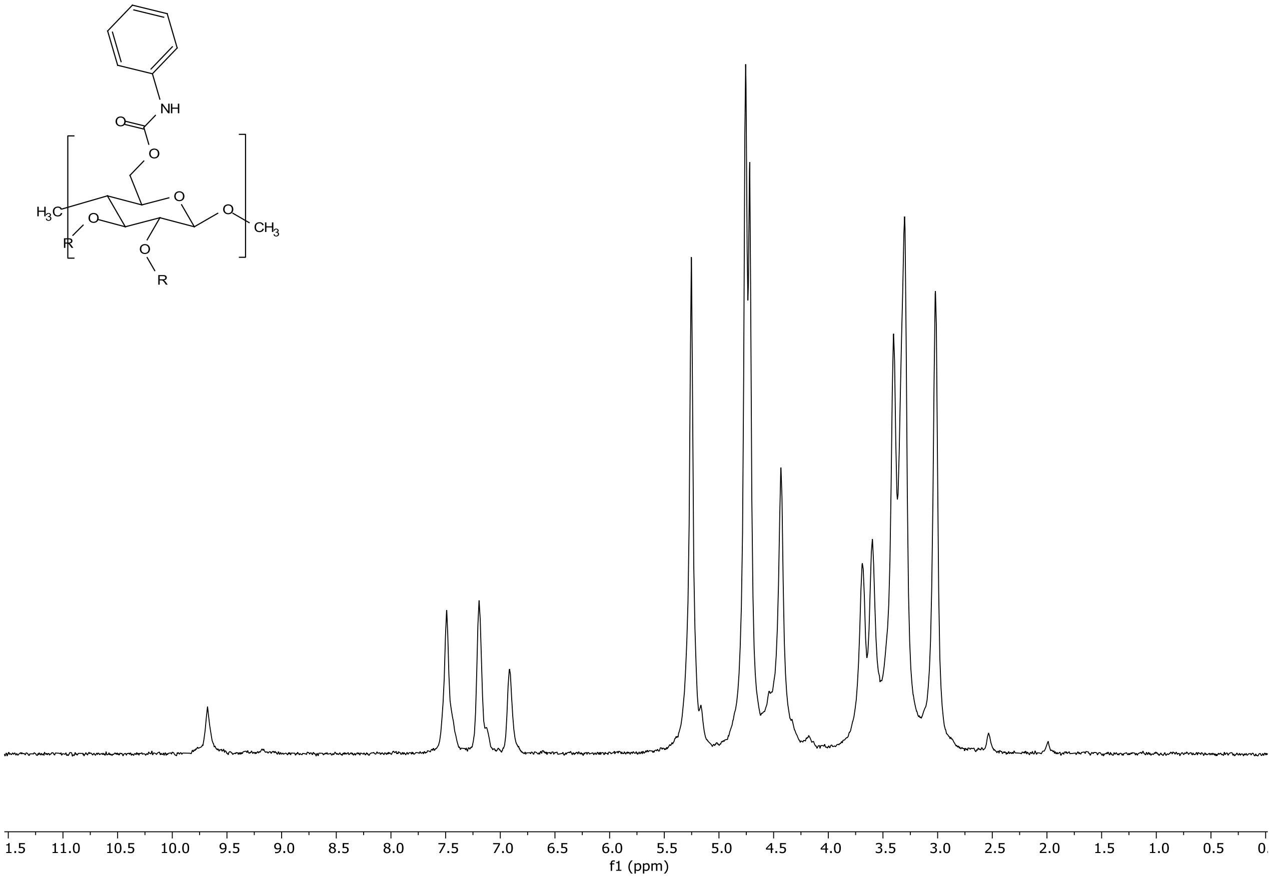

^{1}H and diffusion-edited ^{1}H spectra of **Cellulose *N*-phenyl carbamate DS 0.12** in DMSO-d_6/LiCl solution. (R: H or -CONHPh)

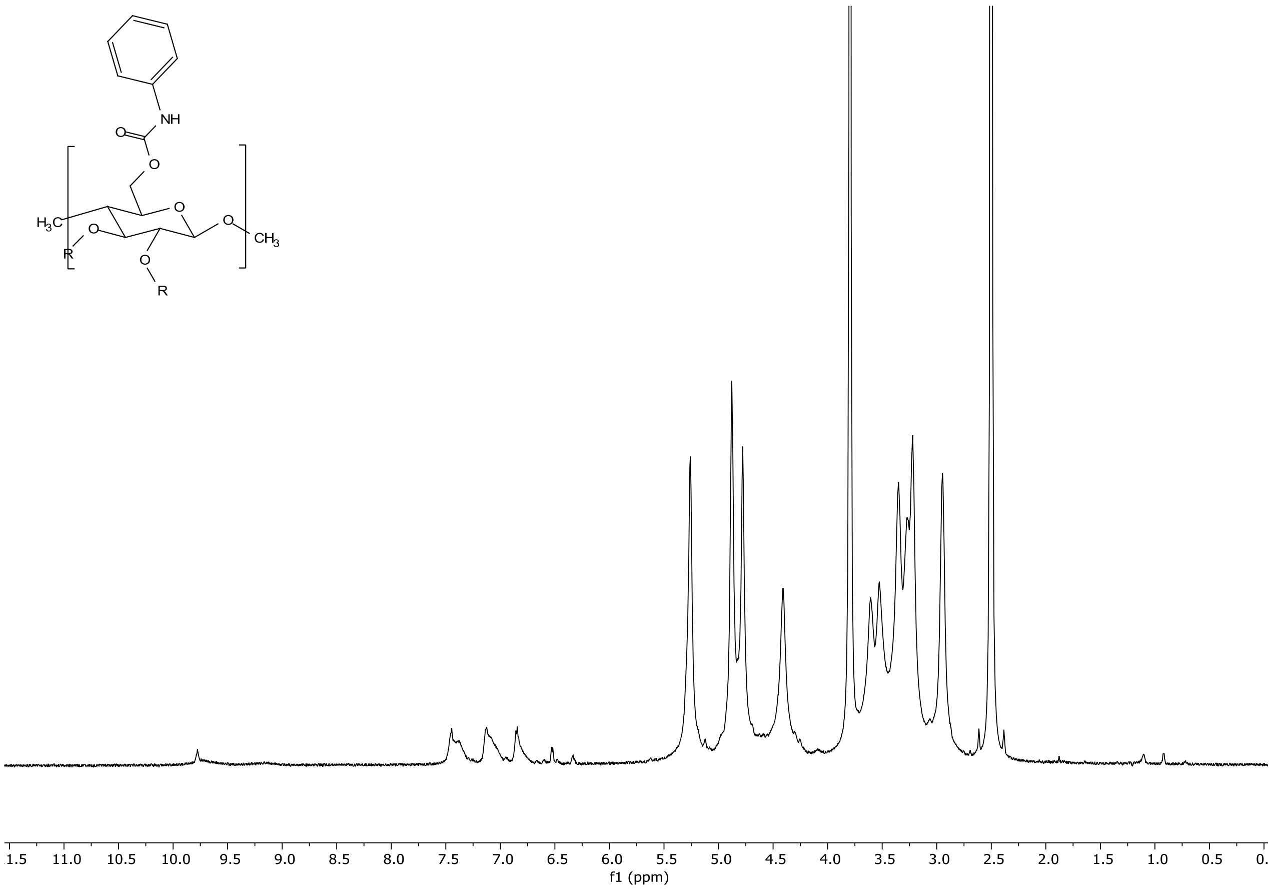

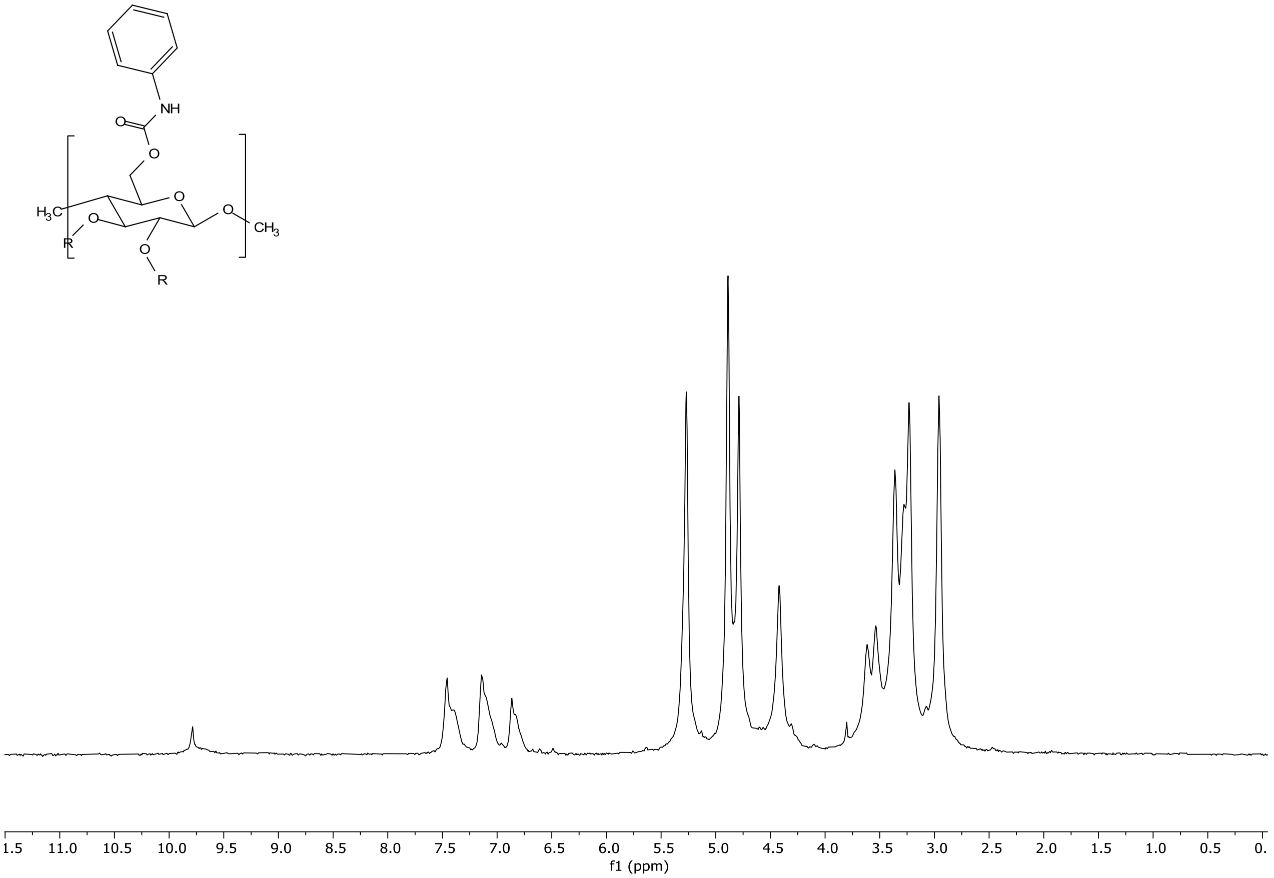

^{1}H and diffusion-edited ^{1}H spectra of **Cellulose *N*-phenyl carbamate DS 0.27** in DMSO-d_6/LiCl solution. (R: H or -CONHPh)

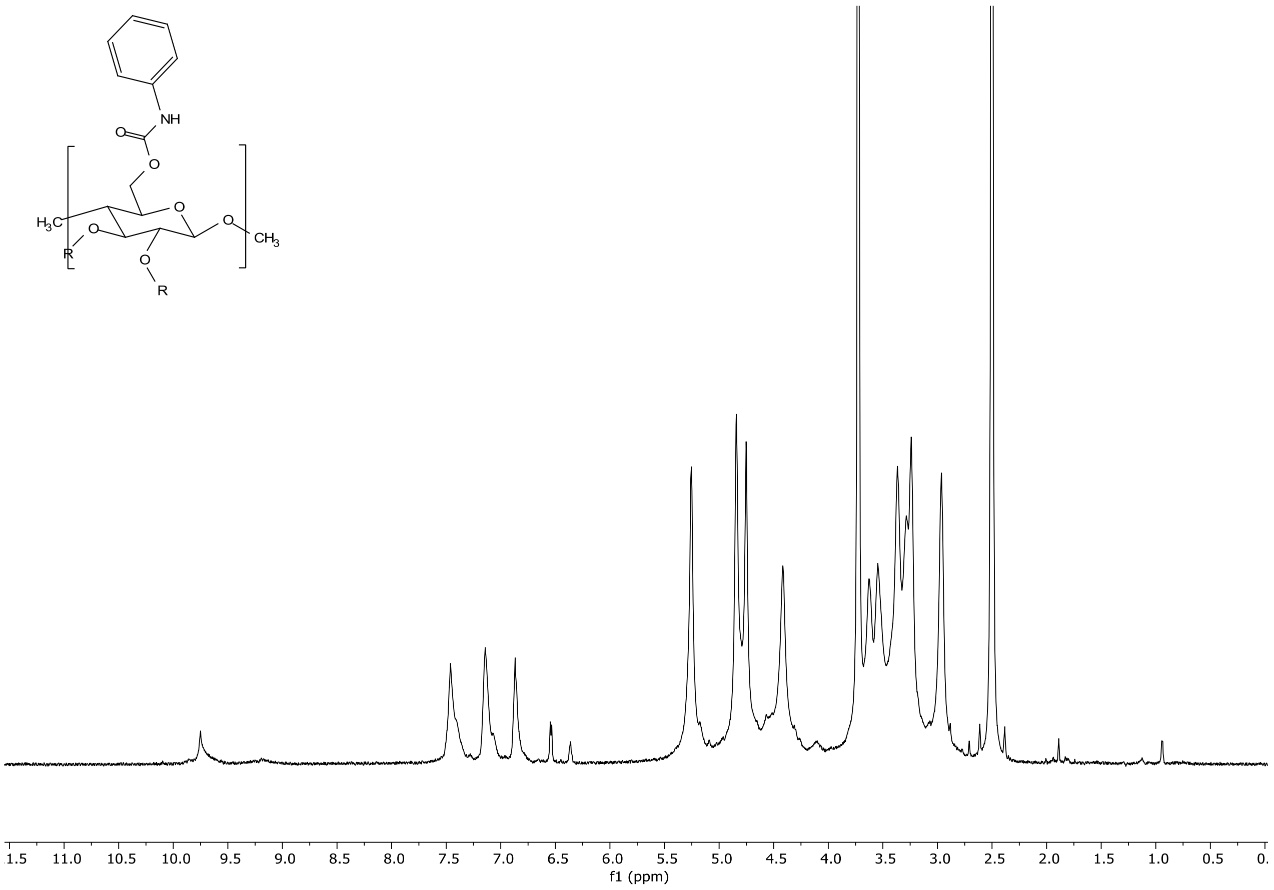

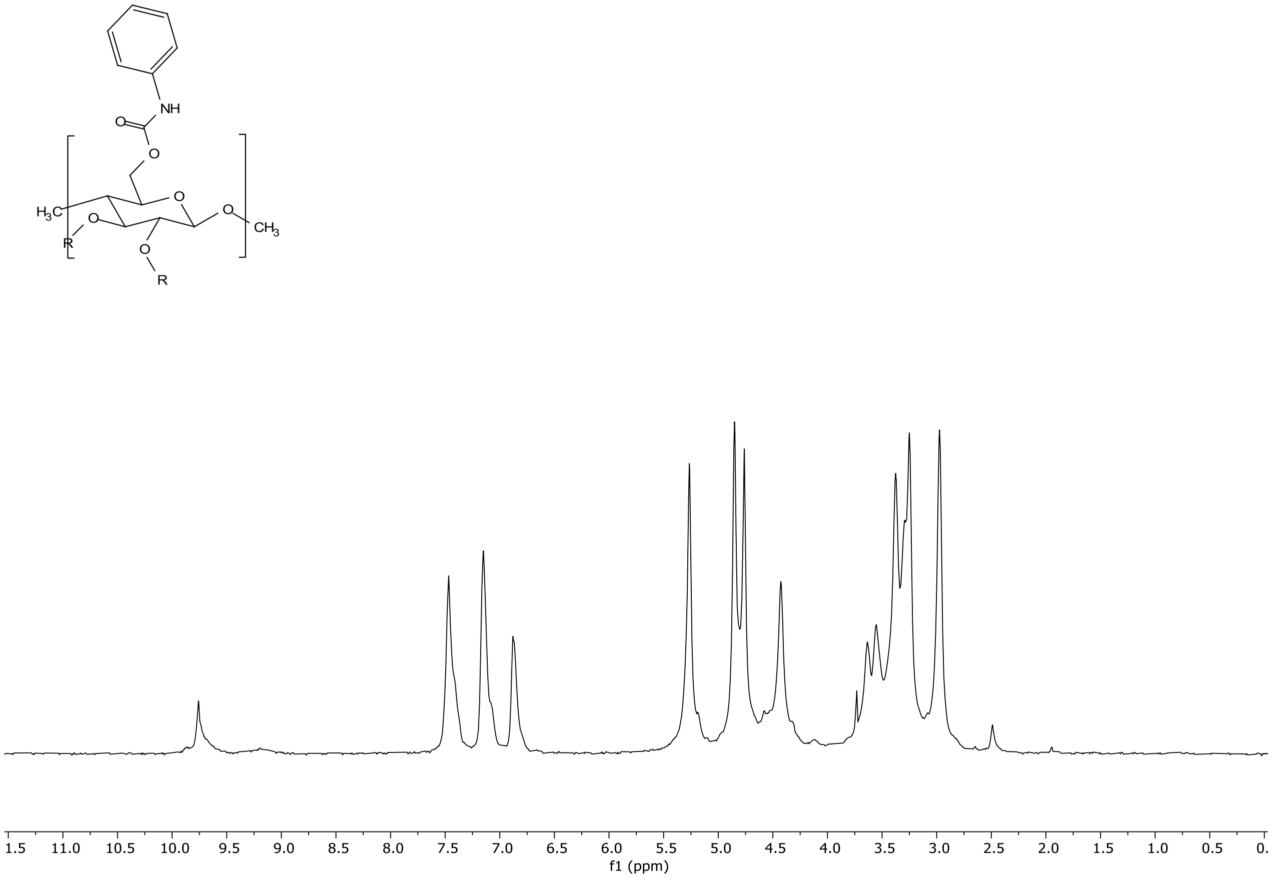

^{1}H and diffusion-edited ^{1}H spectra of **Cellulose *N*-phenyl carbamate DS 0.29** in DMSO-d_6/LiCl solution. (R: H or -CONHPh)

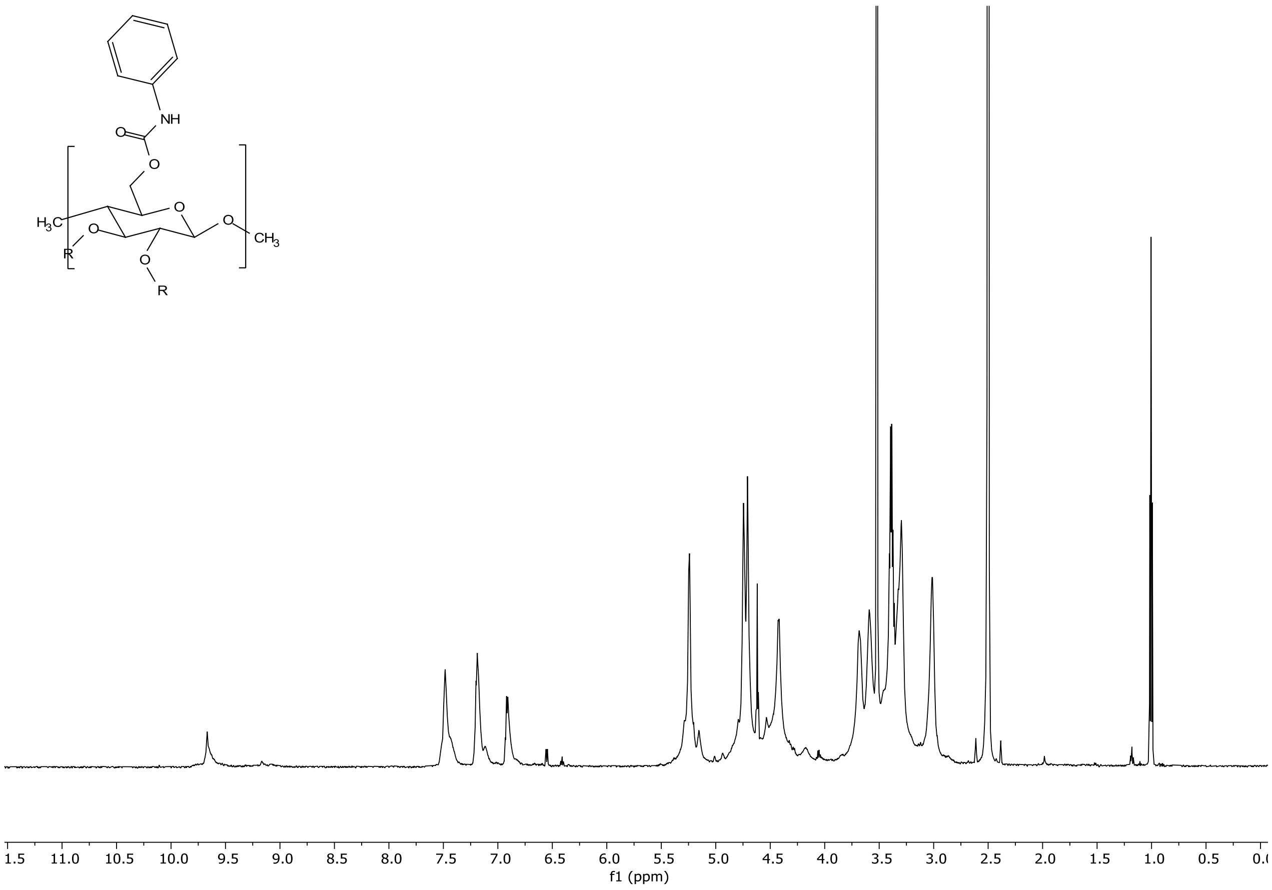

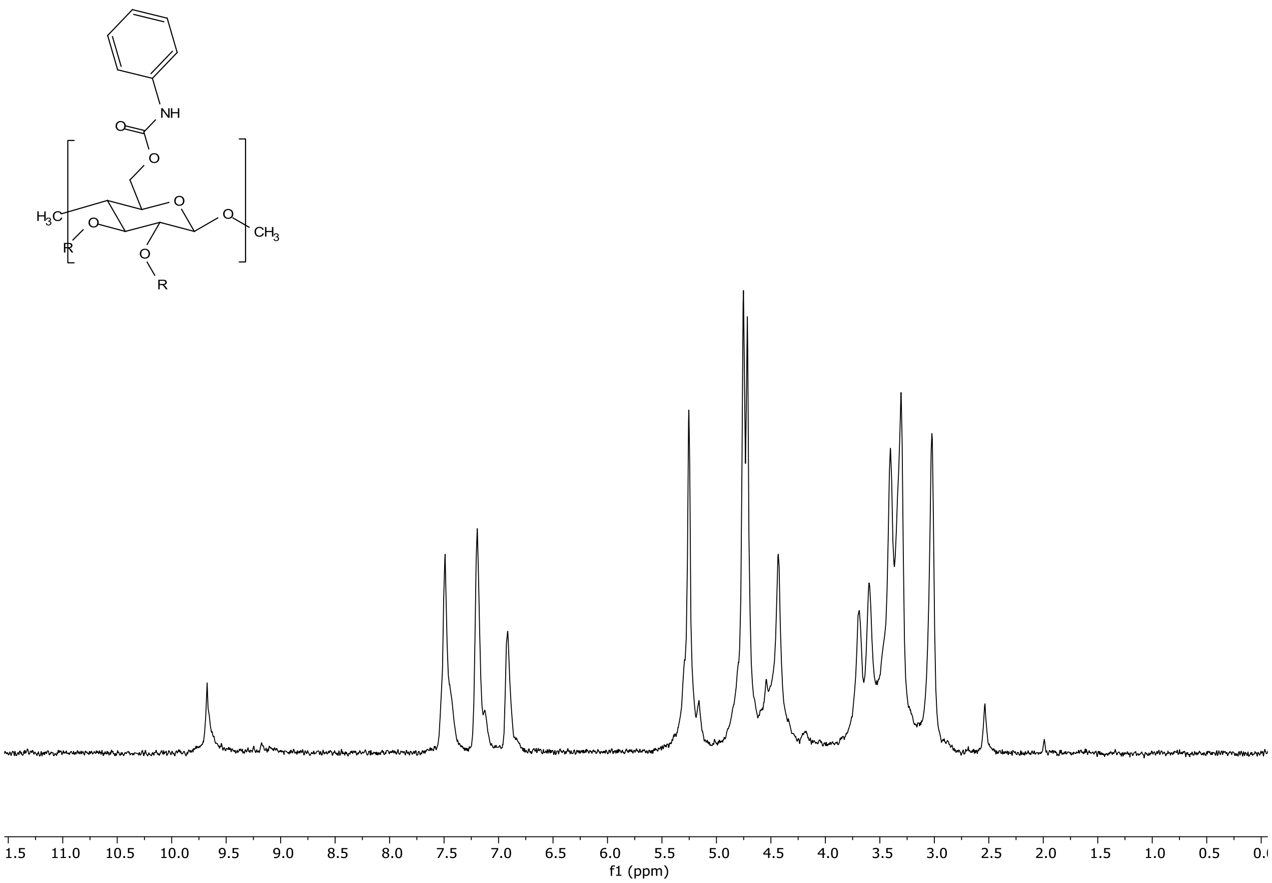

^{1}H and diffusion-edited ^{1}H spectra of **Cellulose *N*-phenyl carbamate DS 0.51** in DMSO-d_6/LiCl solution. (R: H or -CONHPh)

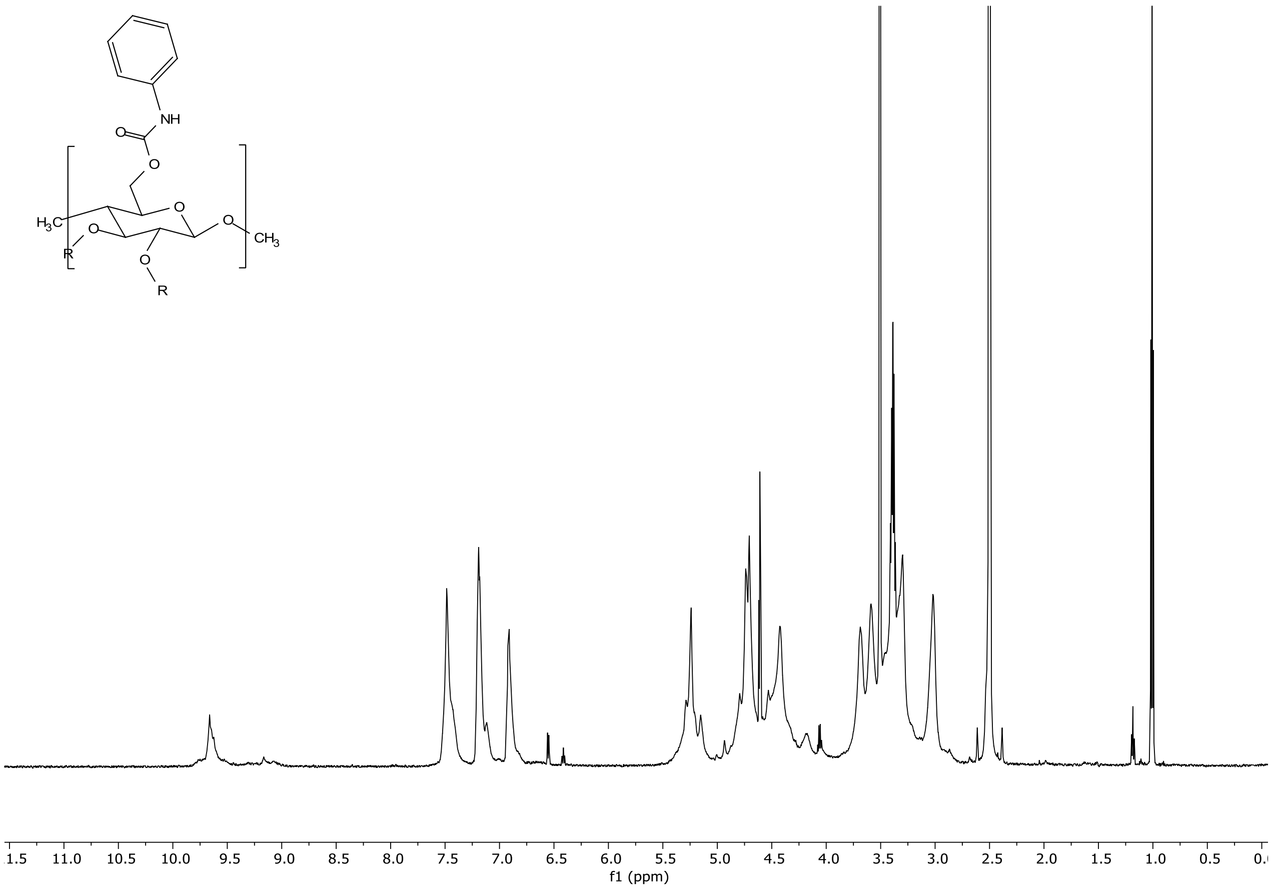

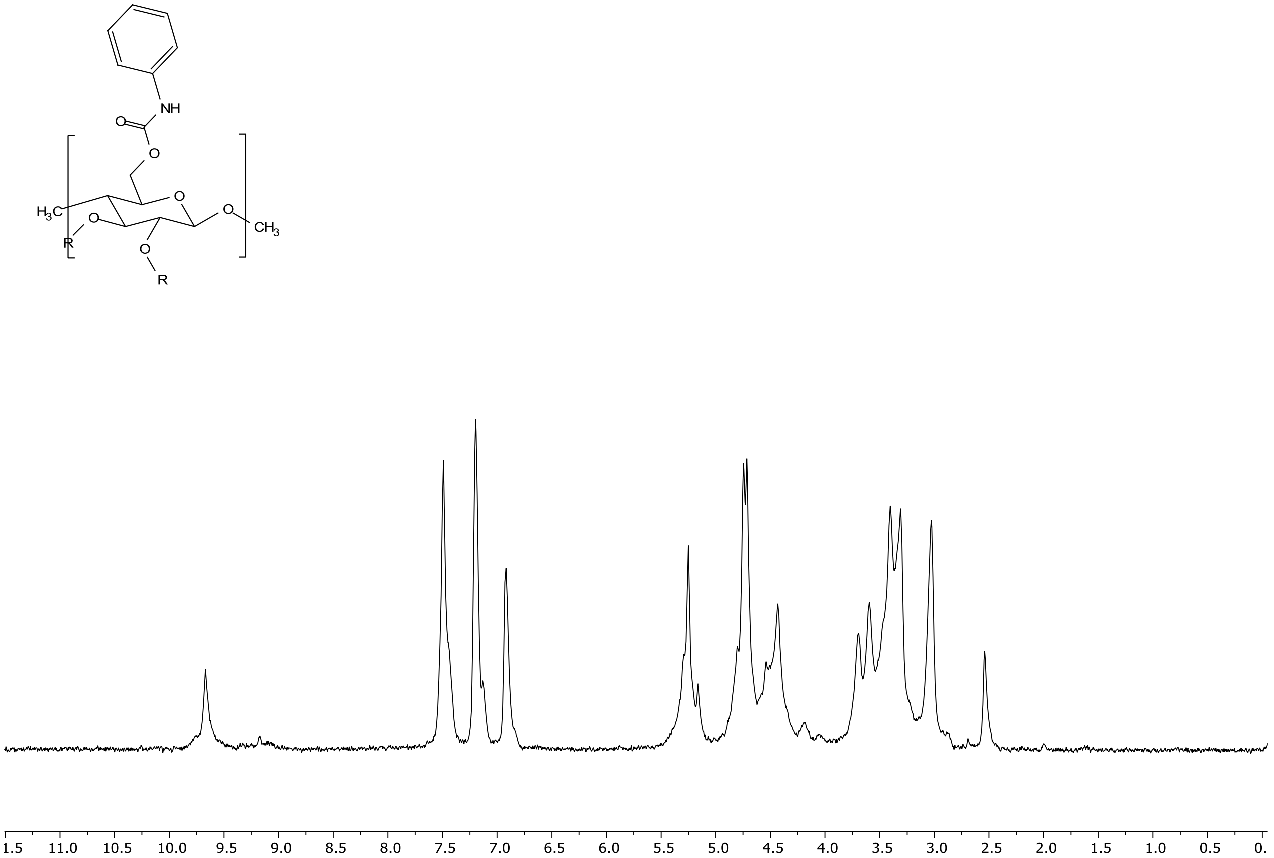

^1H and diffusion-edited ^1H spectra of **Cellulose *N*-phenyl carbamate DS 0.65** in DMSO-d_6/LiCl solution. (R: H or -CONHPh)

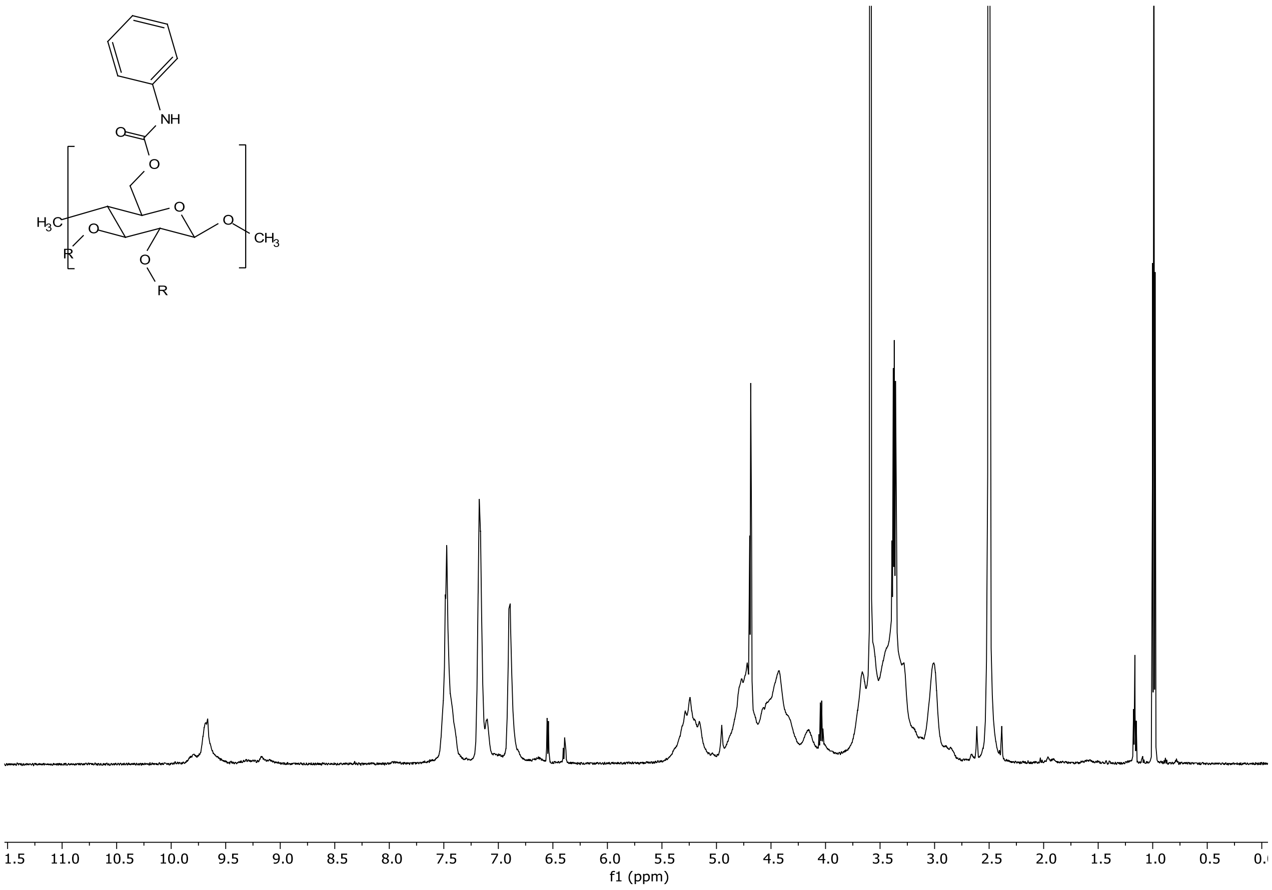

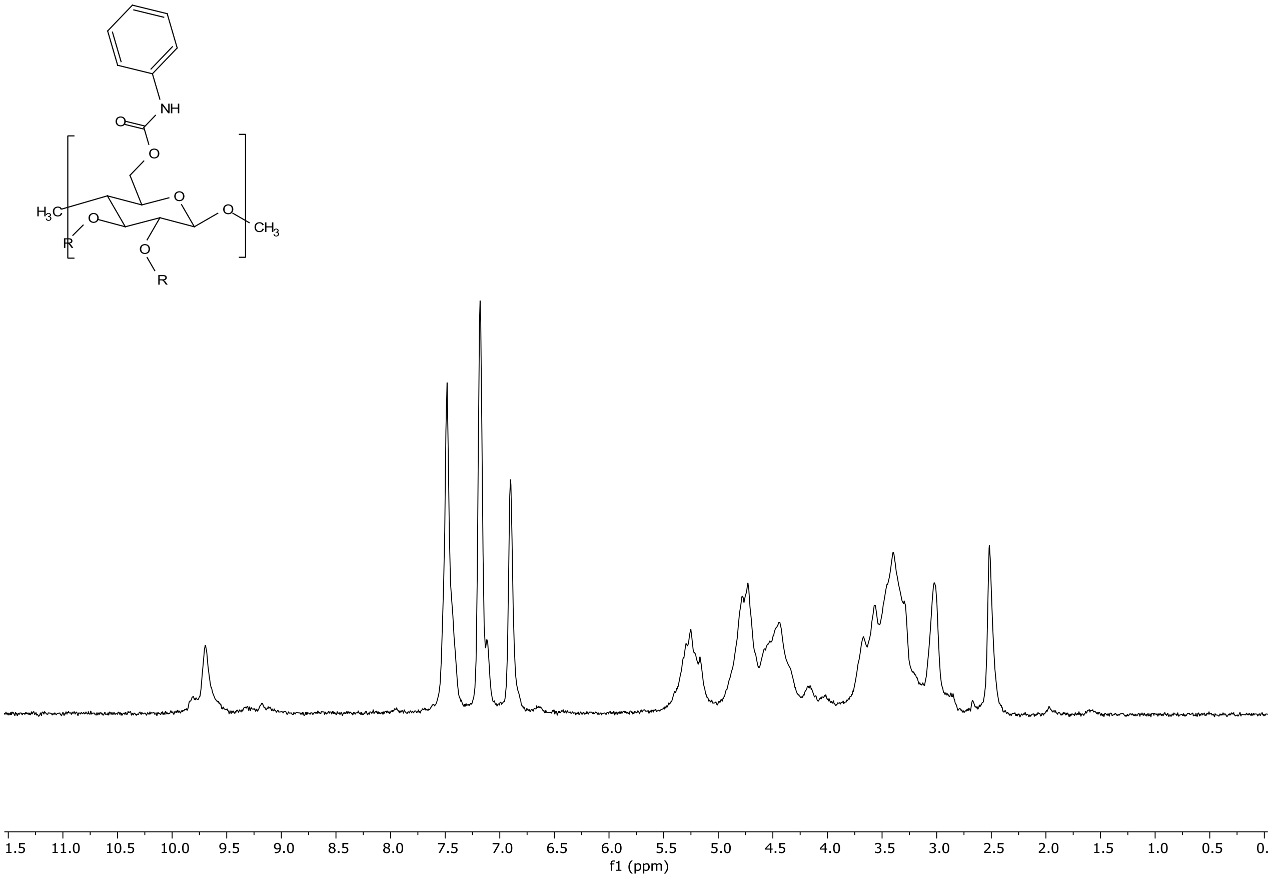

^{1}H and diffusion-edited ^{1}H spectra of **Cellulose *N*-butyl carbamate DS 0.04** in DMSO-d_6/LiCl solution. (R: H or $-CONHCH_2CH_2CH_2CH_3$)

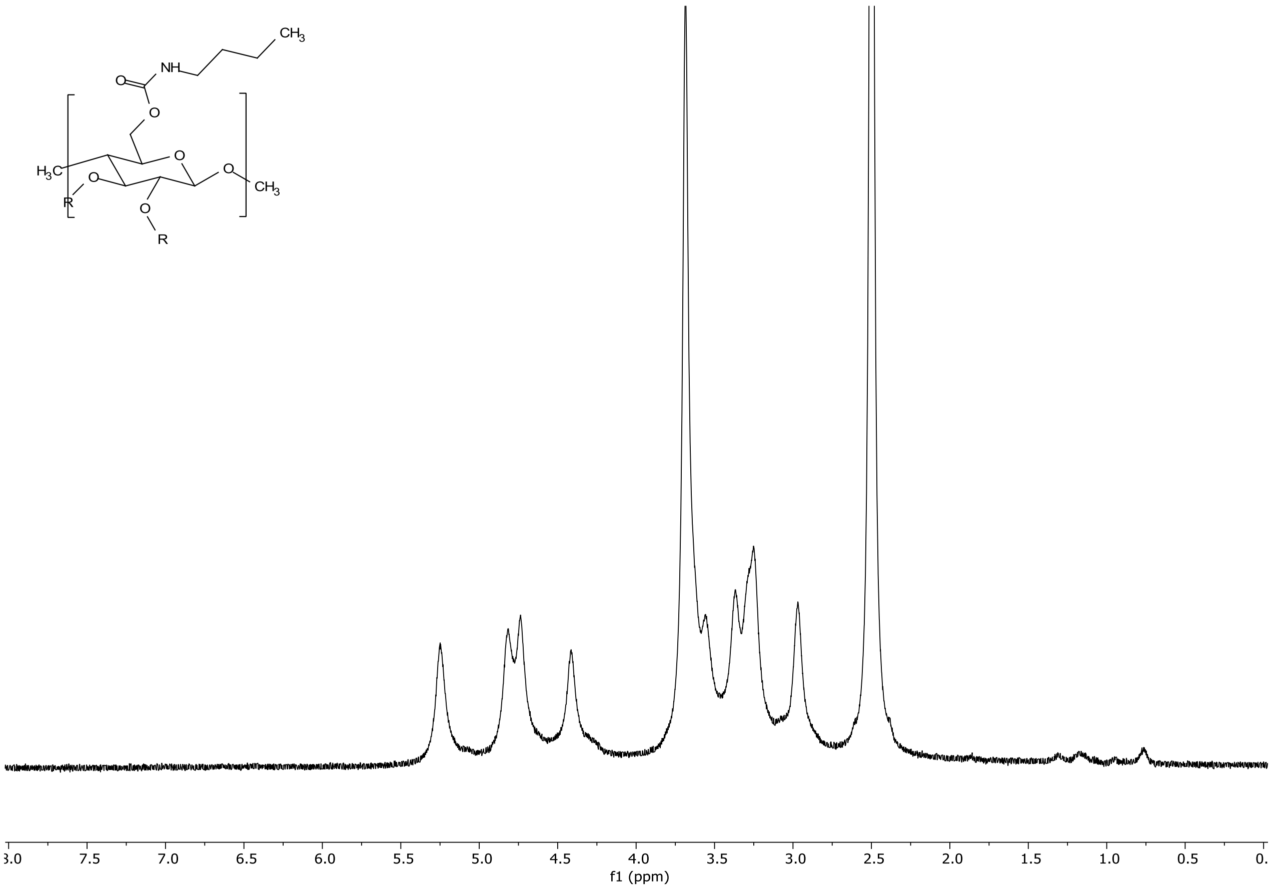

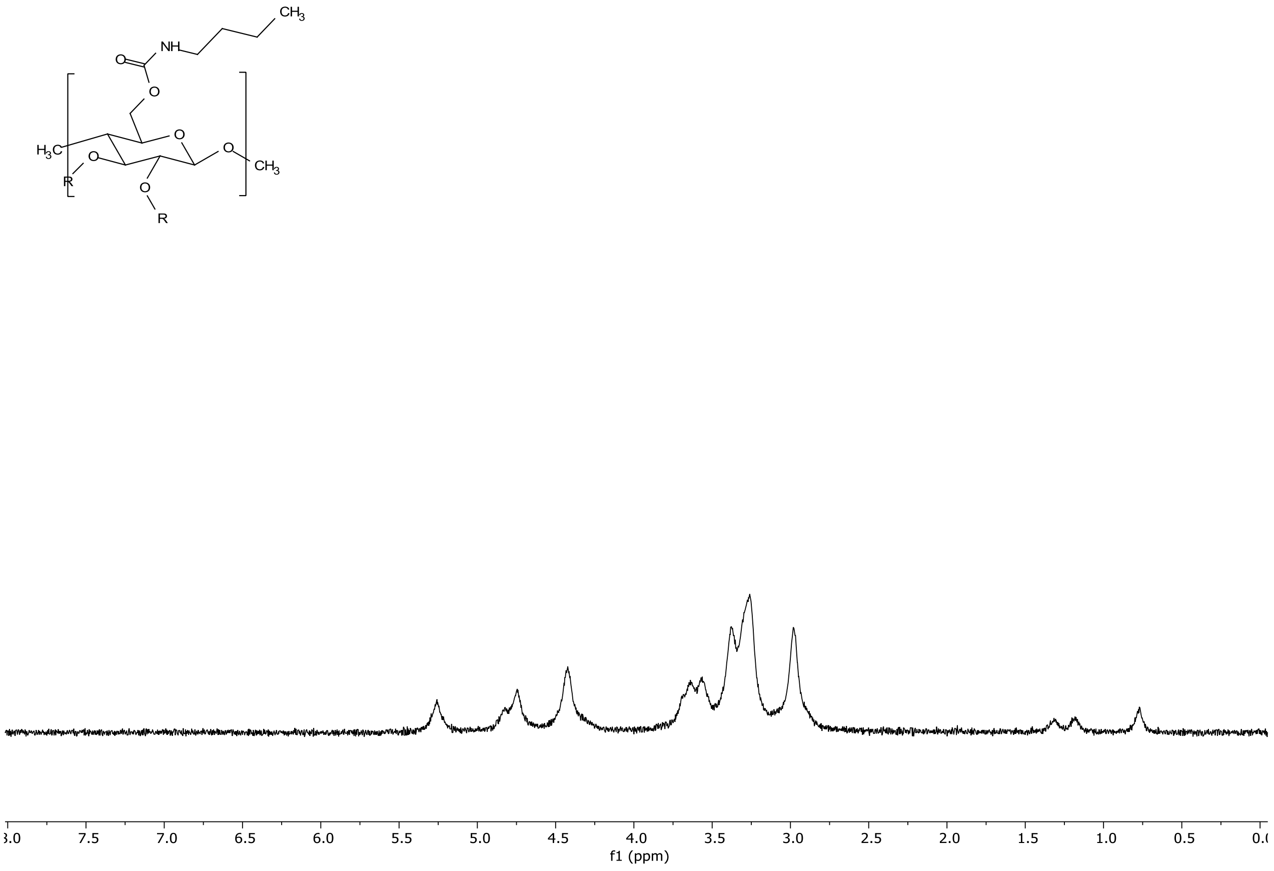

^1H and diffusion-edited ^1H spectra of **Cellulose *N*-butyl carbamate DS 0.11** in DMSO-d_6/LiCl solution. (R: H or $-CONHCH_2CH_2CH_2CH_3$)

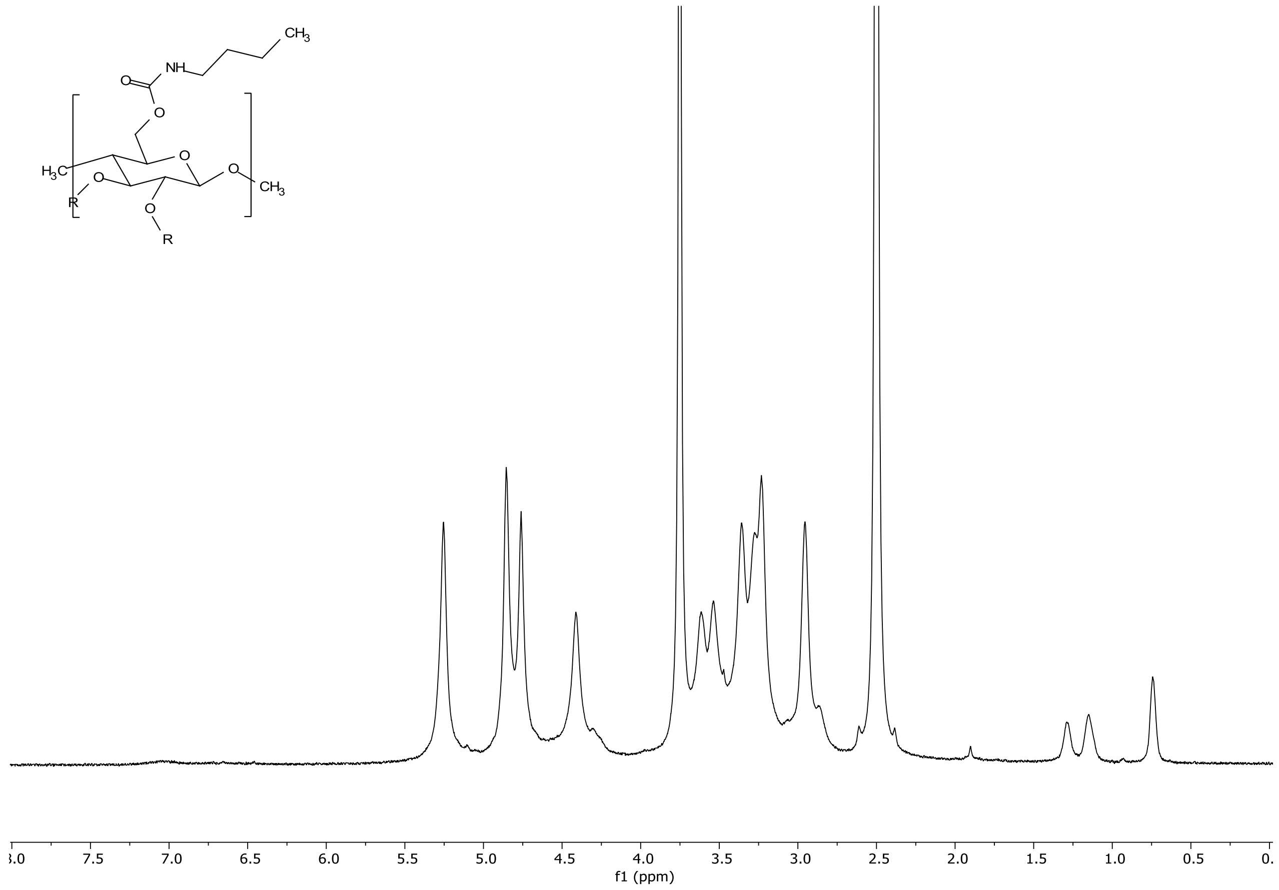

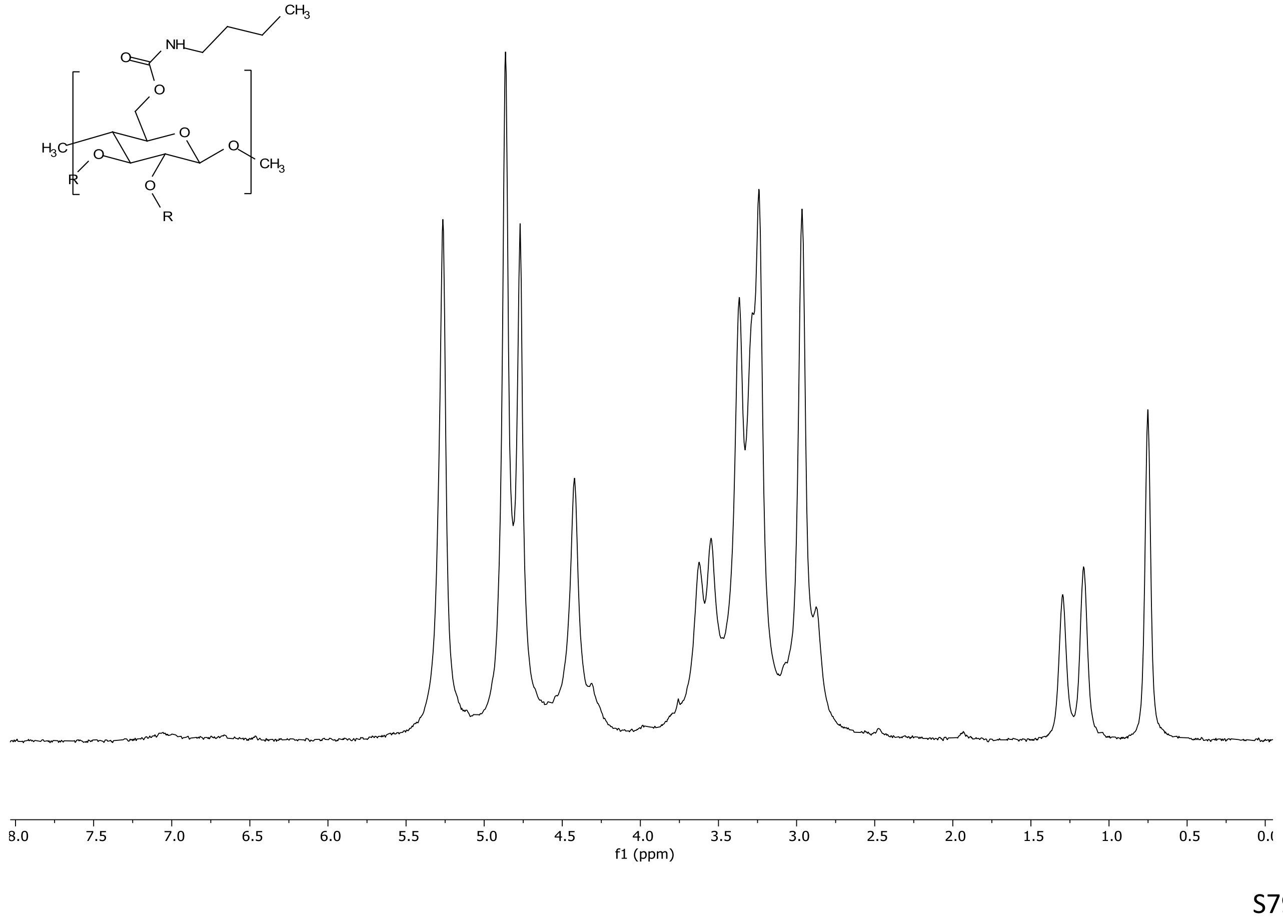

^{1}H and diffusion-edited ^{1}H spectra of **Cellulose *N*-butyl carbamate DS 0.14** in DMSO-d_6/LiCl solution. (R: H or $-CONHCH_2CH_2CH_2CH_3$)

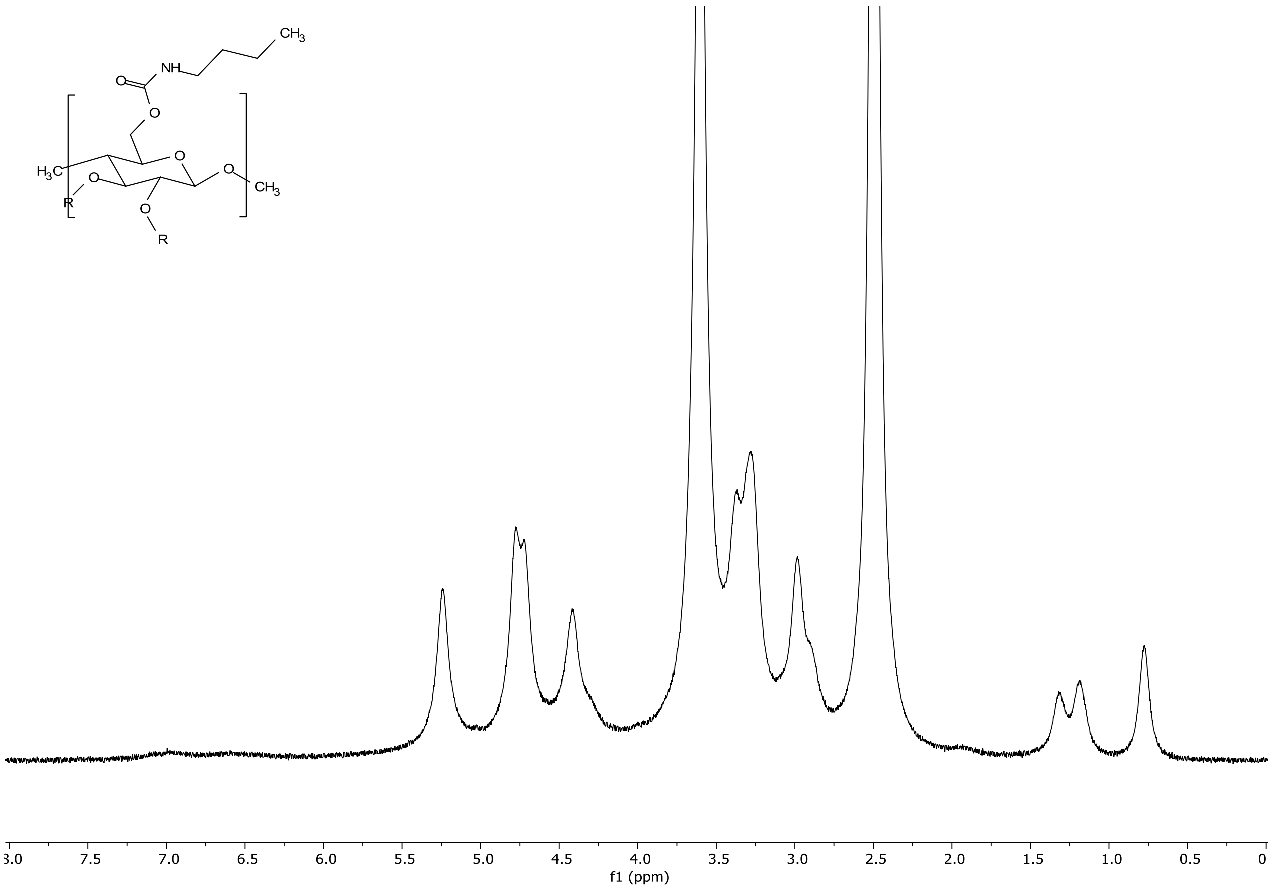

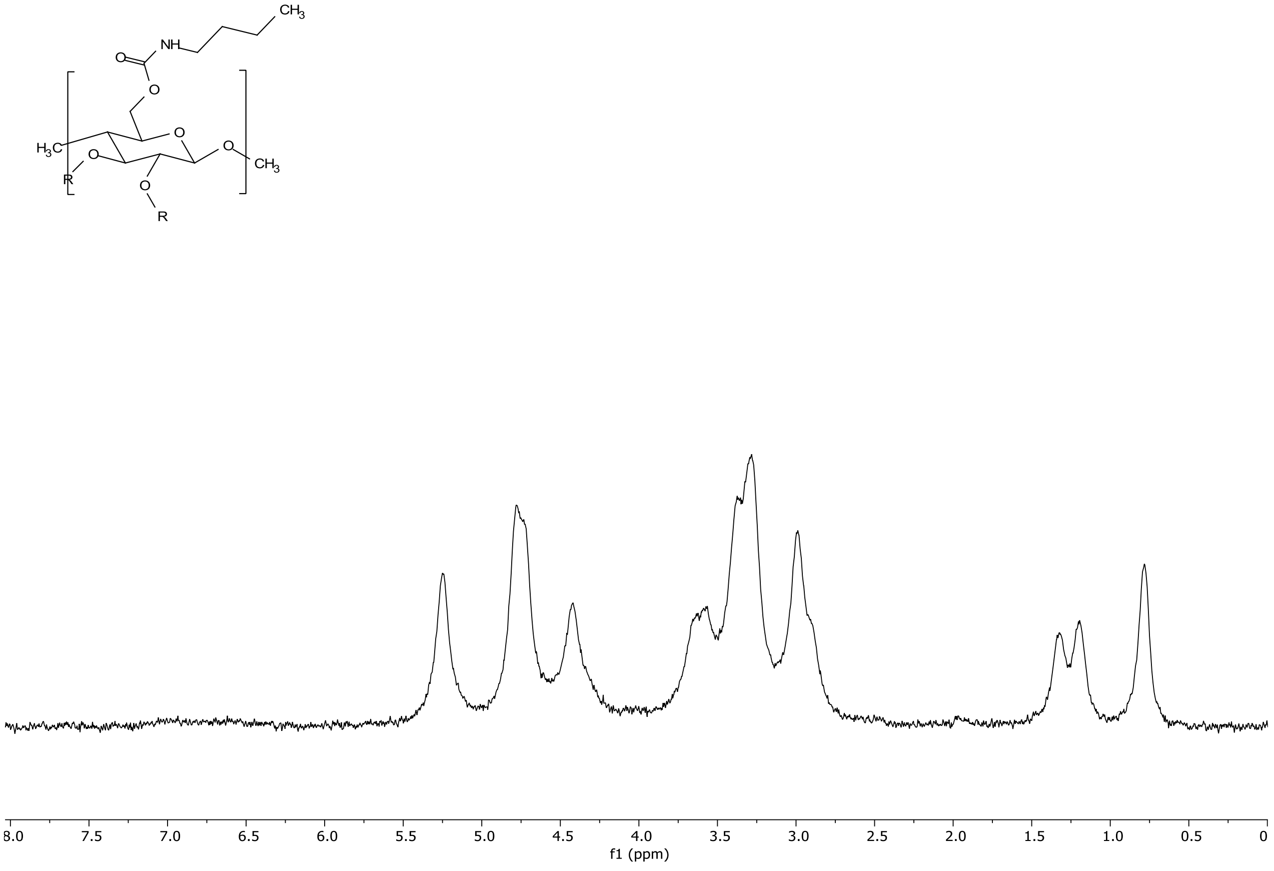

^{1}H and diffusion-edited ^{1}H spectra of **Cellulose *N*-butyl carbamate DS 0.14** in DMSO-*d_6*/LiCl solution. (R: H or $-CONHCH_2CH_2CH_2CH_3$)

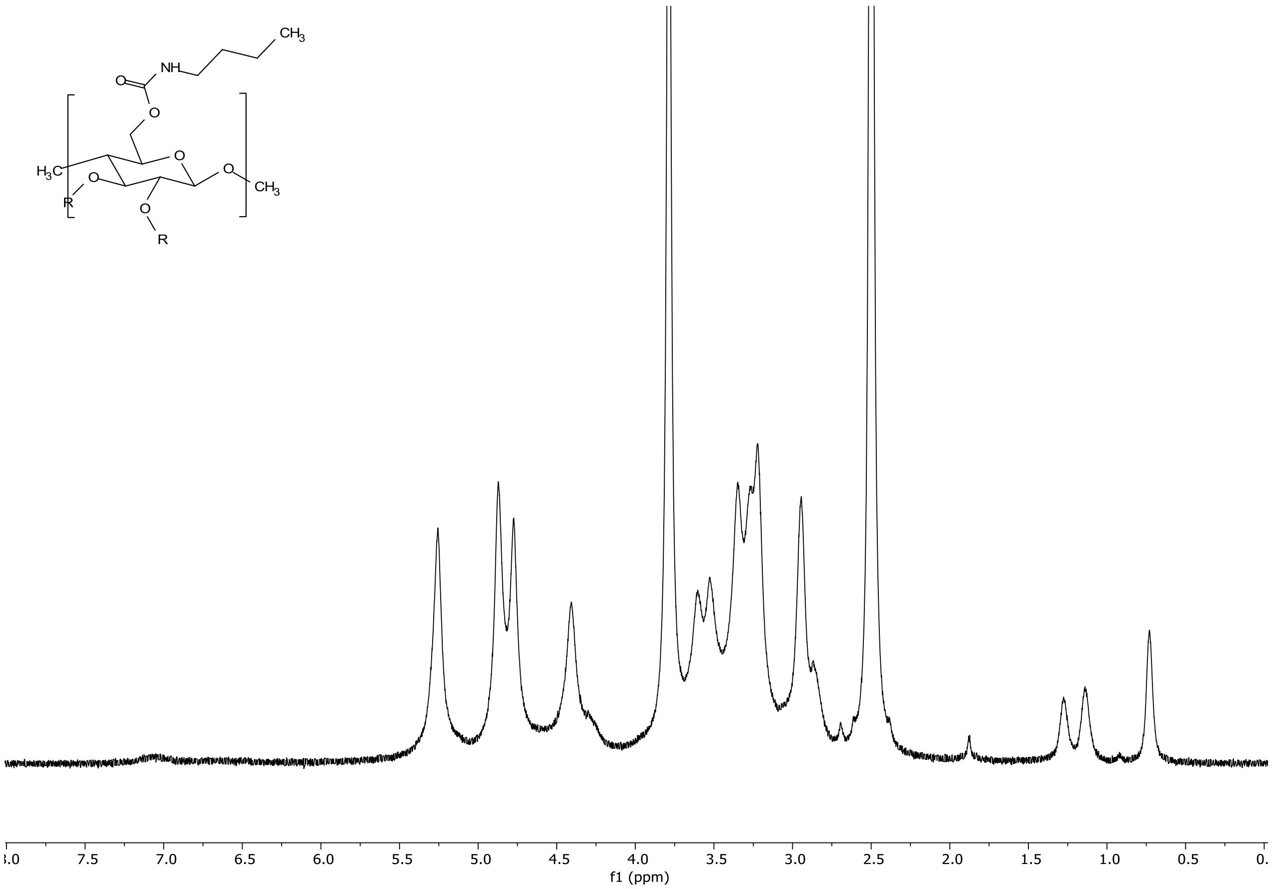

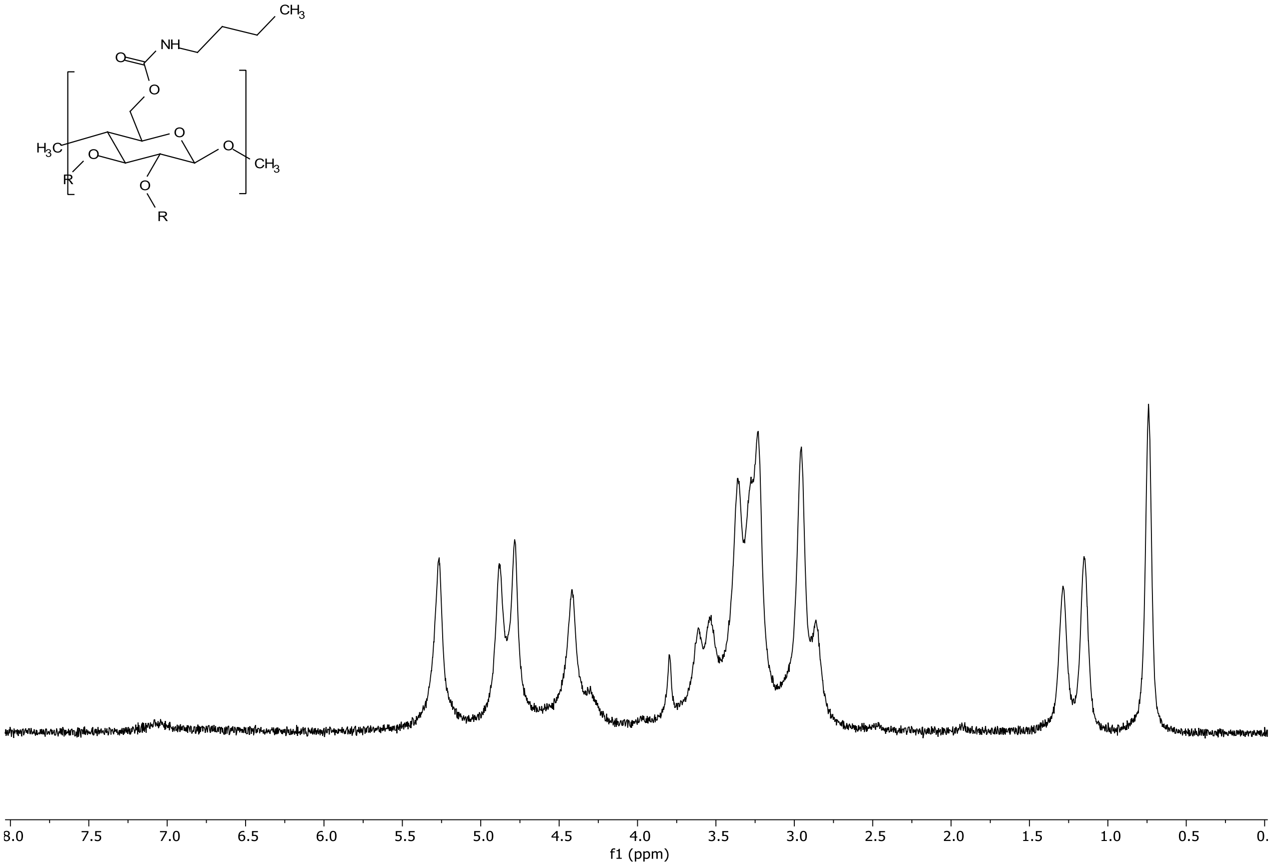

^{1}H and diffusion-edited ^{1}H spectra of **Cellulose *N*-butyl carbamate DS 0.17** in DMSO-d_6/LiCl solution. (R: H or -$CONHCH_2CH_2CH_2CH_3$)

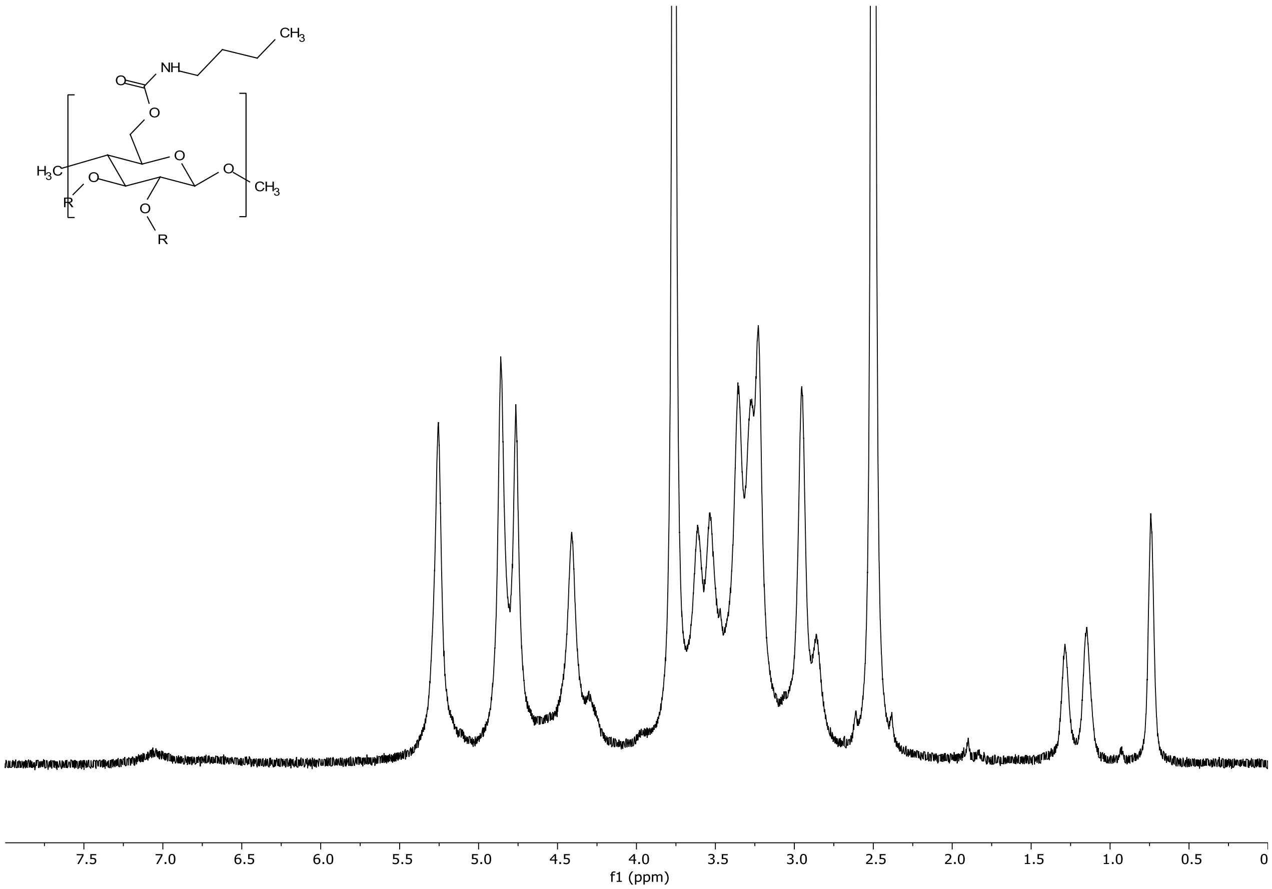

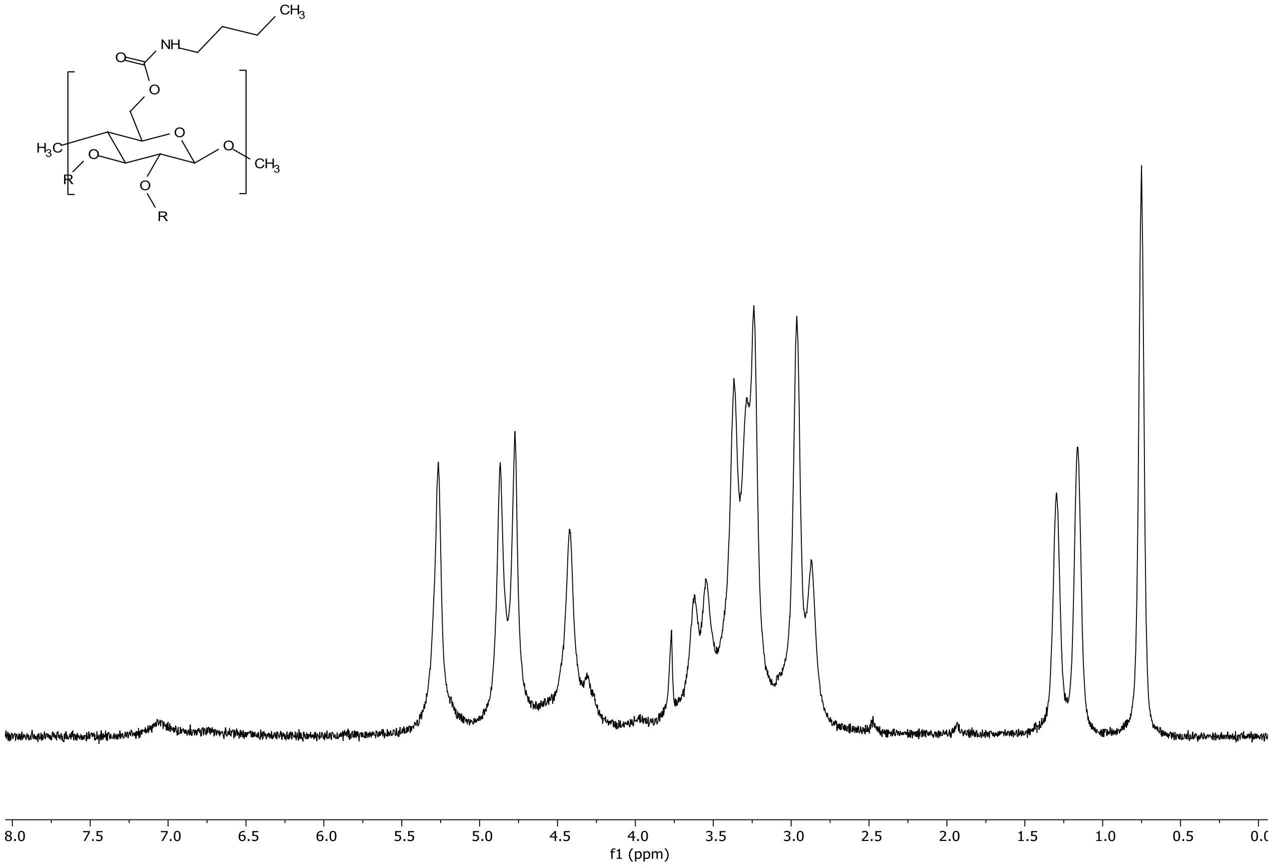

^{1}H and diffusion-edited ^{1}H spectra of **Cellulose *N*-butyl carbamate DS 0.23** in DMSO-d_6/LiCl solution. (R: H or -$CONHCH_2CH_2CH_2CH_3$)

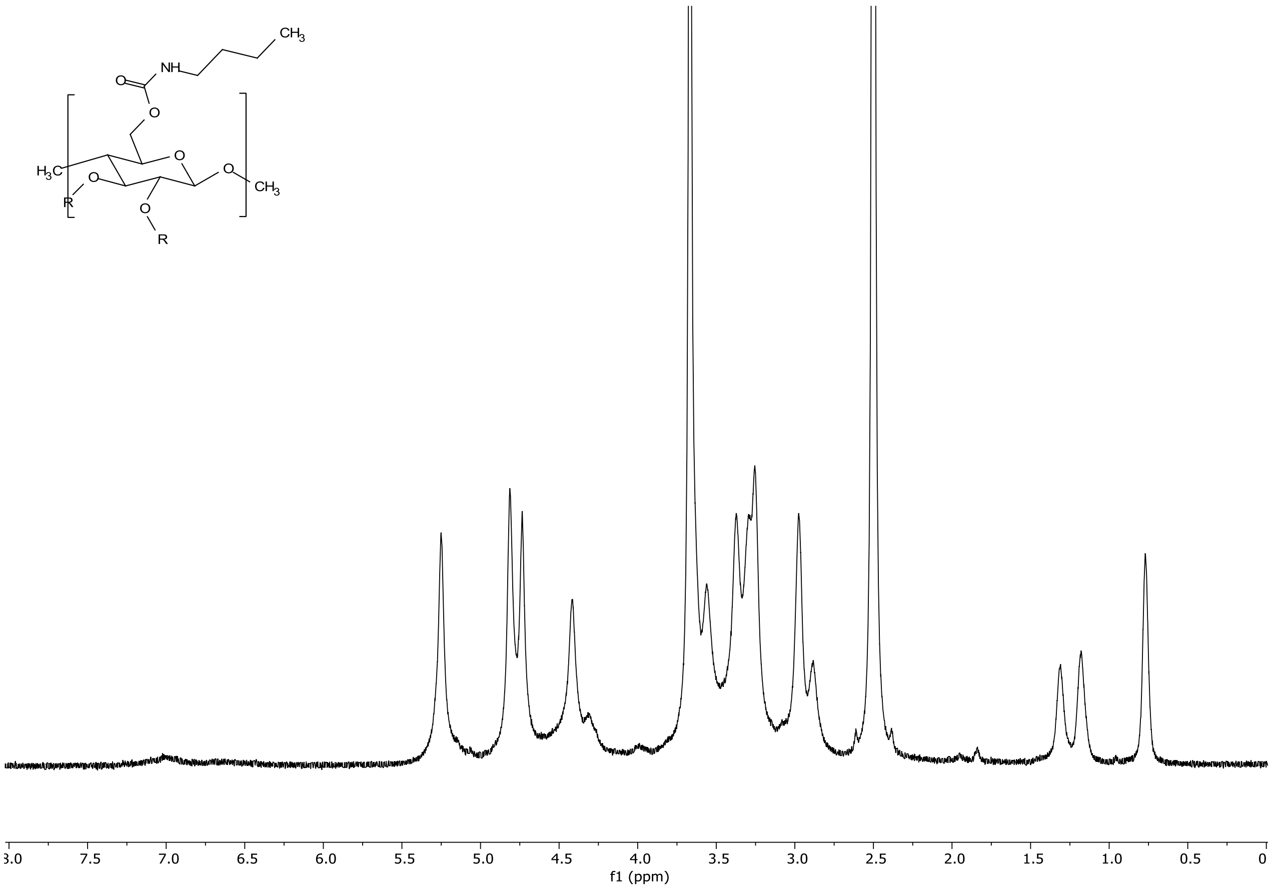

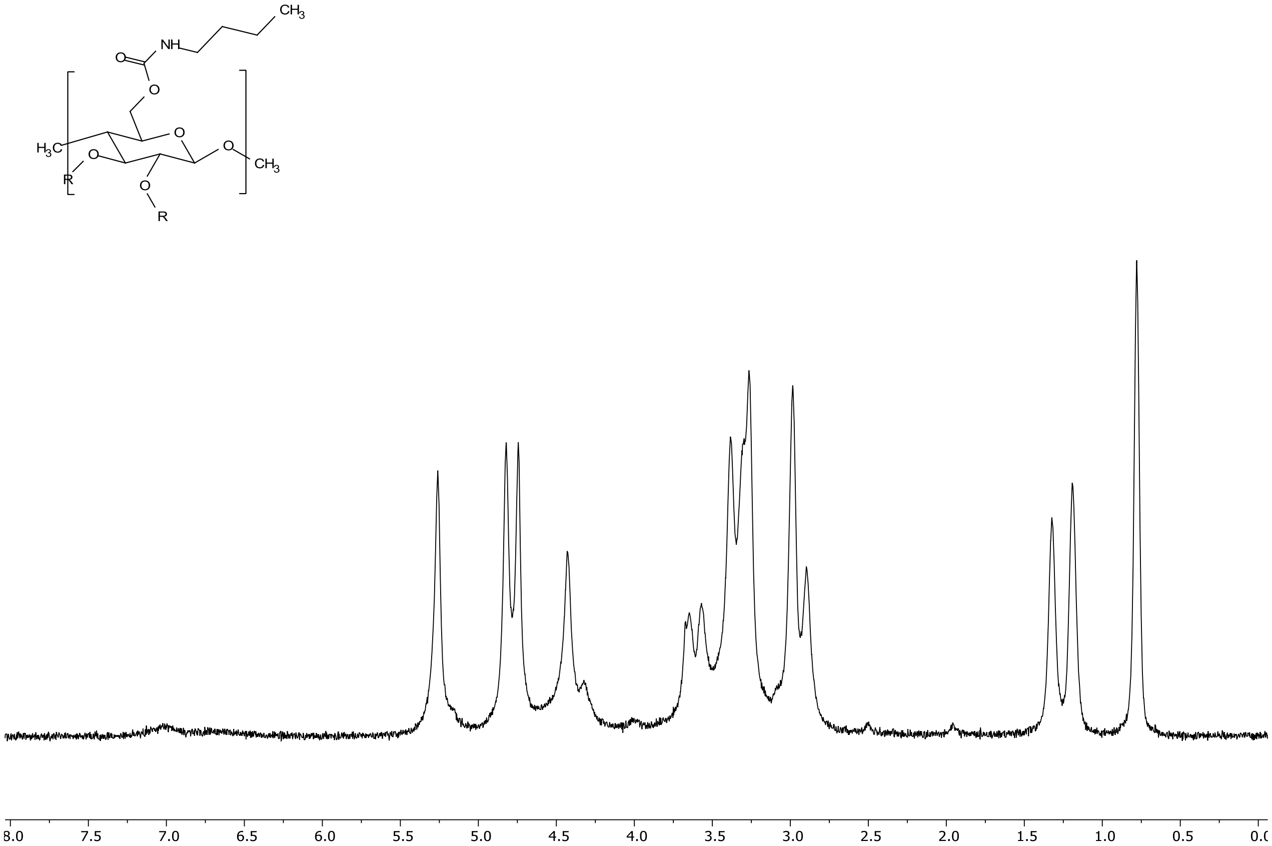

^{1}H and diffusion-edited ^{1}H spectra of **Cellulose *N*-butyl carbamate DS 2.53** in DMSO-*d₆*/LiCl solution. (R: H or -$CONHCH_2CH_2CH_2CH_3$)

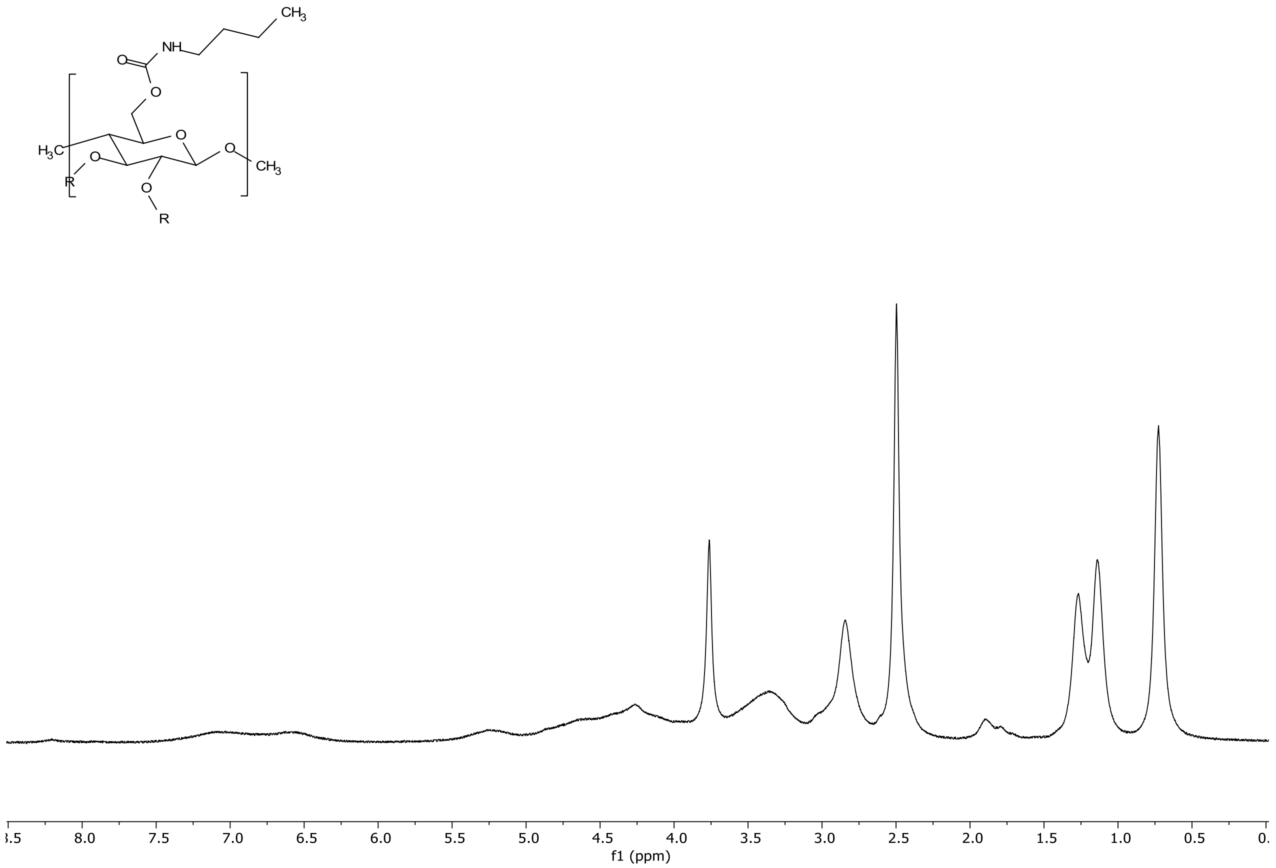

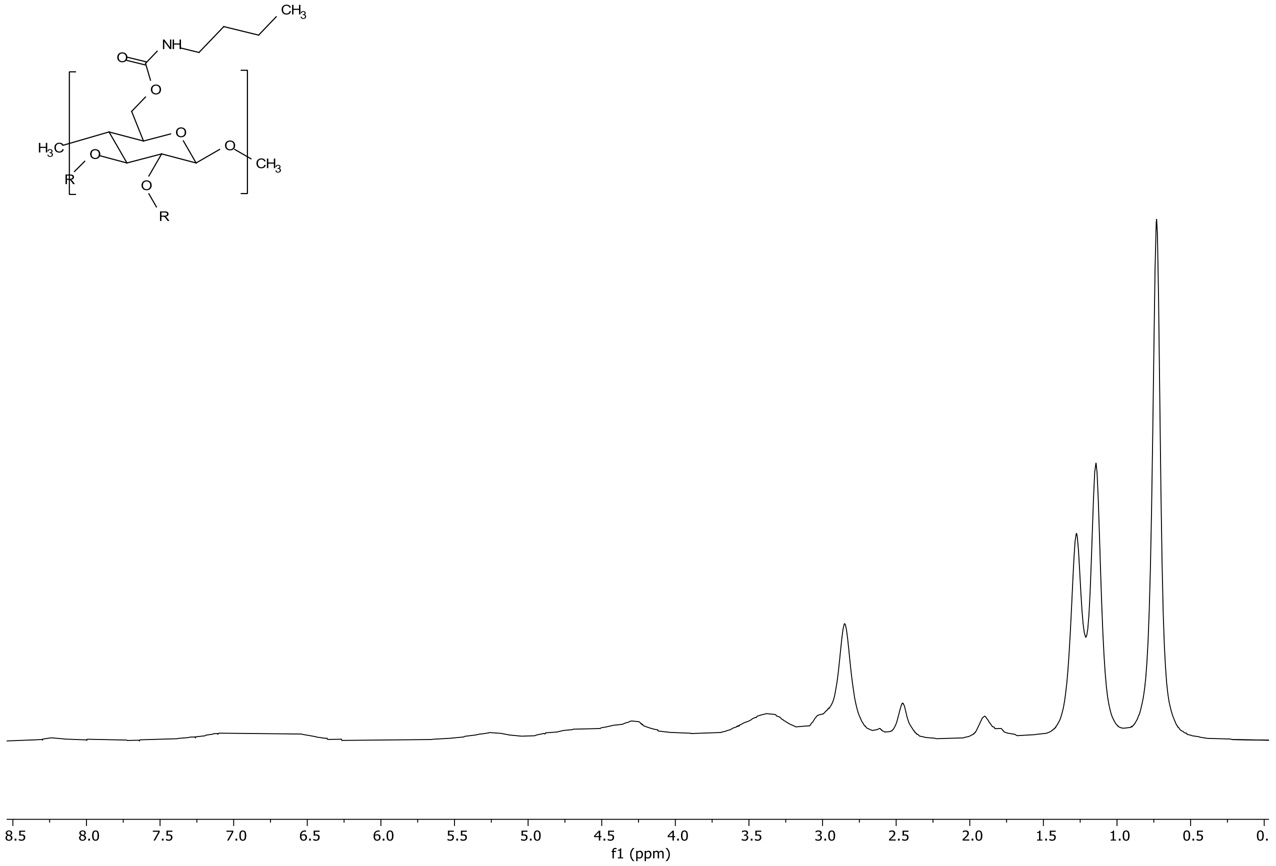

^{1}H and diffusion-edited ^{1}H spectra of **per-O-acetylated Cellulose *N*-phenyl carbamate (Sample A)** in DMSO-d_6. (R: -CONHPh or -$COCH_3$)

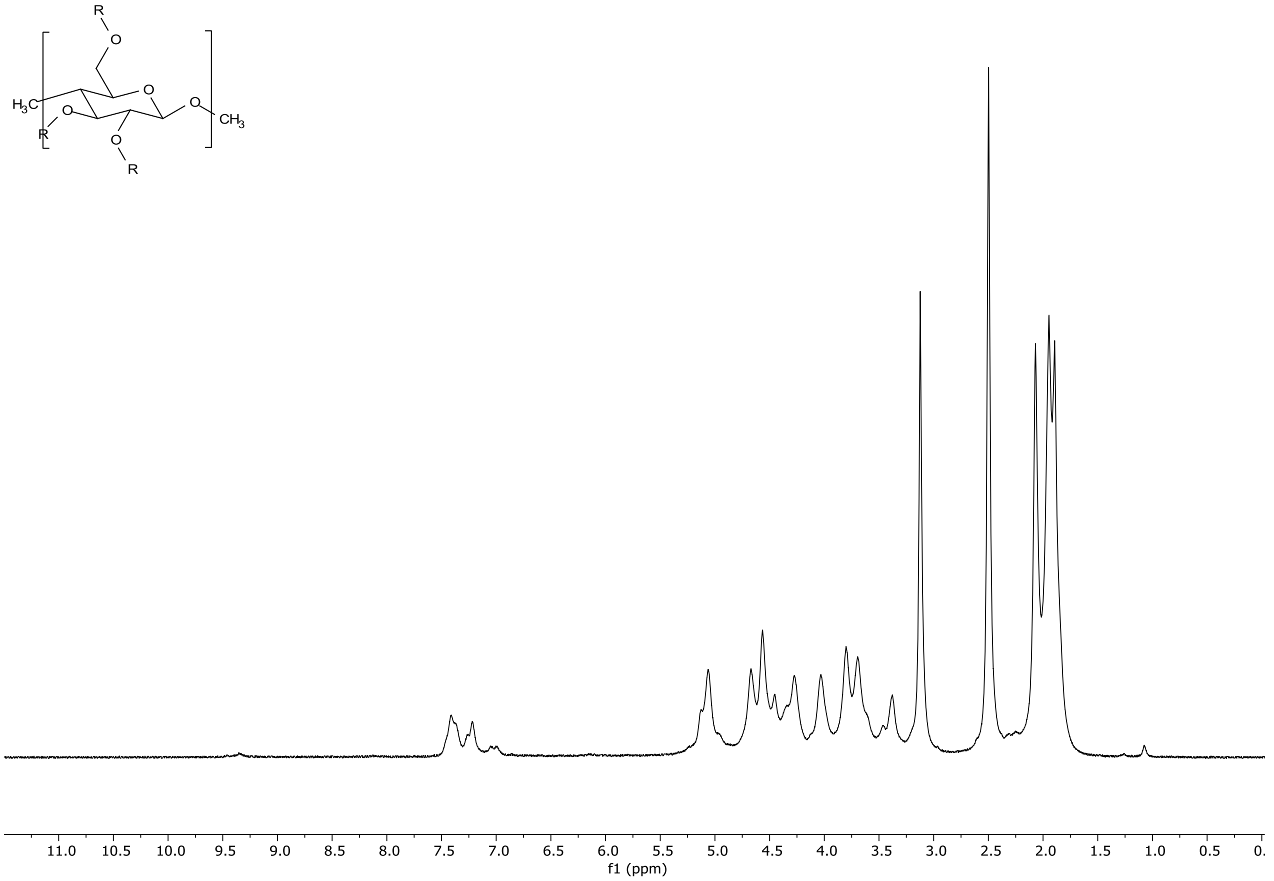

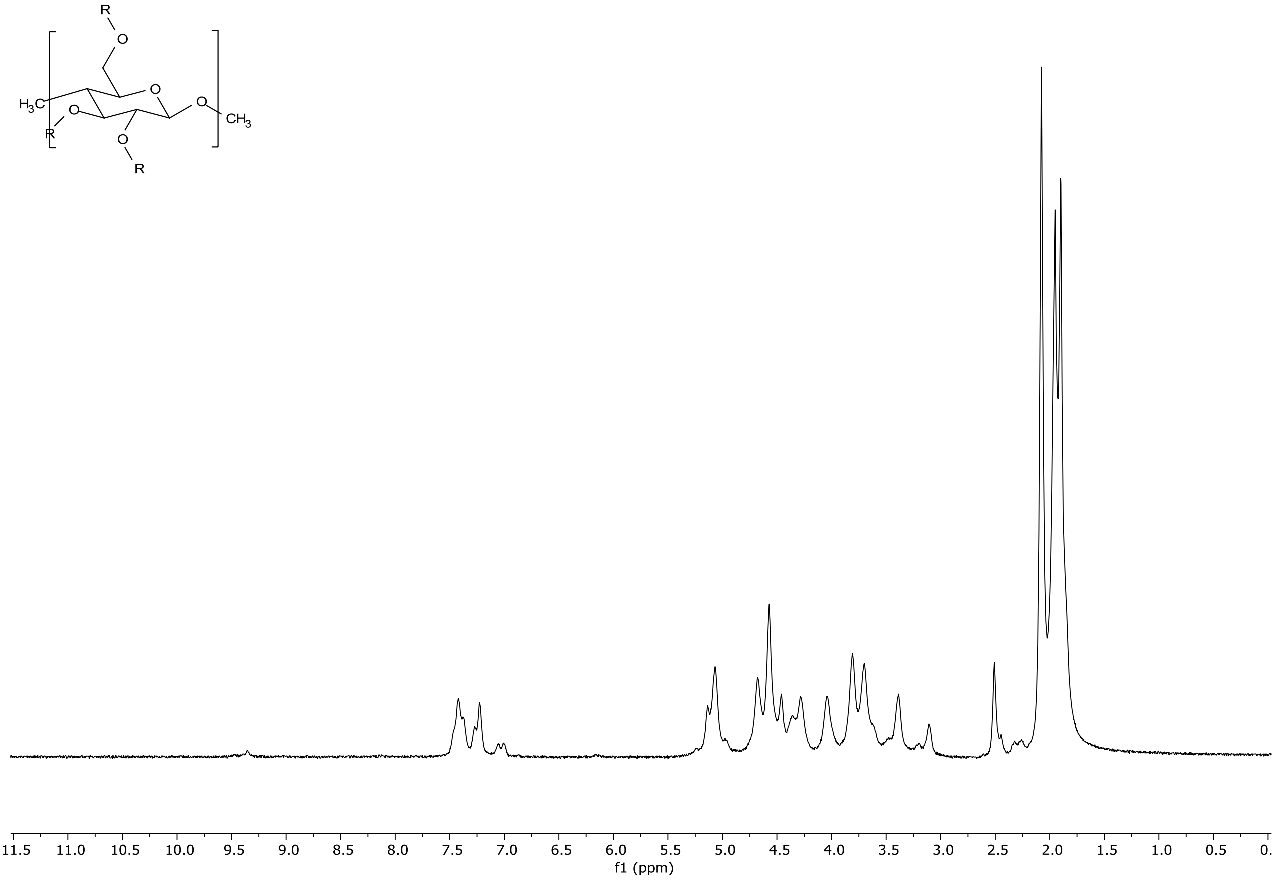

Quantitative $^{13}C\{1H\}$ spectra of **per-O-acetylated Cellulose *N*-phenyl carbamate (Sample A)** in DMSO-d_6. (R: -CONHPh or -$COCH_3$)

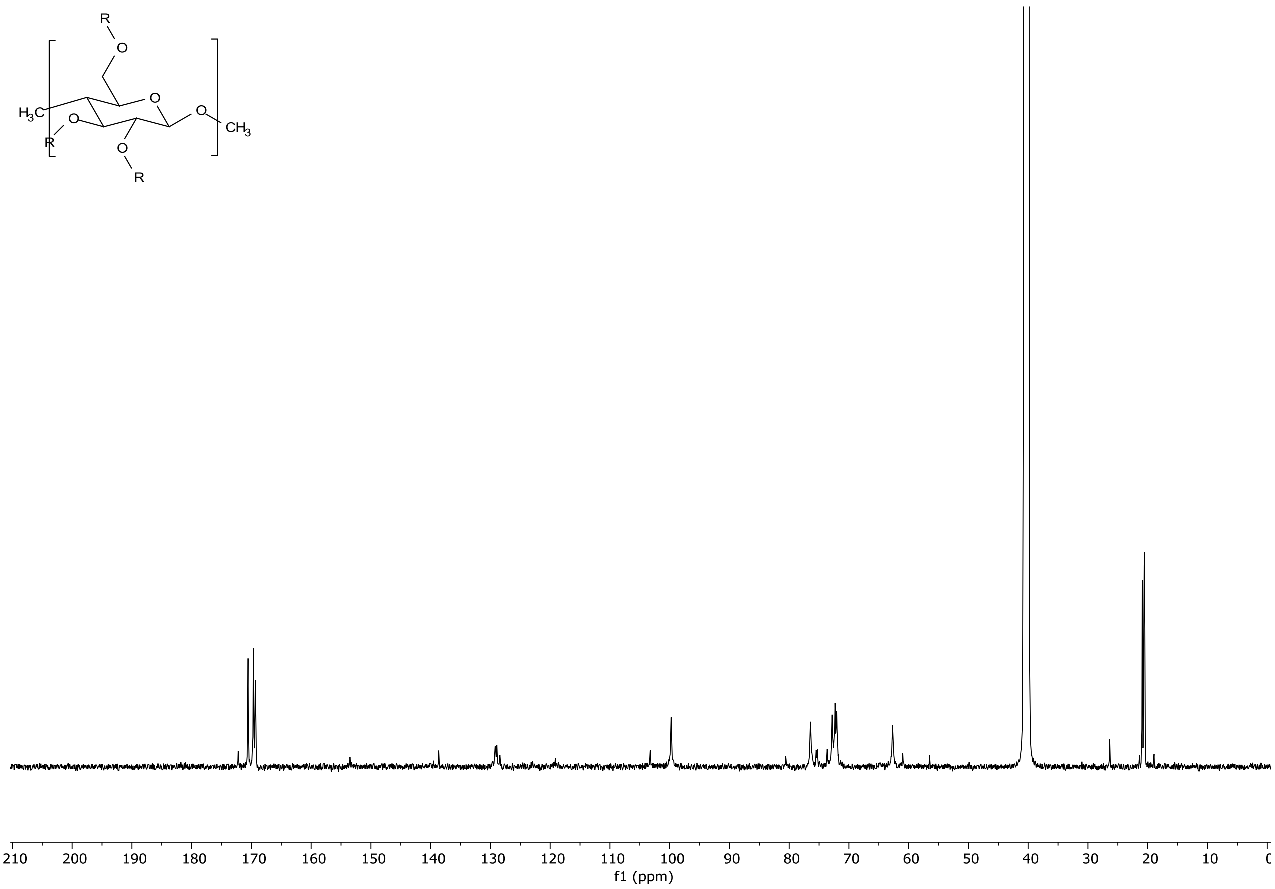

^{1}H spectra of **per-O-acetylated Cellulose *N*-phenyl carbamate (Sample B)** in DMSO-d_6. (R: -CONHPh or -$COCH_3$)

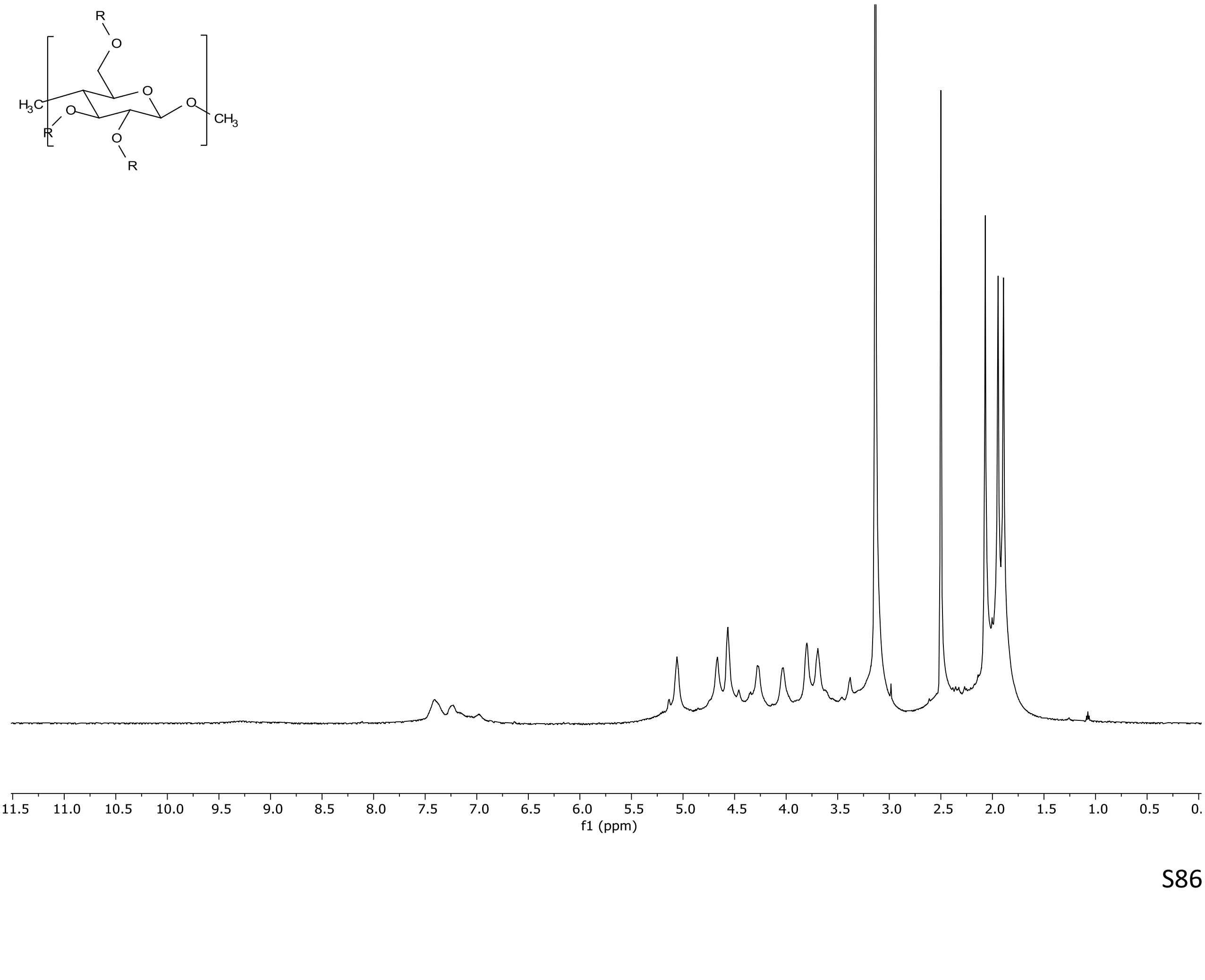

Diffusion-edited ^{1}H and quantitative $^{13}C\{1H\}$ spectra of **per-O-acetylated Cellulose *N*-phenyl carbamate (Sample B)** in DMSO-d_6. (R: -CONHPh or -$COCH_3$)

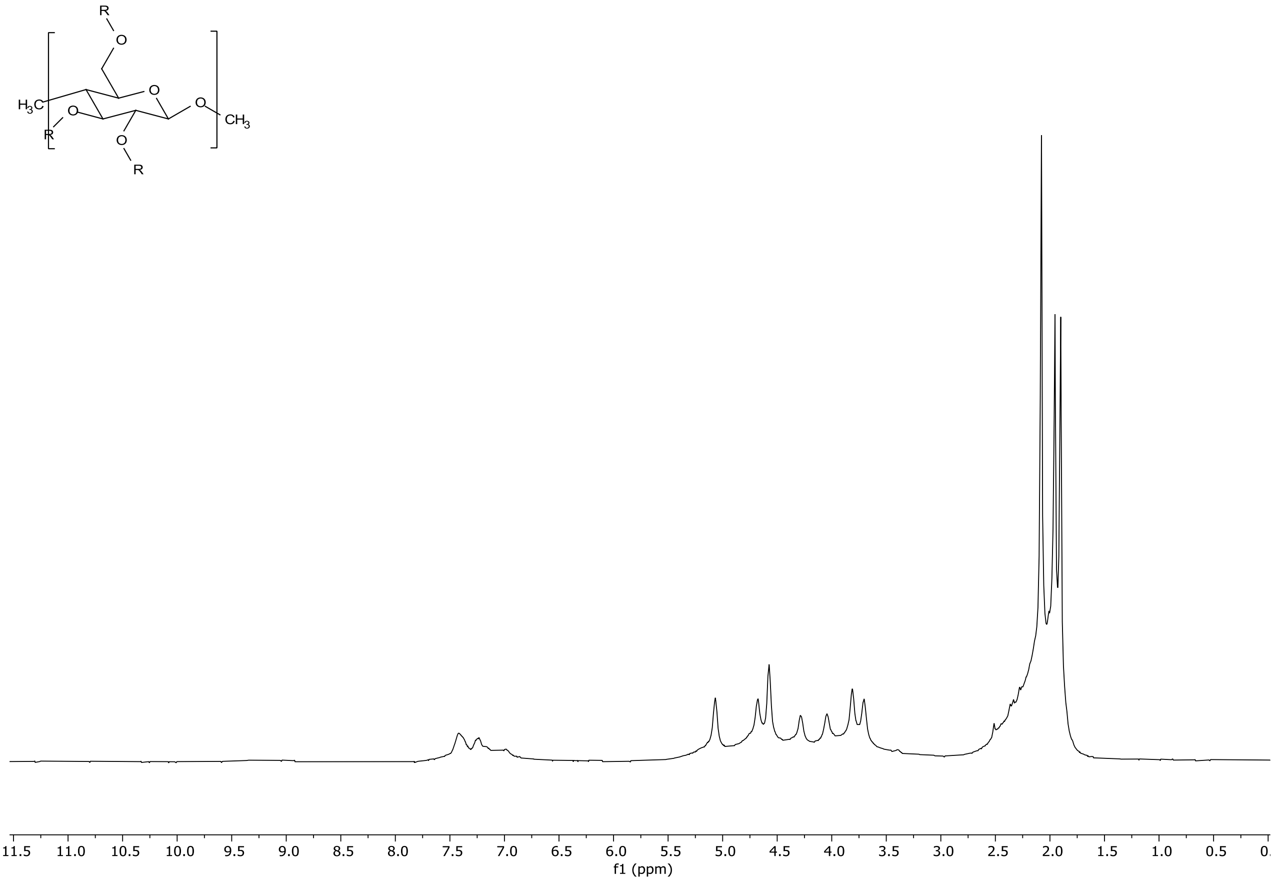

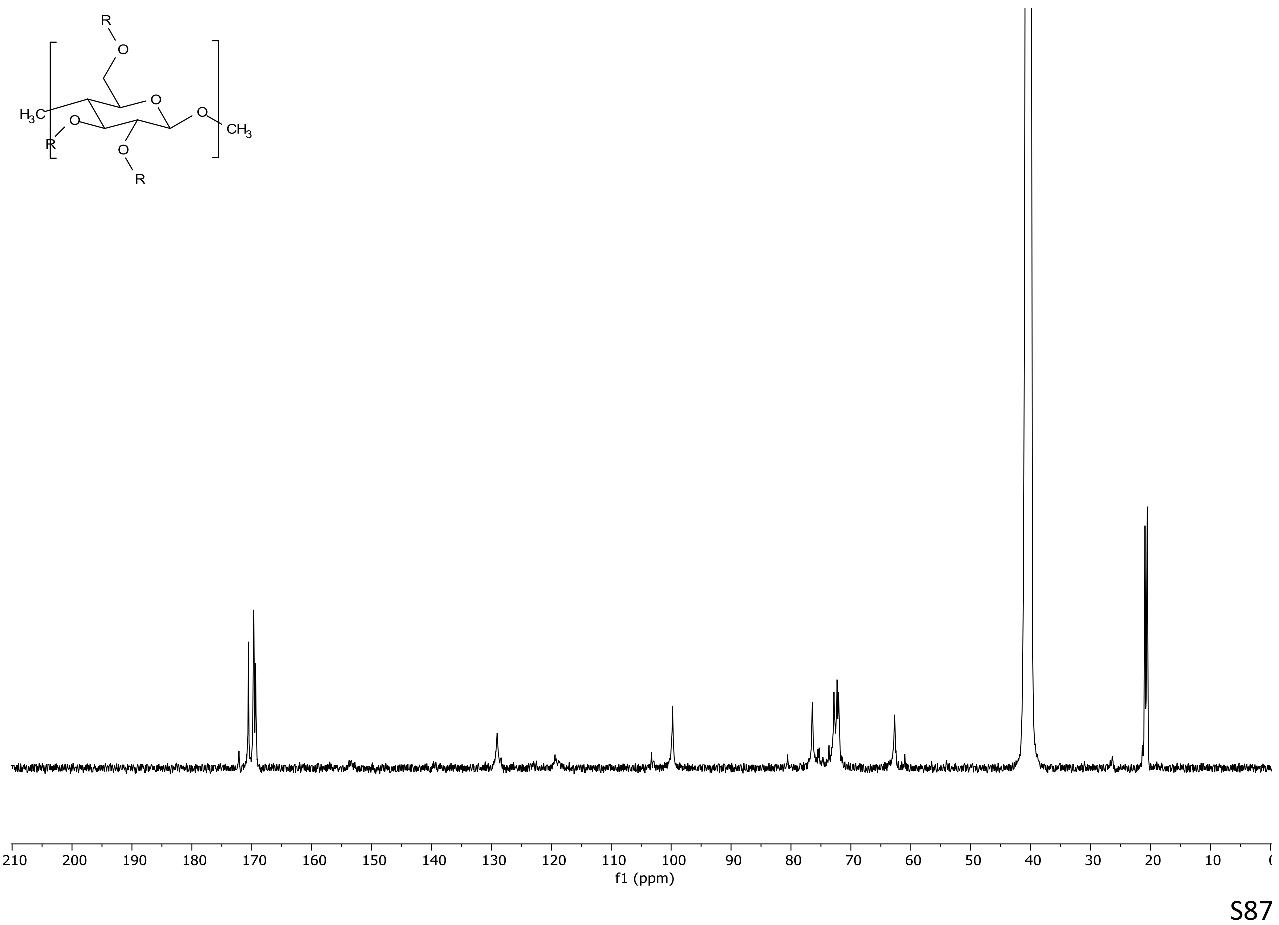

^{1}H and ^{13}C{1H} spectra of **methyl *N*-ethyl carbamate** in DMSO-d_6.

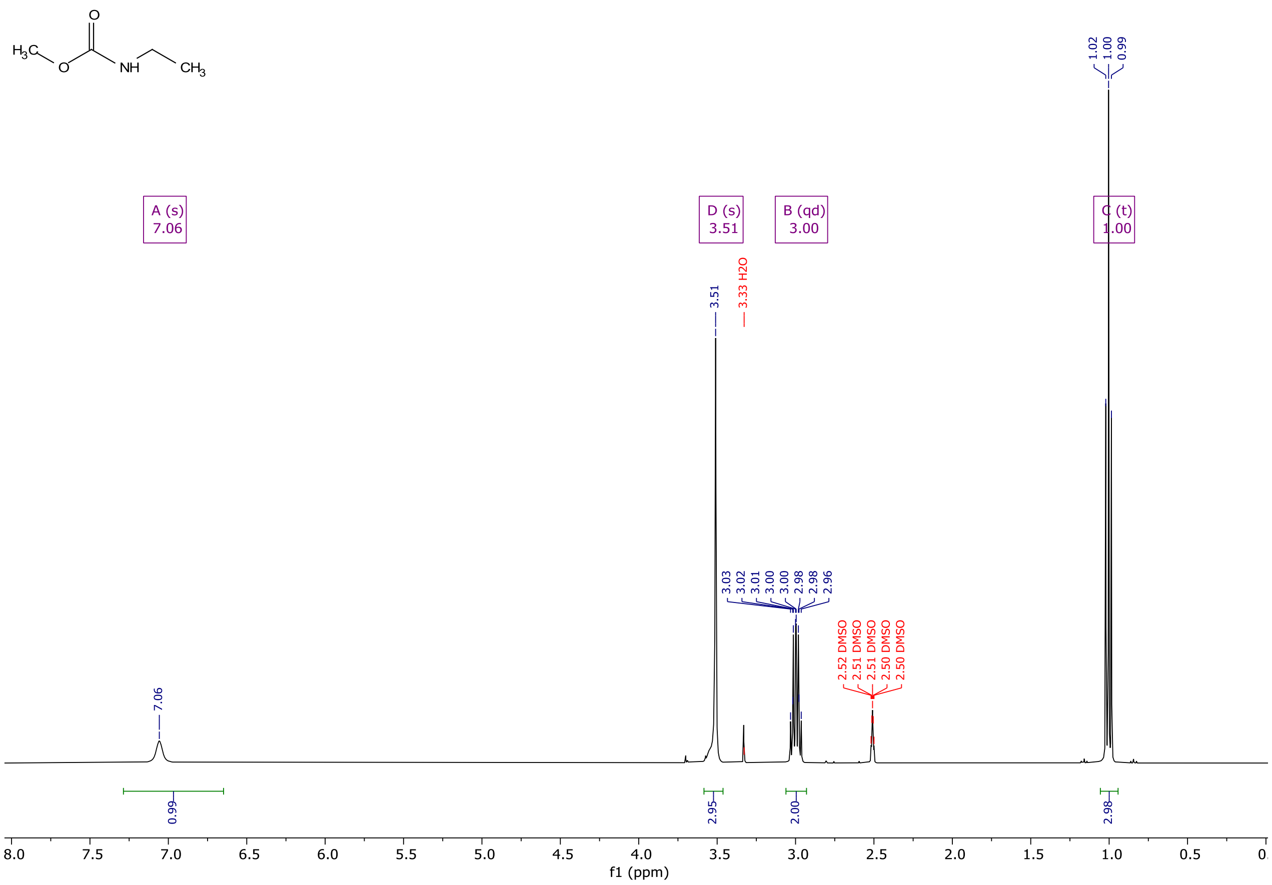

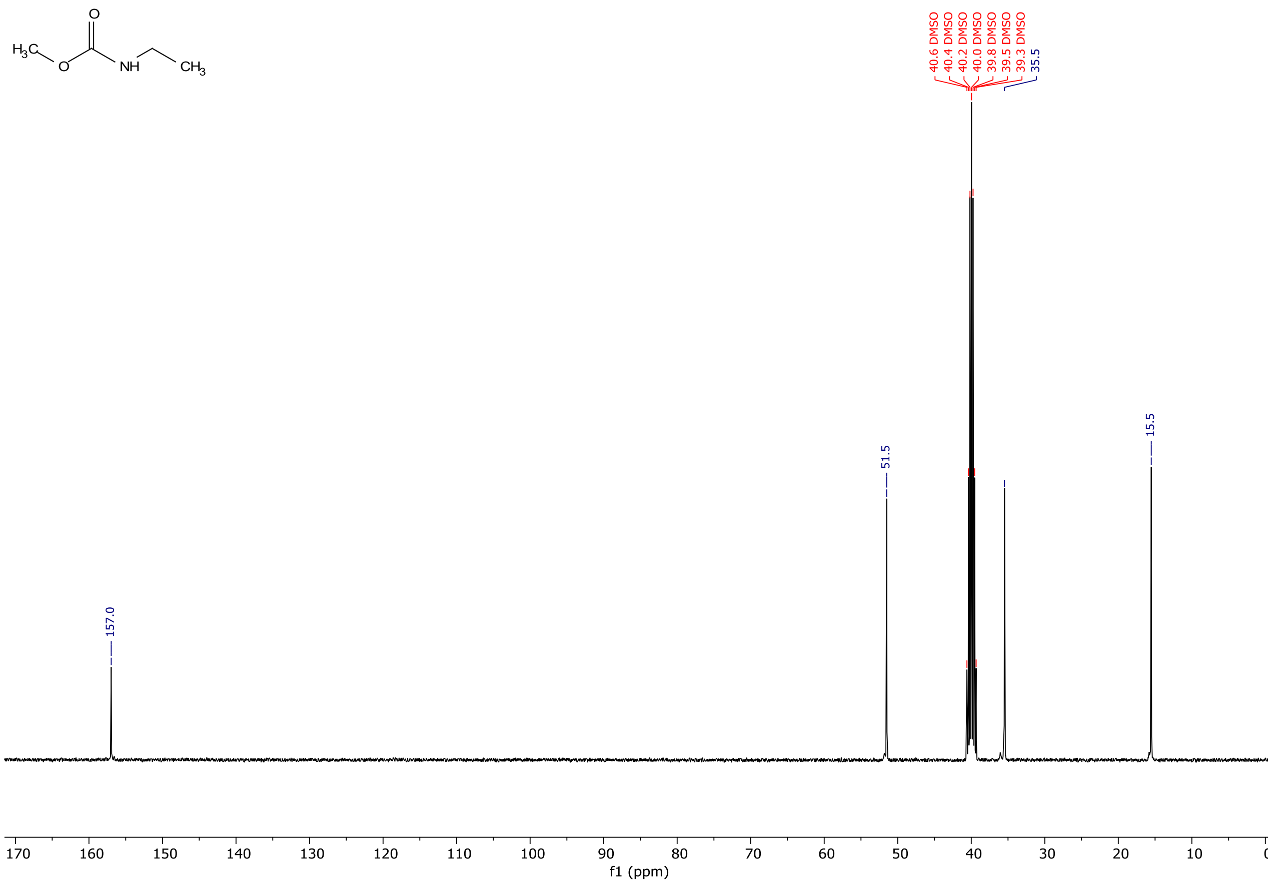

^{1}H and ^{13}C{1H} spectra of **methyl *N*-propyl carbamate** in DMSO-d_6.

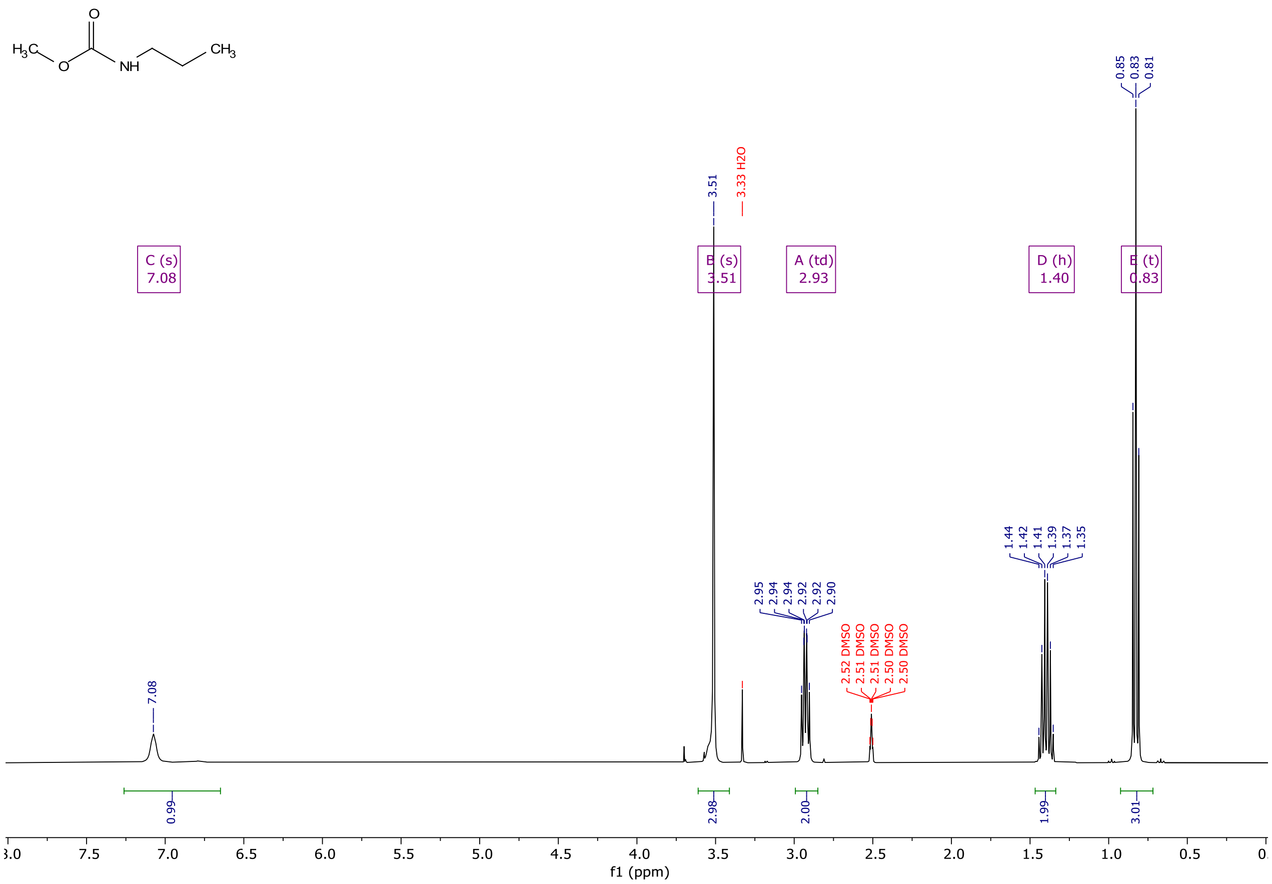

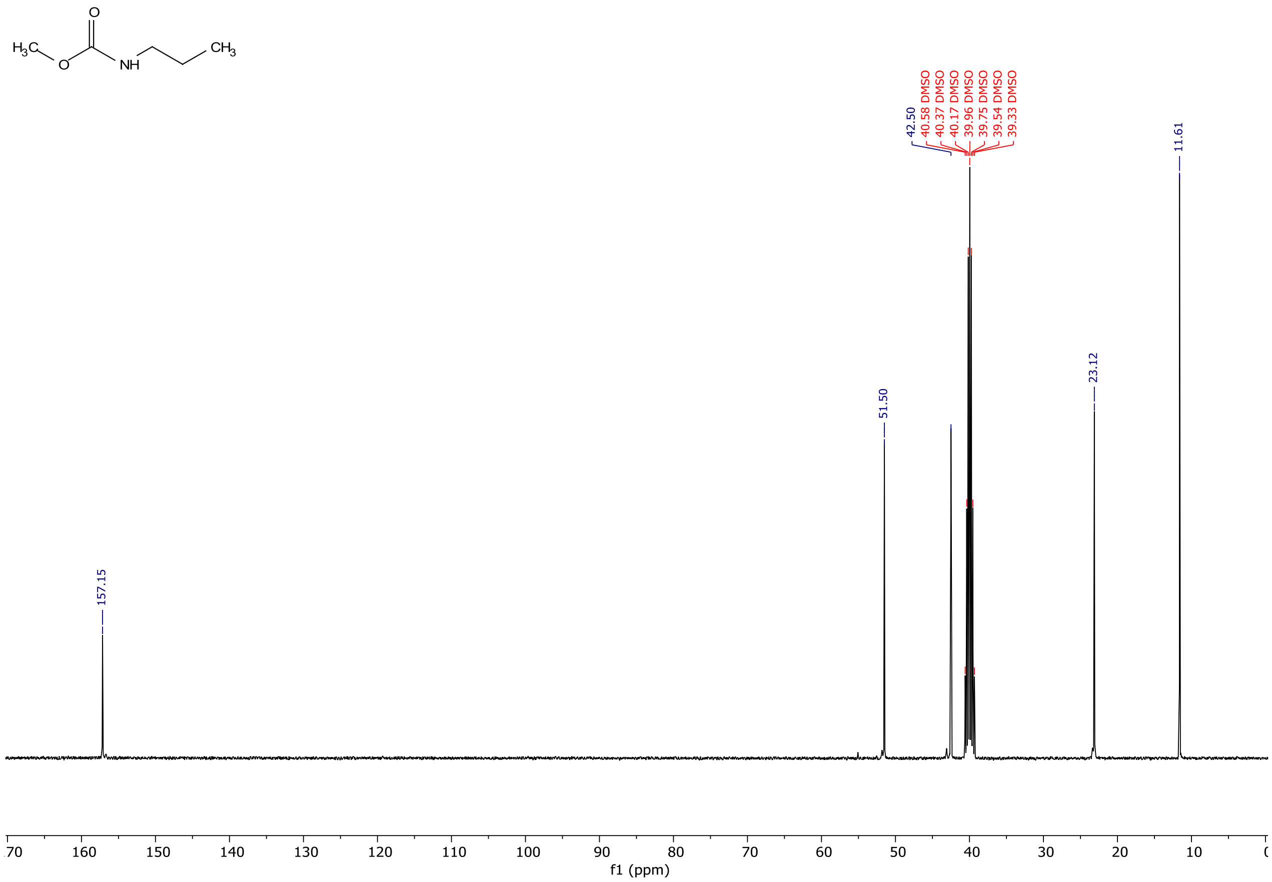

^{1}H and $^{13}C\{1H\}$ spectra of **methyl *N*-butyl carbamate** in DMSO-d_6.

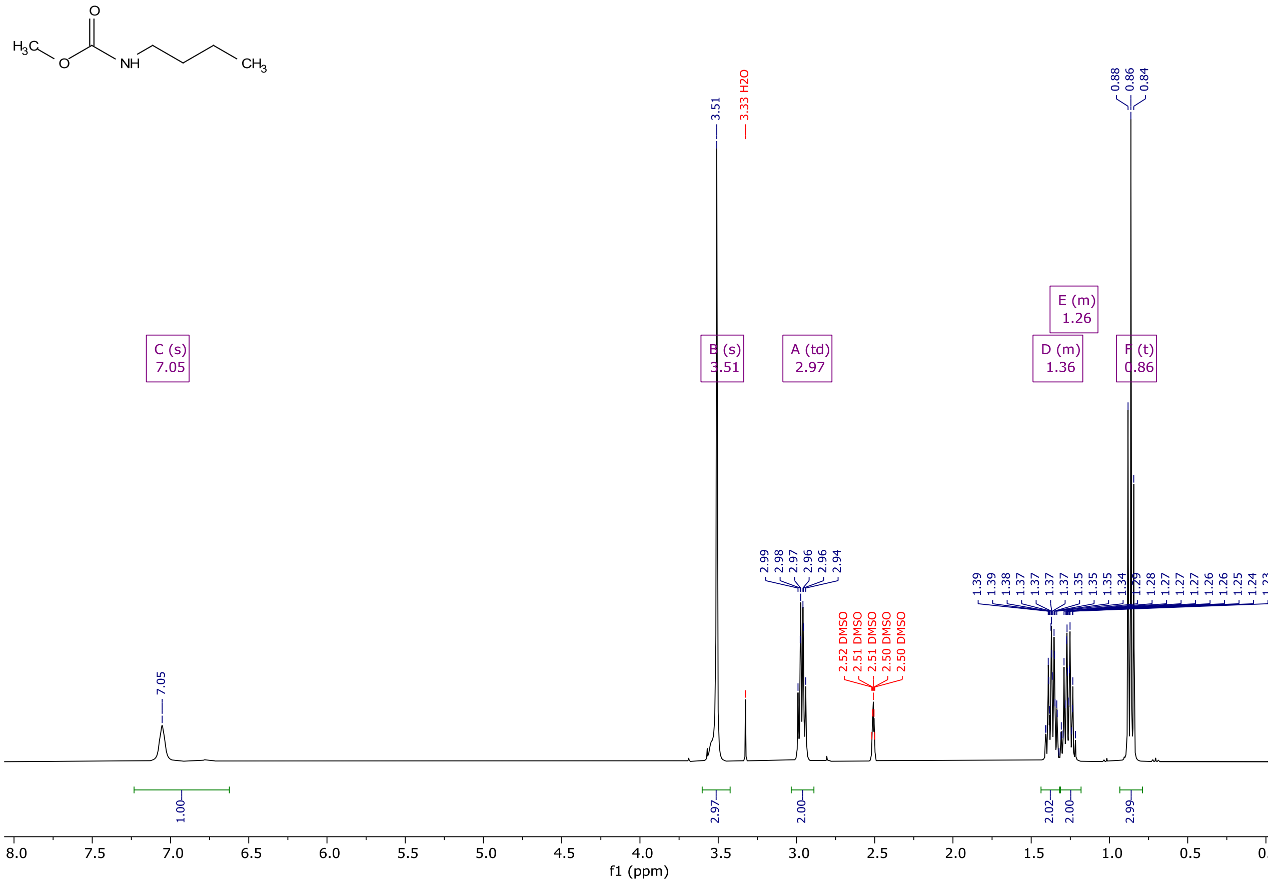

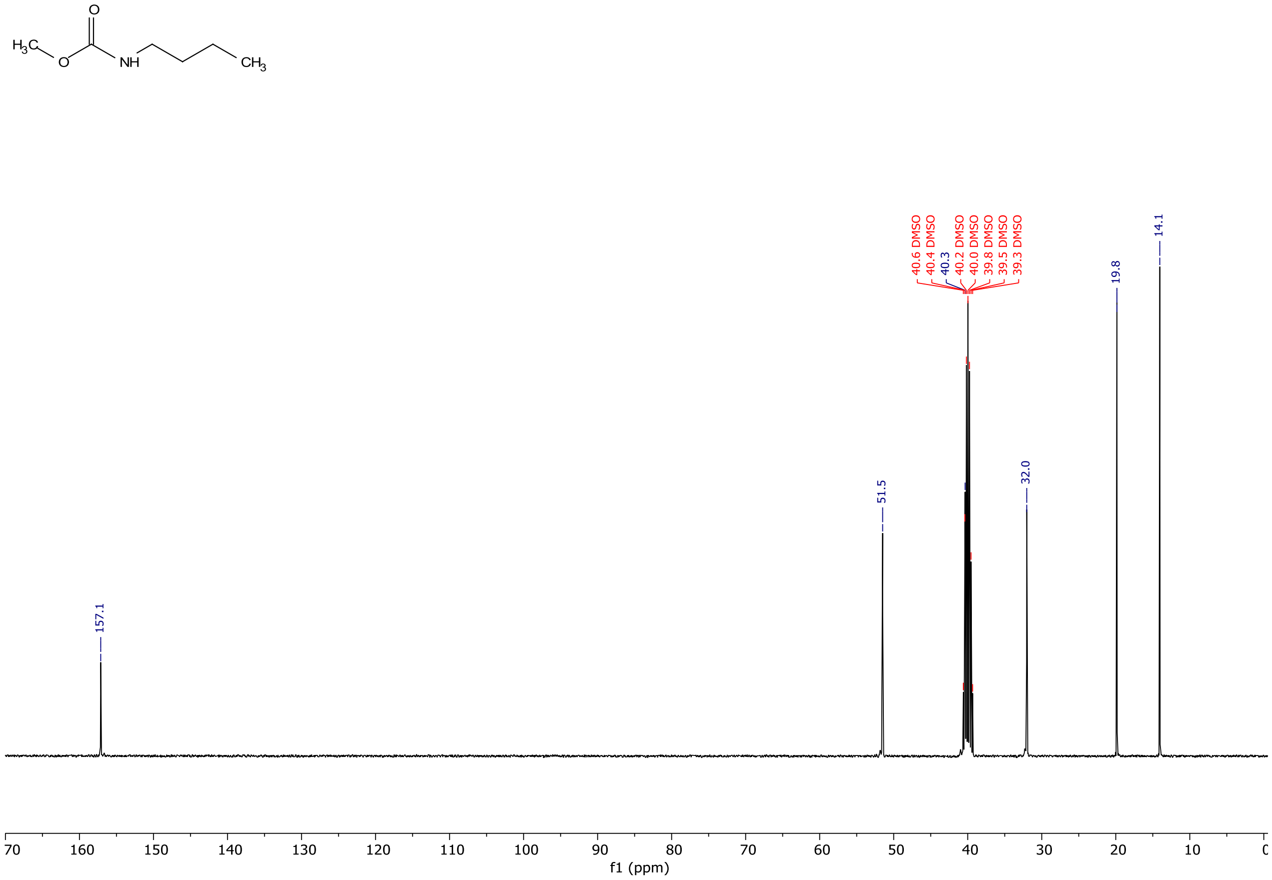

^{1}H and $^{13}C\{1H\}$ spectra of **methyl *N-iso*-propyl carbamate** in DMSO-d_6.

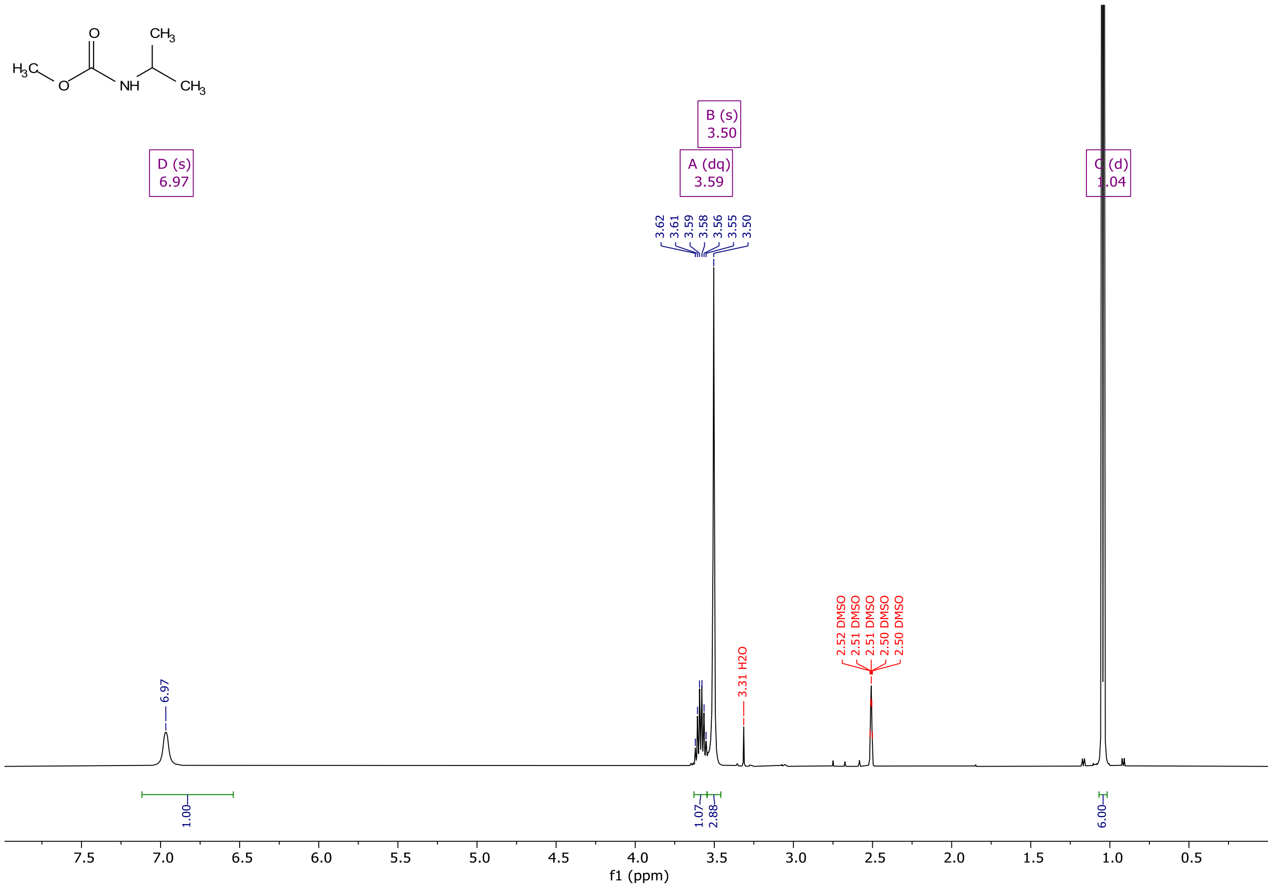

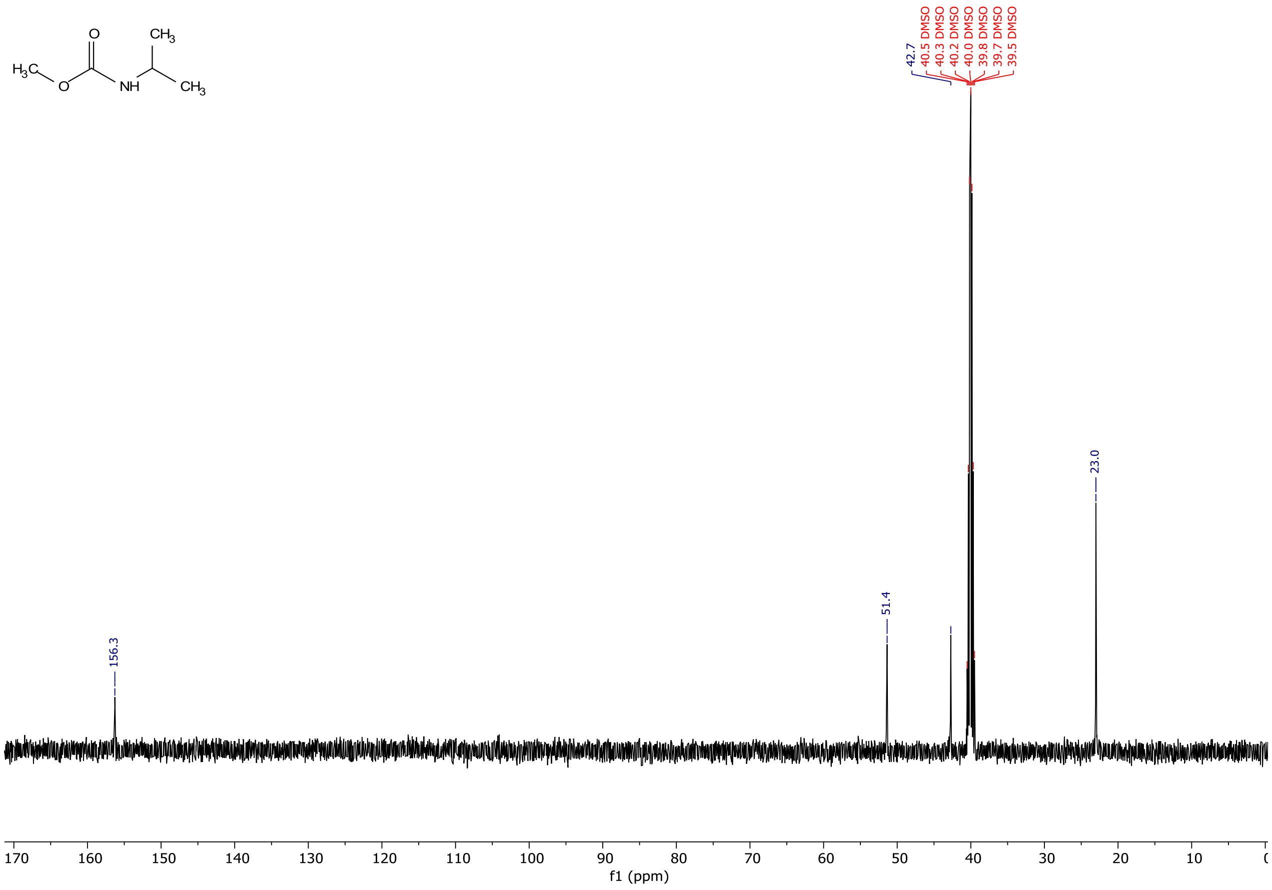

^{1}H and $^{13}C\{^{1}H\}$ spectra of **methyl *N-tert*-butyl carbamate** in DMSO-d_6.

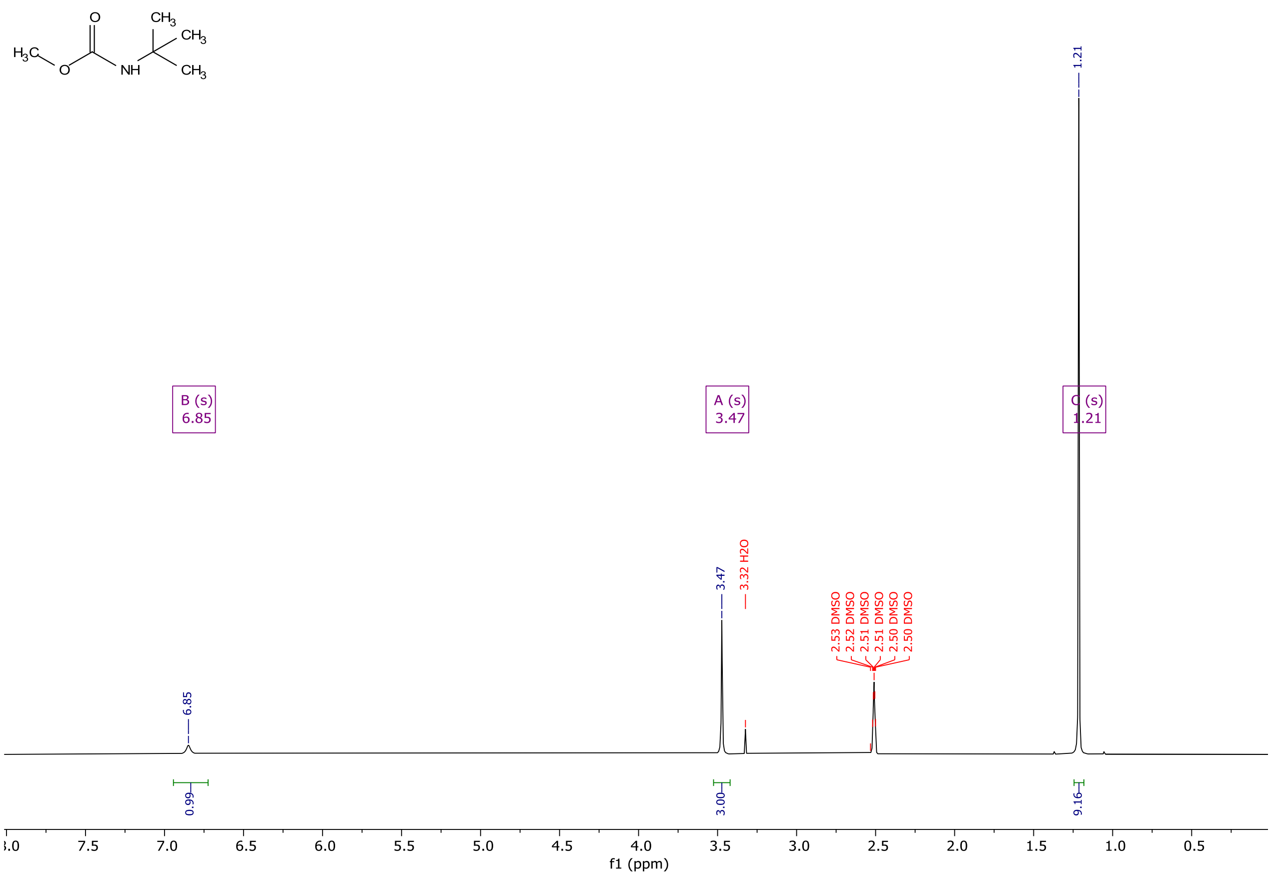

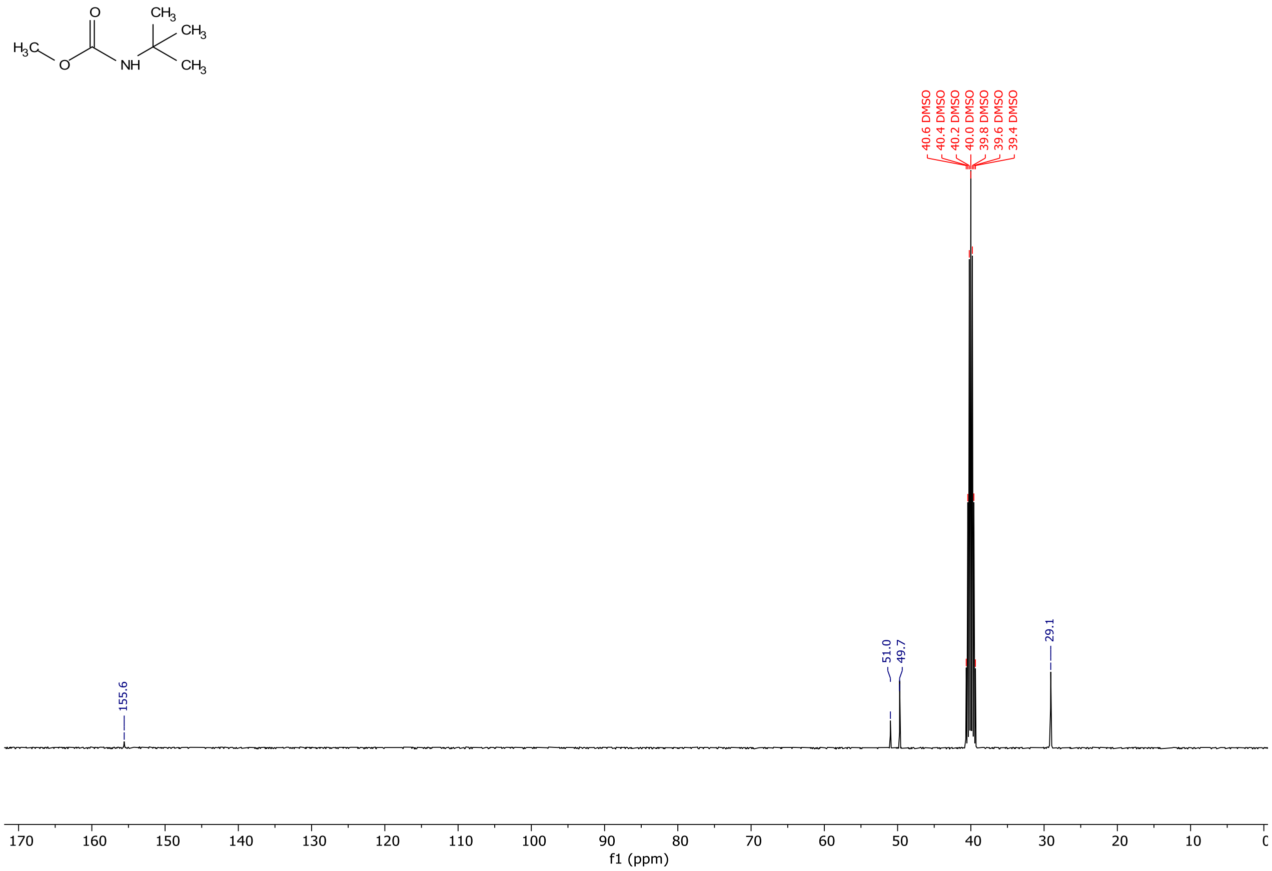

^{1}H and $^{13}C\{1H\}$ spectra of **methyl *N*-4-methoxyphenyl carbamate** in DMSO-d_6.

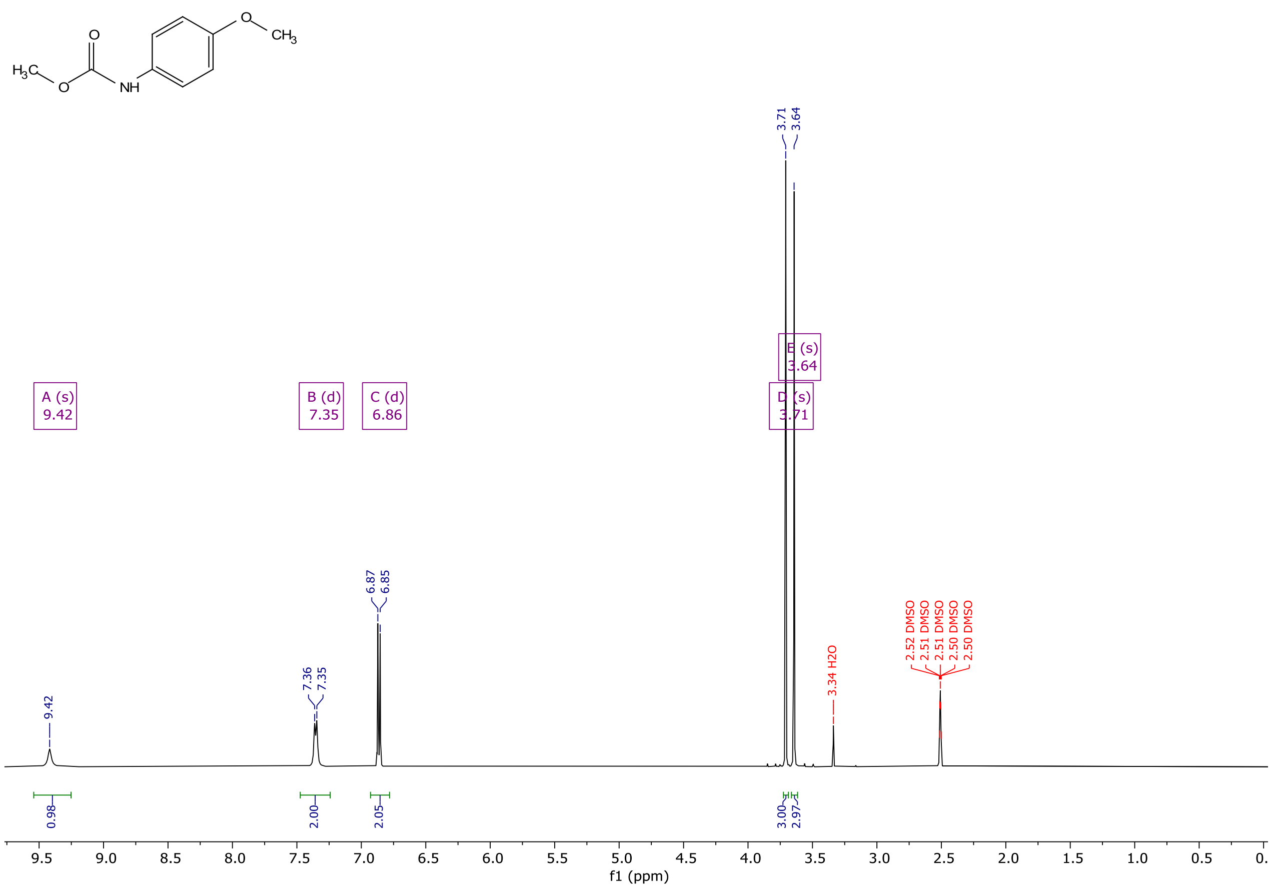

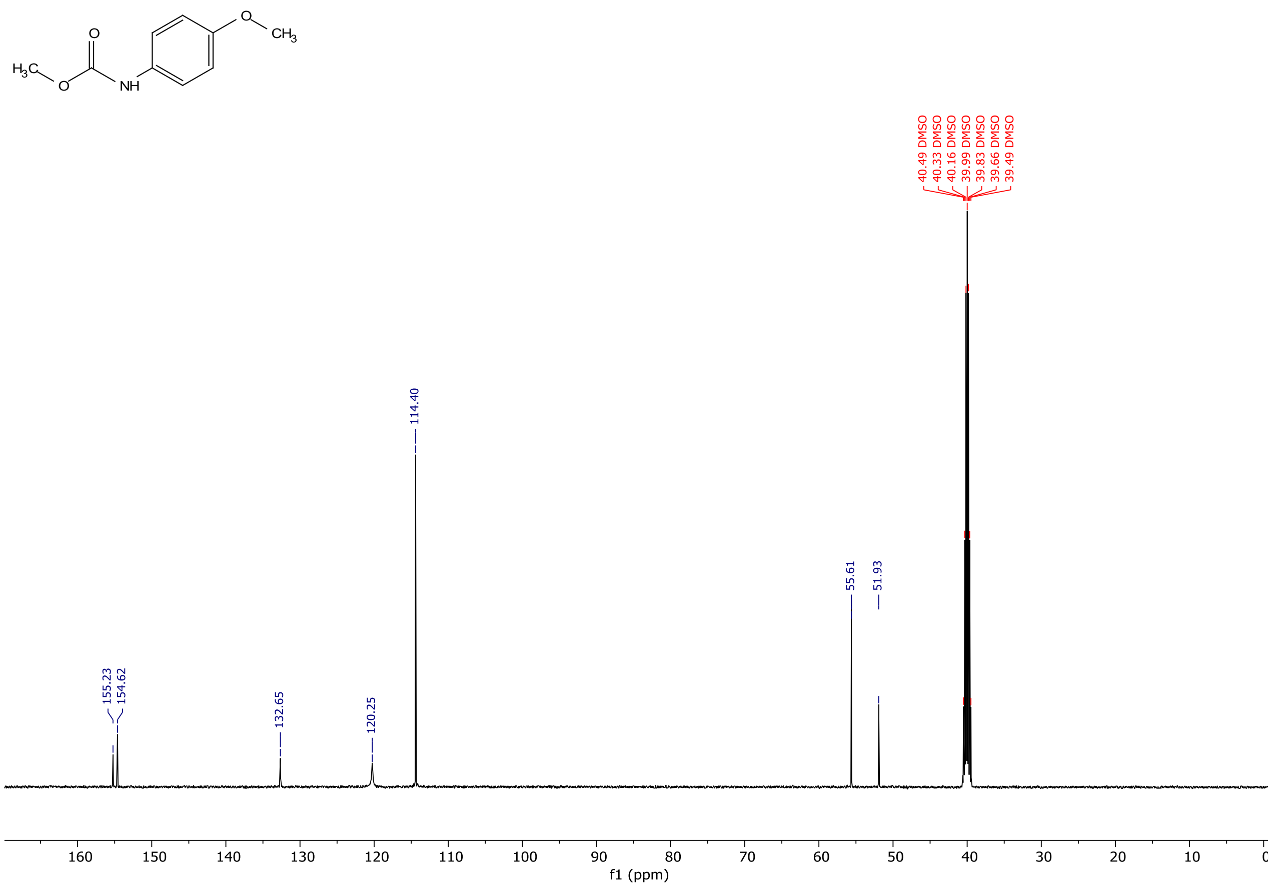

^{1}H and ^{13}C{1H} spectra of **methyl *N*-4-nitrophenyl carbamate** in DMSO-d_6.

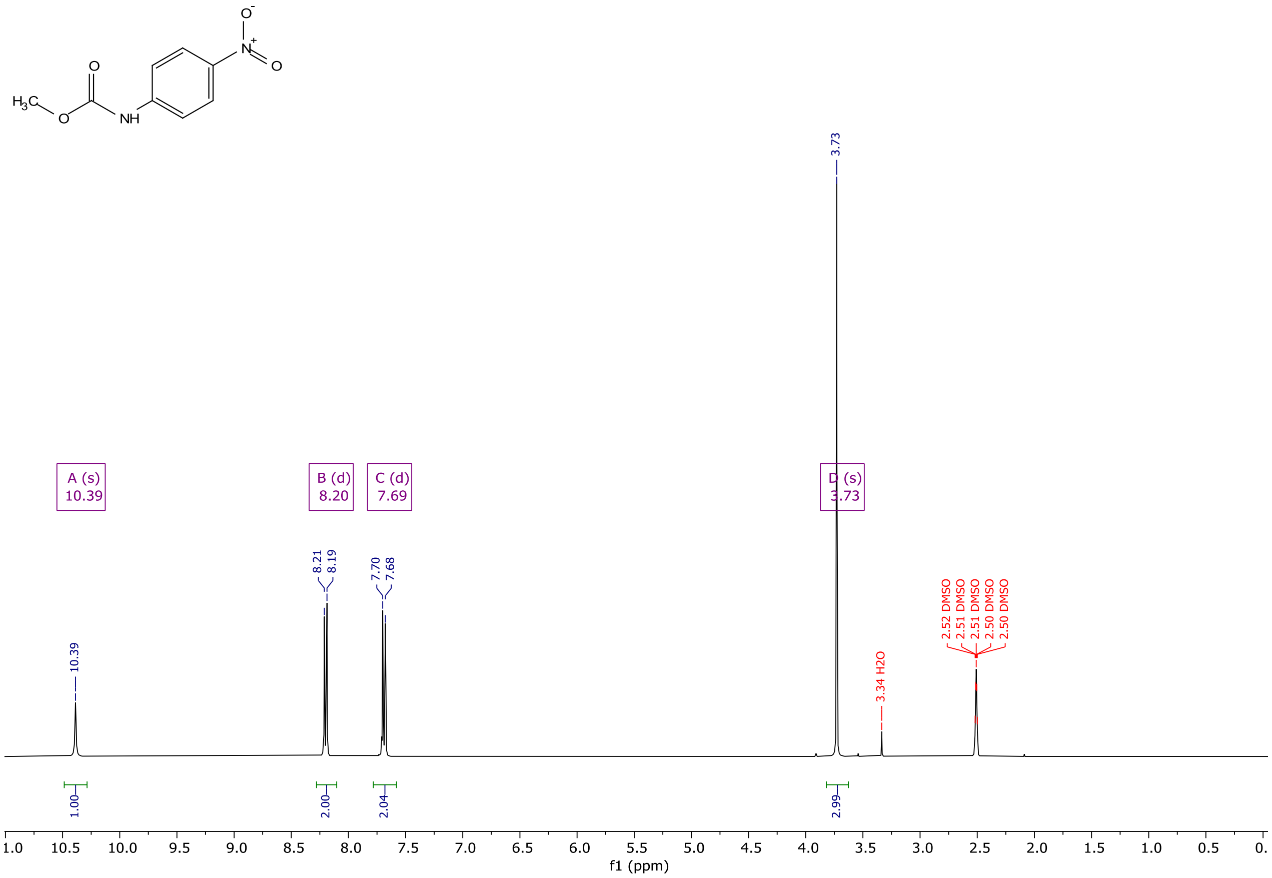

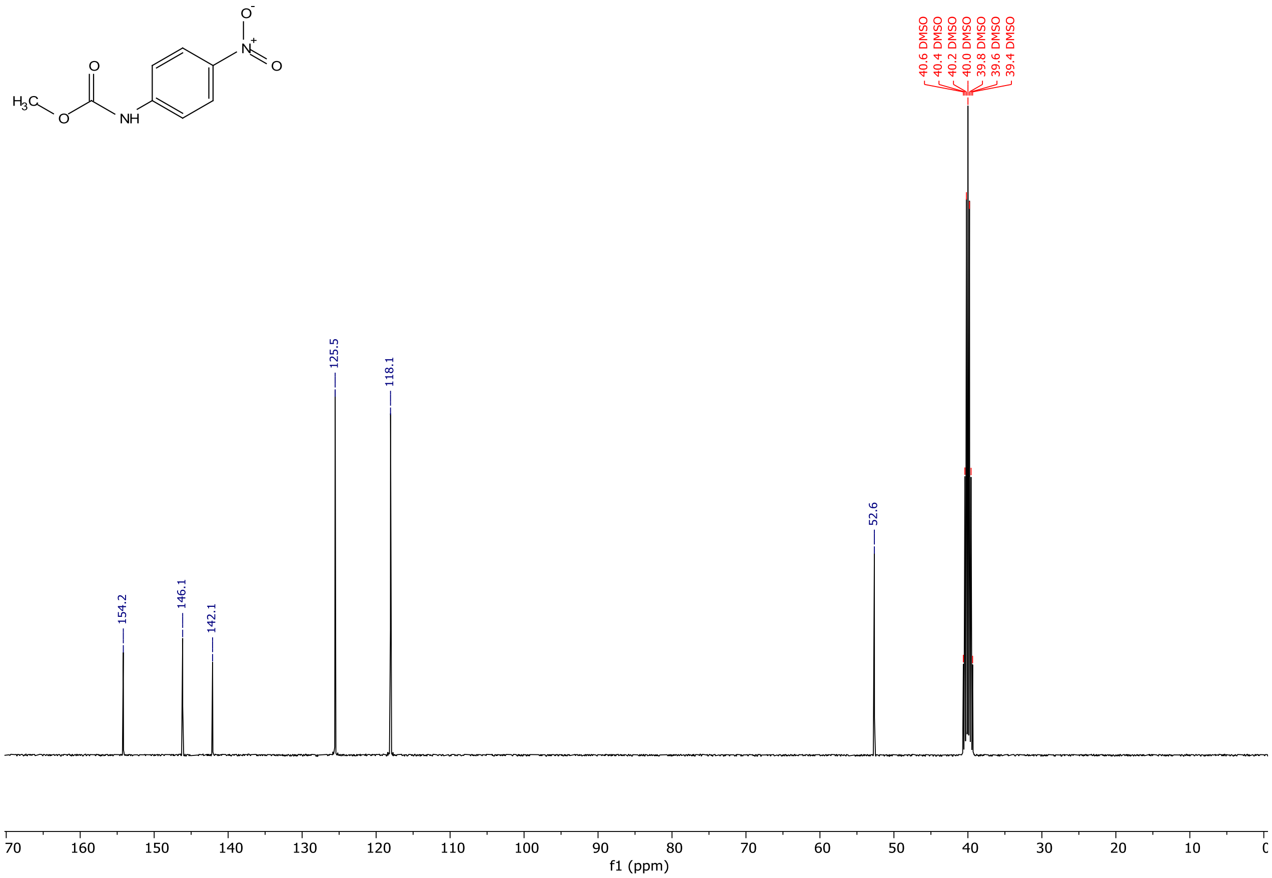

^{1}H and $^{13}C\{1H\}$ spectra of **methyl *N*-styryl carbamate** in DMSO-d_6.

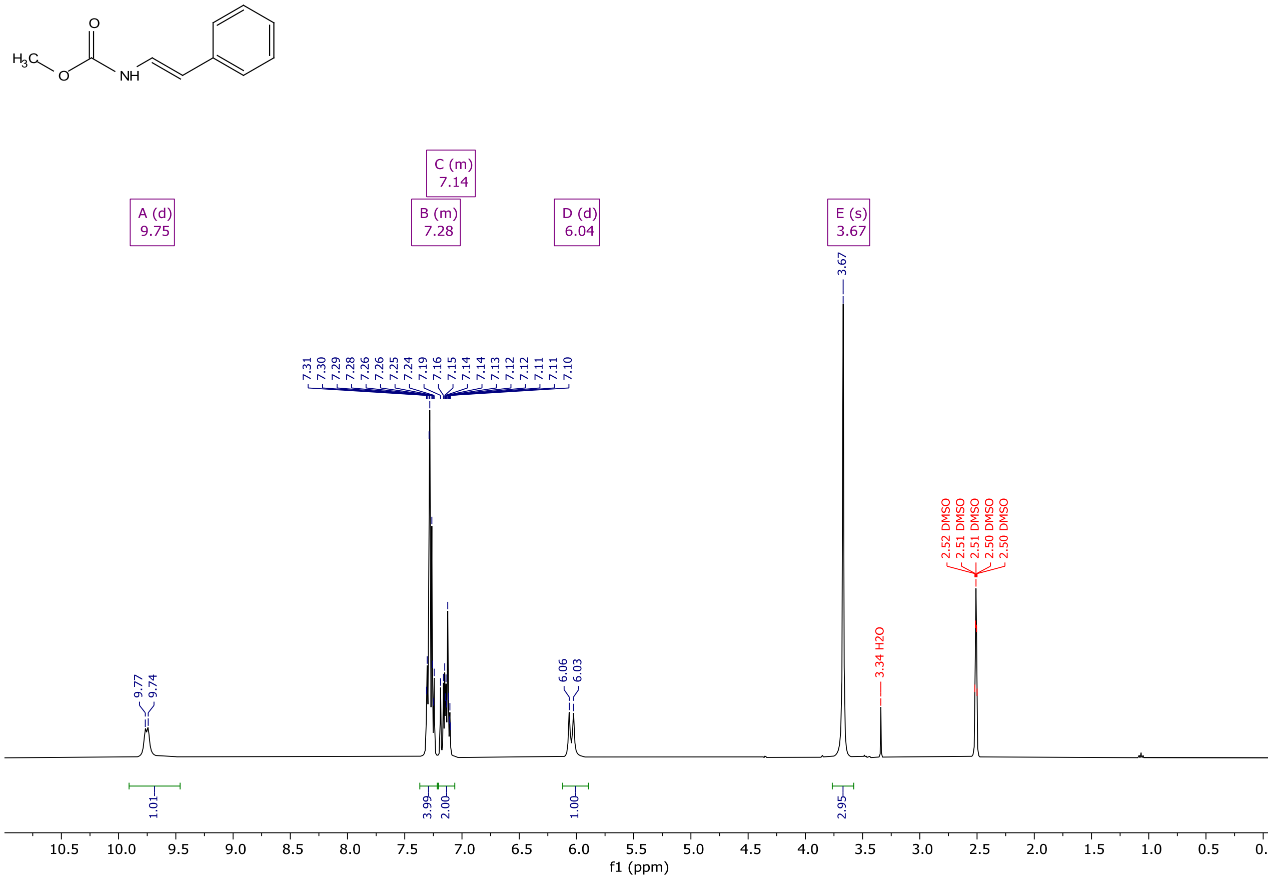

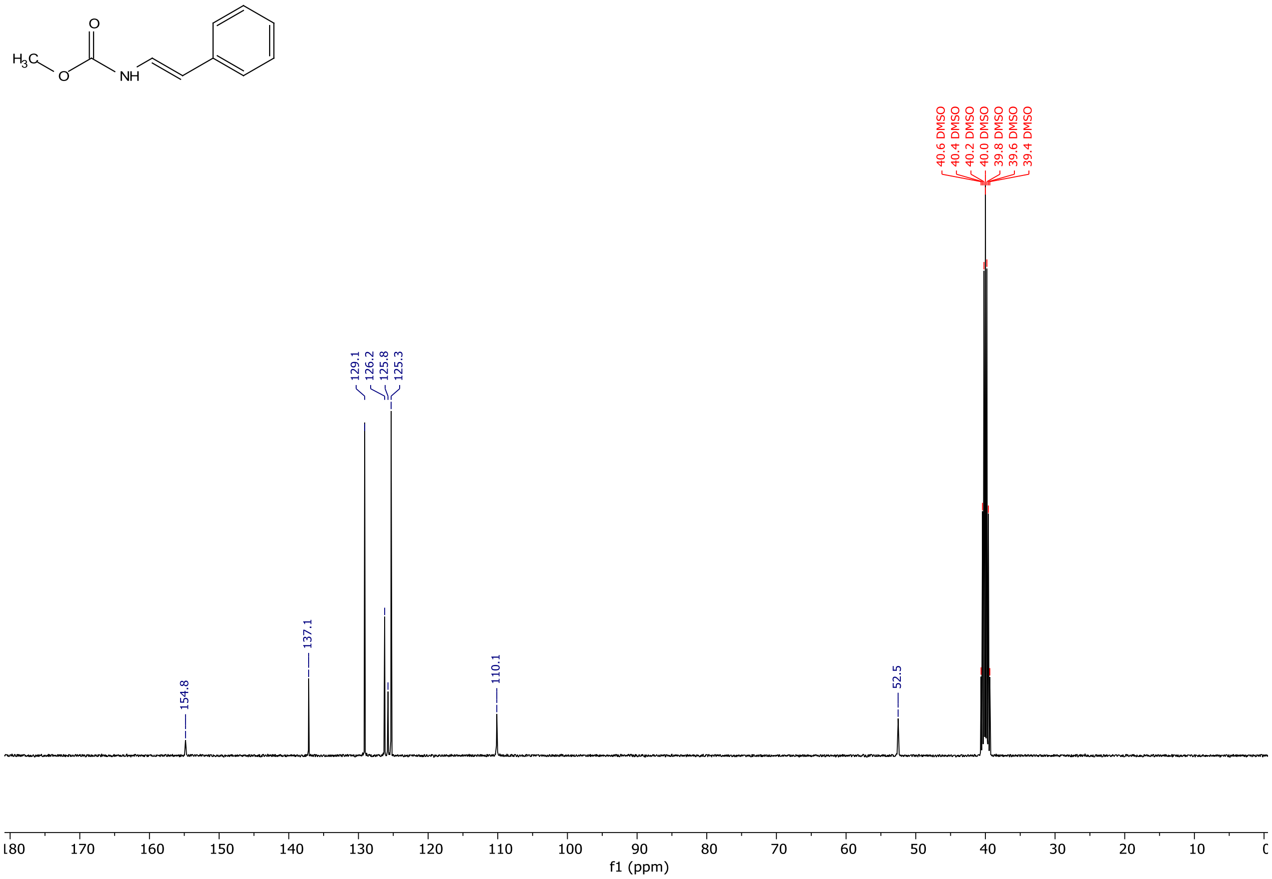

^{1}H and $^{13}C\{1H\}$ spectra of ***iso*-propyl *N*-phenyl carbamate** in DMSO-d_6.

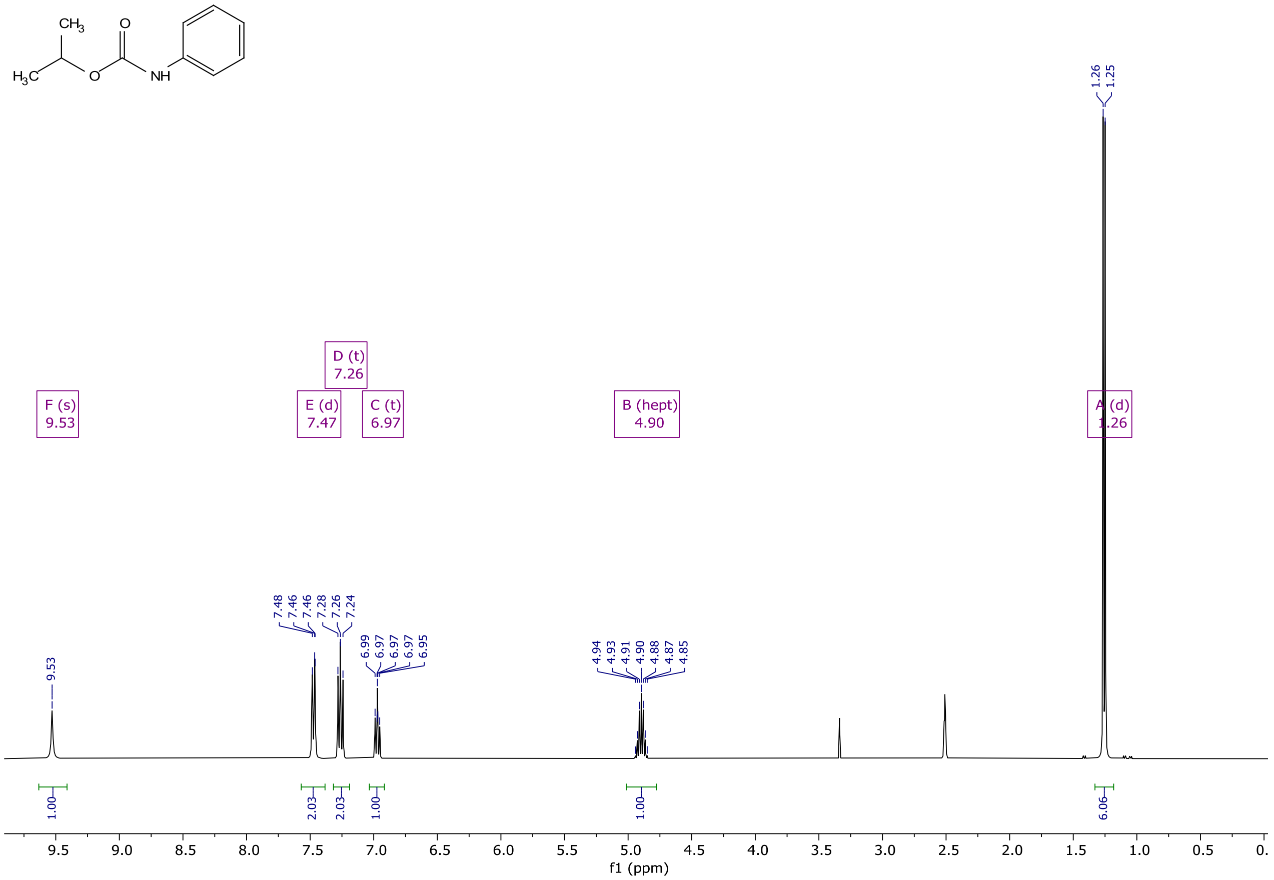

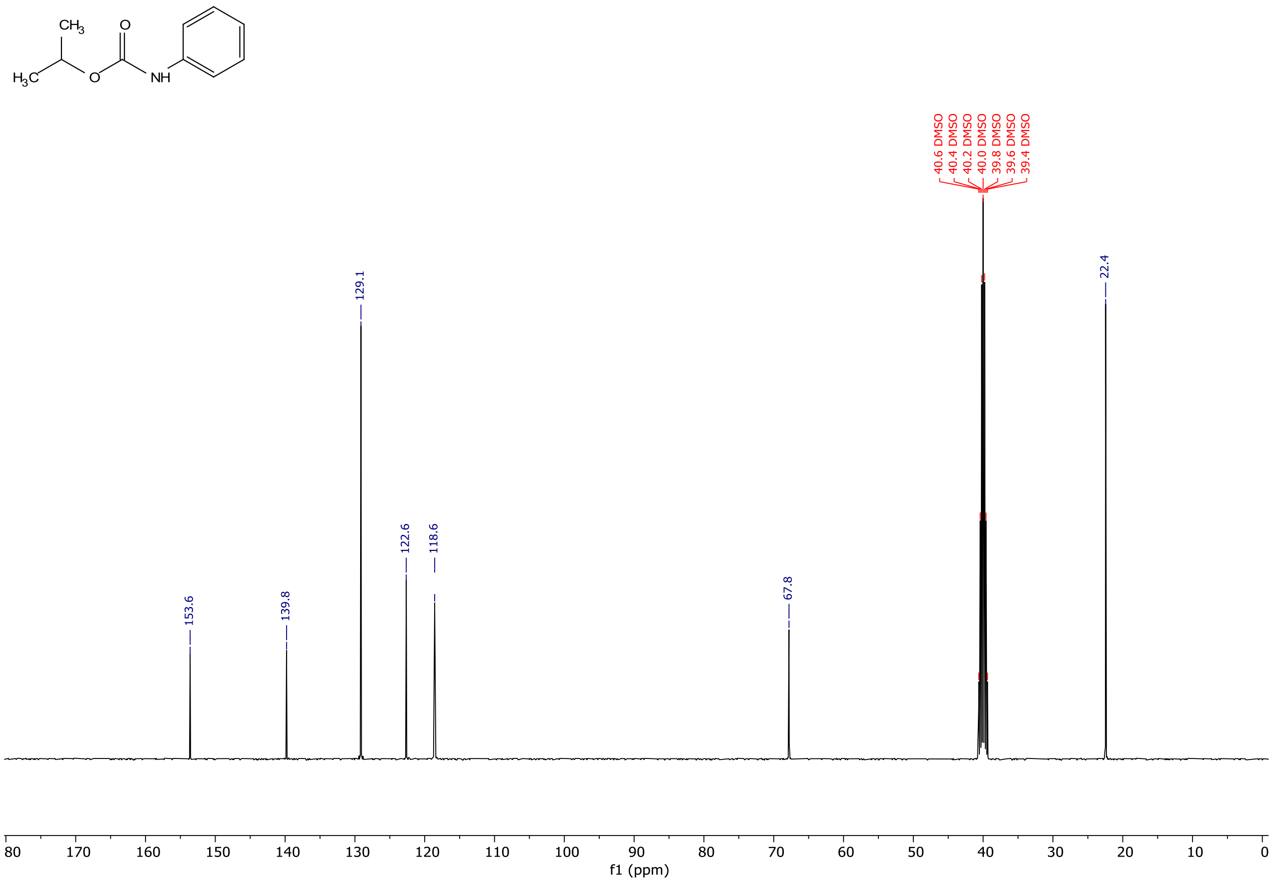

Supplement: RA-014-D4RA04521A-s001 [file RA-014-D4RA04521A-s001.pdf]
